# Genome-wide identification of multifunctional laccase gene family in cotton (*Gossypium spp.*); expression and biochemical analysis during fiber development

**DOI:** 10.1038/srep34309

**Published:** 2016-09-29

**Authors:** Vimal Kumar Balasubramanian, Krishan Mohan Rai, Sandi Win Thu, Mei Mei Hii, Venugopal Mendu

**Affiliations:** 1 Fiber and Biopolymer Research Institute (FBRI), Department of Plant & Soil Science, Texas Tech University, 2802, 15th street, Lubbock, TX, 79409, USA

## Abstract

The single-celled cotton fibers, produced from seed coat epidermal cells are the largest natural source of textile fibers. The economic value of cotton fiber lies in its length and quality. The multifunctional laccase enzymes play important roles in cell elongation, lignification and pigmentation in plants and could play crucial role in cotton fiber quality. Genome-wide analysis of cultivated allotetraploid (*G. hirsutum*) and its progenitor diploid (*G. arboreum* and *G. raimondii*) cotton species identified 84, 44 and 46 laccase genes, respectively. Analysis of chromosomal location, phylogeny, conserved domain and physical properties showed highly conserved nature of laccases across three cotton species. Gene expression, enzymatic activity and biochemical analysis of developing cotton fibers was performed using *G. arboreum* species. Of the total 44, 40 laccases showed expression during different stages of fiber development. The higher enzymatic activity of laccases correlated with higher lignin content at 25 DPA (Days Post Anthesis). Further, analysis of cotton fiber phenolic compounds showed an overall decrease at 25 DPA indicating possible incorporation of these substrates into lignin polymer during secondary cell wall biosynthesis. Overall data indicate significant roles of laccases in cotton fiber development, and presents an excellent opportunity for manipulation of fiber development and quality.

Cotton fiber is a single-celled seed trichome, developed from seed coat epidermal cells through four distinct, yet overlapping stages: 1) initiation (−3 to 5 DPA, days post anthesis); 2) elongation (5–25 DPA); 3) secondary cell wall (SCW) deposition (25–40 DPA) and 4) maturation (40–60 DPA). The widely cultivated cotton species, *G. hirsutum* (AD_1_-genome) is an allotetraploid originated from two diploid ancestor species, *G. arboreum* (A-genome) and *G. raimondii* (D-genome) through a natural hybridization and genome doubling process 1–2 MYA (Million Years Ago)^1^. Globally, cotton fiber is produced from *G. hirsutum* (~93–95%), *G. barbadense* (~5%) and *G. arboreum* (~2%) species^2^. The diploid *G. arboreum* is mainly cultivated in Indian subcontinent which accounts for ~16% of the total fiber production in South Asia^2^. Commercially cultivated white cotton fibers contain ~96% cellulose with less lignin (0.5–2.5%) and hemicelluloses (1.0–3.0%) however, naturally pigmented (brown and green) cotton fibers contain relatively less cellulose (~80%) and higher lignin (9–13%) and hemicelluloses (8.7–11%)^3^,^4^. Domestication, selection and breeding for white fibers have resulted in enhanced cellulose content with a simultaneous reduction in lignin, hemicellulose and phenolic compounds. The phenolic compounds and lignin content are believed to play an important roles in cotton fiber development and quality^5^.

Lignin is synthesized in specialized plant cells that undergo SCW deposition in addition to primary cell walls (PCW). Lignin, the second most abundant biopolymer is primarily composed of three canonical monomers namely, coniferyl (G), sinapyl (S) and *p*-coumaryl (H) alcohols^6^. Lignin monomers are synthesized in cytosol, exported to apoplastic region, oxidized and polymerized into lignin by a random coupling process. Laccases belong to multifunctional oxidase enzyme family, which carry out single electron oxidation of phenolic compounds resulting in resonance structures that undergo polymerization process^7^. Laccases are found to be predominant in fungal kingdom^8^,^9^ followed by bacteria^10^, plants^11^ and insects^12^. The plant laccases are involved in lignin^13^,^14^ and flavonoid^15^ biosynthesis while the microbial laccases are primarily involved in lignin breakdown^16^. Phenolic acids such as, benzoic (vanillic and sinapic acid) and cinnamic acid derivatives (*p*-coumaric, ferulic and caffeic acids) are part of lignin and flavonoid biosynthetic pathways^7^. These phenolic acids play an important role in cell elongation in plants^17^,^18^. *In vitro* studies showed laccase mediated oxidation of ferulic acid forms diferulate bridges between pectin polymers and arrest cell elongation indicating an important role of laccase enzymes in cell elongation through cell wall modification^19^.

Presence of wall linked phenolic acids such as ferulic, sinapic, vanillic and caffeic acids have been reported in *G. hirsutum* fibers and addition of 100 mM ferulic acid arrested fiber cell elongation *in vitro*^20^. In addition, phenolic compounds are associated with initiation^21^ and pigmentation of cotton fibers^22^. Laccases are associated with immature fiber phenotype^23^ and showed enhanced expression during fiber elongation and SCW biosynthetic stages^24^. Overall, emerging evidences indicate important roles of laccases in fiber development and quality through oxidation of phenolic compounds. Despite the importance of laccase genes in cotton fiber development and quality, there was no comprehensive report on laccase gene family in cotton. The present study focused on genome-wide identification and analysis of laccase genes in cultivated allotetraploid cotton (*G. hirsutum*) and its diploid progenitor species (*G. arboreum* and *G. raimondii)*. The identified laccase genes were further analyzed for gene architecture, conserved domain profile, physical properties (protein size, isoelectric point and subcellular localization), chromosomal location, phylogenetic relationships and synteny within the A-, D-, A^T^- and D^T^ genomes (^T^-tetraploid). Due to the presence large number of genes (84) in tetraploid *G. hirsutum* species and lack of fiber production in *G. raimondii*, the gene expression, enzyme activity and biochemical analysis (lignin and soluble/wall bound phenolic acids) were performed using *G. arboreum* fibers.

## Results

### Genome-wide identification of cotton laccase genes from tetraploid and its progenitor diploid species

The availability of *G. hirsutum*, *G. arboreum* and *G. raimondii* genome sequences facilitated identification and analysis of laccase gene family from cultivated tetraploid and its diploid progenitor species^25^,^26^,^27^. The total coding and protein sequences of *G. hirsutum*, *G. arboreum* and *G. raimondii* genomes were downloaded to identify laccase gene family in these cotten species. Total proteomes of three cotton species were searched for laccase family members using blastP similarity search program (Supplementary Table S1). A total of 44 laccase proteins were identified from *G. arboreum* using *Arabidopsis* laccase protein sequences as query (Table 1, Supplementary Table S2). Similarly, 46 and 84 laccase proteins were identified from *G. raimondii* and *G. hirsutum*, respectively (Supplementary Table S2). The identified proteins were validated using NCBI conserved domain database for the presence of laccase specific conserved domains i.e. laccase (TIGR03389), CuRO_1_LCC_plant (cd13849), CuRO_2_LCC_plant (cd13875) and CuRO_3_LCC_plant (cd13897) in these proteins (Supplementary Table S3). These proteins were further confirmed using InterProScan, which showed presence of multicopper oxidase type 1 (IPR001117), multicopper oxidase type 2 (IPR011706), multicopper oxidase type 3 (IPR011707) and laccase (IPR017761) domains (Supplementary Table S3). All the identified laccase proteins from *G. arboreum* were named based on orthologous similarity with *Arabidopsis* laccase protein sequences (Table 1). The *G. raimondii* and *G. hirsutum* laccase proteins were named according to their phylogenetic closeness to *G. arboreum* laccases (Supplementary Table S4). Laccase groups with multiple members (eg. *GaLAC04*) were further classified as members within the group and numbered according to their phylogenetic closeness (eg. *GaLAC04_1* to *GaLAC04_8*).

### Analysis of conserved domain, gene architecture and subcellular localization of laccases

**Figure 1:**
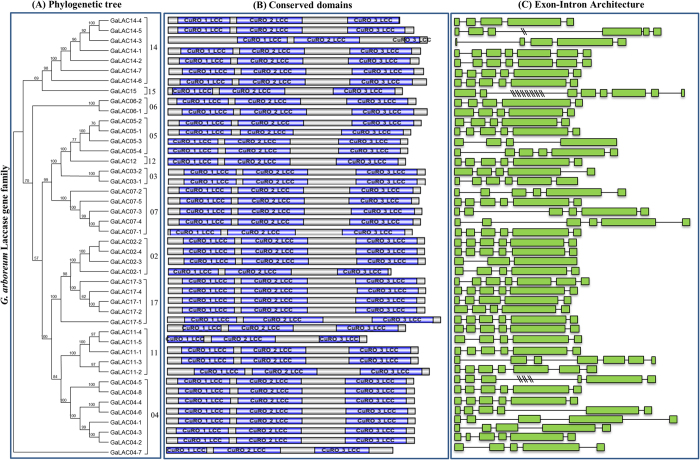
Phylogenetic, conserved domain and gene architecture analysis of *G. arboreum* laccases. (**A**) The phylogenetic tree of 44 *G. arboreum* laccase proteins. (**B**) Characteristic conserved domains present in the laccase proteins [CuRO_1_LCC_plant (cd13849), CuRO_2_LCC_plant (cd13875) and CuRO_3_LCC_plant (13897)] (**C**) Exon-intron architecture of *G. arboreum* laccase genes. Green boxes represent exons whereas introns are represented with black lines. Big introns were compressed in order to fit within space where one double slash (\\) represents reduction of 1 Kb.

The conserved domain architecture of laccase proteins was analyzed using NCBI’s conserved domain database and InterProScan. Out of 44 proteins of *G. arboreum*, four characteristic domains (InterProScan: laccase, IPR001117, IPR011706 and IPR011707; NCBI CDD: laccase, CuRO_1_LCC, CuRO_2_LCC and CuRO_3_LCC) were present in all the identified proteins (Fig. 1, Supplementary Table S3). Similar pattern of conserved domains were present in *G. raimondii* and *G. hirsutum* laccase proteins (Supplementary Fig. S1A–C, Supplementary Table S3). Further, the exon-intron organization of laccase genes was analyzed by comparing coding sequences of laccase genes with their respective genomic sequences. The number of exons showed variation across the three species ranging from 2–8 (*G. arboreum,* 3–8; *G. raimondii,* 4–7 and *G. hirsutum,* A-subgenome 4–7, D-subgenome 2–7) (Fig. 1, Supplementary Fig. S1A–C). An average exon number of 5.75, 5.82, 5.85 and 5.7 was observed in A, D, A^T^-subgenome and D^T^-subgenome, respectively. All 44 laccase orthologous genes identified from *G. arboreum* were further analyzed *in silico* for their genomic and physical characteristics such as chromosomal location, gene size (genomic and coding sequence), protein molecular weight, pI (isoelectric point), and subcellular localizations (Table 1). Comparison of gene length among *G. arboreum* laccases showed *GaLAC14_3* as the longest (11.061 kb) and *GaLAC05_3* (1.994 kb) as the smallest based on genomic sequences while *GaLAC017_5* (1.842 kb) as the longest and *GaLAC11_5* (1.35 kb) as the smallest genes based on coding sequences (Table 1). The predicted molecular weight and isoelectric points of laccase proteins were found to be 49.982-68.113 kDa (pI, 4.76–9.85) in *G. arboreum*, 45.179–65.935 kDa (pI, 4.66–9.87) in *G. raimondii*, 30.954 to 66.534 kDa (pI, 4.98–9.89) in *G. hirsutum* A-subgenome and 36.416 to 66.56 kDa (pI, 4.84–9.87) in *G. hirsutum* D-subgenome (Table 1, Supplementary Table S2). The subcellular localization pattern of cotton laccase proteins were computationally predicted using online tool TargetP 1.1. The subcellular localization prediction showed majority of laccase proteins as secretory (33/44 *G. arboreum*; 43/46 *G. raimondii*; 80/84 *G. hirsutum*) and some as non-secretory (8/44 *G. arboreum*; 2/46 *G. raimondii*; 3/84 *G. hirsutum*) while a few others as mitochondrial localized (3/44 *G. arboreum*; 1/46 *G. raimondii*; 1/84 *G. hirsutum*) (Table 1, Supplementary Tables S2 and S5).

### Phylogeny, chromosomal distribution and synteny analysis

**Figure 2:**
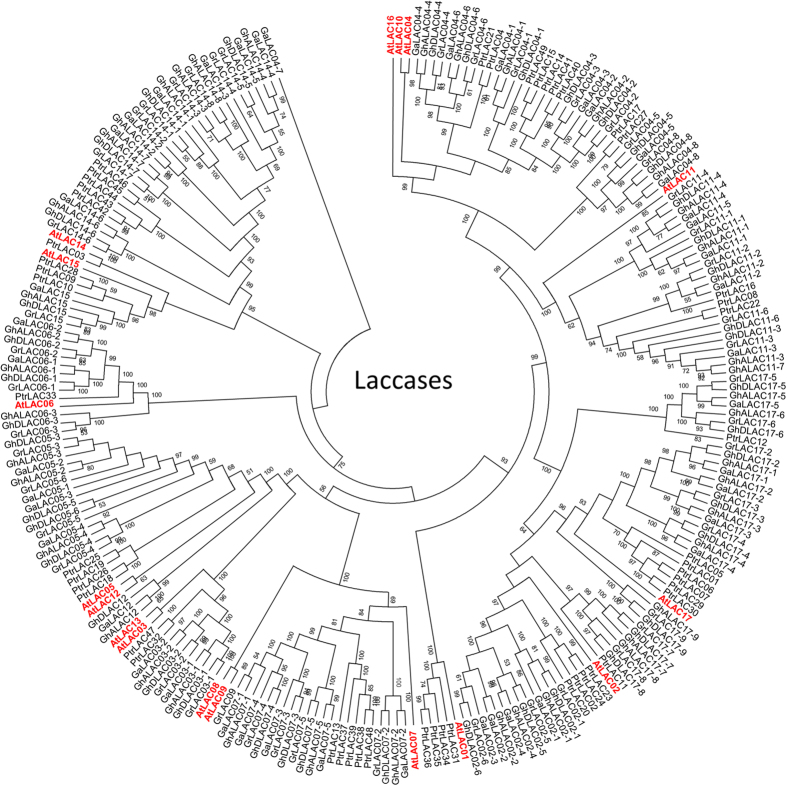
Phylogenetic analysis of cotton laccase gene family. Phylogenetic tree was constructed using 240 protein sequences from *Gossypium arboreum* (44), *G. raimondii* (46), *G. hirsutum* A-subgenome (42), *G. hirsutum* D-subgenome (42), *Arabidopsis thaliana* (17) and *Populus trichocarpa* (49). MEGA v6.2. Neighbor-joining method was used with boot strap replication of 1000 times to create the phylogenetic tree. Arabidopsis laccases are highlighted with red colored text.

**Figure 3:**
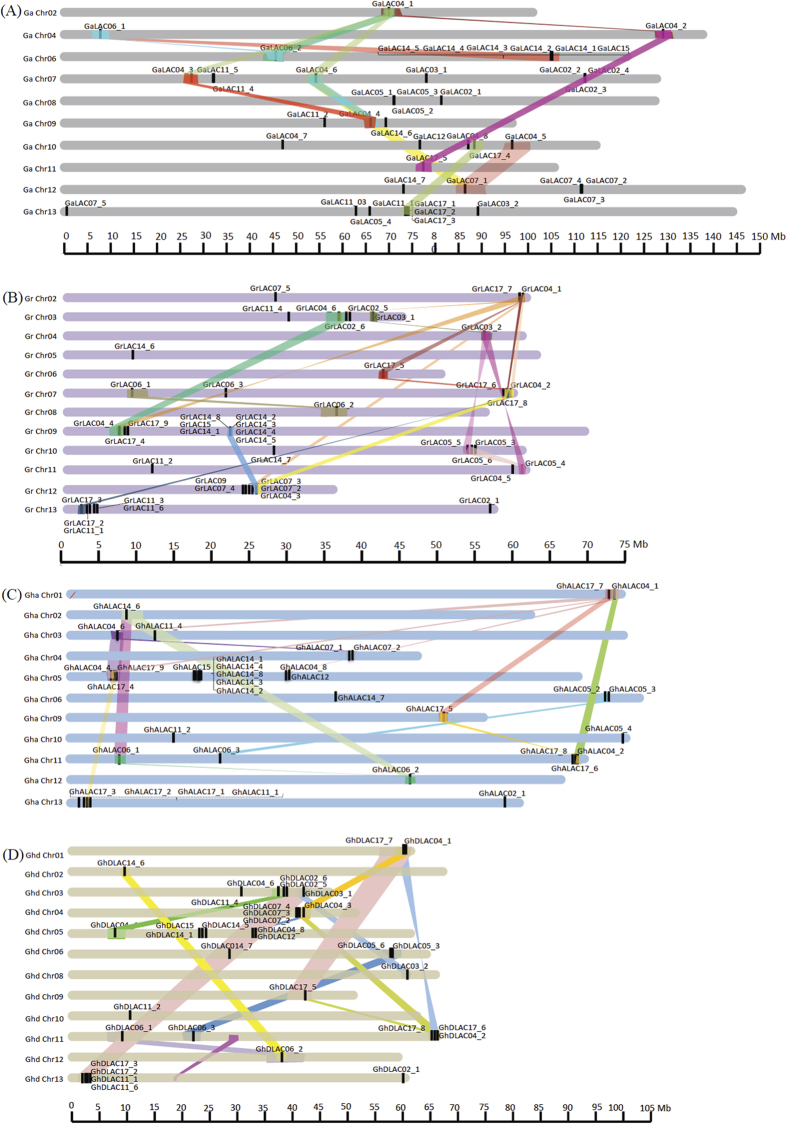
Chromosomal localization and synteny mapping of cotton laccase gene family. (**A**) *G. arboreum* (A-genome) (**B**) *G. raimondii* (D-genome) (**C**) *G. hirsutum* A-subgenome (**D**) *G. hirsutum* D-subgenome. Colored lines represent the syntenic relationships of laccase genes within genome. Chromosomes containing laccase gene(s) were represented in the figure.

Phylogenetic analysis was performed using 240 laccase protein sequences from five different plant species [*A. thaliana* (17), *P. trichocarpa* (49), *G. arboreum* (44), *G. raimondii* (46), *G. hirsutum* A-subgenome (42) and *G. hirsutum* D-subgenome (42)]. The constructed phylogenetic tree revealed clustering of cotton laccase proteins with *Arabidopsis* and poplar laccases into 12 different clades/groups (LAC02, LAC03, LAC04, LAC05, LAC06, LAC07, LAC09, LAC11, LAC12, LAC14, LAC15 and LAC17) (Fig. 2, Supplementary Fig. S2 and Supplementary Table S4). Laccases from all three cotton species represented in 10 of the 12 clades and the number of laccases within clades/groups varied from 1 (LAC09, LAC12 and LAC15) to 9 (LAC17) (Fig. 2, Supplementary Table S4). Together with conserved domain analysis, these results confirmed the conserved nature of laccase proteins across different plant species. Chromosomal distribution analysis of the identified laccase genes was performed in three cotton species. In *G. arboreum*, the identified 44 laccase genes were distributed across 10 chromosomes except chromosome numbers 1, 3 and 5 (Fig. 3A). The *G. arboreum* chromosomes 7 and 13 contained highest number of laccase genes, with 8 on each, while, chromosomes 2 and 11 showed least number of laccase genes with only one on each (Fig. 3A). Similarly, in *G. raimondii*, the identified 46 laccase genes were found to be distributed across all the chromosomes except on chromosome 1 (Fig. 3B) with highest (11) on chromosomes 9 and lowest (1 each) on chromosomes 4, 5, 6 and 8 (Fig. 3B). In *G. hirsutum* A-subgenome, laccase genes were found on all the chromosomes except chromosomes 7 and 8. In *G. hirsutum* A-subgenome, chromosome 5 has the highest number (11) of laccase genes whereas seven of 42 laccase genes were found to be present on scaffolds related to 3^rd^, 8^th^ and 13^th^ chromosomes (Fig. 3D). Laccase genes of *G. hirsutum* D-subgenome were distributed among 12 chromosomes except chromosome 7 while chromosome 5 showed highest number (6) of laccase genes (Fig. 3D). Six of 42 laccase genes from D-subgenome were found to be present on scaffolds related to 5^th^, 6^th^, 10^th^ and 13^th^ chromosomes (Fig. 3C). Tandem duplication analysis among laccase gene family members in three species showed, a total of 5 tandem duplication events involving 13 genes, 6 events involving 19 genes, 6 events involving 14 genes and 4 events involving 10 genes in *G. arboreum*, *G. raimondii*, *G. hirsutum* A-subgenome and D-subgenome, respectively (Fig. 3, Table 1 and Supplementary Table S2). Similarly, 12, 17, 14 and 12 independent segmental duplications of laccase genes were identified in *G. arboreum*, *G. raimondii*, *G. hirsutum* A- and D-subgenomes, respectively (Fig. 3A–D).

### Laccases with miRNA target sites in *G. arboreum*

Laccase gene expression has been shown to be post-transcriptionally regulated by three different miRNA families in *Arabidopsis*^28^. To predict the role of miRNAs in regulating cotton laccase genes, all 44 genes from *G. arboreum* have been analyzed for the presence of miRNA target sites. The three reported laccase targeting miRNAs (miR397, miR408a and miR857) from *Arabidopsis, Brachypodium,* poplar, rice, corn and sorghum (Supplementary Table S6) were used as query sequences to predict the target sites on cotton laccase genes using online target prediction tool psRNATarget with stringent criteria. Out of 44 laccase genes, 22 were predicted to have miRNA target sites for either miR397 (miR397a and miR397b) or miR408a (Table 2, Supplementary Table S6). Among the 22 targeted genes, 18 were targeted by miR397 family, 4 were targeted by miR408, while none were predicted to be targeted by miR857 (Table 2).

### Expression of laccase genes during *G. arboreum* fiber development

**Figure 4:**
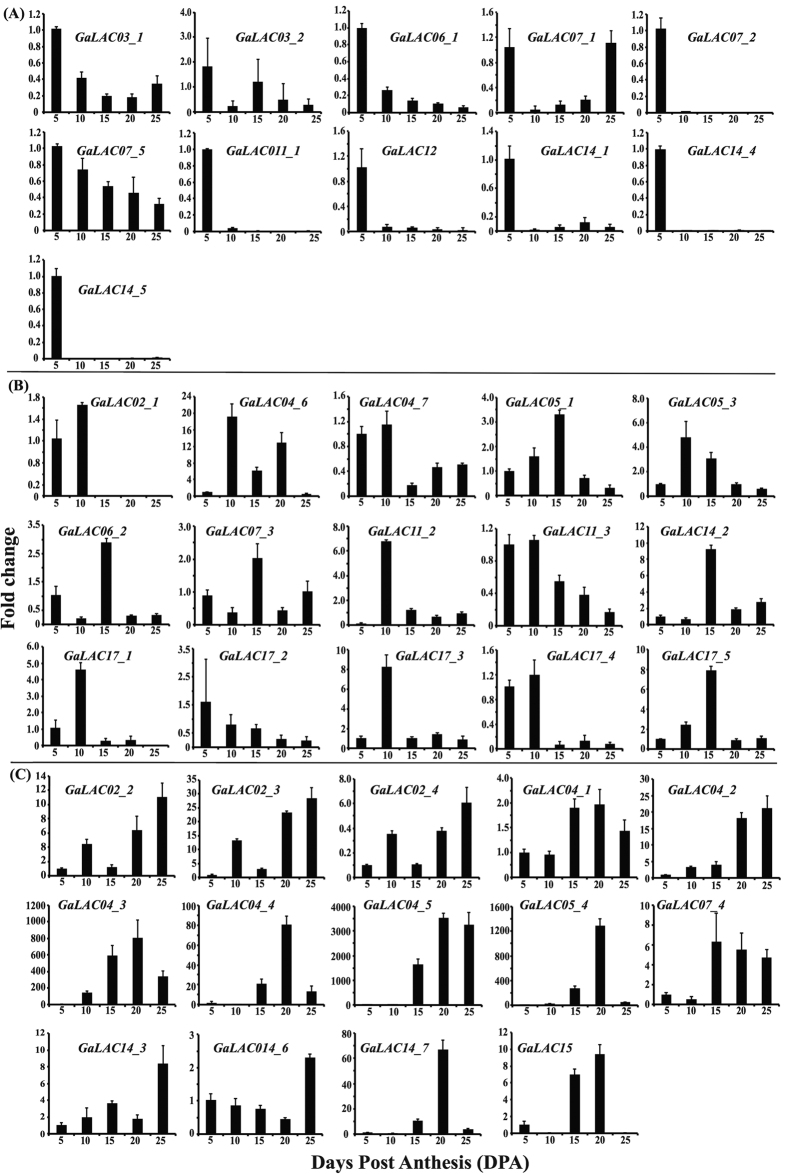
Relative mRNA expression of *G. arboreum* laccase genes during fiber development. (**A**) A total of 11 out of 40 laccases are expressed during active phase of fiber elongation (5 DPA). (**B**) A total of 15 out of 40 laccases are expressed during transition/onset of SCW development of fiber (10 and 15 DPA). (**C**) A total of 14 out of 40 laccases are expressed during active phase of SCW development of fiber (20 and 25 DPA). *GaHistone-3b* was used as internal control to normalize the expression data. Error bars represent difference in expression pattern between technical replicates.

The lignin and phenolic acid content and composition are influenced by several factors including spatio-temporal expression of monolignol biosynthetic and laccase genes^13^, hence it is important to analyze the laccase gene expression during fiber development. The present study performed gene expression analysis of 44 laccase genes at five developmental stages of *G. arboreum* cotton fiber; elongation stage (5 DPA), transition stage (10 and 15 DPA) and SCW deposition stage (20 and 25 DPA). Out of 44 laccase genes, four (*GaLAC11_5, GaLAC11_4, GaLAC05_2* and *GaLAC04_8*) were not detectable in any of the five fiber developmental stages (Fig. 4) while, 40 genes showed differential expression during cotton fiber development. The gene expression (fold change) was calculated using ∆∆Ct method. In order to compare expression of individual gene across different fiber developmental stages, the individual gene expression was normalized to 5 DPA. Hence the fold change is a relative higher or lower expression of a particular gene in relation to its expression at 5 DPA. The highly abundant laccase genes at different developmental stages were identified based on their relative abundance at a particular stage compared to all other stages. The abundant genes at different stages are described in the following sub-headings.

#### Laccase genes with higher relative expression during elongation stage (5 DPA)

Since laccase genes are involved in cell elongation it is important to identify the genes that are highly expressed during cotton fiber elongation stage. The elongation stage specific gene expression analysis showed highest expression of 11 laccases at 5 DPA compared to other stages (Fig. 4A). The highly expressed genes were *GaLAC03_1, GaLAC03_2, GaLAC06_1, GaLAC07_1, GaLAC07_2, GaLAC07_5, GaLAC11_1, GaLAC12, GaLAC14_1, GaLAC14_4* and *GaLAC14_5*. Among these genes, the *LAC11* group is involved in lignin deposition during SCW biosynthesis in *Arabidopsis*; however, the functions of *LAC03, LAC06, LAC07, LAC12* and *LAC14* have not been characterized. Further the 11 abundant laccases were compared for their relative expression levels at 5 DPA by normalizing with the lowest expressed gene (*GaLAC03_2*) and presented as log_10_ fold change in expression (Supplementary Fig. S3). The comparative expression among abundant 5 DPA laccases showed a higher expression of *GaLAC14_4* and *GaLAC14_5* followed by *GaLAC11_1, GaLAC06_1, GaLAC07_2* and *GaLAC03_1*.

#### Laccase genes with higher relative expression during transition/onset of SCW stage (10 and 15 DPA)

Plant cell wall elongation arrest plays an important role in determining the cotton fiber length. Phenolic compounds such as ferulic and *p*-coumaric acids, which are oxidized by laccases, are involved in cell wall elongation arrest. The expression pattern of laccases during transition stage of cotton fiber development, which marks the cessation of elongation and initiation of secondary cell wall biosynthesis helps to identify laccases involved in the cell wall elongation arrest. At 10 and 15 DPA, 15 laccase genes showed higher relative expression compared to other stages (Fig. 4B). The highly expressed laccases were *GaLAC02_1, GaLAC04_6, GaLAC04_7, GaLAC05_1, GaLAC05_3, GaLAC06_2, GaLAC07_3, GaLAC11_2, GaLAC11_3, GaLAC14_2, GaLAC17_1, GaLAC17_2, GaLAC17_3, GaLAC17_4* and *GaLAC17_5*. Among these laccases*, LAC04, LAC11* and *LAC17* have a major role in lignin deposition during secondary cell wall biosynthesis in *Arabidopsis*; however, the roles of *LAC05, LAC06* and *LAC07* have not been characterized. Among the 15 abundant 10 DPA laccases, the comparative expression analysis showed *GaLAC11_2* to be highly expressed followed by *GaLAC05_3, GaLAC04_6* and *GaLAC05_1* while *GaLAC02_1* was the least expressed at 10 DPA (Supplementary Fig. S3). The comparative expression of abundant laccases at 15 DPA showed higher expression of *GaLAC11_2* and *GaLAC14_2,* followed by *GaLAC05_1, GaLAC05_3, GaLAC17_5* and *GaLAC04_6,* while *GaLAC17_2* showed least expression (Supplementary Fig. S3)*. GaLAC11_2,* involved in lignin biosynthesis in *Arabidopsis,* showed higher expression among abundant genes both at 10 and 15 DPA.

#### Laccase genes with higher relative expression during active SCW stage (20 and 25 DPA)

The role of laccases in lignin deposition during SCW biosynthesis in plants is very well known. Interestingly, the cotton fiber, which deposits large amounts of cellulose during SCW biosynthesis, shows minimal lignin deposition. It is interesting to study the expression pattern of laccase genes during the SCW biosynthetic stage in cotton fiber to understand their role in fiber development. Gene expression analysis across different developmental stages showed (Fig. 4C) a higher relative expression of 14 laccase genes at SCW stage (*GaLAC02_2, GaLAC02_3, GaLAC02_4, GaLAC04_1, GaLAC04_2, GaLAC04_3, GaLAC04_4, GaLAC04_5, GaLAC05_4, GaLAC07_4, GaLAC14_3, GaLAC14_6, GaLAC14_7* and *GaLAC15)*. Among these highly abundant laccases, *LAC04* group is involved in lignification of secondary cell wall and *LAC15* group is involved in proanthocyanidin formation in seed coats of *Arabidopsis*. The comparative expression analysis among the 14 abundant laccases at 20 DPA showed *GaLAC04_2* to be highly expressed followed by *GaLAC04_3, GaLAC04_5* and *GaLAC05_4,* while *GaLAC14_3* was the least expressed (Supplementary Fig. S3). At 25 DPA, the comparative expression analysis among highly abundant laccases showed *GaLAC04_2* to be highly expressed followed by *GaLAC04_5, GaLAC04_3* and *GaLAC14_6* while *GaLAC04_4* was the least expressed laccase at 25 DPA (Supplementary Fig. S3). The SCW stage showed lignification and proanthocyanidin related laccase expression apart from other laccases with unknown functions.

#### Overall laccase gene expression during cotton fiber development (5, 10, 15, 20 and 25 DPA)

Laccases are involved in free radical formation of monolignols during lignin biosynthesis. Individual laccases may have same or different substrates for free radical formation, however all the free radicals must be produced simultaneously to be incorporated into the cell wall or lignin polymer. Hence combined expression is important for lignin deposition during SCW biosynthesis. In order to understand the overall role of laccases during different developmental stages of cotton fiber, the combined expression level of all 40 laccases was calculated (Supplementary Fig. S4). The results indicate an 8 fold increase in laccase gene expression between 5 and 10 DPA, 8 fold further increase in expression between 10 and 15 DPA and an additional doubling from 15 DPA to 20 DPA followed by 0.6 fold decrease at 25 DPA. Overall expression of laccases increased during the cotton fiber development, which is consistent with their primary role in secondary cell wall biosynthesis.

### Detection of laccase activity in developing *G. arboreum* fibers

**Figure 5:**
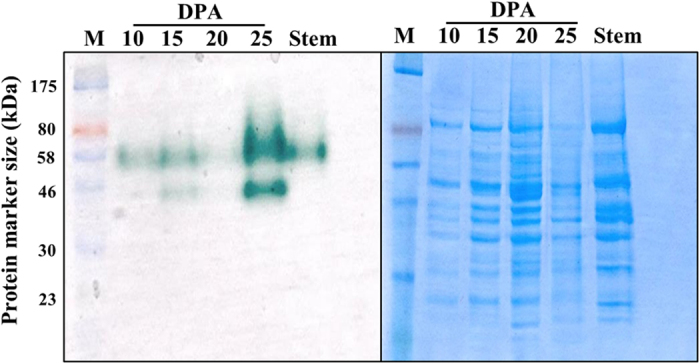
Laccase enzyme activity assay in developing *G. arboreum* fibers. Left panel: Laccase enzyme activity was assayed using 1-D Zymography. 50 μg of total proteins from 10, 15, 20 and 25 DPA fibers along with control (stem), were run on non-denaturing SDS-PAGE gel, incubated in Sodium tartarate buffer and later in ABTS solution to visualize laccase activity. Right panel: Loading control gel ran with same amounts of total proteins and stained with Coomassie Brilliant Blue. M: represents protein marker.

Zymography is an efficient technique to identify the enzyme activity using non-denaturing native PAGE (Poly Acrylamide Gel Electrophoresis) with suitable substrate and optimum activity conditions^29^. Laccase activity was measured using zymography technique with ABTS [2,2′-Azinobis (3-ethylbenzothiazoline-6-sulfonic acid] as a substrate^30^. Fifty micrograms of total protein each from four different stages (10, 15, 20 and 25 DPA) of *G. arboreum* fibers was used to measure laccase enzyme activity using 1-dimensional zymogram. Laccase activity was detected in all four stages including positive control (stem sample of *G. arboreum*). Laccase activity showed a dynamic pattern with relatively moderate activity at 10 and 15 DPA followed by a weaker activity at 20 DPA and a sudden increase in activity at 25 DPA (Fig. 5). The activity at 25 DPA represents a combined activity of all laccases and not of an individual laccase enzyme. The presence of two activity bands (approximate size of 46 and 60 kDa) indicates presence of different laccase enzymes with different sizes, which is consistent with predicted protein sizes (Table 1). The laccase activity is consistent with laccase role in lignin deposition during SCW stage.

### Increase in lignin content during fiber development

**Figure 6:**
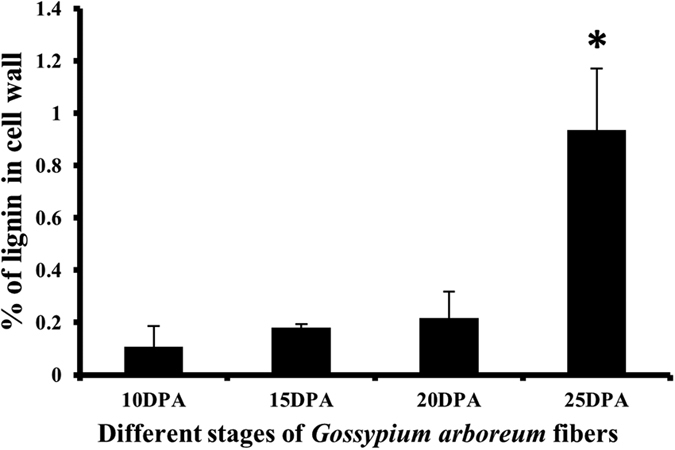
Lignin content of different developmental stages of *G. arboreum* fiber. Lignin was extracted from different stages of cotton fiber development (10, 15, 20 and 25 DPA) and estimated using TGA method (UV absorbance at 280 nm). Industrial lignin was used for standard curve preparation to calculate lignin content in cotton fibers. Asterisk indicates significant difference in lignin quantity (t-test; p < 0.05).

Lignification process involves the coordinated expression of phenylpropanoid pathway genes for the monolignol biosynthesis and laccases for free radical formation^6^,^31^. Since laccases are involved in lignin formation, we estimated the lignin content in different stages of cotton fiber development. Estimation of lignin content using Thioglycolic acid (TGA)^32^ method showed an overall increase in lignin content during fiber development (Fig. 6). The percentage of lignin in dried cell wall was found to be 0.1–0.2% of cell wall weight during 10 and 15 DPA with no increase at 20 DPA. Relatively, a significant increase in lignin content (1%) was observed during SCW biosynthetic stage at 25 DPA (Fig. 6). A similar range of cotton fiber lignin content (0.12–2.6%) has been reported in *G. hirsutum* based on Thioglycolate, Acetyl bromide and Klason estimation methods^3^.

### Estimation of phenolic acid content during *G. arboreum* fiber development

**Figure 7:**
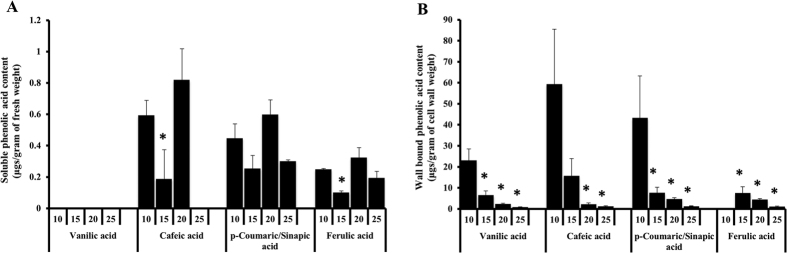
Soluble and wall bound phenolic acid content during *G. arboreum* fiber development. (**A**) Soluble phenolic acid content was represented as μg/gram of fresh fiber weight. (**B**) Wall bound phenolic acid content was represented as μg/gram of cell wall weight. Soluble and wall bound phenolic acids were extracted from different developmental stages (10, 15, 20 and 25 DPA) and analyzed using HPLC with C18 column. Mobile phases composed of 33% A (100% acetonitrile) and 67% B (ultra-pure water pH: 2.1) at a flow rate of 1.5 ml/minute. Standards for ferulic acid, *p-*coumaric acid, caffeic acid, sinapic acid and vanillic acid were used at 1 mg/ml concentration. 1-Napthalene acetic acid (1 mg/ml) was used as an internal control to calculate the phenolic acid content of cotton fibers. Asterisk indicates significant difference in phenolic acid levels (t-test; p < 0.05).

Phenolic acids like caffeic acid, sinapic acid, vanillic acid and ferulic acid are substrates of laccases, which are ester linked to cell wall polysaccharides in developing cotton fiber^3^. These phenolic compounds are known to influence the cell elongation arrest and other cell wall properties in plants^17^,^18^. The wall bound and soluble phenolics were extracted from cotton fibers and estimated using HPLC (High Performance Liquid Chromatography). The absolute quantification of different compounds was calculated using internal control and the retention time of individual phenolic compounds were established by respective standards. The retention time of *p*-coumaric acid and sinapic acid standards were overlapping hence could not be distinguished, therefore represented as combination of *p-*coumaric/sinapic acids (Fig. 7 and Supplementary Fig. S5). The soluble phenolic compounds did not show any statistically significant variation in abundance during fiber development while the wall bound phenolics showed a clear statistically significant decrease in abundance with fiber development. The vanillic acid could not be detected in soluble phenolic fraction in any of the cotton fiber developmental stages (Fig. 7A). The sum of four soluble phenolic acids showed a dynamic change in the abundance (decreased from 10 DPA to 15 DPA and increased in 20 DPA followed by a decrease in 25 DPA) (Supplementary Fig. S6A). The analysis of wall bound phenolics showed a clear pattern of decrease in abundance with the fiber development (5 DPA towards 25 DPA) (Fig. 7B; Supplementary Fig. S6B). A similar pattern of decrease in wall bound phenolic acid abundance was reported in developing fibers of *G. hirsutum*^5^. Ferulic acid showed an interesting pattern during fiber development. At 10 DPA, ferulic acid was detectable in soluble fraction but not in wall bound fraction; however, appeared at 15 DPA onwards in wall bound fraction (Fig. 7A,B).

## Discussion

The multifunctional laccase enzymes are involved in cell elongation^19^, pigmentation^15^ and SCW biosynthesis^13^,^33^ in *Arabidopsis*. Cotton fiber, which undergoes a similar process including pigmentation, serves as an excellent single cell model system to study the functions of laccases. The established primary role of laccases in plants is lignin deposition during SCW biosynthesis which accounts for 20–30% of total plant biomass^34^; however, the lignin content of cotton fiber, which also undergoes SCW biosynthetic process is relatively very low (0.12–2.6%)^3^. The abundance and dynamic expression of different laccases at different stages of *G. arboreum* fiber development indicate a potential role of laccases in oxidizing phenolic compounds that are involved in other developmental functions besides cotton fiber lignin biosynthesis. The present study is focused on studying the relation between laccase gene expression, enzymatic activity and biochemical analyses during cotton fiber development.

### Genome-wide identification and analysis of cotton laccase gene family

Genome-wide analysis of diploid *G. arboreum*^26^ and *G. raimondii*^27^ and allotetraploid *G. hirsutum*^35^ resulted in the identification of 44, 46 and 84 genes, respectively (Table 1, Supplementary Tables S1 and S2). The number of laccases in allotetraploid cotton was not equal to the sum of progenitor species, which is likely due to either gene loss in tetraploid species or gain in progenitor species after polyploidization event. The exon frequency (5–6 exons/gene) and largest exon (second last exon) analysis showed a similar pattern in cotton and *Arabidopsis* laccase genes indicating a conserved nature of gene structure between these two plant species (Fig. 1, Supplementary Fig. S1). The four signature conserved domains (laccase, CuRO_1_LAC, CuRO_2_LAC and CuRO_3_LAC ) of cotton laccases were similar to *Arabidopsis* laccases^11^, which also shows a highly conserved nature of laccase proteins and potential functions. Further, phylogenetic analysis of *Arabidopsis*, poplar and cotton laccases showed presence of cotton laccases in 12 out of 17 clades (Fig. 2). The phylogenetic clustering of cotton laccases further supports the orthology based nomenclature (Fig. 2 and Supplementary Table S4). Chromosomal localization analysis showed that laccase genes were distributed on almost all chromosomes within the genomes of three cotton species (Fig. 3). Though there are more numbers of laccase genes in cotton species, they are found to be orthologues of only 12 out of 17 *Arabidopsis* laccases. The synteny analysis showed that the increase in number of laccase genes in cotton was a result of segmental and tandem duplication events during cotton evolution (Fig. 3).

The physical properties of laccases such as molecular mass and isoelectric point showed variation between diploid and tetraploid species. The molecular mass of laccases in diploid progenitor cotton species is higher than tetraploid subgenomes indicating evolutionary preference of tetraploid species towards smaller laccase proteins. The isoelectric point (pI) determines the solubility, subcellular localization and interactions^36^,^37^ of proteins, interestingly majority (95.2%) of cotton laccases are basic proteins (pI > 7) while a very small percentage of them are acidic (pI < 7) which indicates common physical properties of majority of laccases (Table 1 and Supplementary Table S2). In addition, *in silico* analysis predicted that majority of cotton laccases as secretory proteins, similar to *Arabidopsis*^11^ laccases, which is consistent with their physical properties and primary role in cell wall biosynthesis. However, some laccase proteins are predicted to be non-secretory and while a few others are predicted to be mitochondrial localized. The function of non-secretory and mitochondrial laccases is not known in plants, characterization of these proteins might reveal novel functions, particularly the mitochondrial laccases.

### Gene expression analysis during cotton fiber development

Laccases are multigene and multifunctional genes with different physical properties and localization patterns. To understand their role in cotton fiber development, it is important to study the expression pattern during different stages of cotton fiber development. Analyses of laccase gene expression of five different developmental stages of cotton fiber revealed differential expression of laccase genes during cotton fiber development. Active fiber elongation stage (5 DPA) showed a relatively higher expression of 6 out of 12 laccase groups (*GaLAC03, GaLAC06, GaLAC07, GaLAC11, GaLAC12* and *GaLAC14*) (Fig. 4A); however, the functions of these laccases in cotton fiber elongation are not known. Since the laccases are involved in cell wall polymer bridge formation through oxidation of phenolic compounds, it is possible that these laccases might be playing an active role in the fiber elongation process. Transcriptome analysis of *G. arboreum*, showed that the stages between 10 and 15 DPA marks the transition/ onset of SCW stage^38^. The present study, supported by gene expression and biochemical analysis, is consistent with published report. The expression analysis at 10 and 15 DPA showed higher relative expression of 8 laccase groups (Fig. 4B). Among highly abundant genes, *GaLAC04, GaLAC11* and *GaLAC17* are homologues of *Arabidopsis* laccases (*AtLAC04, AtLAC11* and *AtLAC17*) which are involved in SCW biosynthesis^33^. The expression pattern of *GaLAC04* and *GaLAC17* is consistent with previous reports on *G. arboreum* fiber development^38^ indicating their potential role in the onset of SCW stage.

Large amounts of crystalline cellulose is deposited during SCW biosynthetic stage leading to rapid increase in fiber thickness^39^. The published evidence shows that expression of key genes involved in SCW development in *Arabidopsis* xylem, are also expressed in cotton fiber SCW biosynthetic stage^40^. The gene expression analysis of *G. arboreum* at SCW stage showed an increased relative expression of 6 laccase groups (Fig. 4C). A comparison among the SCW expressed genes showed that *GaLAC04_2, GaLAC04_3* and *GaLAC04_5* are highly expressed (Fig. 4C and Supplementary Fig. S3). Overall gene expression analysis showed similar orthologous SCW laccases are commonly expressed in *Arabidopsis* and cotton fiber despite the amount of lignin deposited in cotton fiber is negligible compared to *Arabidopsis* stems. Though exact reason for low level of lignin deposition in cotton fiber is not known, it is possible that the availability of monolignols is a limiting factor in cotton fibers. Domestication and selection for white cotton fiber (~96% cellulose) must have resulted in gradual reduction in monolignol biosynthesis which is evident from reduction of lignin from ~8% in naturally colored cotton to ~2% in white fibers. In addition, total metabolomic analysis could not detect canonical monolignols in *G. hirsutum* fibers which indicated either absence or below detection levels^41^. Functional studies on these individual laccases using novel genome editing tools such as CRISPR\Cas9 with minimal off target effect^42^ will provide important information on role of individual laccase genes in cotton fiber development.

### Laccase enzyme activity and relevant biochemical analysis

The cell elongation is a dynamic process that requires deposition and re-arrangement of cell wall components. Laccases regulate cell elongation by oxidizing phenolic acids such as ferulic and *p*-coumaric acids which arrest the cell elongation^17^,^18^,^19^. The present study performed laccase enzyme activity assay along with relevant biochemical analysis of developing cotton fibers to correlate laccase activity with biochemical analysis. The total soluble and wall bound phenolic compounds showed high abundance at 10 DPA followed by a steady decrease towards 25 DPA (Fig. 7B and Supplementary Fig. S6A,B). The high abundance of phenolic acids during elongation stage is consistent with their potential roles in fiber elongation. In addition, phenolic acids such as ferulic acid also serves as nucleation sites for lignification^43^, hence directly get incorporated into lignin polymer^44^. In pine xylem, a decrease in phenolic acid level is correlated with early xylem lignification process^45^. The present study showed a decrease in wall bound phenolic acid levels and a simultaneous increase in lignin content from 20 to 25 DPA (Figs 6 and 7), which could be due to the incorporation of phenolic acids into lignin or lignin like polymer in cotton fiber. Further, laccase activity assay showed a higher activity at 25 DPA, perfectly correlating with a rapid increase in lignin content (Figs 5 and 6). The overall combined laccase gene expression showed highest expression at 20 DPA; however, the total laccase activity was high at 25 DPA (Fig. 5 and Supplementary Fig. S4). From gene expression and activity assay results, it appears that there is a gap between transcription and laccase activity between 20 and 25 DPA. This could be due to either delayed translation^46^,^47^ and/or post-translation modifications (N-glycosylation, O-glycosylation and phosphorylation) of laccases^11^ expressed at 20 DPA. Further, some cotton laccases are targeted by miRNAs (Table 2) that are known to inhibit the translation^48^,^49^,^50^.

**Figure 8:**
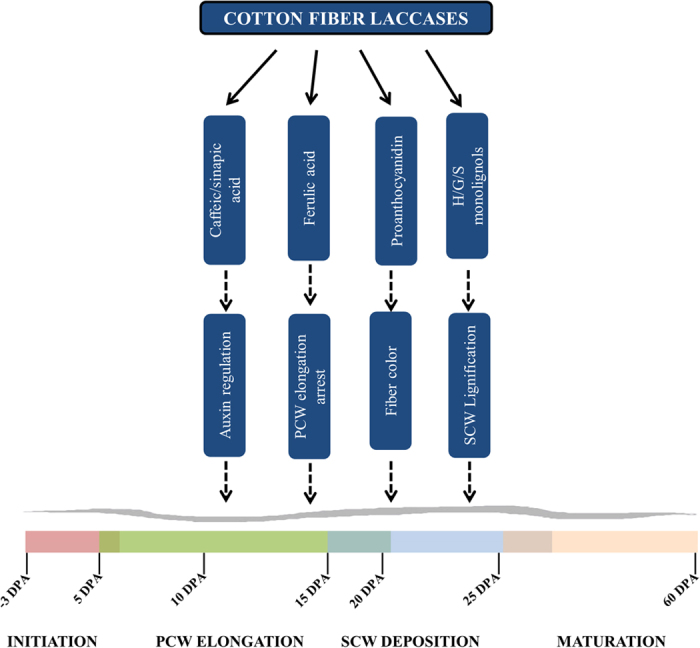
Potential roles of laccases in cotton fiber development. Phenolic compounds are found to have important roles in auxin regulation, cell elongation, lignin biosynthesis, cellular signaling and pigmentation in various plant species. The roles of laccases in cotton fiber development have not been characterized however the numbers and expression levels of laccases at different stages of fiber development indicate their potential roles in cotton fiber development and quality.

Overall, the present study provides comprehensive information on laccase gene family in cultivated tetraploid and its progenitor diploid species along with laccase gene expression, enzymatic and biochemical analysis of developing cotton fiber. The data from present study indicates potentially important roles of laccase genes (Fig. 8) and offers strategies for manipulation of cotton fiber development and properties using laccase genes.

## Materials and Methods

### Identification and nomenclature of cotton laccase family genes

The availability of reference genome sequence for two diploids (*G. arboreum* and *G. raimondii*) and one tetraploid species (*G. hirsutum*) offered an excellent source for the identification and analysis of the large multifunctional laccase gene family^25^,^26^,^27^. The *in silico* identification of cotton laccase gene family was performed by downloading the reference genomes (*G. arboreum*, *G. raimondii* and *G. hirsutum*) from CottonGen database (https://www.cottongen.org/data/download/genome)^51^. The cotton laccase genes were identified using *Arabidopsis* laccase family protein sequences retrieved from Tair10 (https://www.arabidopsis.org/)^52^. Genome-wide blastP similarity search (ftp://ftp.ncbi.nlm.nih.gov/blast/executables/blast+/LATEST/) was performed against cotton protein sequences from all three genomes using *Arabidopsis* laccase protein sequence as query. Default parameters were used to perform blastP with an e-value of 10^−10^, comparison matrix: BLOSUM62 and number of maximum alignment to show was 100^53^. After removing the redundant sequences, the identified sequences were analyzed for the presence of conserved laccase (TIGR03389), CuRO_1_LCC_plant (cd13849), CuRO_2_LCC_plant (cd13875) and CuRO_3_LCC_plant (cd13897) domains using NCBI’s Conserved Domains Database (CDD; http://www.ncbi.nlm.nih.gov/Structure/cdd/wrpsb.cgi)^54^. Further, InterProScan with default parameters was also used to validate the essential conserved domain profile^55^. Genome-wide reciprocal blastN similarity searches were performed by using validated cotton laccase gene sequences in all three cotton genomes. Naming of cotton laccase genes were performed according to the orthologous sequence similarity with *Arabidopsis* laccase proteins. Using individual *Gossypium* laccase sequences as query, *Arabidopsis* proteome was searched using blastP program to identify *Arabidopsis* homologue and named based on best match. Multiple cotton homologues were identified for some *Arabidopsis* laccase proteins. These multiple cotton homologues were sub grouped based on phylogenetic closeness between them (Fig. 2 and Supplementary Table S4).

### Analysis of gene structure, conserved domains and subcellular localization of laccases

The identified laccases were further analyzed for gene structure, organization, domain analysis and subcellular localization. For the exon/intron structure determination, the genomic sequences were retrieved from the database using the SAMtools faidex program (http://samtools.sourceforge.net/)^56^. The coding sequence of each gene was compared with its genomic sequence using Gene Structure Display Server (GSDS 2.0; http://gsds.cbi.pku.edu.cn/)^57^ to obtain the exon/intron structure. Conserved domains in *G. arboreum*, *G. raimondii* and *G. hirsutum* laccase proteins were searched using web based NCBI’s Conserved Domain Database (CDD: http://www.ncbi.nlm.nih.gov/Structure/cdd/wrpsb.cgi)^54^ and represented using the online Illustrator tool for Biological Sequences (IBS: http://ibs.biocuckoo.org/)^58^. The theoretical pI and molecular weight of the laccase proteins were calculated using ExPASy server’s Compute pI/Mw tool (http://web.expasy.org/compute_pi/)^59^. Subcellular localization pattern of laccase genes were predicted using web based tool TargetP 1.1 server (http://www.cbs.dtu.dk/services/TargetP/)^60^. The default cut-off values were used with plant as organismal group to perform subcellular localization analysis.

### Sequence retrieval and phylogenetic analysis

Phylogenetic analysis was performed using laccase family proteins of *Arabidopsis* and *Poplar* (*Populus trichocarpa*) downloaded from TAIR10 (https://www.arabidopsis.org/)^52^ and phytozome databases (http://phytozome.jgi.doe.gov/pz/portal.html)^61^, respectively. Multiple sequence alignment of 240 laccase proteins from 5 plant species (*A. thaliana, P. trichocarpa, G. arboreum, G. raimondii* and *G. hirsutum*) was performed using ClustalW sequence alignment program^62^ with default parameters (pairwise alignment gap open: 10 and gap extension: 0.1, multiple alignment gap open: 10 and gap extension: 0.2, protein weight matrix: BLOSUM). After exporting the alignment to MEGA file, phylogenetic tree was constructed with MEGA v6.2 (http://www.megasoftware.net/)^63^ using Neighbor-Joining (NJ) method with bootstrap test (replicated 1000 times) and other default parameters (phylogenetic reconstruction, substitution type: amino acids, model/methods: Jones-Taylor-Thornton (JTT) model, rates among sites: uniform rates and gap missing data treatment: partial deletion). Further, maximum likelihood method of MEGA v6.2 was also used to validate the results obtained from the NJ method.

### Tandem duplication and synteny analysis

Chromosomal distribution of laccase genes was shown for *G. arboreum, G. raimondii, G. hirsutum* (A^T^- and D^T^- subgenomes) genomes by using their chromosomal coordinates. In A, D and AD_1_ cotton genomes, tandem duplication within laccase gene family was analyzed using genome co-ordinates of respective genomes. Adjacent laccase genes with no more than one intervening gene in between them were considered tandemly duplicated. For segmental duplication, synteny blocks within A, D, A^T^ and D^T^ genomes were analyzed using the MCSCAN (http://chibba.agtec.uga.edu/duplication/mcscan/)^64^ with the default parameters. The MCSCAN input files were prepared using blastP with e-value of 10^−10^ and filtered for redundancy using python program. Synteny blocks with laccase genes were manually identified and plotted using custom made perl program.

### Computational prediction of laccase genes with miRNA target sites

Multiple genes of laccase family were targeted by miRNAs in *Arabidopsis*, rice and poplar^49^,^50^. Since, the expression analysis was performed in *G. arboreum;* we performed miRNA target analysis of laccase genes in this species. *G. arboreum* laccase genes were analyzed for the presence of miRNA target sites using psRNATarget server (http://plantgrn.noble.org/psRNATarget/)^65^ with stringent parameters (maximum expectation values of 2.0 were used in order to avoid the false positives, length for complementarity scoring: 20 bp, target accessibility-allowed maximum energy to unpair the target site (UPE): 25, flanking length around target site for target accessibility analysis: 17 bp upstream and 13 bp downstream, range of central mismatch leading to translational inhibition: 9–11 nucleotides).

### Cotton plant growth, sample collection and preparation

Delinted *G. arboreum* seeds were imbibed in water overnight and germinated in soil pots (12 × 12 cm size) and grown in environmentally controlled growth chambers at 28 °C with 16 h light and 8 h dark conditions. Two week old seedlings (~15 cm height) were transplanted into bigger circular soil pots (30 cm in diameter) containing Metro mix 852^®^, transferred to green house and grown to maturity at 30 °C. Flowers were tagged at 0 DPA and 10 independent cotton bolls from four biological replicates (independent plants) were collected at 5, 10, 15, 20 and 25 DPA. The bolls were split opened, flash frozen immediately in liquid Nitrogen and stored in ultralow freezer at −80 °C. The fibers from four biological replicates were pooled for each DPA and a representative sample was used for RNA extraction. For all biochemical analysis, from the pooled biological replicate, three independent technical replicates of fiber samples were used from stages 10, 15, 20 and 25 DPA. After complete sample collection, the fibers were separated from the frozen ovules by gently grinding using mortar and pestle to separate the fibers from ovules. The 5 DPA fiber sample was very low due the small size of the fiber initials despite the collection of large number of ovules. Hence the samples from four biological replicates were pooled for all stages for a fair comparison of different stages. The pooled samples represent four independent plants, multiple bolls from each plant, multiple seeds from each boll and multiple fiber cells from single seed. The samples are pooled to reduce the variation arising from individual plants, position of bolls, ovules within bolls and fiber position on the seeds. The fibers were ground into fine powder using liquid Nitrogen in SPEX freeze mill 6870 (SPEX sample prep^®^, USA). The finely ground fiber powder was stored at −80 °C and used as a single source for gene expression, enzyme activity and biochemical analysis.

### Primer design for quantitative PCR and specificity analysis

Primers for 44 *G. arboreum* laccase genes were designed using primer 3 software (http://bioinfo.ut.ee/primer3-0.4.0/)^66^ with melting temperatures above 60 °C and with no 3′ or 5′ end complementarity (to prevent primer dimers). Since laccase genes showed high sequence similarity, to distinguish individual genes, the forward primer was designed from 3′ coding region and reverse primer was designed from 3′ untranslated region (UTR) (Supplementary Table S7). All the primer pairs were validated for specificity using regular PCR reactions for their specificity and amplification efficiency under different annealing temperatures (data not shown) and optimized 56 °C was used as common annealing temperature for all primers for quantitative PCR (qPCR) experiments. Further, the amplified PCR products from 10 randomly selected *G. arboreum* laccase genes representing different stages of fiber development were sequenced using Sanger sequencing technology (Genewiz, USA). The sequencing results confirmed the specificity of the primers (Supplementary Fig. S7).

### RNA isolation, cDNA synthesis and gene expression analysis using quantitative PCR

To perform the gene expression analysis using qPCR, total RNA was extracted from ground fiber samples using Sigma total RNA extraction kit (Sigma, USA) and on-column DNase (Sigma, USA) treatment was performed according to the manufacturer instructions. The RNA concentration was measured using UV spectrophotometer (BioSpectrophotometer^®^ Kinetic, Eppendorf) at 260 nm and the integrity of RNA was analyzed on 1% agarose gels. One microgram of total RNA was used for first strand cDNA synthesis using Invitrogen superscript 2^®^ reverse transcriptase kit (Invitrogen, USA) using oligodT primers. Briefly, total RNA was incubated with reverse transcriptase in presence of dNTP, oligodT and first strand buffer at 42 °C for 50 min followed by heat deactivation of enzyme at 70 °C for 50 min. The obtained cDNA was diluted 5 times and used for quantitative PCR reactions using Roche fast start Sybergreen^®^ (Roche, USA) in LightCycler^®^ 96 (Roche, USA) machine. All qPCR runs were performed at 56 °C annealing temperature with a fixed protocol and melt curve analysis was performed. The obtained Ct values were exported to excel file to calculate ∆Ct and ∆∆Ct values. ∆Ct was calculated by subtracting the Ct values of Histone 3 (internal control) with the target laccase gene within the same stage (for example 5 DPA) whereas ∆∆Ct was calculated by subtracting the ∆Ct values of fiber stages 10, 15, 20 and 25 DPA with that of 5 DPA. Hence the results were presented as relative expression of laccase genes in different stages normalized with the 5 DPA.

### Analysis of soluble and wall bound phenolic compounds of developing cotton fiber

The freezer mill ground fiber powder from different stages was used for quantification of soluble and wall bound phenolic compounds. The soluble and wall bound phenolic acid extractions were performed following published protocols^67^. Briefly, 800 μl of 80% methanol was added to 100 mg of fiber powder, vortexed and centrifuged at 13300 rpm for 7 minutes at room temperature. The supernatant was transferred to a new tube and the extraction was repeated again with 800 μl of 80% methanol. Supernatants from the two methanol extraction steps were pooled and were used to analyze soluble phenolics. The pellet was processed for the analysis of wall bound phenolics. One ml of acetone was added to the pellet and vortexed and centrifuged at 13300 rpm for 7 minutes at room temperature. The pellet was air dried overnight at room temperature and the air dried pellet was dissolved in 1 ml of 0.5 M sodium hydroxide and incubated at 96 °C for 2 h to carry out mild saponification of phenolic acids from fiber cell walls. After incubation, the tubes were centrifuged at 13300 rpm for 10 minutes, supernatant was transferred to a fresh tube, neutralized with 250 μl of 2 M HCL and the pellet was dried stored for lignin extraction. The tubes with neutralized supernatant were centrifuged at 20800 rpm for 10 minutes and the resultant supernatant was transferred to a fresh tube. One ml of diethyl ether is added to the supernatant, vortexed and upper ether phase is transferred into a new tube. This step is repeated twice and the obtained 2 ml supernatant was dried in vaccufuge (Eppendorf). The dried pellet is dissolved in 1 ml of 10% methanol (pH: 2.1) and then analyzed using HPLC to detect wall bound phenolic acids. Agilent HPLC 1200 series with a standard Zorbax SB-C18 column (3.5 μl, 4.6*150 mm) was used. The mobile phase was 33% A (Acetonitrile) and 67% B (milli-Q water-pH 2.1) with a flow rate of 1.5 ml/minute. Appropriate standards, at a concentration of 1 mg/ml were used for caffeic acid, *p-*coumaric acid, ferulic acid, sinapic acid and vanillic acid. 1-Napthalene acetic acid (1 mg/ml) was used as internal control to calculate the concentration of phenolic acids, which was represented as the amount per gram of extracted cell wall weight in case of wall bound compounds and per gram of fresh cell wall weight in case of soluble compounds. The results obtained were analyzed for statistical significance using Student’s t-test program with p-value < 0.05.

### Lignin content and composition of developing cotton fiber

The pellets obtained after soluble/wall bound phenolic extraction was pooled and used for TGA derivatization and lignin quantification. The lignin content was estimated using Thioglycolic acid (TGA) method^32^. TGA reagent (10%) was prepared using 3 M HCl. One ml of TGA reagent was added to the pellet and incubated at 80 °C for 3 h to add thioglycolic groups to the lignin structure to increase the solubility of lignin. All centrifugation steps from here onwards were carried out at 20000 g for 10 minutes at room temperature. After incubation, the tubes were centrifuged and the supernatant was discarded. The resultant pellet was then washed with 1 ml milli-Q water, centrifuged and supernatant was discarded. The pellet was vacuum dried and incubated with 1 ml of 1 M sodium hydroxide on thermal shaker at 37 °C for 24 h. The tube was centrifuged; the supernatant was acidified with 0.2 ml of concentrated HCL and incubated at 4 °C for 4 h to extract the trace amount of the derivatized lignin. After incubation, the tube was centrifuged, vacuum dried, solubilized in Dimethyl Sulfoxide (DMSO) to estimate lignin content using UV absorbance at 280 nm. A standard curve was plotted from different quantities of derivatized industrial lignin based on the absorbance at 280 nm. The quantity of lignin at different stages of *G. arboreum* cotton fiber was calculated using the established standard curve. The results obtained were analyzed for statistical significance using Student’s t-test program with p-value < 0.05.

### Laccase activity assay of developing cotton fibers using 1-D zymography

The laccase activity was assayed using 1-D zymography method^29^. Briefly, 200 mg of ground fiber samples from 10, 15, 20 and 25 DPA and stem sample (control for laccase activity) from *G. arboreum* were homogenized in 400 μl of protein extraction buffer (100 mM sodium phosphate buffer, pH 8.5 and protease inhibitors) and incubated on ice for 1 h with intermittent mixing to allow complete and efficient lysis. The homogenate was centrifuged at 10000 g for 20 minutes at 4 °C and the supernatant was taken and stored in aliquots at −20 °C. Protein concentration was estimated using Bicinchoninic Acid (BCA) protein assay kit (Fisher Scientific, USA). 50 micrograms of total proteins from different stages were run on non-denaturing PAGE (Poly Acrylamide Gel Electrophoresis) (12% separating and 6% stacking) at 20 mA for 2 h and the gel was rinsed with milli-Q water. The gel was incubated in 2.5% TritonX-100 for 1 h at 4 °C, washed thoroughly with distilled water and incubated for 2 h at 25 °C in 50 mM sodium tartrate buffer (pH 4). Laccase activity (appearance of green bands) was visualized by incubating in 5 mM ABTS solution for 20 minutes. Simultaneously, the loading control gel was run, stained with Coomassie brilliant blue solution for 1 h, destained (50:40:10 methanol: water: acetic acid) for 6 h and scanned.

## Additional Information

**How to cite this article**: Balasubramanian, V. K. *et al*. Genome-wide identification of multifunctional laccase gene family in cotton (*Gossypium spp.*); expression and biochemical analysis during fiber development. *Sci. Rep.* **6**, 34309; doi: 10.1038/srep34309 (2016).

## Supplementary Material

Supplementary Information

## Figures and Tables

**Table 1:** *In silico* identification and analyses of *G. arboreum* laccase gene family.

Gene Nos	*G. arboreum* (Gene Id)	Chromosomal location	Gene size		Protein		Sub-cellular localization
			Genomic (bp)	CDS (bp)	Molecular Weight (kDa)	pI	
*LAC02_1*	Cotton_A_20043	Chr8:81279651:81282485	2834	1503	55.103	9.26	Mitochondrial
*LAC02_2*	Cotton_A_30643	Chr7:112198734:112200887	2153	1734	63.723	9.36	Secretory
*LAC02_3*	Cotton_A_30645	Chr7:112238136:112240289	2153	1734	63.776	9.21	Secretory
*LAC02_4*	Cotton_A_30646	Chr7:112301808:112303961	2153	1734	63.635	9.35	Secretory
*LAC03_1*	Cotton_A_04178	Chr7:78051563:78053707	2144	1728	64.51	8.71	Secretory
*LAC03_2*	Cotton_A_24290	Chr13:89192357:89194554	2197	1728	63.92	8.52	Secretory
*LAC04_1*	Cotton_A_00335	Chr2:69974070:69977957	3887	1674	61.245	7.65	Secretory
*LAC04_2*	Cotton_A_05572	Chr4:129106164:129108201	2037	1671	60.628	9.28	Secretory
*LAC04_3*	Cotton_A_06597	Chr7:27437711:27439798	2087	1671	60.761	9.26	Secretory
*LAC04_4*	Cotton_A_12917	Chr9:66032424:66035279	2855	1671	61.108	9.42	Secretory
*LAC04_5*	Cotton_A_13553	Chr10:96546298:96548750	2452	1740	64.234	8.45	Secretory
*LAC04_6*	Cotton_A_20282	Chr7:54244095:54246807	2712	1668	61.301	9.1	Secretory
*LAC04_7*	Cotton_A_29171	Chr10:47067405:47070913	3508	1527	57.243	5.19	Non-secretory
*LAC04_8*	Cotton_A_32213	Chr10:87092454:87095892	3438	1668	61.279	8.32	Secretory
*LAC05_1*	Cotton_A_13817	Chr8:71027959:71034475	6516	1635	60.683	9.41	Secretory
*LAC05_2*	Cotton_A_13818	Chr8:71068414:71070598	2184	1704	63.281	9.25	Secretory
*LAC05_3*	Cotton_A_13820	Chr8:71134606:71136600	1994	1614	59.726	9.23	Non-secretory
*LAC05_4*	Cotton_A_15837	Chr13:66318063:66320195	2132	1617	59.411	6.56	Non-secretory
*LAC06_1*	Cotton_A_14417	Chr4:7772175:7774365	2190	1707	63.011	6.49	Secretory
*LAC06_2*	Cotton_A_25874	Chr6:45584114:45586109	1995	1671	61.874	7.97	Non-secretory
*LAC07_1*	Cotton_A_22687	Chr12:86399040:86401261	2221	1650	60.477	7.42	Mitochondrial
*LAC07_2*	Cotton_A_30033	Chr12:111604680:111608069	3389	1704	62.69	8.57	Secretory
*LAC07_3*	Cotton_A_30034	Chr12:111490687:111493668	2981	1713	62.683	8.8	Secretory
*LAC07_4*	Cotton_A_30035	Chr12:111394263:111396884	2621	1626	59.578	8.14	Non-secretory
*LAC07_5*	Cotton_A_35771	Chr13:539188:543296	4108	1695	62.267	7.06	Secretory
*LAC11_1*	Cotton_A_00882	Chr13:73503949:73506086	2137	1692	62.768	9.11	Secretory
*LAC11_2*	Cotton_A_17036	Chr9:56144460:56146555	2095	1764	65.228	8.69	Secretory
*LAC11_3*	Cotton_A_19723	Chr13:63352239:63354366	2127	1692	62.213	8.76	Secretory
*LAC11_4*	Cotton_A_26217	Chr7:32225217:32227325	2108	1608	59.677	9.16	Secretory
*LAC11_5*	Cotton_A_26221	Chr7:32087805:32092421	4616	1350	49.982	9.46	Mitochondrial
*LAC12*	Cotton_A_31477	Chr10:76670719:76672888	2169	1602	59.049	9.43	Non-secretory
*LAC14_1*	Cotton_A_04514	Chr6:105156736:105159112	2376	1701	63.286	5.48	Secretory
*LAC14_2*	Cotton_A_04517	Chr6:105080230:105082613	2383	1692	63.128	6.83	Secretory
*LAC14_3*	Cotton_A_04519	Chr6:105038752:105049813	11061	1749	64.751	5.01	Non-secretory
*LAC14_4*	Cotton_A_04522	Chr6:105012062:105014213	2151	1563	58.041	4.76	Secretory
*LAC14_5*	Cotton_A_04526	Chr6:104947203:104949548	2345	1656	61.351	4.81	Secretory
*LAC14_6*	Cotton_A_10403	Chr9:69308986:69311473	2487	1743	66.001	6.05	Secretory
*LAC14_7*	Cotton_A_37880	Chr12:73129546:73131714	2168	1716	63.839	6.25	Secretory
*LAC15*	Cotton_A_04513	Chr6:105171153:105174123	2970	1584	59.121	7.68	Non-secretory
*LAC17_1*	Cotton_A_00902	Chr13:73653118:73655337	2219	1728	63.511	8.48	Secretory
*LAC17_2*	Cotton_A_00905	Chr13:73732088:73734303	2215	1728	63.494	8.73	Secretory
*LAC17_3*	Cotton_A_00947	Chr13:74192475:74194689	2214	1728	63.511	8.75	Secretory
*LAC17_4*	Cotton_A_07013	Chr10:88398331:88400538	2207	1737	63.98	8.93	Secretory
*LAC17_5*	Cotton_A_12054	Chr11:77398558:77400798	2240	1842	68.113	9.85	Secretory

**Table 2:** List of *G. arboreum* laccase genes with putative miRNA target sites.

Gene	*G. arboreum*	Predicted miRNA target sites	miRNA length	Expectation	UPE
*GaLAC02_1*	Cotton_A_20043	miR397, miR397a, miR397b	20–21	0.5–2	16.09–16.44
*GaLAC02_2*	Cotton_A_30643	miR397, miR397a, miR397b	20	0.5–2	18.078–21.35
*GaLAC02_3*	Cotton_A_30645	miR397, miR397a, miR397b	20	1.5–2	17.66
*GaLAC02_4*	Cotton_A_30646	miR397, miR397a, miR397b	20	1.5–2	19.84
*GaLAC03_1*	Cotton_A_04178	miR408a	21	1	16.08
*GaLAC03_2*	Cotton_A_24290	miR408a	21	1.5	20.77
*GaLAC04_2*	Cotton_A_05572	miR397, miR397a, miR397b	20–21	1–2	15.76–16.13
*GaLAC04_3*	Cotton_A_06597	miR397, miR397a, miR397b	20–21	0-1.5	13.10–14.323
*GaLAC04_4*	Cotton_A_12917	miR397, miR397a, miR397b	20–21	1.5–2	12.03–12.99
*GaLAC04_6*	Cotton_A_20282	miR397, miR397a, miR397b	20–21	1.5–2	12.13–13.29
*GaLAC05_4*	Cotton_A_15837	miR408a	20	1	19.55
*GaLAC07_1*	Cotton_A_22687	miR397, miR397b	20	2	10.92
*GaLAC07_2*	Cotton_A_30033	miR397	20	1.5	12.5
*GaLAC07_5*	Cotton_A_35771	miR397, miR397a, miR397b	20–21	0.5–2	11.11–12.01
*GaLAC11_1*	Cotton_A_00882	miR397, miR397a, miR397b	20–21	0–1.5	14.67–16.82
*GaLAC11_3*	Cotton_A_19723	miR397, miR397a, miR397b	20–21	1.5–2	12.18–13.39
*GaLAC11_4*	Cotton_A_26217	miR397, miR397a, miR397b	20–21	2	13.56
*GaLAC11_5*	Cotton_A_26221	miR397, miR397a, miR397b	20–21	1–2	13.41–14.27
*GaLAC12*	Cotton_A_31477	miR408a	20	1	14.93
*GaLAC17_1*	Cotton_A_00902	miR397, miR397a, miR397b	20	1.5–2	13.39
*GaLAC17_2*	Cotton_A_00905	miR397	20	2	12.75
*GaLAC17_5*	Cotton_A_12054	miR397, miR397a, miR397b	20–21	2	17.70–17.54
