# Supplementary material for: Genome-wide identification of multifunctional laccase gene family in cotton (Gossypium spp.); expression and biochemical analysis during fiber development
Source: Sci Rep. 2016 Sep 29;6:34309. doi: 10.1038/srep34309 (PMC5041144; doi:10.1038/srep34309)
Supplement: Supplementary Information [file srep34309-s1.pdf]

Supplementary Information

Genome-wide identification of multifunctional laccase gene family in cotton (*Gossypium spp.*); expression and biochemical analysis during fiber development

Vimal Kumar Balasubramanian[1], Krishan Mohan Rai[1], Sandi Win Thu[1], Mei Mei Hii[1,2] and Venugopal Mendu[1]*

[1] Fiber and Biopolymer Research Institute (FBRI), Department of Plant & Soil Science, Texas Tech University, 2802, 15th street, Lubbock, TX, 79409, USA.

[2] Current address Sarawak Biodiversity Centre, KM20, Jalan Borneo Heights, Semengoh, Locked Bag No. 3032, Kuching, Sarawak, 93990, Malaysia.

Supplementary Figure S1A

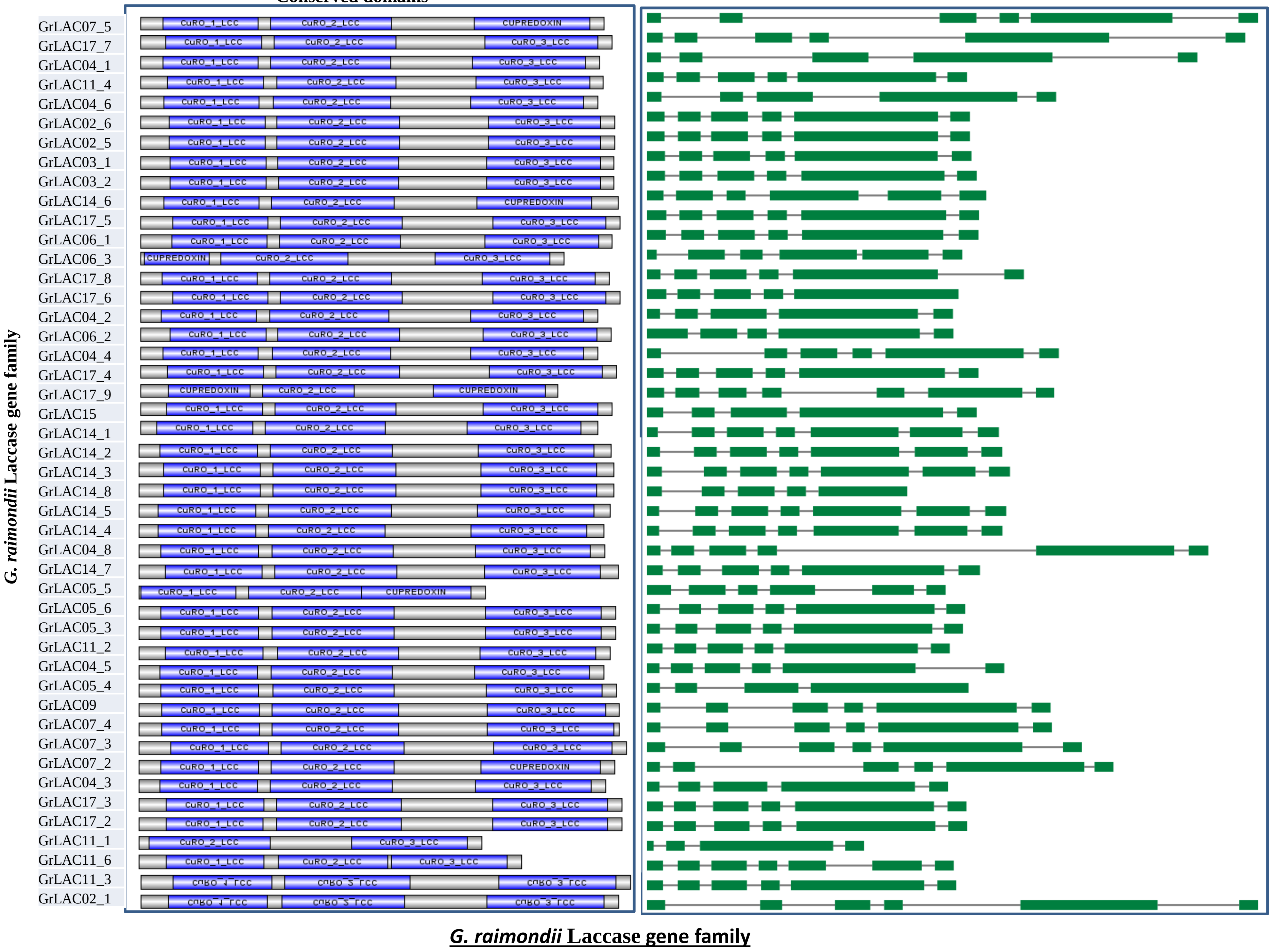

Supplementary Figure S1B

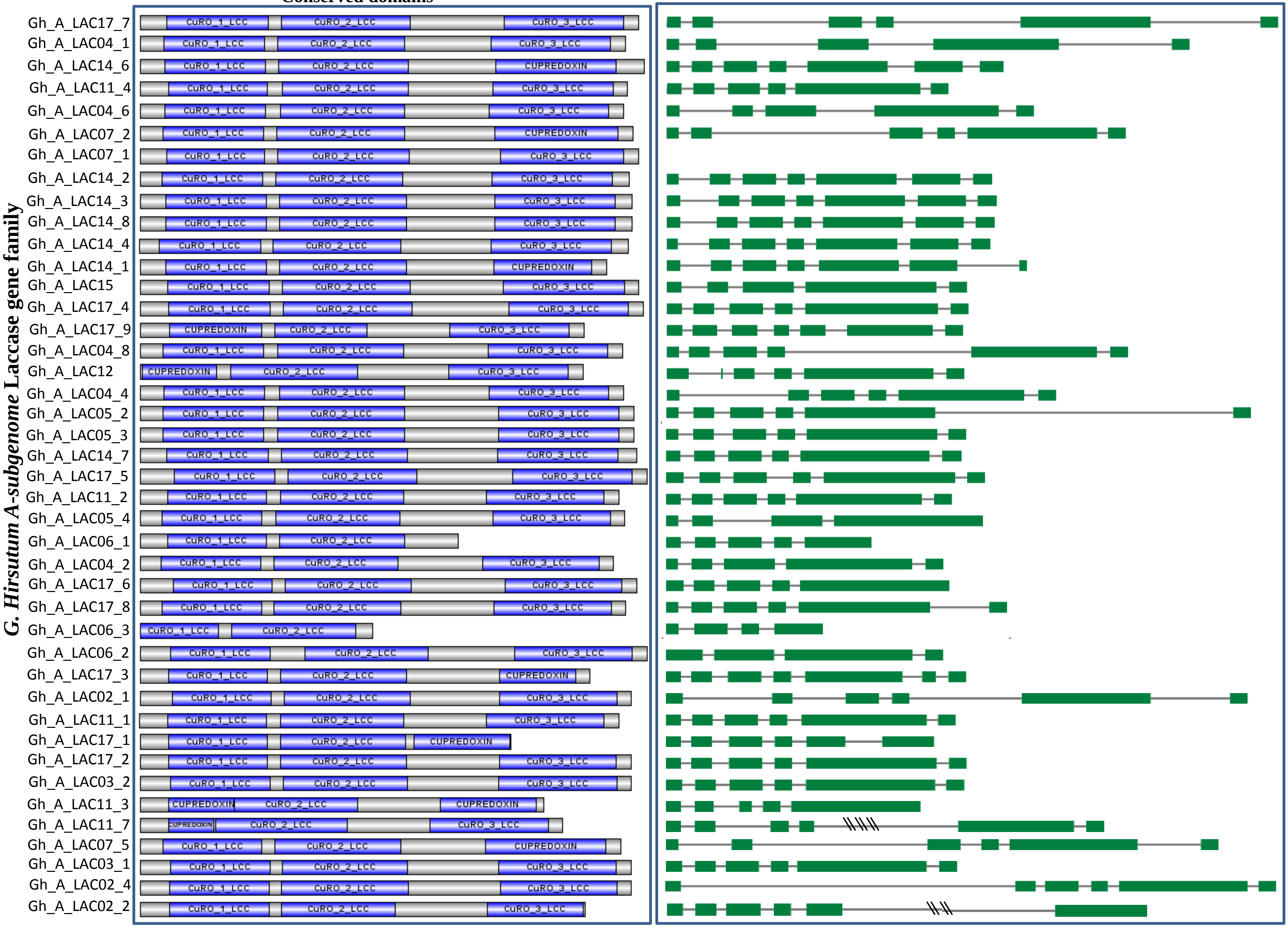

G. hirsutum A-subgenome Laccase gene family

Supplementary Figure S1C

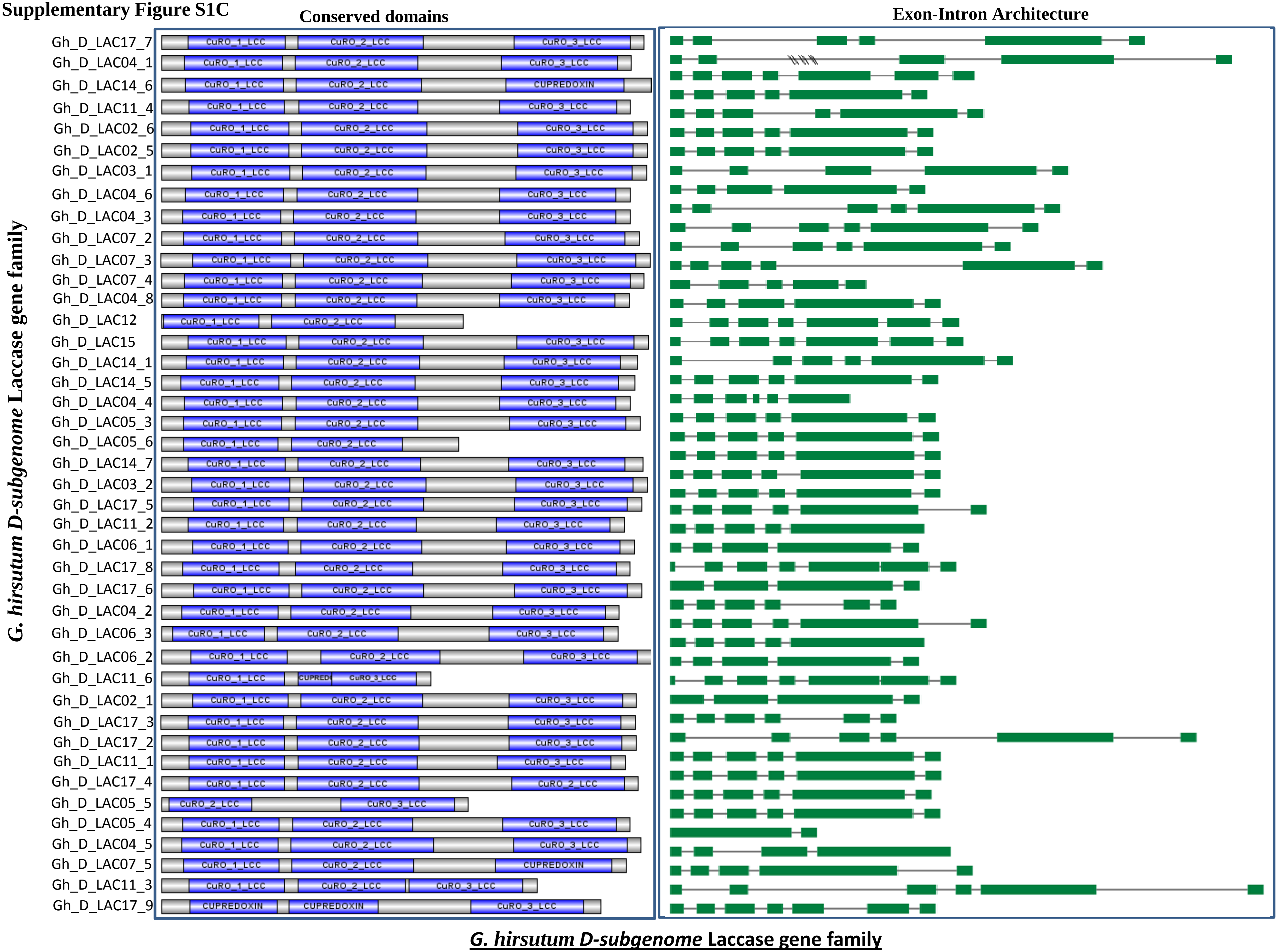

Supplementary Figure S1. Detailed conserved domain and Exon-intron architecture analysis from (A) *G. raimondii*, (B) *G. hirsutum* A-subgenome and (C) *G. hirsutum* D-subgenome. Green boxes represent exons whereas introns were represented with black lines. Big introns are compressed in order to fit within space either with one double slash representing reduction of 1 Kb. Gene structure analysis for Gh_A04G0743 has been eliminated from this analysis due to possible mis-assembly of this gene.

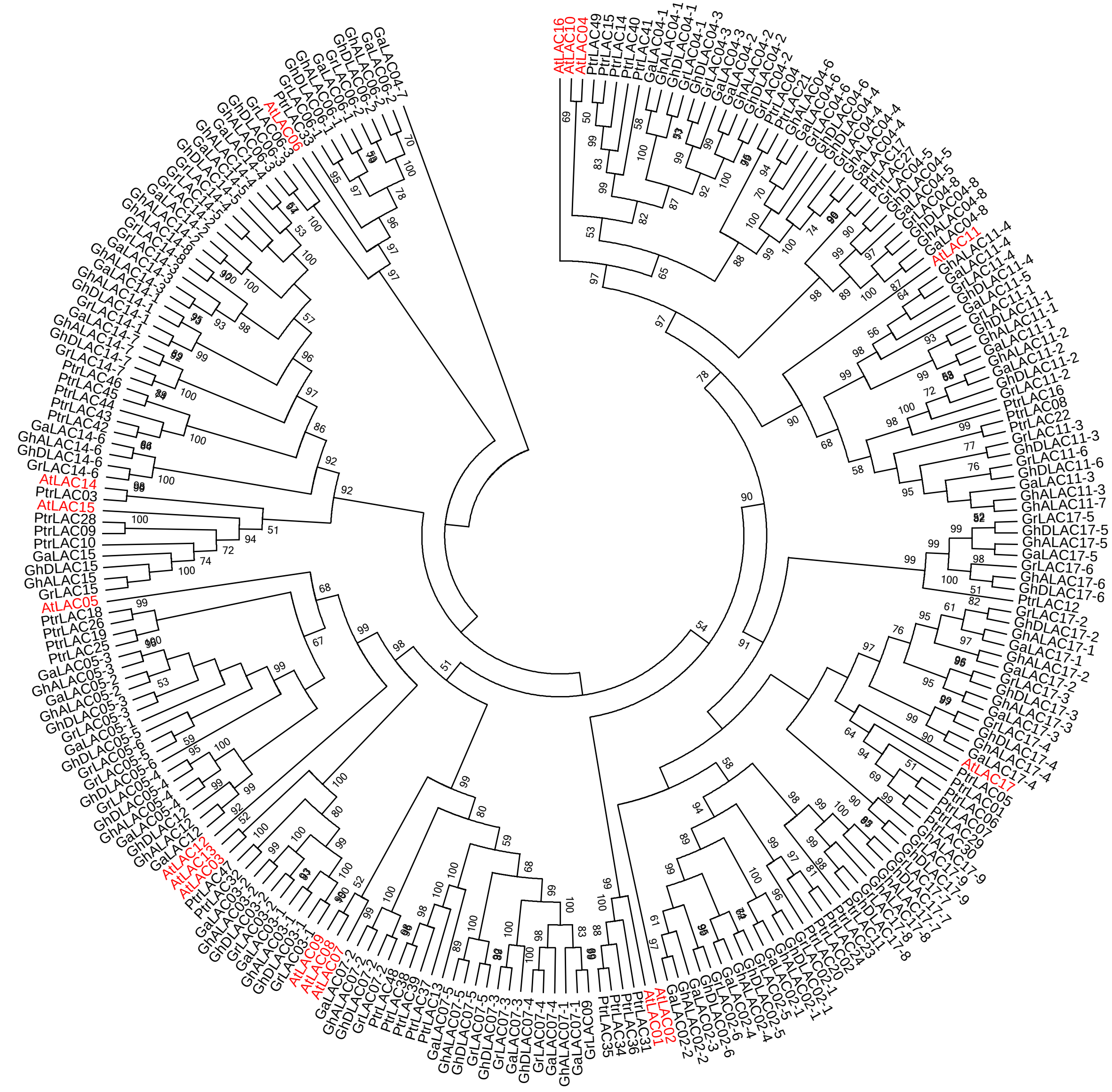

Supplementary Figure S2: Phylogenetic tree of cotton laccase proteins along with Arabidopsis and poplar laccases using maximum likelihood method. The constructed phylogenetic tree is supporting the results obtained in phylogenetic tree constructed using neighbor joining method.

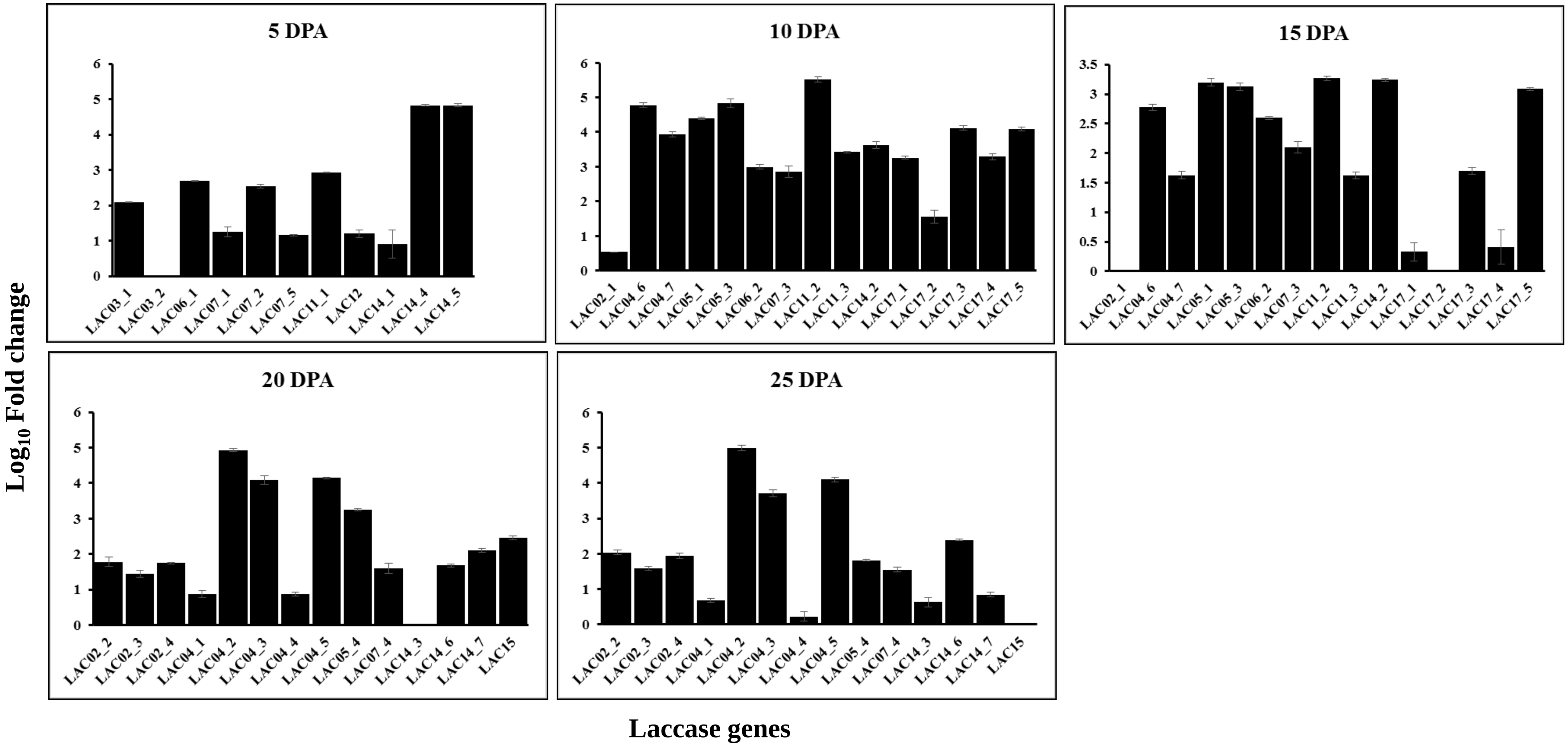

Supplementary Figure S3. Logarithmic fold change in expression level of laccase genes that were highly expressed in different stages of fiber development in *G.arboreum*. The expression of all laccases were normalized to the lowest expressing laccase in each stage separately.

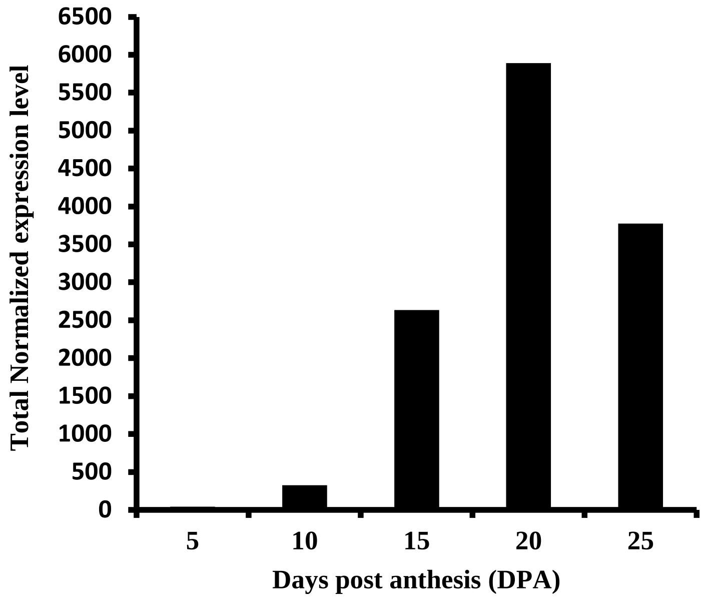

Supplementary Figure S4. The sum of normalized expression of all 40 laccase genes from each fiber developmental stage. Expression level of all 40 laccase genes from each developmental stage were added showing a maximum expression at 20DPA. *GaHisone-3b* was used as internal control to normalize the expression data of each laccase. 5DPA was set to be 1 and expression level at other stages was calculated accordingly.

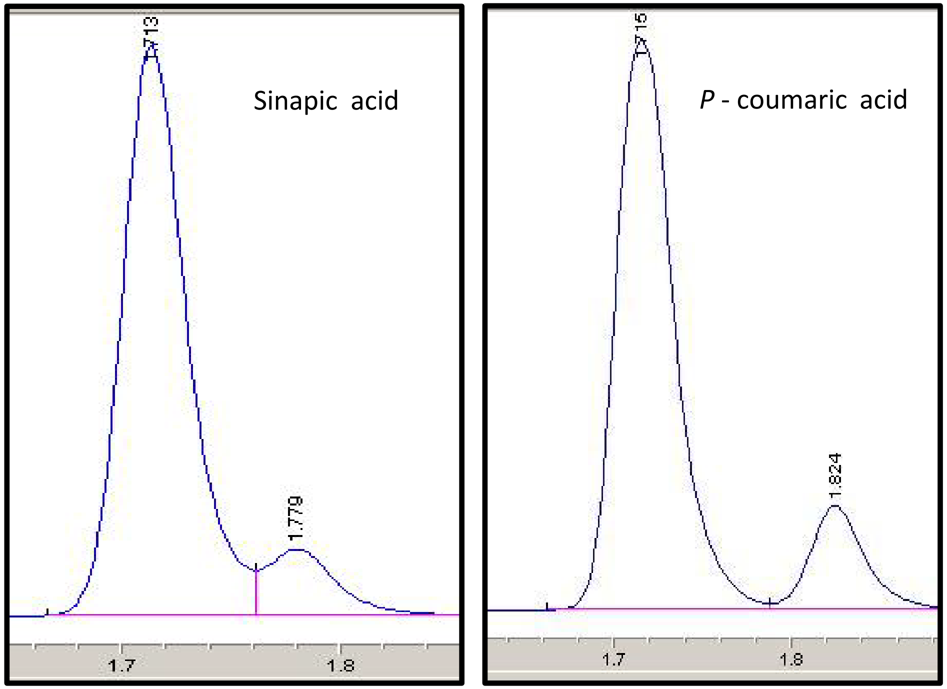

Supplementary Figure S5. HPLC elution profile of sinapic acid (elution time-1.713 minutes) and *p*-coumaric acid (elution time-1.715 minutes) controls analyzed on C18 column in HPLC using mobile phases of 33% A (100% acetonitrile) and 67% B (ultra-pure water pH: 2.1) at a flow rate of 1.5ml/minute. Both the control compounds eluted very closely under different composition of mobile phases and flow rates (data not shown). The shorter peaks adjacent to the taller peak could be a dimer of respective phenolic acid control compounds.

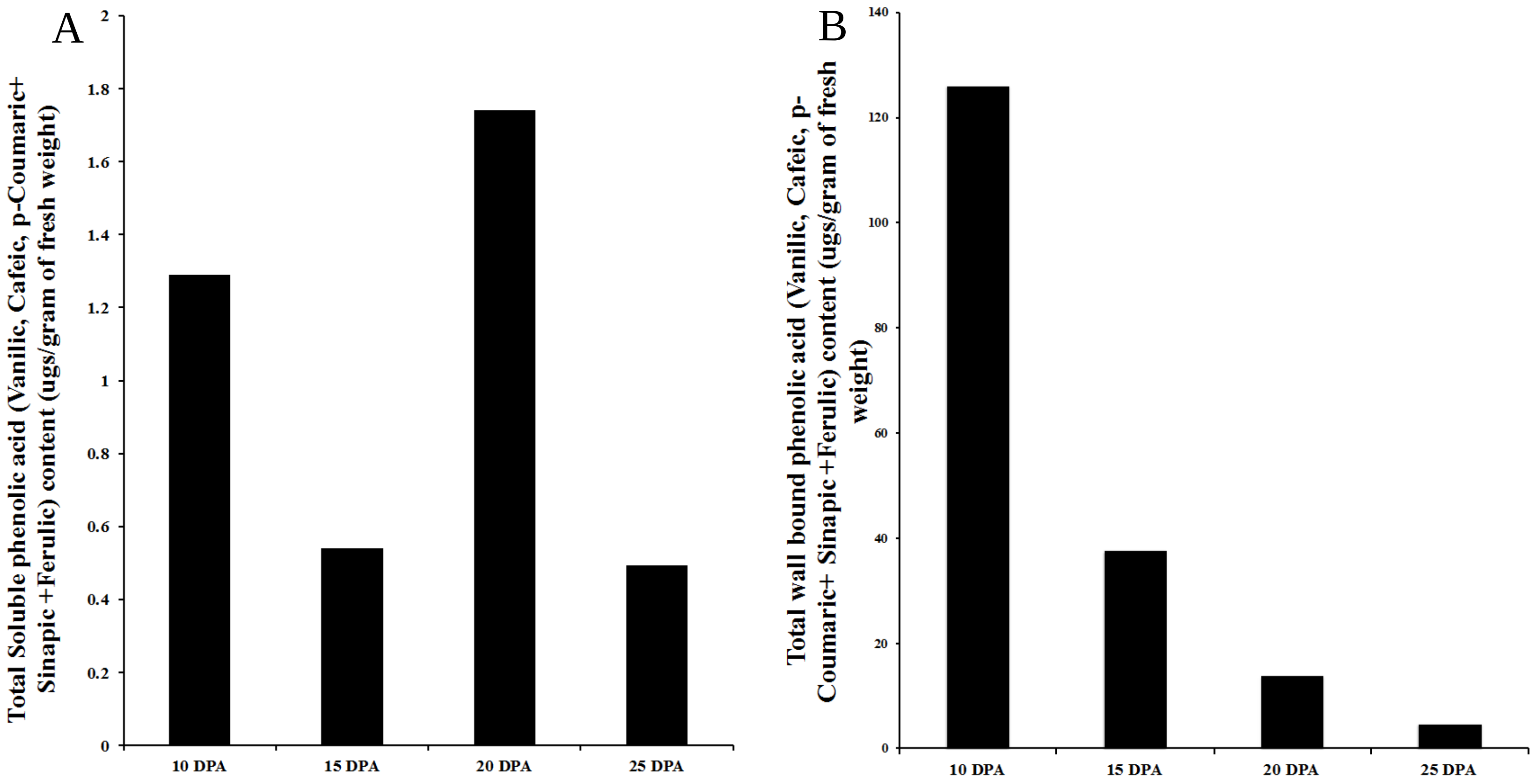

Supplementary figure S6. Total Soluble and Wall bound phenolic acid content during *G. arboreum* fiber development.

A. Total soluble phenolic acid content was represented as µg/gram of fresh weight.
B. Total wall bound phenolic acid content was represented as µg/gram of fresh weight.

Soluble and wall bound phenolic acids fraction were extracted from different developmental stages (10, 15, 20 and 25 DPA) and analyzed using HPLC with C18 column. Mobile phases composed of 33% A (100% acetonitrile) and 67% B (ultra-pure water pH: 2.1) at a flow rate of 1.5ml/minute. Standards for ferulic acid, coumaric acid, caffeic acid, sinapic acid and vanillic acid were used at 1mg/ml concentration. 1-Napthalene acetic acid (1mg/ml) was used as an internal control to calculate the phenolic acid content of cotton fibers.

Supplementary Figure S7. Specific amplification of laccase genes by the qPCR primers. The amplified PCR products from 10 representative laccase genes were sequenced and used as query and BLASTN was performed against 44 *G.arboreum* laccase (CDS+3'UTR) sequences. The BLASTN results showed highest similarity match with the respective laccase gene.

1) **GaLAC04_2**

Cotton_A_05572 locus=CA_chr4:129106164:129108201:-

Sequence ID: lcl|Query_14684 **Length:** 707 **Number of Matches:** 1

Range 1: 345 to 515 Graphics ▼ Next Match ▲ Previous Match

Score	Expect	Identities	Gaps	Strand
303 bits(164)	2e-86	169/171(99%)	2/171(1%)	Plus/Minus

```
Query  6    GTTTC-TAGTGTCTTCTTCGAAACTTTCGATGTGGAAAAGGGGATGCTGCTGCCATTATT  64
            ||||| |||||||||||||||||||||||||||||||||||||||||||||||||||||
Sbjct  515  GTTTCATAGTGTCTTCTTCGAAACTTTCGATGTGGAAAAGGGGATGCTGCTGCCATTATT  456

Query  65   -AACATTTTGGAAGATCACTCGGCGGAGGAACAAGTGACTGATTTGGACCTTTTCCATTC  123
             |||||||||||||||||||||||||||||||||||||||||||||||||||||||||||
Sbjct  455  TAACATTTTGGAAGATCACTCGGCGGAGGAACAAGTGACTGATTTGGACCTTTTCCATTC  396

Query  124  TCCACCAAGAATGCCATCTTAAGCCCCCATGTCGTATGCACTTCCAAATGG  174
            |||||||||||||||||||||||||||||||||||||||||||||||||||
Sbjct  395  TCCACCAAGAATGCCATCTTAAGCCCCCATGTCGTATGCACTTCCAAATGG  345
```

2) **GaLAC04_3**

Cotton_A_06597 locus=CA_chr7:27437711:27439798:-

Sequence ID: lcl|Query_220477 **Length:** 707 **Number of Matches:** 2

Range 1: 378 to 586 Graphics ▼ Next Match ▲ Previous Match

Score	Expect	Identities	Gaps	Strand
379 bits(205)	2e-109	208/209(99%)	1/209(0%)	Plus/Minus

```
Query  11   TCTTCTTTTGCTTATTAAATAACACAGGTTCACAGCCTCATCTTCTGCACATGAAAGAAA  70
            ||||||||||||||||||||||||||||||||||||||||||||||||||||||||||||
Sbjct  586  TCTTCTTTTGCTTATTAAATAACACAGGTTCACAGCCTCATCTTCTGCACATGAAAGAAA  527

Query  71   TTCTTTCATTCTCTTTCCACGTTTCCTTTCATCGTAAAATCTCGGTGCTGGTGTCCATAG  130
            ||||||||||||||||||||||||||||||||||||||||||||||||||||||||||||
Sbjct  526  TTCTTTCATTCTCTTTCCACGTTTCCTTTCATCGTAAAATCTCGGTGCTGGTGTCCATAG  467

Query  131  TTGCCTGGTT-CAACACTTTGGAAGATCACTTGGAGGAGGAAGAAGTGACTGATTAGGCC  189
            |||||||||| |||||||||||||||||||||||||||||||||||||||||||||||||
Sbjct  466  TTGCCTGGTTTCAACACTTTGGAAGATCACTTGGAGGAGGAAGAAGTGACTGATTAGGCC  407

Query  190  CTTTTCCATTGTCTACCAAAAATGCCATC  218
            |||||||||||||||||||||||||||||
Sbjct  406  CTTTTCCATTGTCTACCAAAAATGCCATC  378
```

3) **GaLAC04_5**

Cotton_A_13553 locus=CA_chr10:96546298:96548750:-

Sequence ID: lcl|Query_212256 **Length:** 776 **Number of Matches:** 2

Range 1: 368 to 599 Graphics ▼ Next Match ▲ Previous Match

Score	Expect	Identities	Gaps	Strand
412 bits(223)	2e-119	229/232(99%)	1/232(0%)	Plus/Plus

```
Query  201  CTGCAATAAGATTCNGAGCAGATAACCCAGGGATCTGGTTTTTTCATTGCCATTTGGAAG  260
            |||||||||||||| |||||||||||||||||||||||||||||||||||||||||||||
Sbjct  368  CTGCAATAAGATTCAGAGCAGATAACCCAGGGATCTGGTTTTTTCATTGCCATTTGGAAG  427

Query  261  TTCATACAACATGGGGACTTAAGATGGCATTCTTGGTGGAAAACGGAAAAGGCCCGAACG  320
            ||||||||||||||||||||||||||||||||||||||||||||||||||||||||||||
Sbjct  428  TTCATACAACATGGGGACTTAAGATGGCATTCTTGGTGGAAAACGGAAAAGGCCCGAACG  487

Query  321  AGTCGATCGAGCCGCCTCCGAGCGATCTTCCGAAGTG-TTGAACTGGGTTTTAAGACAAC  379
            ||||||||||||||||||||||||||||||||||||| ||||||||||||||||||||||
Sbjct  488  AGTCGATCGAGCCGCCTCCGAGCGATCTTCCGAAGTGTTTGAACTGGGTTTTAAGACAAC  547

Query  380  TTTGAGAATCAAAGTTTGAGGGCAGAGAGGAGAATCAGATGCTGAGTTAGAA  431
            |||||||||||||||||||||| |||||||||||||||||||||||||||||
Sbjct  548  TTTGAGAATCAAAGTTTGAGGGTAGAGAGGAGAATCAGATGCTGAGTTAGAA  599
```

4) GaLAC04_6

lcl|Cotton_A_20282 locus=CA_chr7:54244095:54246807:+
Sequence ID: lcl|Query_176021 **Length:** 718 **Number of Matches:** 1

Range 1: 283 to 452 Graphics ▼ Next Match ▲ Previous Match

Score	Expect	Identities	Gaps	Strand
315 bits(170)	5e-90	170/170(100%)	0/170(0%)	Plus/Minus

```
Query  12   CTTGGAGGTGGTAGAAGAGACTCATTTGGGCCTTTTCCATTGTCCACAACAAACGCCATC  71
            ||||||||||||||||||||||||||||||||||||||||||||||||||||||||||||
Sbjct  452  CTTGGAGGTGGTAGAAGAGACTCATTTGGGCCTTTTCCATTGTCCACAACAAACGCCATC  393

Query  72   TTAAGCCCCCATGTTGTATGCACTTCCAAATGGCAATGCATGAACCAAACCCCTGGATTA  131
            ||||||||||||||||||||||||||||||||||||||||||||||||||||||||||||
Sbjct  392  TTAAGCCCCCATGTTGTATGCACTTCCAAATGGCAATGCATGAACCAAACCCCTGGATTA  333

Query  132  TCTGCCCTAAACCTTATGGCAGTCCATCCACCAGATGGAACCCCAATGGT  181
            ||||||||||||||||||||||||||||||||||||||||||||||||||
Sbjct  332  TCTGCCCTAAACCTTATGGCAGTCCATCCACCAGATGGAACCCCAATGGT  283
```

5) GaLAC05_1

Cotton_A_13817 locus=CA_chr8:71027959:71034475:-
Sequence ID: lcl|Query_235091 **Length:** 752 **Number of Matches:** 1

Range 1: 334 to 536 Graphics ▼ Next Match ▲ Previous Match

Score	Expect	Identities	Gaps	Strand
340 bits(184)	8e-98	197/203(97%)	1/203(0%)	Plus/Minus

```
Query  8    TTGATTACGGTGTGATAACTGATGAACTCTCTT-AACATATGGGCAAATCTGGCGGCGGC  66
            |||||||||| |||||||||||||||  ||||| |||||||||||||||||||||||||||
Sbjct  536  TTGATTACGGCGTGATAACTGATGAAACCTCTTAAACATATGGGCAAATCTGGCGGCGGC  477

Query  67   AGTTGGATGGTCTGCAATTCACCAACTCCATTTTCGACAAGGAAAGCCATTGCCAAACCC  126
             |||||||||||||||||| ||||||||||||||||||||||||||||||||||||||||
Sbjct  476  GGTTGGATGGTCTGCAATTTACCAACTCCATTTTCGACAAGGAAAGCCATTGCCAAACCC  417

Query  127  CAATTGATATGAACATCCAGGTGACAGTGCATGATCCACACTCCTGGATTATCAGCGACG  186
            ||||||||||||||||||||||||||||||||||||||||||||||||||||||||||||
Sbjct  416  CAATTGATATGAACATCCAGGTGACAGTGCATGATCCACACTCCTGGATTATCAGCGACG  357

Query  187  AATCTAATGACTGCCCATCCATT  209
            |||||||||||||||||||||||
Sbjct  356  AATCTAATGACTGCCCATCCATT  334
```

6) GaLAC07_2

lcl|Cotton_A_30033 locus=CA_chr12:111604680:111608069:+
Sequence ID: lcl|Query_29003 **Length:** 754 **Number of Matches:** 1

Range 1: 432 to 610 Graphics ▼ Next Match ▲ Previous Match

Score	Expect	Identities	Gaps	Strand
220 bits(119)	1e-61	161/179(90%)	16/179(8%)	Plus/Minus

```
Query  18   TCTGTTTTC--NNATT-AAGGGACCATATATGAAATCAAATCCAAATAAATATTAAGAAA  74
            |||||||||    ||| |||||||||||||||||||||||||||||||||||||||||||
Sbjct  610  TCTGTTTTCATAAATTAAAGGGACCATATATGAAATCAAATCCAAATAAATATTAAGAAA  551

Query  75   CTTCAGCCATGGTTTTGTGCAAAATTTGAACTTCTAGCAATTTGGT-------------A  121
            ||||||||||||||||||||||||||||||||||||||||||||||             |
Sbjct  550  CTTCAGCCATGGTTTTGTGCAAAATTTGAACTTCTAGCAATTTGGTCTAGCAATTTGGTA  491

Query  122  GATCTACCGGAGGTAAAGGCAAAGTTGTTTCGGGAGTTGGTCCATTCTCAACCATGAAA  180
            |||||||||||||||||||||||||||||||||||||||||||||||||||||||||||
Sbjct  490  GATCTACCGGAGGTAAAGGCAAAGTTGTTTCGGGAGTTGGTCCATTCTCAACCATGAAA  432
```

7) GaLAC11_2

lcl|Cotton_A_17036 locus=CA_chr9:56144460:56146555:+

Sequence ID: lcl|Query_147669 **Length:** 734 **Number of Matches:** 1

Range 1: 351 to 573 Graphics ▼ Next Match ▲ Previous Match

Score	Expect	Identities	Gaps	Strand
324 bits(175)	9e-93	209/223(94%)	13/223(5%)	Plus/Minus

```
Query  14   TTGNTTTTTG-AAATACATTTATAAGCAAACTTGGGTGGTGTTTGTTGAACATTGAGAAG  72
            ||| |||||| |||||||||||||||||||||||||||||||||||||||||||||||||
Sbjct  573  TTGGTTTTTGAAAATACATTTATAAGCAAACTTGGGTGGTGTTTGTTGAACATTGAGAAG  514

Query  73   CTCTTTCTAACAGTTTAACATGGTGGCAAA------------TCCTTGGGTGGAGGCAGA  120
            ||||||||||||||||||||||||||||||            ||||||||||||||||||
Sbjct  513  CTCTTTCTAACAGTTTAACATGGTGGCAAACATGGTGGCAAATCCTTGGGTGGAGGCAGA  454

Query  121  ATTGATTGATCAGCTCCTTTTCCATCTTCAACCTCAAATGCCATTTTCAATCCCCACCCA  180
            ||||||||||||||||||||||||||||||||||||||||||||||||||||||||||||
Sbjct  453  ATTGATTGATCAGCTCCTTTTCCATCTTCAACCTCAAATGCCATTTTCAATCCCCACCCA  394

Query  181  GTATGGAGCTCCAAATGACAATGCATAAACCAAACACCTGGAT  223
            |||||||||||||||||||||||||||||||||||||||||||
Sbjct  393  GTATGGAGCTCCAAATGACAATGCATAAACCAAACACCTGGAT  351
```

8) GaLAC14_2

lcl|Cotton_A_04517 locus=CA_chr6:105080230:105082613:+

Sequence ID: lcl|Query_204981 **Length:** 662 **Number of Matches:** 1

Range 1: 271 to 405 Graphics ▼ Next Match ▲ Previous Match

Score	Expect	Identities	Gaps	Strand
239 bits(129)	3e-67	133/135(99%)	1/135(0%)	Plus/Minus

```
Query  14   CGATGA-NACGAGGCATGGTAGATGGCCGTGGGCGGATGCTGGTTTCAATGGTGCTACCG  72
            ||||||  ||||||||||||||||||||||||||||||||||||||||||||||||||||
Sbjct  405  CGATGACAACGAGGCATGGTAGATGGCCGTGGGCGGATGCTGGTTTCAATGGTGCTACCG  346

Query  73   TTCCTCACAATCAAAACAGTGCTCATTCCCCAGCTAGTATGTCTTTCGAAATGGCAATGC  132
            ||||||||||||||||||||||||||||||||||||||||||||||||||||||||||||
Sbjct  345  TTCCTCACAATCAAAACAGTGCTCATTCCCCAGCTAGTATGTCTTTCGAAATGGCAATGC  286

Query  133  ATATACCACACCCCG  147
            |||||||||||||||
Sbjct  285  ATATACCACACCCCG  271
```

9) GaLAC14_5

lcl|Cotton_A_04526 locus=CA_chr6:104947203:104949548:+

Sequence ID: lcl|Query_231048 **Length:** 706 **Number of Matches:** 1

Range 1: 320 to 441 Graphics ▼ Next Match ▲ Previous Match

Score	Expect	Identities	Gaps	Strand
226 bits(122)	4e-63	122/122(100%)	0/122(0%)	Plus/Minus

```
Query  14   ACGAGGCATGGTAGATGGCGGTGGGCGCATGCTGGTTTCGGTGGTGGTTCCATTCCTCAC  73
            ||||||||||||||||||||||||||||||||||||||||||||||||||||||||||||
Sbjct  441  ACGAGGCATGGTAGATGGCGGTGGGCGCATGCTGGTTTCGGTGGTGGTTCCATTCCTCAC  382

Query  74   AATCAAAACAGTACTCATTCCCCAGCTACTATGCCTTTCGAAATGGCAATGCATATACCA  133
            ||||||||||||||||||||||||||||||||||||||||||||||||||||||||||||
Sbjct  381  AATCAAAACAGTACTCATTCCCCAGCTACTATGCCTTTCGAAATGGCAATGCATATACCA  322

Query  134  CA  135
            ||
Sbjct  321  CA  320
```

10) GaLAC17_5

lcl|Cotton_A_12054 locus=CA_chr11:77398558:77400798:+

Sequence ID: lcl|Query_160362 **Length:** 732 **Number of Matches:** 1

Range 1: 405 to 630 Graphics ▼ Next Match ▲ Previous Match

Score	Expect	Identities	Gaps	Strand
333 bits(180)	1e-95	213/226(94%)	13/226(5%)	Plus/Minus

```
Query  27   AAACAGTGAAGATCGGGGGGACCTAACAAGTGCTATATCGTTTCGTATATAAAGTGTATA  86
            ||||||||||||||||||||||||||||||||||||||||||||||||||||||||||||
Sbjct  630  AAACAGTGAAGATCGGGGGGACCTAACAAGTGCTATATCGTTTCGTATATAAAGTGTATA  571

Query  87   GGAAAGGAAATAAGAGAATATAGTGGAAAATTGTATCGAAACATGGTAAAGACGACAACG  146
            ||||||||||||||||||||||||||||||||||||||||||||||||||||||||||||
Sbjct  570  GGAAAGGAAATAAGAGAATATAGTGGAAAATTGTATCGAAACATGGTAAAGACGACAACG  511

Query  147  ATGGGTGGGGTTTCAGCATTTAGGAAGA-------------TCGGCAGGCGGAGGTGGTA  193
            ||||||||||||||||||||||||||||             |||||||||||||||||||
Sbjct  510  ATGGGTGGGGTTTCAGCATTTAGGAAGAGCATTTAGGAAGATCGGCAGGCGGAGGTGGTA  451

Query  194  GCTTCTGTTTGCGCCCTTTTCCGTCGTTGACCACCCAAGCCATCTT  239
            ||||||||||||||||||||||||||||||||||||||||||||||
Sbjct  450  GCTTCTGTTTGCGCCCTTTTCCGTCGTTGACCACCCAAGCCATCTT  405
```

Supplementary Table S1: Details of BlastP similarity search perform to identify the cotton laccases using Arabidopsis laccase proteins as query. After the validation with conserve domain analysis, protein identified as laccases are highlighted with red text.

Query id	Subject id	% identity	alignment length	mismatches	gap openings	q. start	q. end	s. start	s. end	e-value	bit score
**AtLAC01**	Cotton_A_07013	52.42	557	254	6	28	581	31	579	8.00E-164	573
	Cotton_A_00902	52.6	557	250	6	28	581	31	576	8.00E-164	573
	Cotton_A_00905	52.24	557	252	6	28	581	31	576	2.00E-163	572
	Cotton_A_00947	51.71	557	255	6	28	581	31	576	6.00E-161	564
	Cotton_A_19723	52.16	556	245	6	26	581	29	563	6.00E-160	561
	Cotton_A_17036	52.82	549	234	8	35	581	63	588	7.00E-160	560
	Cotton_A_12054	47.71	589	268	4	25	581	34	614	8.00E-159	557
	Cotton_A_30645	51.52	559	252	8	28	581	33	577	3.00E-158	555
	Cotton_A_30646	51.16	559	254	8	28	581	33	577	1.00E-156	550
	Cotton_A_30643	50.81	559	256	8	28	581	33	577	4.00E-155	545
	Cotton_A_00882	49.47	562	259	8	22	581	25	563	7.00E-155	544
	Cotton_A_06597	47.94	557	266	8	27	581	22	556	6.00E-149	524
	Cotton_A_05572	47.76	557	267	8	27	581	22	556	5.00E-147	518
	Cotton_A_12917	48.85	563	262	9	21	581	18	556	7.00E-147	517
	Cotton_A_13818	48.94	566	266	9	23	581	19	568	4.00E-145	511
	Cotton_A_13553	49.08	546	252	8	38	581	59	580	6.00E-144	508
	Cotton_A_32213	49.11	564	259	10	21	581	17	555	8.00E-143	504
	Cotton_A_20282	48.49	563	263	10	21	581	19	556	1.00E-142	503
	Cotton_A_00335	47.58	559	267	8	26	581	23	558	1.00E-142	503
	Cotton_A_13820	48.82	553	262	7	35	581	1	538	4.00E-142	501
	Cotton_A_04178	49.47	566	262	12	23	581	28	576	8.00E-142	500
	Cotton_A_26217	46.77	558	246	7	24	581	29	535	9.00E-142	500
	Cotton_A_24290	50.09	557	251	11	33	581	39	576	2.00E-140	496
	Cotton_A_31477	48.19	554	260	9	35	581	1	534	7.00E-139	491
	Cotton_A_15837	48.19	554	265	8	35	581	1	539	8.00E-138	487
	Cotton_A_20043	51.08	509	229	7	35	539	1	493	7.00E-137	484
	Cotton_A_13817	47.08	565	255	8	23	581	19	545	2.00E-134	476
	Cotton_A_30033	46.77	558	269	9	33	581	29	567	4.00E-134	475
	Cotton_A_25874	44.52	566	285	9	23	581	13	556	6.00E-129	457
	Cotton_A_14417	44.54	568	284	10	22	581	24	568	7.00E-128	454
	Cotton_A_35771	46.58	556	269	10	33	581	30	564	1.00E-126	450
	Cotton_A_26221	42.23	547	218	8	35	581	1	449	2.00E-122	436
	Cotton_A_04513	45.73	527	272	7	58	581	12	527	2.00E-118	422
	Cotton_A_30034	44.42	556	287	9	33	581	30	570	2.00E-117	419
	Cotton_A_30035	44.53	539	268	11	39	564	5	525	6.00E-117	418
	Cotton_A_22687	43.33	547	281	12	30	564	3	532	9.00E-115	410
	Cotton_A_04514	41.49	564	305	10	22	581	21	563	1.00E-112	403
	Cotton_A_37880	42.11	551	297	8	35	581	36	568	3.00E-112	402
	Cotton_A_10403	38.27	554	322	6	31	581	29	565	5.00E-107	385
	Cotton_A_04517	39.82	565	308	11	25	581	20	560	2.00E-106	383
	Cotton_A_04526	39.39	556	301	12	31	581	24	548	4.00E-100	362
	Cotton_A_04522	39.77	523	289	10	25	542	18	519	2.00E-92	336
	Cotton_A_04519	38.46	429	239	11	35	459	157	564	6.00E-77	285
	Cotton_A_00379	53.04	247	113	2	336	581	24	268	4.00E-62	236
	Cotton_A_11229	31.45	585	310	23	29	571	34	569	8.00E-51	198
	Cotton_A_41171	31.33	581	330	21	22	571	19	561	1.00E-50	197
	Cotton_A_05112	28.47	590	318	22	23	571	25	551	2.00E-44	177
	Cotton_A_04527	33.22	292	171	5	24	313	23	292	2.00E-41	167
	Cotton_A_04521	34.94	269	157	6	24	290	23	275	1.00E-40	165
	Cotton_A_04515	33.91	289	167	6	24	310	23	289	1.00E-40	164
	Cotton_A_04523	32.88	292	172	5	24	313	23	292	2.00E-40	164
	Cotton_A_20741	33.21	271	163	5	22	290	22	276	1.00E-39	160
	Cotton_A_04520	39.46	185	108	2	24	206	23	205	3.00E-36	150
	Cotton_A_14657	38.34	253	118	9	216	465	3	220	4.00E-36	149
	Cotton_A_29171	25.75	536	341	18	56	581	2	490	6.00E-35	145
	Cotton_A_03574	25.35	572	342	19	33	581	27	536	4.00E-33	139
	Cotton_A_16611	27.89	570	343	19	31	581	30	550	6.00E-33	139
	Cotton_A_30797	25.43	527	291	21	47	547	47	497	3.00E-30	130
	Cotton_A_04677	25.48	522	333	15	26	543	24	493	6.00E-30	129
	Cotton_A_25793	25.14	533	310	17	29	541	15	478	4.00E-29	126
	Cotton_A_32443	24.53	530	339	19	24	547	24	498	6.00E-29	125
	Cotton_A_21767	25	528	329	18	29	547	31	500	1.00E-28	124
	Cotton_A_17325	25.34	517	329	18	29	541	27	490	2.00E-28	124
	Cotton_A_33725	26.15	520	285	18	47	541	48	493	3.00E-28	123
	Cotton_A_31751	24.35	542	349	15	31	556	30	526	5.00E-28	122
	Cotton_A_28552	24.86	523	324	15	29	541	27	490	6.00E-28	122
	Cotton_A_02508	23.79	538	339	14	31	554	29	509	2.00E-27	120

	Cotton_A_36377	25.37	536	326	16	28	547	10	487	3.00E-27	120
	Cotton_A_02504	25.79	535	325	15	28	547	26	503	5.00E-27	119
	Cotton_A_25524	24.81	524	330	16	29	544	27	494	5.00E-27	119
	Cotton_A_02507	25.79	535	325	15	28	547	26	503	6.00E-27	119
	Cotton_A_02505	24.44	532	330	15	31	547	11	485	8.00E-27	119
	Cotton_A_27307	24.44	532	330	15	31	547	13	487	8.00E-27	119
	Cotton_A_02506	24.44	532	330	15	31	547	11	485	3.00E-26	117
	Cotton_A_29170	22.73	550	365	17	43	578	44	547	5.00E-26	115
	Cotton_A_02502	24.25	532	331	15	31	547	29	503	1.00E-25	115
	Cotton_A_02040	25.98	535	309	20	31	547	31	496	2.00E-25	114
	Cotton_A_29696	27.37	285	187	8	29	310	27	294	3.00E-24	110
	Cotton_A_33724	26.05	526	308	18	31	541	29	488	1.00E-22	105
	Cotton_A_13811	62.03	79	26	2	92	168	2	78	2.00E-21	100
	Cotton_A_16427	24.91	281	195	7	31	309	16	282	7.00E-21	99
	Cotton_A_04525	32.67	150	89	2	164	313	6	143	9.00E-16	82.4
	Cotton_A_16999	23.67	169	126	3	31	198	31	197	3.00E-11	67
**AtLAC02**	Cotton_A_30646	73.37	567	149	2	8	573	12	577	0	862
	Cotton_A_30645	72.66	567	153	2	8	573	12	577	0	858
	Cotton_A_30643	72.66	567	153	2	8	573	12	577	0	857
	Cotton_A_07013	71.1	571	157	3	9	573	11	579	0	845
	Cotton_A_00902	72.36	550	147	3	27	573	29	576	0	838
	Cotton_A_00905	71.27	550	153	3	27	573	29	576	0	827
	Cotton_A_00947	71.09	550	154	3	27	573	29	576	0	822
	Cotton_A_12054	62.06	601	191	4	8	573	16	614	0	758
	Cotton_A_20043	73.24	497	128	5	36	531	1	493	0	743
	Cotton_A_06597	56.45	574	231	8	1	573	1	556	8.00E-180	627
	Cotton_A_12917	55.65	575	234	10	1	573	1	556	2.00E-178	622
	Cotton_A_20282	55.92	574	232	9	1	573	3	556	3.00E-178	622
	Cotton_A_00882	53.52	568	248	8	7	573	11	563	3.00E-176	615
	Cotton_A_05572	54.69	565	239	7	9	573	9	556	1.00E-175	612
	Cotton_A_13553	51.42	599	246	8	1	573	1	580	4.00E-172	601
	Cotton_A_17036	51.35	592	244	9	9	573	14	588	6.00E-171	597
	Cotton_A_32213	52.62	572	252	7	2	573	3	555	7.00E-171	597
	Cotton_A_19723	54.08	551	238	7	24	573	27	563	3.00E-170	595
	Cotton_A_00335	54.28	573	247	7	1	573	1	558	3.00E-169	592
	Cotton_A_13818	51.39	576	251	10	12	573	8	568	2.00E-162	569
	Cotton_A_13820	52.36	552	235	9	36	573	1	538	1.00E-159	560
	Cotton_A_31477	52.81	551	230	10	36	573	1	534	2.00E-159	559
	Cotton_A_04178	51.47	577	249	10	9	573	19	576	2.00E-159	558
	Cotton_A_24290	51.91	549	250	7	31	573	36	576	8.00E-159	557
	Cotton_A_26217	49.39	571	241	10	7	573	9	535	2.00E-158	555
	Cotton_A_15837	51.55	547	248	8	36	573	1	539	4.00E-154	541
	Cotton_A_13817	49.48	576	239	11	12	573	8	545	5.00E-151	531
	Cotton_A_30033	48.74	556	271	7	24	573	20	567	1.00E-147	520
	Cotton_A_35771	49.27	548	263	6	31	573	27	564	4.00E-142	501
	Cotton_A_30034	45.71	571	298	6	11	573	4	570	1.00E-134	477
	Cotton_A_30035	47.44	527	261	6	40	556	5	525	2.00E-133	472
	Cotton_A_25874	46.42	558	277	11	24	573	13	556	1.00E-132	470
	Cotton_A_22687	46.34	533	270	6	34	556	6	532	2.00E-130	462
	Cotton_A_14417	45.26	559	286	10	22	573	23	568	4.00E-129	458
	Cotton_A_26221	45.45	539	203	12	36	573	1	449	6.00E-129	458
	Cotton_A_04514	41.11	574	318	8	2	573	8	563	2.00E-124	442
	Cotton_A_04513	48.95	523	252	8	59	573	12	527	2.00E-123	439
	Cotton_A_04517	38.99	577	329	11	1	573	3	560	6.00E-114	408
	Cotton_A_37880	38.56	568	334	10	10	573	12	568	7.00E-111	398
	Cotton_A_04526	38.13	577	324	12	1	573	1	548	4.00E-109	392
	Cotton_A_10403	38.74	573	327	11	9	573	9	565	2.00E-106	383
	Cotton_A_04522	39.7	534	301	10	1	531	1	516	4.00E-104	375
	Cotton_A_00379	66.42	268	88	2	307	573	2	268	6.00E-102	368
	Cotton_A_04519	37.41	417	243	11	36	447	157	560	1.00E-77	287
	Cotton_A_41171	31.13	559	312	20	30	552	26	547	1.00E-53	207
	Cotton_A_11229	29.49	573	335	17	9	547	13	550	3.00E-53	206
	Cotton_A_05112	30.51	590	324	20	11	563	11	551	3.00E-50	196
	Cotton_A_20741	36.18	304	172	6	8	311	8	289	2.00E-48	191
	Cotton_A_04521	36.59	276	154	5	20	292	18	275	1.00E-47	187
	Cotton_A_04527	35.39	308	175	6	10	317	11	294	2.00E-47	187
	Cotton_A_04523	35.57	298	171	5	20	317	18	294	2.00E-47	187
	Cotton_A_04515	37	273	157	5	20	292	18	275	5.00E-47	186
	Cotton_A_04520	35.75	207	125	1	1	207	7	205	1.00E-42	171
	Cotton_A_14657	42.15	223	99	8	215	436	1	194	5.00E-38	156
	Cotton_A_29171	27.34	534	326	18	57	573	2	490	6.00E-38	155

	Cotton_A_17325	26.13	532	346	16	8	538	10	495	9.00E-36	148
	Cotton_A_16611	26.42	564	350	15	32	573	30	550	1.00E-34	144
	Cotton_A_29170	24.63	544	358	15	44	573	44	549	5.00E-34	142
	Cotton_A_28552	25.95	528	344	16	12	538	14	495	2.00E-31	134
	Cotton_A_03574	25.13	565	345	17	34	573	25	536	2.00E-31	134
	Cotton_A_21767	25.48	526	345	15	9	533	15	494	2.00E-31	134
	Cotton_A_25524	25.54	509	330	14	30	533	27	491	4.00E-31	133
	Cotton_A_04677	25.2	508	333	14	30	533	27	491	5.00E-31	132
	Cotton_A_30797	24.86	555	331	17	8	539	6	497	1.00E-30	131
	Cotton_A_32443	25.64	507	331	15	30	533	29	492	2.00E-29	127
	Cotton_A_29696	25.66	534	346	18	11	538	7	495	4.00E-28	123
	Cotton_A_25793	25.79	508	329	14	30	533	15	478	8.00E-28	122
	Cotton_A_33725	26.88	506	322	14	32	533	32	493	4.00E-27	120
	Cotton_A_13811	66.67	78	26	0	92	169	1	78	4.00E-27	119
	Cotton_A_27307	25.39	512	347	15	29	539	10	487	5.00E-27	119
	Cotton_A_02505	25.39	512	347	15	29	539	8	485	6.00E-27	119
	Cotton_A_02506	25.71	490	329	15	51	539	30	485	1.00E-26	118
	Cotton_A_02502	25	532	363	18	10	539	6	503	2.00E-26	117
	Cotton_A_02040	25.82	488	316	14	48	533	47	490	2.00E-26	117
	Cotton_A_02508	23.49	545	377	13	32	573	29	536	2.00E-25	114
	Cotton_A_33724	24.8	496	319	14	48	537	45	492	1.00E-23	108
	Cotton_A_16427	28.68	265	178	5	32	295	16	270	8.00E-23	105
	Cotton_A_02507	24.9	494	328	19	51	539	48	503	9.00E-22	102
	Cotton_A_02504	25.2	492	329	19	51	539	48	503	9.00E-22	102
	Cotton_A_36377	23.42	491	339	15	51	539	32	487	1.00E-20	98.6
	Cotton_A_31751	23.95	526	344	18	32	538	30	518	3.00E-20	97.4
	Cotton_A_04525	35.1	151	85	4	167	317	8	145	1.00E-15	82
	Cotton_A_11986	24.59	423	276	15	116	532	77	462	6.00E-11	66.2
**AtLAC03**	Cotton_A_04178	76.98	543	124	1	28	570	35	576	0	889
	Cotton_A_24290	74.4	543	138	1	28	570	35	576	0	858
	Cotton_A_15837	68.15	540	168	3	34	570	1	539	0	771
	Cotton_A_13818	66	547	183	3	26	570	23	568	0	764
	Cotton_A_13820	66.6	539	177	3	34	570	1	538	0	758
	Cotton_A_31477	66.05	539	176	4	34	570	1	534	0	746
	Cotton_A_13817	63.07	547	176	4	26	570	23	545	0	716
	Cotton_A_17036	51.68	536	240	5	39	570	68	588	9.00E-164	573
	Cotton_A_00882	51.74	545	244	6	30	570	34	563	6.00E-163	570
	Cotton_A_19723	52.84	545	238	5	30	570	34	563	9.00E-163	570
	Cotton_A_07013	51.09	550	256	8	30	570	34	579	1.00E-161	566
	Cotton_A_00902	50.64	549	257	9	30	570	34	576	3.00E-161	565
	Cotton_A_30033	50.64	551	252	6	30	570	27	567	1.00E-160	563
	Cotton_A_00905	50.09	549	260	9	30	570	34	576	5.00E-160	561
	Cotton_A_06597	49.72	545	256	5	30	570	26	556	6.00E-160	561
	Cotton_A_00947	50.27	549	259	9	30	570	34	576	2.00E-159	559
	Cotton_A_12054	45.85	578	273	5	30	570	40	614	6.00E-159	557
	Cotton_A_30645	50.91	550	253	9	30	570	36	577	6.00E-158	554
	Cotton_A_30643	50.91	550	253	9	30	570	36	577	8.00E-158	553
	Cotton_A_35771	51.99	552	239	8	30	570	28	564	3.00E-157	552
	Cotton_A_12917	49.72	545	254	7	30	570	28	556	7.00E-157	550
	Cotton_A_30646	50.73	550	254	9	30	570	36	577	1.00E-156	550
	Cotton_A_05572	50.19	534	248	5	41	570	37	556	2.00E-156	549
	Cotton_A_20282	49.72	545	253	7	30	570	29	556	8.00E-155	544
	Cotton_A_30034	51.54	553	246	8	30	570	28	570	7.00E-154	540
	Cotton_A_00335	50.64	545	251	5	30	570	28	558	7.00E-154	540
	Cotton_A_30035	51.22	531	232	7	39	553	6	525	1.00E-149	527
	Cotton_A_25874	47.71	547	269	7	30	570	21	556	1.00E-149	526
	Cotton_A_13553	47.27	550	267	7	28	570	47	580	7.00E-149	524
	Cotton_A_22687	50.85	531	234	7	39	553	13	532	1.00E-148	523
	Cotton_A_14417	47.62	546	271	6	30	570	33	568	1.00E-147	520
	Cotton_A_20043	52.52	497	220	7	39	528	6	493	7.00E-147	517
	Cotton_A_26217	48.26	545	233	7	30	570	36	535	3.00E-144	508
	Cotton_A_32213	46.42	545	272	6	30	570	27	555	4.00E-143	505
	Cotton_A_04513	48.85	520	256	7	57	570	12	527	6.00E-142	501
	Cotton_A_04514	44.08	549	288	6	28	570	28	563	6.00E-133	471
	Cotton_A_10403	42.42	554	297	8	26	570	25	565	8.00E-129	457
	Cotton_A_04517	41.74	551	299	7	28	570	24	560	2.00E-127	452
	Cotton_A_37880	40.91	550	307	8	28	570	30	568	5.00E-124	441
	Cotton_A_04526	40.18	550	299	8	28	570	22	548	7.00E-123	437
	Cotton_A_26221	44.03	536	204	11	39	570	6	449	5.00E-118	421
	Cotton_A_04522	41.68	511	278	6	28	531	22	519	1.00E-117	420
	Cotton_A_04519	39.36	404	220	8	41	435	164	551	6.00E-82	301

	Cotton_A_00379	53.39	251	107	6	326	570	22	268	5.00E-68	255
	Cotton_A_11229	31.23	538	306	14	43	548	49	554	2.00E-60	230
	Cotton_A_41171	32.71	538	298	19	43	548	41	546	3.00E-59	226
	Cotton_A_05112	29.44	540	298	18	43	548	46	536	1.00E-57	221
	Cotton_A_20741	36.84	285	161	6	26	310	27	292	2.00E-50	197
	Cotton_A_04521	38.85	260	146	4	28	287	28	274	2.00E-49	194
	Cotton_A_04515	37.99	279	157	5	30	305	30	295	3.00E-49	193
	Cotton_A_04523	36.14	285	163	4	28	312	28	293	5.00E-48	189
	Cotton_A_04527	36.49	285	162	5	28	312	28	293	1.00E-47	188
	Cotton_A_04520	42.7	178	102	0	28	205	28	205	3.00E-44	176
	Cotton_A_04677	29.94	501	305	18	34	530	33	491	3.00E-43	173
	Cotton_A_25524	29.3	488	301	16	46	530	45	491	1.00E-40	164
	Cotton_A_29171	26.1	521	348	11	55	570	2	490	1.00E-40	164
	Cotton_A_03574	28.65	534	328	20	49	570	44	536	2.00E-39	160
	Cotton_A_16611	28.68	523	333	17	30	541	30	523	1.00E-38	158
	Cotton_A_29170	24.82	544	374	13	33	570	35	549	3.00E-37	153
	Cotton_A_17325	27.68	495	303	14	49	535	48	495	7.00E-37	152
	Cotton_A_02508	26.7	543	329	18	46	570	45	536	9.00E-36	148
	Cotton_A_28552	28.1	484	305	13	49	530	48	490	3.00E-35	146
	Cotton_A_30797	26.57	527	337	15	49	570	50	531	1.00E-34	145
	Cotton_A_14657	42.01	219	93	6	218	432	6	194	1.00E-34	144
	Cotton_A_02502	26.41	496	328	15	47	539	46	507	2.00E-34	144
	Cotton_A_02505	26.16	497	328	15	47	539	28	489	3.00E-34	143
	Cotton_A_27307	26.16	497	328	15	47	539	30	491	3.00E-34	143
	Cotton_A_02040	27.14	490	306	16	46	530	47	490	5.00E-34	142
	Cotton_A_02506	26.16	497	328	15	47	539	28	489	1.00E-33	141
	Cotton_A_36377	25.1	498	332	15	47	539	30	491	2.00E-33	140
	Cotton_A_32443	26.49	487	315	13	46	530	47	492	5.00E-33	139
	Cotton_A_25793	27.69	520	291	16	34	530	21	478	1.00E-32	138
	Cotton_A_02504	25.51	494	328	14	47	536	46	503	2.00E-32	137
	Cotton_A_02507	25.51	494	328	14	47	536	46	503	2.00E-32	137
	Cotton_A_33724	26.76	497	303	16	46	532	45	490	1.00E-31	134
	Cotton_A_33725	26.62	541	326	15	46	570	48	533	2.00E-31	134
	Cotton_A_21767	26.72	494	305	15	46	530	49	494	1.00E-30	131
	Cotton_A_31751	27.27	528	326	19	34	541	37	526	3.00E-30	130
	Cotton_A_13811	73.08	78	21	0	90	167	1	78	8.00E-30	128
	Cotton_A_13811	67.5	40	13	0	531	570	79	118	5.00E-11	66.2
	Cotton_A_29696	31.7	265	162	6	49	310	48	296	3.00E-29	126
	Cotton_A_16427	29.77	262	171	6	30	288	16	267	1.00E-23	108
	Cotton_A_04525	35.81	148	84	3	165	312	8	144	7.00E-17	85.9
	Cotton_A_16999	34.42	154	98	3	46	198	47	198	4.00E-16	83.6
**AtLAC04**	Cotton_A_05572	79.67	541	109	1	19	558	16	556	0	903
	Cotton_A_06597	79.3	541	111	1	19	558	16	556	0	893
	Cotton_A_20282	78.7	540	113	1	19	558	19	556	0	883
	Cotton_A_12917	76.52	541	124	2	19	558	18	556	0	868
	Cotton_A_00335	75.6	541	131	1	19	558	18	558	0	830
	Cotton_A_32213	66.13	555	185	2	5	558	3	555	0	764
	Cotton_A_13553	61.79	581	193	3	5	558	2	580	0	739
	Cotton_A_19723	62.22	540	200	3	21	558	26	563	0	719
	Cotton_A_00882	61.3	540	205	3	21	558	26	563	0	717
	Cotton_A_17036	59.72	566	198	4	21	558	25	588	0	714
	Cotton_A_12054	55.99	584	212	5	20	558	31	614	0	665
	Cotton_A_07013	57.04	554	222	5	21	558	26	579	0	661
	Cotton_A_00905	57.89	551	219	5	21	558	26	576	0	657
	Cotton_A_00902	58.08	551	218	5	21	558	26	576	0	657
	Cotton_A_26217	57.67	541	195	4	20	558	27	535	0	654
	Cotton_A_00947	56.81	551	225	5	21	558	26	576	0	650
	Cotton_A_30645	55.49	546	231	6	25	558	32	577	5.00E-179	624
	Cotton_A_30646	54.99	551	236	6	20	558	27	577	2.00E-178	622
	Cotton_A_30643	54.63	551	238	6	20	558	27	577	2.00E-177	619
	Cotton_A_13818	50.81	553	252	7	22	558	20	568	1.00E-164	576
	Cotton_A_13820	52.14	537	237	7	38	558	6	538	5.00E-163	571
	Cotton_A_20043	54.97	493	213	5	33	516	1	493	3.00E-160	561
	Cotton_A_31477	52.35	533	238	7	38	558	6	534	1.00E-159	560
	Cotton_A_15837	51.49	538	240	7	38	558	6	539	4.00E-159	558
	Cotton_A_24290	50.09	543	261	5	26	558	34	576	4.00E-158	555
	Cotton_A_04178	50.27	551	256	6	22	558	30	576	7.00E-158	554
	Cotton_A_26221	52.08	528	172	5	33	558	1	449	2.00E-156	549
	Cotton_A_13817	49.19	553	238	8	22	558	20	545	4.00E-153	538
	Cotton_A_30033	49.45	548	265	6	22	558	21	567	4.00E-151	531
	Cotton_A_14417	47.82	550	269	6	21	558	25	568	8.00E-148	520

	Cotton_A_25874	48.35	544	263	6	27	558	19	556	1.00E-147	520
	Cotton_A_35771	46.85	540	280	4	26	558	25	564	4.00E-139	491
	Cotton_A_30034	46.73	550	279	8	22	558	22	570	2.00E-135	479
	Cotton_A_04514	44.24	538	294	6	26	558	27	563	5.00E-135	478
	Cotton_A_30035	47.02	521	260	6	37	541	5	525	2.00E-134	476
	Cotton_A_22687	46.92	520	260	6	38	541	13	532	7.00E-133	471
	Cotton_A_04513	46.9	516	261	6	56	558	12	527	9.00E-130	460
	Cotton_A_04517	43.15	540	298	5	26	558	23	560	3.00E-129	459
	Cotton_A_10403	39.78	548	313	7	23	558	23	565	2.00E-123	440
	Cotton_A_04526	41	539	301	7	26	558	21	548	3.00E-123	439
	Cotton_A_37880	41.14	542	308	7	26	558	29	568	7.00E-121	431
	Cotton_A_04522	42.48	499	282	4	26	519	21	519	7.00E-118	421
	Cotton_A_04519	42.36	406	226	7	36	435	160	563	3.00E-91	332
	Cotton_A_00379	56.85	241	99	3	323	558	28	268	9.00E-78	288
	Cotton_A_41171	32.16	541	324	15	23	536	22	546	5.00E-62	235
	Cotton_A_11229	31.85	540	327	14	23	536	30	554	1.00E-60	231
	Cotton_A_14657	49.76	211	89	2	211	421	1	194	5.00E-53	205
	Cotton_A_14657	59.32	59	14	1	510	558	178	236	3.00E-13	73.9
	Cotton_A_05112	30.81	542	313	17	24	536	28	536	3.00E-52	202
	Cotton_A_17325	28.07	538	357	12	22	558	22	530	9.00E-51	198
	Cotton_A_04515	38.69	274	154	4	20	292	21	281	2.00E-50	197
	Cotton_A_20741	39.62	260	143	4	28	286	30	276	3.00E-50	196
	Cotton_A_04521	38.66	269	145	5	22	286	23	275	4.00E-49	192
	Cotton_A_04523	37.5	264	151	4	22	284	23	273	2.00E-48	190
	Cotton_A_04520	45.41	185	101	0	22	206	23	207	2.00E-48	190
	Cotton_A_04527	37.12	264	152	4	22	284	23	273	3.00E-48	190
	Cotton_A_21767	26.95	538	363	12	22	558	26	534	3.00E-46	183
	Cotton_A_28552	26.67	540	366	12	20	558	20	530	5.00E-46	182
	Cotton_A_32443	27.12	520	345	14	22	538	24	512	1.00E-44	177
	Cotton_A_29696	26.47	544	362	12	20	558	20	530	2.00E-44	177
	Cotton_A_29171	26.97	519	335	15	54	558	2	490	4.00E-43	172
	Cotton_A_04677	28	550	341	17	23	558	23	531	1.00E-42	171
	Cotton_A_30797	26.46	514	331	12	20	524	22	497	3.00E-42	170
	Cotton_A_25793	27.09	550	345	13	23	558	11	518	1.00E-40	164
	Cotton_A_25524	26.9	539	361	13	23	558	23	531	2.00E-40	164
	Cotton_A_02040	26.5	532	335	15	22	540	24	512	1.00E-38	157
	Cotton_A_29170	24.34	530	365	10	41	558	44	549	6.00E-38	155
	Cotton_A_33725	27.86	499	314	12	29	518	32	493	6.00E-37	152
	Cotton_A_33724	26.65	514	321	15	22	522	22	492	1.00E-34	144
	Cotton_A_16611	25.86	553	355	18	29	558	30	550	2.00E-34	144
	Cotton_A_03574	25.24	523	349	14	48	558	44	536	1.00E-33	141
	Cotton_A_02508	25.28	538	364	14	29	558	29	536	4.00E-32	136
	Cotton_A_16427	25.98	508	320	15	29	523	16	480	1.00E-29	128
	Cotton_A_02506	25.83	511	328	16	29	524	11	485	2.00E-29	127
	Cotton_A_02504	25.38	528	354	16	29	548	29	524	6.00E-29	125
	Cotton_A_27307	25.83	511	328	16	29	524	13	487	9.00E-29	125
	Cotton_A_02507	25.38	528	354	16	29	548	29	524	1.00E-28	125
	Cotton_A_02505	25.83	511	328	16	29	524	11	485	1.00E-28	125
	Cotton_A_36377	24.9	506	339	16	29	524	13	487	1.00E-28	124
	Cotton_A_02502	25.69	510	330	15	29	524	29	503	4.00E-28	123
	Cotton_A_13811	66.67	78	26	0	89	166	1	78	3.00E-27	120
	Cotton_A_31751	23.8	521	339	15	29	523	30	518	2.00E-25	114
	Cotton_A_04525	33.87	124	76	3	162	284	6	124	1.00E-15	82
	Cotton_A_16999	25.42	177	129	3	22	197	24	198	2.00E-12	71.2
	Cotton_A_17000	27.37	285	169	9	243	518	3	258	3.00E-11	67
**AtLAC05**	Cotton_A_13818	78.6	570	114	2	11	580	7	568	0	947
	Cotton_A_13820	81.5	546	93	2	35	580	1	538	0	936
	Cotton_A_15837	79.85	546	103	2	35	580	1	539	0	916
	Cotton_A_13817	75.96	570	106	3	11	580	7	545	0	899
	Cotton_A_31477	77.66	546	110	2	35	580	1	534	0	889
	Cotton_A_04178	66.09	575	184	5	6	580	13	576	0	783
	Cotton_A_24290	66.12	552	177	4	29	580	35	576	0	768
	Cotton_A_30033	51.61	558	247	4	30	580	26	567	2.00E-172	602
	Cotton_A_00882	50.69	578	254	8	7	580	13	563	3.00E-169	592
	Cotton_A_30643	51.1	589	254	12	8	580	7	577	3.00E-166	582
	Cotton_A_35771	52.6	557	237	6	31	580	28	564	7.00E-166	580
	Cotton_A_30645	50.08	593	262	11	4	580	3	577	2.00E-165	579
	Cotton_A_00902	50.72	558	254	10	30	580	33	576	4.00E-164	575
	Cotton_A_30034	50.52	582	264	7	8	580	4	570	6.00E-164	574
	Cotton_A_30646	50.42	593	260	12	4	580	3	577	6.00E-164	574
	Cotton_A_00905	50.72	558	254	10	30	580	33	576	2.00E-163	572

	Cotton_A_07013	48.88	581	278	10	7	580	11	579	2.00E-162	569
	Cotton_A_12054	46.15	624	272	13	2	580	10	614	3.00E-162	568
	Cotton_A_00947	49.82	558	259	9	30	580	33	576	1.00E-161	566
	Cotton_A_06597	50.09	555	250	6	30	580	25	556	3.00E-161	565
	Cotton_A_19723	50.72	554	245	6	31	580	34	563	4.00E-161	565
	Cotton_A_17036	46.94	605	270	9	3	580	8	588	1.00E-160	563
	Cotton_A_05572	47.84	579	273	7	6	580	3	556	9.00E-158	553
	Cotton_A_20282	47.91	574	268	8	11	580	10	556	1.00E-157	553
	Cotton_A_22687	50.55	542	238	7	35	563	8	532	1.00E-157	553
	Cotton_A_30035	50.37	542	239	7	35	563	1	525	1.00E-156	550
	Cotton_A_12917	47.14	577	275	8	8	580	6	556	7.00E-156	547
	Cotton_A_14417	46.9	580	288	5	4	580	6	568	2.00E-154	543
	Cotton_A_25874	46.85	572	286	5	11	580	1	556	2.00E-152	536
	Cotton_A_13553	49.54	543	245	7	42	580	63	580	2.00E-151	533
	Cotton_A_00335	47.76	580	275	7	5	580	3	558	1.00E-150	530
	Cotton_A_26217	46.62	577	250	8	8	580	13	535	3.00E-149	525
	Cotton_A_20043	51.57	510	224	8	35	538	1	493	7.00E-148	521
	Cotton_A_32213	44.39	579	289	7	10	580	2	555	1.00E-144	509
	Cotton_A_04513	48.58	527	256	4	58	580	12	527	7.00E-144	507
	Cotton_A_04514	41.22	575	309	10	13	580	11	563	8.00E-129	457
	Cotton_A_04517	39.9	574	319	8	13	580	7	560	2.00E-127	452
	Cotton_A_10403	39.48	580	321	10	7	580	10	565	1.00E-125	447
	Cotton_A_26221	44.55	550	200	11	35	580	1	449	1.00E-123	440
	Cotton_A_37880	39.93	586	317	13	7	580	6	568	5.00E-119	425
	Cotton_A_04526	37.8	574	321	10	13	580	5	548	6.00E-117	418
	Cotton_A_04522	38.91	532	299	8	13	538	5	516	9.00E-112	400
	Cotton_A_04519	36.96	414	234	7	35	442	157	549	6.00E-81	298
	Cotton_A_00379	52.16	255	110	6	332	580	20	268	2.00E-68	257
	Cotton_A_41171	31.76	551	295	20	44	558	41	546	2.00E-59	227
	Cotton_A_11229	30.94	585	328	21	7	558	13	554	3.00E-57	219
	Cotton_A_05112	28.08	584	326	20	11	558	11	536	1.00E-55	214
	Cotton_A_20741	35.62	306	174	5	7	312	10	292	2.00E-52	203
	Cotton_A_04521	35.9	312	173	6	1	309	1	288	6.00E-52	202
	Cotton_A_04527	36.01	311	178	5	1	311	1	290	5.00E-51	199
	Cotton_A_04523	35.37	311	180	5	1	311	1	290	7.00E-50	195
	Cotton_A_04515	34.29	312	183	5	1	307	1	295	2.00E-49	194
	Cotton_A_04520	40.1	207	121	2	1	206	1	205	1.00E-47	188
	Cotton_A_29170	24.22	578	396	13	8	580	9	549	1.00E-35	148
	Cotton_A_04677	26.2	542	345	15	3	540	1	491	1.00E-34	145
	Cotton_A_14657	39.13	230	98	6	216	441	3	194	2.00E-34	143
	Cotton_A_25524	26.52	543	344	17	1	540	1	491	3.00E-34	143
	Cotton_A_13811	80.77	78	15	0	91	168	1	78	4.00E-34	143
	Cotton_A_13811	80	40	8	0	541	580	79	118	2.00E-13	73.9
	Cotton_A_30797	25.43	582	371	16	5	577	2	529	3.00E-33	140
	Cotton_A_17325	25	576	368	14	13	580	11	530	3.00E-33	140
	Cotton_A_03574	24.74	570	349	21	32	580	26	536	1.00E-30	131
	Cotton_A_02040	26.49	502	309	14	50	546	50	496	2.00E-30	131
	Cotton_A_29696	26.38	508	316	14	43	545	41	495	7.00E-30	129
	Cotton_A_25793	24.9	494	317	12	50	540	36	478	9.00E-30	128
	Cotton_A_16611	24.39	570	381	17	1	561	1	529	1.00E-29	128
	Cotton_A_28552	23.73	569	374	13	18	580	16	530	2.00E-29	127
	Cotton_A_29171	24.91	530	354	15	55	580	1	490	3.00E-29	127
	Cotton_A_02508	24.91	542	349	13	47	580	45	536	9.00E-29	125
	Cotton_A_33724	25.25	503	310	15	50	544	48	492	9.00E-27	118
	Cotton_A_32443	24.6	500	325	14	43	540	43	492	4.00E-26	116
	Cotton_A_21767	22.88	542	365	13	3	540	2	494	2.00E-25	114
	Cotton_A_02505	23.32	506	336	16	50	549	30	489	1.00E-24	111
	Cotton_A_27307	22.97	505	339	14	50	549	32	491	1.00E-24	111
	Cotton_A_02506	22.97	505	339	14	50	549	30	489	1.00E-24	111
	Cotton_A_02502	23.23	508	334	14	50	549	48	507	1.00E-23	108
	Cotton_A_33725	25.14	537	342	14	50	580	51	533	1.00E-23	108
	Cotton_A_02504	21.91	534	370	13	50	580	48	537	5.00E-22	102
	Cotton_A_02507	21.91	534	370	13	50	580	48	537	8.00E-22	102
	Cotton_A_31751	23.12	558	347	17	31	561	30	532	2.00E-21	101
	Cotton_A_36377	21.39	533	374	12	50	580	32	521	2.00E-21	100
	Cotton_A_16427	27.48	262	177	5	31	289	16	267	3.00E-20	97.1
	Cotton_A_04525	38.36	146	81	4	161	304	5	143	4.00E-19	93.6
	Cotton_A_16999	27.32	194	138	3	7	199	7	198	3.00E-12	70.5
**AtLAC06**	Cotton_A_14417	70.66	542	159	0	28	569	27	568	0	839
	Cotton_A_25874	69.19	542	167	0	28	569	15	556	0	824
	Cotton_A_06597	46.27	549	278	5	27	569	19	556	9.00E-149	523

Cotton_A_00905	44.32	555	284	9	31	569	31	576	2.00E-148	522
Cotton_A_15837	45.89	547	273	5	38	569	1	539	3.00E-147	518
Cotton_A_04178	46.92	552	275	8	29	569	32	576	3.00E-147	518
Cotton_A_13818	45.27	550	289	5	29	569	22	568	4.00E-147	518
Cotton_A_31477	46.55	537	279	4	38	569	1	534	5.00E-147	518
Cotton_A_30033	44.81	549	292	5	29	569	22	567	6.00E-147	518
Cotton_A_13820	45.84	541	281	5	38	569	1	538	6.00E-147	517
Cotton_A_12054	41.34	595	294	9	22	569	28	614	8.00E-147	517
Cotton_A_07013	44.46	560	279	11	31	569	31	579	1.00E-146	516
Cotton_A_00902	44.14	555	285	9	31	569	31	576	3.00E-146	515
Cotton_A_24290	45.7	547	281	7	33	569	36	576	3.00E-146	515
Cotton_A_19723	46.17	548	275	9	29	569	29	563	9.00E-146	513
Cotton_A_30645	45.39	553	280	9	31	569	33	577	3.00E-145	512
Cotton_A_00335	46.27	549	278	5	27	569	21	558	4.00E-145	511
Cotton_A_05572	44.44	549	288	5	27	569	19	556	2.00E-144	509
Cotton_A_12917	45.4	544	278	6	32	569	26	556	3.00E-144	509
Cotton_A_30643	45.03	553	282	9	31	569	33	577	2.00E-143	505
Cotton_A_00882	45.7	547	279	7	29	569	29	563	6.00E-143	504
Cotton_A_17036	45.05	555	281	11	23	569	50	588	2.00E-142	503
Cotton_A_00947	44.14	555	285	10	31	569	31	576	2.00E-142	502
Cotton_A_30646	44.67	553	284	9	31	569	33	577	5.00E-142	501
Cotton_A_13817	45.09	550	267	6	29	569	22	545	1.00E-141	500
Cotton_A_13553	45.49	532	271	6	44	569	62	580	4.00E-140	495
Cotton_A_20282	44.81	549	283	7	27	569	22	556	4.00E-140	495
Cotton_A_32213	42.81	549	295	6	27	569	20	555	1.00E-134	477
Cotton_A_35771	44.57	543	288	5	34	569	28	564	8.00E-134	474
Cotton_A_30034	43.94	553	297	6	27	569	21	570	2.00E-130	462
Cotton_A_30035	43.94	528	280	6	38	552	1	525	1.00E-128	457
Cotton_A_26217	43.17	549	260	11	29	569	31	535	1.00E-128	457
Cotton_A_22687	43.58	530	283	6	36	552	6	532	1.00E-128	457
Cotton_A_04514	41.27	550	303	8	29	569	25	563	9.00E-127	450
Cotton_A_20043	45.11	501	256	9	38	527	1	493	2.00E-126	449
Cotton_A_04513	43.7	524	272	6	61	569	12	527	8.00E-124	441
Cotton_A_04517	38.59	552	316	8	29	569	21	560	2.00E-121	432
Cotton_A_04526	40.54	555	291	14	29	569	19	548	3.00E-120	429
Cotton_A_10403	39.18	559	319	7	22	569	17	565	5.00E-118	421
Cotton_A_37880	38.92	555	312	12	29	569	27	568	2.00E-114	409
Cotton_A_04522	40.94	513	274	12	29	527	19	516	5.00E-109	391
Cotton_A_26221	39.03	538	233	11	38	569	1	449	2.00E-103	373
Cotton_A_04519	36.79	424	244	9	37	450	156	565	9.00E-79	291
Cotton_A_41171	30.73	563	326	18	27	553	21	555	6.00E-60	228
Cotton_A_00379	43.25	252	128	6	326	569	24	268	5.00E-57	219
Cotton_A_05112	27.98	554	323	18	27	547	26	536	2.00E-53	207
Cotton_A_04527	38.37	258	146	3	29	286	25	269	1.00E-50	197
Cotton_A_04523	37.21	258	149	2	29	286	25	269	5.00E-49	192
Cotton_A_04521	35.98	264	156	3	29	292	25	275	5.00E-47	186
Cotton_A_04515	35.61	264	157	3	33	296	29	279	2.00E-46	183
Cotton_A_20741	32.96	267	166	3	32	298	29	282	3.00E-43	173
Cotton_A_29170	25.56	532	362	14	46	569	44	549	9.00E-40	161
Cotton_A_17325	26.06	545	342	16	32	562	27	524	2.00E-39	160
Cotton_A_04677	26.48	506	323	12	32	529	27	491	1.00E-38	158
Cotton_A_03574	26.5	551	353	15	33	569	24	536	3.00E-38	157
Cotton_A_30797	26.29	544	354	13	32	569	29	531	6.00E-38	155
Cotton_A_16611	27.13	516	333	14	33	534	29	515	1.00E-37	154
Cotton_A_28552	26.05	522	330	11	32	542	27	503	1.00E-37	154
Cotton_A_29696	25.38	524	331	13	32	542	27	503	1.00E-36	151
Cotton_A_04520	37.57	181	113	0	29	209	25	205	1.00E-36	151
Cotton_A_25524	25.1	506	330	12	32	529	27	491	3.00E-35	146
Cotton_A_29171	26.31	536	313	20	59	567	2	482	4.00E-35	146
Cotton_A_25793	25	520	338	12	32	542	15	491	4.00E-34	142
Cotton_A_11229	30.9	288	169	8	21	285	23	303	3.00E-33	140
Cotton_A_11229	37.08	178	101	4	384	553	389	563	4.00E-24	110
Cotton_A_21767	24.27	515	348	12	32	542	31	507	4.00E-33	139
Cotton_A_02508	24.95	541	370	13	33	569	28	536	4.00E-33	139
Cotton_A_32443	24.33	522	339	15	32	542	29	505	7.00E-33	139
Cotton_A_31751	27.67	524	323	20	33	534	29	518	6.00E-31	132
Cotton_A_33725	28.63	524	305	20	33	541	31	500	1.00E-30	131
Cotton_A_02040	25.05	523	330	14	21	529	16	490	1.00E-29	127
Cotton_A_02505	25.73	517	346	15	28	538	5	489	4.00E-29	126
Cotton_A_27307	25.73	517	346	15	28	538	7	491	4.00E-29	126
Cotton_A_02502	24.81	524	342	15	28	538	23	507	1.00E-28	124
Cotton_A_02506	25.73	517	346	15	28	538	5	489	3.00E-28	123

	Cotton_A_33724	24.74	489	312	15	51	529	46	488	6.00E-28	122
	Cotton_A_36377	24.73	558	365	16	28	563	7	531	4.00E-27	119
	Cotton_A_02504	24.71	510	341	16	28	529	23	497	2.00E-26	117
	Cotton_A_02507	24.71	510	341	16	28	529	23	497	3.00E-26	117
	Cotton_A_16427	25.05	511	339	16	28	534	10	480	1.00E-25	114
	Cotton_A_14657	35.32	218	109	8	217	428	1	192	1.00E-25	114
	Cotton_A_13811	58.44	77	32	0	95	171	2	78	3.00E-21	100
	Cotton_A_04525	40.98	122	67	1	165	286	4	120	2.00E-20	97.4
	Cotton_A_16999	28.65	185	127	4	21	202	16	198	2.00E-14	78.2
**AtLAC07**	Cotton_A_30033	63.4	530	185	3	26	550	25	550	0	726
	Cotton_A_35771	65.28	527	176	4	26	550	26	547	0	719
	Cotton_A_30034	61.58	531	195	4	26	550	26	553	0	666
	Cotton_A_22687	60.9	532	192	5	30	550	6	532	0	663
	Cotton_A_30035	60.38	530	194	5	32	550	1	525	0	662
	Cotton_A_13820	50.75	530	241	7	32	550	1	521	5.00E-157	551
	Cotton_A_13818	50.28	535	246	7	27	550	26	551	8.00E-157	550
	Cotton_A_15837	51.04	531	239	8	32	550	1	522	6.00E-156	547
	Cotton_A_31477	51.42	529	235	9	32	550	1	517	1.00E-154	543
	Cotton_A_00882	50.67	525	247	7	27	550	33	546	8.00E-151	530
	Cotton_A_04178	49.34	533	250	8	28	550	37	559	1.00E-149	526
	Cotton_A_30643	49.53	533	251	6	28	550	36	560	1.00E-147	520
	Cotton_A_13817	48.97	535	230	8	27	550	26	528	1.00E-147	520
	Cotton_A_30645	49.63	534	249	7	28	550	36	560	2.00E-147	519
	Cotton_A_24290	48.78	533	253	8	28	550	37	559	2.00E-146	516
	Cotton_A_30646	48.97	533	254	6	28	550	36	560	6.00E-146	514
	Cotton_A_17036	48.94	519	255	6	32	550	63	571	7.00E-145	511
	Cotton_A_12054	46.19	565	255	9	28	550	40	597	1.00E-144	510
	Cotton_A_00902	47.38	534	262	6	28	550	34	559	4.00E-143	504
	Cotton_A_19723	48.09	524	262	5	27	550	33	546	6.00E-143	504
	Cotton_A_07013	47.1	535	265	5	28	550	34	562	7.00E-143	504
	Cotton_A_00905	47.19	534	263	6	28	550	34	559	2.00E-142	502
	Cotton_A_00947	46.99	532	267	5	28	550	34	559	4.00E-140	495
	Cotton_A_20043	50.7	497	228	7	37	525	6	493	8.00E-140	494
	Cotton_A_12917	47.04	523	266	6	28	550	28	539	2.00E-136	483
	Cotton_A_05572	46.97	528	261	7	28	550	26	539	2.00E-135	479
	Cotton_A_20282	46.85	523	266	6	28	550	29	539	5.00E-135	478
	Cotton_A_13553	46.98	513	261	5	38	550	62	563	2.00E-134	476
	Cotton_A_06597	46.97	528	261	7	28	550	26	539	3.00E-134	475
	Cotton_A_26217	46.86	525	237	8	27	550	35	518	2.00E-133	473
	Cotton_A_04513	45.76	507	262	6	52	550	9	510	8.00E-131	464
	Cotton_A_32213	45.12	523	276	5	28	550	27	538	6.00E-130	461
	Cotton_A_14417	44.26	531	276	8	28	550	33	551	2.00E-129	459
	Cotton_A_25874	43.34	533	278	9	28	550	21	539	7.00E-128	454
	Cotton_A_00335	45.75	529	268	7	27	550	27	541	3.00E-126	449
	Cotton_A_04514	41.97	529	289	12	28	550	30	546	2.00E-115	412
	Cotton_A_04517	38.4	526	313	7	28	550	26	543	2.00E-107	386
	Cotton_A_10403	38.72	532	305	9	28	550	29	548	2.00E-106	382
	Cotton_A_26221	41.63	514	213	7	37	550	6	432	3.00E-105	379
	Cotton_A_04522	40.24	507	284	10	28	527	24	518	9.00E-103	371
	Cotton_A_04526	39.06	530	294	11	28	550	24	531	2.00E-102	369
	Cotton_A_37880	39.16	526	302	11	32	550	36	550	2.00E-100	363
	Cotton_A_04519	38.96	403	236	8	26	424	151	547	2.00E-78	290
	Cotton_A_41171	32.41	543	320	18	27	545	27	546	2.00E-63	240
	Cotton_A_11229	30.52	534	314	18	41	545	49	554	7.00E-56	215
	Cotton_A_00379	43.75	256	130	5	303	550	2	251	4.00E-54	209
	Cotton_A_05112	29.96	534	302	17	41	545	46	536	6.00E-53	205
	Cotton_A_04527	38.22	259	146	4	28	286	30	274	2.00E-50	197
	Cotton_A_20741	38.46	260	146	5	28	287	31	276	8.00E-50	195
	Cotton_A_04521	37.69	260	148	5	28	287	30	275	2.00E-49	194
	Cotton_A_04515	35.98	264	155	4	28	291	30	279	7.00E-49	192
	Cotton_A_04523	36.68	259	150	4	28	286	30	274	2.00E-48	190
	Cotton_A_04520	40.91	176	104	0	28	203	30	205	3.00E-40	163
	Cotton_A_29170	27.83	503	323	18	34	527	38	509	5.00E-38	155
	Cotton_A_03574	26.61	511	321	15	30	527	27	496	9.00E-36	148
	Cotton_A_33725	29.25	506	308	15	28	527	32	493	2.00E-33	140
	Cotton_A_17325	26.89	502	325	12	28	527	29	490	2.00E-33	140
	Cotton_A_16611	27.29	513	332	14	26	527	28	510	2.00E-33	140
	Cotton_A_30797	27.08	517	316	17	28	533	31	497	6.00E-33	139
	Cotton_A_04677	27.18	504	320	17	29	527	30	491	1.00E-32	138
	Cotton_A_02508	26.13	509	336	14	29	533	30	502	2.00E-32	137
	Cotton_A_29171	26.28	506	325	17	53	547	2	470	3.00E-32	137

	Cotton_A_28552	27.09	491	314	14	40	527	41	490	3.00E-31	133
	Cotton_A_29696	26.27	491	318	14	40	527	41	490	3.00E-31	133
	Cotton_A_21767	25.15	501	331	13	30	527	35	494	7.00E-31	132
	Cotton_A_25793	25.9	502	330	11	28	527	17	478	8.00E-31	132
	Cotton_A_25524	26.19	504	325	15	29	527	30	491	3.00E-30	130
	Cotton_A_13811	71.05	76	22	0	90	165	3	78	1.00E-29	127
	Cotton_A_32443	24.08	490	330	11	40	527	43	492	1.00E-28	125
	Cotton_A_14657	36.11	216	113	6	211	424	1	193	8.00E-27	118
	Cotton_A_27307	25.31	490	327	17	48	533	33	487	2.00E-26	117
	Cotton_A_02505	25.31	490	327	17	48	533	31	485	2.00E-26	117
	Cotton_A_02040	25.44	507	322	14	29	527	32	490	4.00E-26	116
	Cotton_A_02506	25.2	492	325	17	48	533	31	485	1.00E-25	115
	Cotton_A_36377	25.15	493	324	16	48	533	33	487	1.00E-25	114
	Cotton_A_02502	24.69	490	330	17	48	533	49	503	4.00E-25	113
	Cotton_A_02504	24.95	489	330	16	48	533	49	503	9.00E-25	112
	Cotton_A_16427	26.88	491	310	16	41	527	30	475	1.00E-24	111
	Cotton_A_02507	25.36	489	328	17	48	533	49	503	1.00E-24	111
	Cotton_A_31751	24.33	522	339	16	26	527	28	513	2.00E-24	110
	Cotton_A_33724	24.65	507	328	14	28	527	29	488	4.00E-24	109
	Cotton_A_04525	39.23	130	73	2	161	290	6	129	1.00E-20	98.2
	Cotton_A_11986	23.96	384	244	14	151	525	117	461	2.00E-12	71.2
**AtLAC08**	Cotton_A_30033	55.97	536	228	3	20	549	17	550	0	644
	Cotton_A_35771	57.66	529	212	6	27	549	25	547	0	642
	Cotton_A_30034	55.41	545	231	6	14	549	12	553	0	635
	Cotton_A_30035	56.88	545	220	6	34	566	1	542	0	632
	Cotton_A_22687	55.14	535	225	6	27	549	1	532	0	630
	Cotton_A_13818	46.57	554	270	9	12	549	8	551	3.00E-146	515
	Cotton_A_04178	48.46	551	259	9	13	549	20	559	3.00E-144	509
	Cotton_A_13820	47.27	531	255	8	34	549	1	521	1.00E-143	507
	Cotton_A_13817	45.44	548	262	7	12	549	8	528	7.00E-142	501
	Cotton_A_31477	48.2	527	252	8	34	549	1	517	1.00E-141	500
	Cotton_A_24290	47.22	557	269	8	7	549	14	559	1.00E-140	496
	Cotton_A_19723	46.72	533	265	6	23	549	27	546	1.00E-139	493
	Cotton_A_15837	45.86	532	262	7	34	549	1	522	4.00E-139	491
	Cotton_A_17036	45.54	538	272	7	19	549	48	571	4.00E-138	488
	Cotton_A_00882	44.34	548	280	8	13	549	13	546	9.00E-138	487
	Cotton_A_00905	46.86	525	257	7	39	549	43	559	6.00E-135	478
	Cotton_A_00902	46.67	525	258	7	39	549	43	559	9.00E-135	477
	Cotton_A_06597	45.2	542	279	7	14	549	10	539	3.00E-133	472
	Cotton_A_05572	44.46	542	283	8	14	549	10	539	7.00E-133	471
	Cotton_A_12054	43.12	589	275	12	12	549	18	597	1.00E-132	470
	Cotton_A_07013	43.6	555	288	7	12	549	16	562	8.00E-132	467
	Cotton_A_00947	45.33	525	265	7	39	549	43	559	1.00E-131	467
	Cotton_A_32213	44.63	549	279	8	12	549	4	538	6.00E-131	464
	Cotton_A_12917	44.32	546	279	12	15	549	8	539	1.00E-130	463
	Cotton_A_13553	44.34	557	285	9	3	549	22	563	2.00E-130	462
	Cotton_A_30645	46.22	543	271	10	20	549	26	560	4.00E-130	461
	Cotton_A_30643	45.06	557	278	11	13	549	12	560	1.00E-129	460
	Cotton_A_20282	45.24	546	273	13	15	549	9	539	4.00E-129	458
	Cotton_A_30646	46.37	524	254	10	42	549	48	560	1.00E-126	450
	Cotton_A_00335	46.08	523	261	8	36	549	31	541	6.00E-125	444
	Cotton_A_26217	41.1	545	270	8	12	549	18	518	1.00E-122	437
	Cotton_A_20043	44.83	493	254	8	42	524	9	493	7.00E-121	431
	Cotton_A_04513	43.5	508	269	6	54	549	9	510	5.00E-120	428
	Cotton_A_14417	41.37	527	290	7	32	549	35	551	1.00E-116	417
	Cotton_A_25874	40.8	527	293	7	32	549	23	539	5.00E-116	415
	Cotton_A_04514	40.33	553	297	12	13	549	11	546	1.00E-111	400
	Cotton_A_04517	38.98	567	324	9	13	568	7	562	3.00E-107	385
	Cotton_A_10403	39.51	577	324	10	12	573	6	572	2.00E-105	380
	Cotton_A_37880	38.04	552	316	9	12	549	11	550	4.00E-104	375
	Cotton_A_04526	37.27	550	309	13	13	549	5	531	5.00E-97	352
	Cotton_A_04522	38.67	525	296	13	13	524	5	516	6.00E-97	351
	Cotton_A_26221	38.14	472	224	8	85	549	22	432	4.00E-94	342
	Cotton_A_04519	38.52	405	231	9	28	422	151	547	6.00E-76	281
	Cotton_A_41171	31.95	554	315	17	24	544	22	546	4.00E-66	249
	Cotton_A_11229	31.62	563	325	17	14	544	20	554	8.00E-64	241
	Cotton_A_00379	43.88	237	126	2	314	549	21	251	2.00E-56	217
	Cotton_A_05112	28.97	573	317	18	13	544	13	536	2.00E-55	214
	Cotton_A_04527	39.05	274	153	5	11	278	6	271	3.00E-49	193
	Cotton_A_04515	37.18	277	162	4	7	282	10	275	3.00E-48	190
	Cotton_A_20741	38.58	267	153	4	17	282	20	276	7.00E-48	188

	Cotton_A_04523	37.73	273	158	4	7	278	10	271	1.00E-47	187
	Cotton_A_04521	37.55	277	161	4	7	282	10	275	5.00E-47	186
	Cotton_A_30797	29.26	499	291	14	41	526	42	491	3.00E-40	163
	Cotton_A_29170	25.99	531	340	15	13	526	15	509	1.00E-37	155
	Cotton_A_04520	42.42	198	106	4	15	205	14	210	1.00E-36	151
	Cotton_A_04677	25.67	526	336	17	12	526	10	491	2.00E-34	144
	Cotton_A_29171	26.23	488	305	16	55	526	2	450	3.00E-34	143
	Cotton_A_21767	27.71	498	305	17	39	526	42	494	5.00E-34	142
	Cotton_A_17325	27.6	500	303	17	39	526	38	490	5.00E-34	142
	Cotton_A_25793	27.09	502	303	15	39	526	26	478	9.00E-33	138
	Cotton_A_32443	26.96	497	306	17	41	526	42	492	1.00E-32	138
	Cotton_A_33725	26.87	521	324	13	17	526	19	493	4.00E-32	136
	Cotton_A_29696	28.23	496	301	17	41	526	40	490	1.00E-31	134
	Cotton_A_28552	26.44	503	305	16	39	526	38	490	2.00E-31	134
	Cotton_A_03574	25.48	522	300	18	37	526	32	496	2.00E-31	134
	Cotton_A_14657	36.41	217	113	6	207	422	1	193	3.00E-31	133
	Cotton_A_16611	26.92	535	331	16	14	526	14	510	7.00E-31	132
	Cotton_A_25524	25.7	498	316	16	39	526	38	491	9.00E-31	132
	Cotton_A_02508	25.84	507	311	16	38	526	37	496	3.00E-30	130
	Cotton_A_02040	27.03	492	301	15	45	526	47	490	8.00E-30	128
	Cotton_A_16427	26.96	497	308	16	39	526	25	475	4.00E-28	123
	Cotton_A_33724	26.58	489	307	14	45	526	45	488	1.00E-27	121
	Cotton_A_31751	25.73	517	317	17	37	526	37	513	5.00E-26	116
	Cotton_A_13811	56.41	78	34	0	90	167	1	78	3.00E-23	106
	Cotton_A_36377	25.96	493	301	21	52	526	35	481	3.00E-21	100
	Cotton_A_02504	25.61	492	304	19	52	526	51	497	4.00E-21	100
	Cotton_A_02506	25.3	498	317	19	52	535	33	489	6.00E-21	99.4
	Cotton_A_02507	25.61	492	304	19	52	526	51	497	7.00E-21	99.4
	Cotton_A_02505	25.1	498	318	20	52	535	33	489	7.00E-21	99.4
	Cotton_A_27307	25.1	498	318	20	52	535	35	491	8.00E-21	99
	Cotton_A_02502	25.35	497	318	20	52	535	51	507	3.00E-20	97.1
	Cotton_A_04525	33.33	144	87	3	167	309	10	145	5.00E-16	83.2
**AtLAC09**	Cotton_A_30033	55.14	555	239	3	20	568	17	567	3.00E-180	628
	Cotton_A_35771	58.54	533	207	5	42	568	40	564	1.00E-177	620
	Cotton_A_30035	57.12	541	215	6	40	568	7	542	4.00E-176	614
	Cotton_A_22687	56.68	524	210	6	40	551	14	532	6.00E-176	614
	Cotton_A_30034	56.03	564	234	6	14	568	12	570	8.00E-174	607
	Cotton_A_13818	45.9	573	282	9	12	568	8	568	2.00E-140	496
	Cotton_A_13820	47.23	542	259	8	42	568	9	538	4.00E-140	495
	Cotton_A_31477	48.14	538	256	8	42	568	9	534	4.00E-139	491
	Cotton_A_04178	47.54	570	272	8	13	568	20	576	2.00E-137	486
	Cotton_A_24290	48.61	541	251	8	42	568	49	576	5.00E-137	484
	Cotton_A_13817	44.8	567	274	7	12	568	8	545	2.00E-136	483
	Cotton_A_15837	46.22	543	264	7	42	568	9	539	3.00E-136	482
	Cotton_A_17036	44.6	556	287	7	19	568	48	588	7.00E-134	474
	Cotton_A_12054	43.06	576	270	9	40	568	50	614	3.00E-133	472
	Cotton_A_00882	44.17	566	291	7	13	568	13	563	9.00E-133	471
	Cotton_A_19723	45.11	552	282	7	23	568	27	563	2.00E-132	469
	Cotton_A_00905	46.41	543	267	7	40	568	44	576	9.00E-132	467
	Cotton_A_00902	46.22	543	268	7	40	568	44	576	2.00E-131	466
	Cotton_A_05572	44.37	568	296	8	7	568	3	556	9.00E-131	464
	Cotton_A_06597	44.56	561	291	7	14	568	10	556	2.00E-130	462
	Cotton_A_30645	47.23	542	263	9	40	568	46	577	6.00E-130	461
	Cotton_A_30643	47.05	542	264	9	40	568	46	577	9.00E-130	461
	Cotton_A_12917	42.43	568	305	7	7	568	5	556	1.00E-129	460
	Cotton_A_32213	45.07	568	285	10	12	568	4	555	4.00E-129	458
	Cotton_A_07013	45.42	546	271	7	40	568	44	579	5.00E-129	458
	Cotton_A_13553	45.98	535	267	7	40	568	62	580	7.00E-129	457
	Cotton_A_00947	44.75	543	276	7	40	568	44	576	1.00E-128	457
	Cotton_A_30646	46.49	542	267	9	40	568	46	577	2.00E-127	453
	Cotton_A_20282	42.88	562	298	8	13	568	12	556	7.00E-126	447
	Cotton_A_00335	43.97	564	294	9	12	568	10	558	9.00E-123	437
	Cotton_A_20043	46.28	497	247	9	40	526	7	493	1.00E-118	423
	Cotton_A_26217	41.03	563	281	7	12	568	18	535	2.00E-118	422
	Cotton_A_14417	42.57	538	288	7	40	568	43	568	5.00E-118	421
	Cotton_A_25874	41.23	536	294	7	42	568	33	556	3.00E-115	412
	Cotton_A_04513	43.83	527	276	7	54	568	9	527	1.00E-114	410
	Cotton_A_04514	40.74	567	311	11	13	568	11	563	2.00E-109	393
	Cotton_A_10403	38.97	580	325	10	12	575	6	572	9.00E-108	387
	Cotton_A_04517	38.66	569	325	9	13	570	7	562	1.00E-104	377
	Cotton_A_37880	39.11	542	304	10	40	569	42	569	9.00E-101	364

	Cotton_A_04526	38.27	567	316	12	13	568	5	548	6.00E-99	358
	Cotton_A_04522	39.43	525	294	11	13	526	5	516	6.00E-94	342
	Cotton_A_26221	36.53	490	243	11	85	568	22	449	5.00E-77	285
	Cotton_A_04519	39.59	394	219	9	40	423	163	547	2.00E-70	263
	Cotton_A_41171	32.91	556	303	20	40	560	38	558	2.00E-62	237
	Cotton_A_00379	43.92	255	135	2	315	568	21	268	2.00E-60	231
	Cotton_A_11229	31.68	584	325	20	14	560	20	566	3.00E-60	229
	Cotton_A_05112	29.59	561	300	18	40	560	43	548	4.00E-54	209
	Cotton_A_04527	36.6	306	171	6	11	310	6	294	6.00E-44	176
	Cotton_A_20741	39.34	244	139	3	40	282	41	276	7.00E-43	172
	Cotton_A_04515	38.11	244	142	3	40	282	40	275	2.00E-42	170
	Cotton_A_04521	35.57	298	171	5	8	304	11	288	7.00E-42	169
	Cotton_A_04523	37.13	272	153	4	40	310	40	294	9.00E-42	168
	Cotton_A_30797	27.49	542	331	16	41	570	42	533	5.00E-37	152
	Cotton_A_04520	41.71	199	106	4	13	205	16	210	7.00E-36	149
	Cotton_A_29170	25.48	569	379	14	12	568	14	549	1.00E-35	148
	Cotton_A_29171	26.67	525	338	15	55	568	2	490	8.00E-34	142
	Cotton_A_04677	26.95	512	316	18	41	541	40	504	8.00E-32	135
	Cotton_A_14657	35.94	217	115	4	207	423	1	193	2.00E-30	130
	Cotton_A_29696	26.85	540	338	15	41	570	40	532	7.00E-30	129
	Cotton_A_25793	26.69	502	303	15	41	528	28	478	7.00E-30	129
	Cotton_A_02040	25.75	536	333	16	46	568	47	530	1.00E-29	128
	Cotton_A_17325	26.11	540	342	14	41	570	40	532	3.00E-29	127
	Cotton_A_28552	24.86	539	350	14	41	570	40	532	2.00E-28	124
	Cotton_A_33725	26.1	544	337	14	41	570	43	535	3.00E-28	124
	Cotton_A_21767	26.34	543	337	18	41	570	44	536	3.00E-28	124
	Cotton_A_25524	26.42	511	320	17	41	541	40	504	3.00E-27	120
	Cotton_A_33724	24.72	534	345	12	46	570	45	530	3.00E-27	120
	Cotton_A_02508	25	548	342	16	40	568	39	536	4.00E-27	119
	Cotton_A_32443	25.59	512	322	17	41	541	42	505	6.00E-27	119
	Cotton_A_16611	26.09	548	344	17	40	565	40	548	5.00E-26	116
	Cotton_A_03574	24.01	558	339	16	40	568	35	536	4.00E-25	113
	Cotton_A_13811	55.13	78	35	0	90	167	1	78	5.00E-23	106
	Cotton_A_02504	25.75	497	303	18	50	528	49	497	3.00E-22	103
	Cotton_A_16427	24.78	561	331	21	40	570	26	525	4.00E-22	103
	Cotton_A_02507	25.75	497	303	18	50	528	49	497	5.00E-22	103
	Cotton_A_36377	24.8	500	304	18	50	528	33	481	1.00E-21	102
	Cotton_A_02505	25.09	542	321	20	50	552	31	526	8.00E-21	99
	Cotton_A_31751	25.95	555	343	19	40	568	40	552	9.00E-21	98.6
	Cotton_A_27307	25.09	542	321	20	50	552	33	528	1.00E-20	98.6
	Cotton_A_02506	25.09	542	321	20	50	552	31	526	2.00E-20	97.8
	Cotton_A_02502	24.81	540	325	19	50	552	49	544	8.00E-20	95.9
	Cotton_A_04525	33.79	145	86	2	167	310	10	145	7.00E-13	72.4
	Cotton_A_16999	32.24	152	96	3	46	191	47	197	5.00E-12	69.7
**AtLAC10**	Cotton_A_06597	76.16	558	131	2	1	558	1	556	0	853
	Cotton_A_05572	75.77	553	132	2	6	558	6	556	0	848
	Cotton_A_12917	73.29	554	145	2	5	558	6	556	0	827
	Cotton_A_00335	72.38	554	152	1	5	558	6	558	0	815
	Cotton_A_20282	72.2	554	150	3	5	558	7	556	0	812
	Cotton_A_32213	66.24	551	183	2	8	558	8	555	0	752
	Cotton_A_13553	63.18	584	185	4	1	558	1	580	0	735
	Cotton_A_00882	61.4	557	209	4	3	558	12	563	0	691
	Cotton_A_19723	61.45	537	203	3	23	558	30	563	0	681
	Cotton_A_17036	58.16	576	209	5	10	558	18	588	0	673
	Cotton_A_26217	57.59	547	196	5	13	558	24	535	0	633
	Cotton_A_07013	55.24	563	238	5	10	558	17	579	4.00E-180	627
	Cotton_A_00902	55.54	551	232	5	20	558	27	576	1.00E-177	619
	Cotton_A_00905	55.17	551	234	5	20	558	27	576	3.00E-177	618
	Cotton_A_12054	52.16	602	242	7	1	558	15	614	9.00E-176	613
	Cotton_A_00947	54.45	551	238	5	20	558	27	576	4.00E-175	611
	Cotton_A_30645	53.31	574	248	8	4	558	5	577	5.00E-174	607
	Cotton_A_30646	53.31	574	248	8	4	558	5	577	2.00E-173	605
	Cotton_A_30643	53.14	574	249	8	4	558	5	577	6.00E-173	604
	Cotton_A_13818	50.35	570	261	6	3	558	7	568	1.00E-161	566
	Cotton_A_13820	52.6	538	237	5	35	558	5	538	1.00E-160	563
	Cotton_A_15837	51.49	538	242	5	36	558	6	539	1.00E-156	550
	Cotton_A_31477	51.22	533	246	5	36	558	6	534	4.00E-155	545
	Cotton_A_04178	49.73	565	264	8	7	558	19	576	9.00E-154	540
	Cotton_A_30033	51.1	546	258	3	22	558	22	567	8.00E-153	537
	Cotton_A_26221	51.89	528	175	5	31	558	1	449	2.00E-151	533
	Cotton_A_24290	50.36	548	254	7	24	558	34	576	4.00E-151	531

	Cotton_A_13817	48.6	570	248	7	3	558	7	545	8.00E-151	530
	Cotton_A_25874	48.81	547	264	4	22	558	16	556	2.00E-150	529
	Cotton_A_20043	53.13	495	221	5	31	516	1	493	2.00E-149	525
	Cotton_A_14417	48.99	547	263	4	22	558	28	568	8.00E-149	524
	Cotton_A_35771	48.07	543	271	4	24	558	25	564	2.00E-139	493
	Cotton_A_30034	44.99	569	296	7	6	558	3	570	7.00E-132	468
	Cotton_A_30035	45.87	521	268	5	35	541	5	525	9.00E-129	457
	Cotton_A_04514	42.01	557	315	5	5	558	12	563	4.00E-128	455
	Cotton_A_22687	45.03	533	277	6	24	541	1	532	7.00E-128	454
	Cotton_A_04513	47.67	516	259	4	54	558	12	527	9.00E-127	450
	Cotton_A_04517	42.47	558	312	5	5	558	8	560	3.00E-126	449
	Cotton_A_10403	39.86	562	329	6	3	558	7	565	2.00E-121	433
	Cotton_A_04526	41.04	558	310	7	5	558	6	548	7.00E-121	431
	Cotton_A_04522	41.81	519	293	5	5	519	6	519	5.00E-112	401
	Cotton_A_37880	39.72	564	326	10	4	558	10	568	1.00E-111	400
	Cotton_A_04519	41.67	396	224	5	31	422	157	549	2.00E-87	320
	Cotton_A_00379	54.77	241	104	3	323	558	28	268	1.00E-75	280
	Cotton_A_05112	30.05	559	331	15	5	536	11	536	8.00E-51	198
	Cotton_A_17325	28.11	555	364	12	8	558	7	530	1.00E-48	191
	Cotton_A_20741	38.99	277	156	3	9	285	13	276	1.00E-47	188
	Cotton_A_04521	38.3	282	151	5	7	285	14	275	3.00E-46	183
	Cotton_A_04515	38.61	259	146	3	27	285	30	275	4.00E-46	182
	Cotton_A_14657	46.7	212	95	3	210	421	1	194	5.00E-46	182
	Cotton_A_14657	57.63	59	15	1	510	558	178	236	3.00E-13	73.6
	Cotton_A_04527	38.46	260	147	3	24	283	27	273	4.00E-45	179
	Cotton_A_04523	38.46	260	147	3	24	283	27	273	5.00E-45	179
	Cotton_A_28552	27.39	555	368	12	8	558	7	530	2.00E-43	174
	Cotton_A_04677	27.03	518	336	14	11	518	6	491	1.00E-42	171
	Cotton_A_04520	42	200	112	1	5	204	12	207	1.00E-42	171
	Cotton_A_02040	28.4	507	313	13	43	540	47	512	5.00E-42	169
	Cotton_A_21767	26.52	558	368	16	5	558	15	534	7.00E-42	168
	Cotton_A_29696	26.6	515	346	11	25	538	27	510	1.00E-41	168
	Cotton_A_32443	27.13	516	342	13	25	538	29	512	4.00E-41	166
	Cotton_A_29171	27.73	512	342	12	52	558	2	490	8.00E-41	165
	Cotton_A_30797	27.32	538	328	14	5	524	5	497	8.00E-41	165
	Cotton_A_25524	27.33	516	333	13	13	518	8	491	7.00E-40	162
	Cotton_A_33724	27.98	504	315	11	27	522	29	492	2.00E-38	157
	Cotton_A_33725	28.49	502	309	11	27	518	32	493	2.00E-37	154
	Cotton_A_29170	27.45	572	371	16	3	558	6	549	3.00E-37	153
	Cotton_A_25793	26.34	558	359	14	13	558	1	518	3.00E-37	153
	Cotton_A_02508	26.3	540	358	16	27	558	29	536	6.00E-37	152
	Cotton_A_16611	26.89	543	364	15	27	558	30	550	2.00E-36	150
	Cotton_A_41171	37.06	286	153	10	20	283	21	301	9.00E-34	141
	Cotton_A_41171	39.51	162	92	3	380	536	386	546	1.00E-28	124
	Cotton_A_11229	33.88	304	171	9	6	283	10	309	2.00E-33	140
	Cotton_A_11229	38.41	164	91	3	380	536	394	554	2.00E-27	120
	Cotton_A_03574	25.66	530	346	16	43	558	41	536	2.00E-33	140
	Cotton_A_16427	26.26	514	313	15	27	523	16	480	6.00E-33	139
	Cotton_A_36377	25.59	512	339	14	46	549	32	509	3.00E-31	133
	Cotton_A_02506	25	532	355	15	27	549	11	507	1.00E-30	131
	Cotton_A_02504	26.55	516	329	16	46	549	48	525	1.00E-30	131
	Cotton_A_02505	25	532	355	15	27	549	11	507	1.00E-30	131
	Cotton_A_27307	25.25	507	338	14	27	524	13	487	2.00E-30	131
	Cotton_A_02507	26.55	516	329	16	46	549	48	525	2.00E-30	130
	Cotton_A_02502	24.85	507	340	14	27	524	29	503	4.00E-29	126
	Cotton_A_13811	67.95	78	25	0	87	164	1	78	5.00E-28	122
	Cotton_A_13811	70	40	12	0	519	558	79	118	9.00E-11	65.5
	Cotton_A_31751	25.53	521	332	17	27	523	30	518	1.00E-26	118
	Cotton_A_04525	35.48	124	75	2	160	283	6	124	1.00E-15	81.6
	Cotton_A_17000	27.72	285	169	8	242	518	3	258	2.00E-13	74.3
	Cotton_A_16999	28.24	170	119	3	27	195	31	198	3.00E-12	70.1
**AtLAC11**	Cotton_A_19723	81.6	538	99	0	20	557	26	563	0	925
	Cotton_A_00882	78.81	538	114	0	20	557	26	563	0	914
	Cotton_A_17036	75.53	564	112	1	20	557	25	588	0	910
	Cotton_A_26217	75.7	539	101	1	19	557	27	535	0	858
	Cotton_A_12917	63.82	539	194	1	19	557	19	556	0	736
	Cotton_A_06597	61.97	539	202	2	21	557	19	556	0	720
	Cotton_A_20282	62.2	537	201	2	21	557	22	556	0	718
	Cotton_A_05572	61.6	539	204	2	21	557	19	556	0	715
	Cotton_A_26221	66.16	526	101	2	32	557	1	449	0	701
	Cotton_A_13553	60.41	533	202	3	29	557	53	580	0	679

Cotton_A_00335	60.11	539	212	2	21	557	21	558	0	676
Cotton_A_32213	59.3	543	212	3	19	557	18	555	0	669
Cotton_A_00902	58.7	552	213	8	20	557	26	576	0	668
Cotton_A_07013	58.02	555	215	8	20	557	26	579	0	667
Cotton_A_00905	58.15	552	216	8	20	557	26	576	0	665
Cotton_A_00947	58.15	552	216	8	20	557	26	576	0	662
Cotton_A_12054	55.02	578	213	9	26	557	38	614	0	648
Cotton_A_30646	56.78	546	220	9	26	557	34	577	0	631
Cotton_A_30643	57.33	546	217	10	26	557	34	577	0	631
Cotton_A_30645	56.78	546	220	10	26	557	34	577	9.00E-180	627
Cotton_A_13818	51.09	552	250	6	22	557	21	568	2.00E-168	589
Cotton_A_04178	51.09	550	251	5	22	557	31	576	5.00E-168	587
Cotton_A_13820	51.85	542	241	6	32	557	1	538	3.00E-167	585
Cotton_A_15837	52.3	543	238	6	32	557	1	539	5.00E-165	578
Cotton_A_24290	50.46	547	253	5	25	557	34	576	8.00E-164	573
Cotton_A_31477	52.5	539	238	9	32	557	1	534	1.00E-163	573
Cotton_A_20043	56.48	494	204	6	32	515	1	493	9.00E-163	570
Cotton_A_13817	50	552	233	7	22	557	21	545	3.00E-160	562
Cotton_A_30033	49.91	547	263	5	22	557	21	567	2.00E-159	559
Cotton_A_14417	49	551	261	8	20	557	25	568	9.00E-154	540
Cotton_A_25874	49.45	550	254	11	23	557	16	556	4.00E-152	535
Cotton_A_35771	48.33	540	272	4	25	557	25	564	2.00E-147	519
Cotton_A_30034	46.73	550	280	6	21	557	21	570	4.00E-141	498
Cotton_A_30035	47.24	525	259	6	34	540	1	525	2.00E-140	496
Cotton_A_22687	45.68	532	273	4	25	540	1	532	8.00E-140	494
Cotton_A_04513	49.42	516	248	5	55	557	12	527	1.00E-137	486
Cotton_A_04514	43.78	539	299	3	23	557	25	563	6.00E-135	478
Cotton_A_04517	43.33	540	301	3	23	557	21	560	2.00E-132	469
Cotton_A_10403	42.28	544	307	5	21	557	22	565	3.00E-132	468
Cotton_A_04526	41.67	540	300	5	23	557	19	548	3.00E-127	452
Cotton_A_37880	41.39	546	305	8	23	557	27	568	3.00E-125	445
Cotton_A_04522	42.91	501	281	3	23	518	19	519	1.00E-119	427
Cotton_A_04519	39.95	408	239	5	32	434	157	563	7.00E-88	321
Cotton_A_00379	57.6	250	98	4	315	557	20	268	3.00E-77	286
Cotton_A_14657	65.24	210	58	1	211	420	1	195	3.00E-75	279
Cotton_A_11229	35.22	548	304	15	19	535	27	554	3.00E-75	279
Cotton_A_41171	32.72	544	321	14	21	536	21	547	2.00E-71	266
Cotton_A_05112	29.91	545	312	18	25	536	30	537	2.00E-60	230
Cotton_A_04515	36.8	269	157	3	25	293	27	282	3.00E-50	196
Cotton_A_04527	37.79	262	150	3	23	284	25	273	4.00E-50	196
Cotton_A_04521	37.88	264	151	4	23	286	25	275	7.00E-50	195
Cotton_A_04523	37.79	262	150	3	23	284	25	273	4.00E-49	192
Cotton_A_20741	36.33	278	158	4	28	305	31	289	5.00E-49	192
Cotton_A_17325	29.41	544	332	18	26	557	27	530	1.00E-46	184
Cotton_A_04677	29.06	523	320	19	47	557	48	531	1.00E-44	177
Cotton_A_29171	29.13	515	331	15	52	557	1	490	3.00E-44	176
Cotton_A_28552	30.21	523	313	18	47	557	48	530	9.00E-44	175
Cotton_A_04520	42.31	182	105	0	22	203	24	205	2.00E-43	173
Cotton_A_21767	28.29	516	332	15	47	557	52	534	2.00E-43	173
Cotton_A_29170	28.69	495	322	14	40	524	44	517	5.00E-43	172
Cotton_A_25524	28.68	523	322	19	47	557	48	531	1.00E-41	168
Cotton_A_30797	27.18	515	312	14	44	541	47	515	3.00E-41	166
Cotton_A_32443	28.43	496	317	16	47	537	50	512	4.00E-41	166
Cotton_A_29696	28.68	523	321	17	47	557	48	530	3.00E-40	163
Cotton_A_33725	28.66	506	317	13	44	541	48	517	3.00E-39	159
Cotton_A_25793	28.13	544	339	16	26	557	15	518	1.00E-38	158
Cotton_A_02040	29.61	510	301	18	44	539	47	512	1.00E-38	157
Cotton_A_03574	27.04	540	324	16	44	557	41	536	2.00E-38	157
Cotton_A_02508	27.49	542	347	17	28	557	29	536	3.00E-38	156
Cotton_A_16611	27.74	548	351	17	28	557	30	550	4.00E-38	156
Cotton_A_33724	28.25	485	304	15	44	521	45	492	4.00E-36	149
Cotton_A_16427	28.57	511	303	18	28	522	16	480	1.00E-34	144
Cotton_A_02505	27.1	487	314	16	47	523	30	485	9.00E-33	138
Cotton_A_27307	27.1	487	314	16	47	523	32	487	1.00E-32	138
Cotton_A_02506	26.54	486	318	15	47	523	30	485	3.00E-32	136
Cotton_A_02502	26.38	489	315	15	47	523	48	503	4.00E-32	136
Cotton_A_02504	28.49	516	318	18	47	552	48	522	6.00E-31	132
Cotton_A_02507	28.49	516	318	18	47	552	48	522	1.00E-30	131
Cotton_A_31751	26.86	551	341	18	28	550	30	546	2.00E-30	131
Cotton_A_36377	27.87	488	309	17	47	523	32	487	8.00E-30	128
Cotton_A_13811	57.95	88	37	0	89	176	2	89	7.00E-26	115
Cotton_A_04525	36.8	125	74	2	160	284	5	124	5.00E-17	86.3

	Cotton_A_17000	26.5	283	177	10	241	517	1	258	3.00E-13	73.9
	Cotton_A_16999	27.32	194	136	4	17	207	18	209	6.00E-11	66.2
**AtLAC12**	Cotton_A_13818	79.38	548	106	2	24	565	22	568	0	930
	Cotton_A_13820	79.96	539	101	2	33	565	1	538	0	920
	Cotton_A_15837	79.26	540	104	2	33	565	1	539	0	905
	Cotton_A_31477	79.25	535	108	2	33	565	1	534	0	902
	Cotton_A_13817	76.82	548	97	3	24	565	22	545	0	879
	Cotton_A_04178	65.76	549	177	5	24	565	32	576	0	760
	Cotton_A_24290	66.18	547	174	5	26	565	34	576	0	759
	Cotton_A_00882	54.76	546	232	6	24	565	29	563	3.00E-176	615
	Cotton_A_30033	52.46	549	251	3	24	565	22	567	2.00E-172	602
	Cotton_A_19723	53.48	546	239	6	24	565	29	563	1.00E-168	589
	Cotton_A_17036	52.51	537	240	6	33	565	63	588	4.00E-168	588
	Cotton_A_35771	53.83	548	239	5	25	565	24	564	4.00E-168	588
	Cotton_A_06597	52.39	544	245	5	26	565	23	556	3.00E-166	582
	Cotton_A_12054	49.83	584	241	10	27	565	38	614	4.00E-166	581
	Cotton_A_30643	53.72	551	236	10	27	565	34	577	4.00E-165	578
	Cotton_A_30645	53.36	551	238	10	27	565	34	577	1.00E-164	576
	Cotton_A_30034	53.08	552	245	6	24	565	23	570	2.00E-163	572
	Cotton_A_30646	52.99	551	240	10	27	565	34	577	6.00E-163	571
	Cotton_A_05572	50.74	544	254	5	26	565	23	556	1.00E-162	570
	Cotton_A_20282	51.47	544	247	7	26	565	26	556	3.00E-162	568
	Cotton_A_30035	53.12	529	231	6	33	548	1	525	4.00E-162	568
	Cotton_A_00902	50.91	552	251	9	27	565	32	576	1.00E-161	566
	Cotton_A_00905	50.91	552	251	9	27	565	32	576	2.00E-161	566
	Cotton_A_22687	52.74	529	233	6	33	548	8	532	2.00E-161	565
	Cotton_A_12917	50.92	544	251	6	26	565	25	556	3.00E-161	565
	Cotton_A_00947	50.36	552	254	10	27	565	32	576	3.00E-160	561
	Cotton_A_07013	50.45	555	252	9	27	565	32	579	4.00E-160	561
	Cotton_A_13553	52.64	530	235	5	40	565	63	580	8.00E-160	560
	Cotton_A_00335	51.65	546	250	5	24	565	23	558	6.00E-159	557
	Cotton_A_25874	49.54	541	268	3	27	565	19	556	2.00E-157	552
	Cotton_A_14417	50.28	541	264	3	27	565	31	568	9.00E-157	550
	Cotton_A_26217	49.45	546	231	7	24	565	31	535	5.00E-155	544
	Cotton_A_32213	48.9	546	263	6	24	565	22	555	3.00E-154	541
	Cotton_A_20043	53.4	500	217	8	33	523	1	493	1.00E-149	526
	Cotton_A_04513	50.19	520	245	4	56	565	12	527	1.00E-146	516
	Cotton_A_04514	44.44	549	288	7	24	565	25	563	2.00E-135	479
	Cotton_A_10403	43.01	551	296	9	24	565	24	565	1.00E-134	477
	Cotton_A_04517	43.14	547	299	6	24	565	21	560	2.00E-134	476
	Cotton_A_26221	46.43	532	193	10	38	565	6	449	4.00E-129	458
	Cotton_A_37880	42.86	553	294	12	24	565	27	568	8.00E-128	454
	Cotton_A_04526	40.95	547	301	8	24	565	19	548	5.00E-124	441
	Cotton_A_04522	42.83	509	271	6	24	523	19	516	7.00E-120	427
	Cotton_A_04519	40.2	403	223	6	33	427	157	549	6.00E-88	322
	Cotton_A_00379	51.28	273	117	7	303	565	2	268	3.00E-69	259
	Cotton_A_41171	31.83	553	315	19	24	543	23	546	2.00E-63	240
	Cotton_A_11229	32.08	558	307	21	24	543	31	554	4.00E-61	232
	Cotton_A_05112	30.55	550	309	18	25	543	29	536	1.00E-60	231
	Cotton_A_04523	38.97	290	157	4	24	313	25	294	5.00E-57	219
	Cotton_A_04527	38.62	290	158	4	24	313	25	294	2.00E-55	213
	Cotton_A_04521	38.62	290	156	6	24	312	25	293	4.00E-55	213
	Cotton_A_20741	38.38	284	155	4	28	311	30	293	5.00E-55	212
	Cotton_A_04515	37.72	289	160	4	24	312	25	293	1.00E-53	207
	Cotton_A_04520	45.3	181	99	0	24	204	25	205	1.00E-49	194
	Cotton_A_29170	26.43	541	357	18	36	565	39	549	6.00E-43	172
	Cotton_A_04677	28.57	504	313	16	25	525	32	491	6.00E-41	166
	Cotton_A_03574	25.81	554	350	17	30	565	26	536	4.00E-39	159
	Cotton_A_02040	27.81	525	334	13	45	565	47	530	1.00E-38	158
	Cotton_A_14657	43.32	217	94	6	214	426	3	194	3.00E-38	156
	Cotton_A_25524	28.13	487	312	13	41	525	41	491	7.00E-38	155
	Cotton_A_30797	26.22	492	317	12	45	531	47	497	8.00E-37	152
	Cotton_A_17325	27.94	494	303	14	41	525	41	490	1.00E-36	151
	Cotton_A_28552	27.55	490	314	13	45	531	45	496	2.00E-36	150
	Cotton_A_29171	25	516	358	10	53	565	1	490	3.00E-36	149
	Cotton_A_16611	27.13	505	336	14	29	525	30	510	1.00E-35	147
	Cotton_A_13811	82.05	78	14	0	89	166	1	78	5.00E-35	145
	Cotton_A_13811	77.5	40	9	0	526	565	79	118	8.00E-14	75.9
	Cotton_A_29696	28.08	495	311	14	41	530	41	495	1.00E-34	145
	Cotton_A_33724	26.53	524	342	13	45	565	45	528	8.00E-34	142
	Cotton_A_02508	24.67	527	356	12	45	565	45	536	1.00E-31	134

	Cotton_A_25793	25.87	487	322	11	41	525	29	478	4.00E-31	132
	Cotton_A_21767	26.58	489	316	15	41	525	45	494	1.00E-30	131
	Cotton_A_33725	26.49	487	311	13	45	525	48	493	3.00E-30	130
	Cotton_A_32443	25.8	531	347	17	41	565	43	532	7.00E-30	129
	Cotton_A_02506	22.59	487	345	12	47	531	29	485	4.00E-28	123
	Cotton_A_02505	22.59	487	345	12	47	531	29	485	6.00E-28	122
	Cotton_A_27307	22.59	487	345	12	47	531	31	487	6.00E-28	122
	Cotton_A_16427	24.85	503	329	14	29	525	16	475	3.00E-27	120
	Cotton_A_02502	23	487	343	12	47	531	47	503	5.00E-27	119
	Cotton_A_36377	22.87	481	339	12	47	525	31	481	3.00E-25	113
	Cotton_A_02504	23.2	487	342	12	47	531	47	503	4.00E-25	113
	Cotton_A_31751	24.18	517	349	16	29	530	30	518	1.00E-24	111
	Cotton_A_02507	23	487	343	12	47	531	47	503	2.00E-24	110
	Cotton_A_04525	39.33	150	79	3	164	313	8	145	7.00E-22	102
	Cotton_A_16999	29.22	154	106	3	45	197	47	198	5.00E-12	69.7
**AtLAC13**	Cotton_A_04178	74.51	561	139	4	9	569	20	576	0	807
	Cotton_A_24290	73.8	561	143	4	9	569	20	576	0	785
	Cotton_A_13818	66.73	565	180	7	8	569	9	568	0	706
	Cotton_A_13820	67.59	543	168	7	30	569	1	538	0	684
	Cotton_A_15837	67.03	543	172	6	30	569	1	539	0	682
	Cotton_A_31477	66.11	543	172	7	30	569	1	534	0	658
	Cotton_A_13817	63.89	565	173	8	8	569	9	545	0	655
	Cotton_A_00882	50	570	260	10	7	569	12	563	8.00E-142	500
	Cotton_A_12054	45.98	609	280	10	4	569	12	614	2.00E-141	499
	Cotton_A_19723	50.45	555	253	8	19	569	27	563	8.00E-140	494
	Cotton_A_06597	49.38	565	267	9	8	569	8	556	1.00E-139	493
	Cotton_A_30033	48.04	562	273	7	16	569	17	567	5.00E-139	491
	Cotton_A_17036	50.18	544	249	9	30	569	63	588	1.00E-137	486
	Cotton_A_30645	49.73	551	261	9	26	569	36	577	3.00E-137	485
	Cotton_A_07013	46.97	577	286	10	6	569	10	579	3.00E-137	485
	Cotton_A_00902	47.82	550	274	8	26	569	34	576	3.00E-136	482
	Cotton_A_30034	49.47	570	267	11	10	569	12	570	4.00E-136	482
	Cotton_A_05572	46.93	586	264	9	1	569	1	556	8.00E-136	480
	Cotton_A_00905	47.01	551	279	8	25	569	33	576	6.00E-135	478
	Cotton_A_30643	49.55	551	262	9	26	569	36	577	6.00E-135	478
	Cotton_A_35771	48.91	552	259	10	26	569	28	564	6.00E-135	478
	Cotton_A_25874	46.01	552	276	8	25	569	20	556	2.00E-134	476
	Cotton_A_00947	47.82	550	274	8	26	569	34	576	5.00E-134	474
	Cotton_A_30646	49.36	551	263	9	26	569	36	577	2.00E-133	473
	Cotton_A_20282	45.44	570	287	9	4	569	7	556	5.00E-131	464
	Cotton_A_14417	45.11	552	281	8	25	569	32	568	3.00E-130	462
	Cotton_A_13553	48.1	553	261	11	24	569	47	580	4.00E-130	462
	Cotton_A_12917	47.16	564	281	10	7	569	9	556	5.00E-130	461
	Cotton_A_00335	47.38	572	280	8	2	569	4	558	1.00E-129	460
	Cotton_A_30035	49.53	537	245	10	30	552	1	525	3.00E-128	455
	Cotton_A_22687	47.37	532	264	9	30	552	8	532	7.00E-127	451
	Cotton_A_32213	44.39	570	288	10	7	569	8	555	3.00E-124	442
	Cotton_A_26217	46.07	573	254	10	4	569	11	535	5.00E-124	441
	Cotton_A_20043	49.21	504	239	8	30	527	1	493	2.00E-122	436
	Cotton_A_04513	47.04	523	264	8	53	569	12	527	8.00E-121	431
	Cotton_A_04514	42.71	569	304	8	7	569	11	563	5.00E-119	425
	Cotton_A_04517	39.65	575	326	8	1	569	1	560	1.00E-113	407
	Cotton_A_37880	39.72	574	316	10	9	569	12	568	4.00E-110	395
	Cotton_A_10403	39.02	574	331	9	4	569	3	565	2.00E-109	393
	Cotton_A_04526	40.49	568	309	11	7	569	5	548	2.00E-107	386
	Cotton_A_04522	42.31	520	277	10	18	530	16	519	5.00E-103	372
	Cotton_A_26221	41.11	540	217	12	35	569	6	449	5.00E-96	348
	Cotton_A_04519	37.91	401	228	8	14	408	140	525	2.00E-68	256
	Cotton_A_41171	29.82	560	328	16	19	547	21	546	7.00E-56	215
	Cotton_A_00379	49.8	253	118	5	322	569	20	268	1.00E-54	211
	Cotton_A_04521	40	265	144	6	10	274	14	263	3.00E-52	203
	Cotton_A_04527	40.67	268	142	7	8	275	14	264	4.00E-51	199
	Cotton_A_04523	39.55	268	145	6	8	275	14	264	2.00E-50	197
	Cotton_A_04515	38.72	266	148	6	10	275	14	264	2.00E-49	193
	Cotton_A_20741	38.27	277	152	9	3	275	4	265	2.00E-48	190
	Cotton_A_04520	40.4	198	114	3	8	203	10	205	1.00E-46	184
	Cotton_A_11229	29.93	294	168	8	10	277	20	301	5.00E-30	129
	Cotton_A_11229	47.27	110	55	1	441	547	445	554	1.00E-25	114
	Cotton_A_13811	73.75	80	19	2	86	165	1	78	3.00E-28	123
	Cotton_A_13811	70	40	12	0	530	569	79	118	5.00E-11	66.2
	Cotton_A_17325	32.22	270	164	8	12	276	13	268	1.00E-27	121

	Cotton_A_05112	28.57	301	160	9	8	277	11	287	3.00E-27	120
	Cotton_A_05112	44.55	110	57	2	441	547	428	536	2.00E-23	107
	Cotton_A_29170	28.67	279	184	8	6	275	7	279	4.00E-26	116
	Cotton_A_04677	30.66	274	171	8	8	277	11	269	6.00E-26	115
	Cotton_A_29696	31.23	269	168	7	11	276	14	268	8.00E-26	115
	Cotton_A_25524	30.69	277	171	8	9	277	6	269	1.00E-24	111
	Cotton_A_28552	33.62	235	139	7	45	276	48	268	2.00E-24	110
	Cotton_A_25793	28.57	266	177	5	13	276	2	256	2.00E-23	107
	Cotton_A_02040	32.48	274	166	8	8	277	11	269	4.00E-23	106
	Cotton_A_21767	29.45	275	173	8	7	275	12	271	3.00E-22	103
	Cotton_A_29171	30.4	227	152	6	51	275	2	224	3.00E-21	100
	Cotton_A_30797	28.62	283	173	10	1	272	1	265	8.00E-21	99
	Cotton_A_32443	30.47	233	147	6	45	275	50	269	1.00E-20	98.6
	Cotton_A_16611	29.45	275	184	6	7	275	10	280	1.00E-20	98.2
	Cotton_A_02508	31.22	237	155	7	42	277	45	274	4.00E-20	96.3
	Cotton_A_03574	29.18	257	171	5	25	275	24	275	2.00E-19	94
	Cotton_A_33724	29.2	274	173	7	9	277	10	267	5.00E-19	93.2
	Cotton_A_02504	29.24	236	155	8	45	277	48	274	5.00E-17	86.3
	Cotton_A_02507	29.24	236	155	8	45	277	48	274	5.00E-17	86.3
	Cotton_A_36377	27.35	234	162	6	45	277	32	258	2.00E-16	84.7
	Cotton_A_14657	38.19	199	89	6	213	407	3	171	2.00E-16	84
	Cotton_A_04525	38.94	113	64	2	163	275	8	115	4.00E-16	83.2
	Cotton_A_02506	26.92	234	163	6	45	277	30	256	1.00E-15	81.6
	Cotton_A_02505	26.92	234	163	6	45	277	30	256	1.00E-15	81.3
	Cotton_A_27307	26.92	234	163	6	45	277	32	258	2.00E-15	81.3
	Cotton_A_02502	26.07	234	165	6	45	277	48	274	3.00E-15	80.1
	Cotton_A_33725	28.82	288	166	8	1	272	1	265	6.00E-15	79.3
	Cotton_A_16427	25.6	250	173	5	25	273	15	252	2.00E-14	77.8
	Cotton_A_31751	26.89	264	173	8	25	275	29	285	3.00E-14	77
	Cotton_A_16999	33.85	192	116	6	10	196	13	198	6.00E-14	76.3
**AtLAC14**	Cotton_A_13818	49.82	564	265	6	20	569	9	568	5.00E-155	544
	Cotton_A_24290	52.38	546	246	7	35	569	34	576	6.00E-154	541
	Cotton_A_13820	51.11	542	247	6	42	569	1	538	5.00E-153	538
	Cotton_A_04178	50.71	562	262	8	19	569	19	576	6.00E-153	537
	Cotton_A_15837	51.2	543	246	7	42	569	1	539	6.00E-153	537
	Cotton_A_31477	51.4	535	252	5	42	569	1	534	1.00E-152	536
	Cotton_A_04513	55.41	518	216	5	65	569	12	527	7.00E-152	534
	Cotton_A_13817	48.85	563	246	7	21	569	11	545	3.00E-146	515
	Cotton_A_00882	47.31	558	280	7	18	569	14	563	6.00E-143	504
	Cotton_A_17036	48.97	535	259	7	41	569	62	588	3.00E-141	499
	Cotton_A_07013	45.94	579	289	13	11	569	5	579	4.00E-141	498
	Cotton_A_19723	48.44	545	267	7	31	569	27	563	4.00E-141	498
	Cotton_A_00902	46.91	550	269	10	37	569	33	576	7.00E-141	497
	Cotton_A_12054	43.36	602	290	11	15	569	17	614	2.00E-140	496
	Cotton_A_00905	46.36	550	272	10	37	569	33	576	1.00E-139	493
	Cotton_A_00947	46.62	547	275	11	37	569	33	576	1.00E-138	489
	Cotton_A_04514	45.92	564	289	9	16	569	6	563	1.00E-137	486
	Cotton_A_05572	46.35	561	283	8	19	569	4	556	8.00E-136	481
	Cotton_A_30033	43.19	551	298	6	31	569	20	567	8.00E-136	480
	Cotton_A_30646	45.17	580	293	12	12	569	1	577	9.00E-136	480
	Cotton_A_12917	46.24	558	287	7	17	569	7	556	1.00E-135	479
	Cotton_A_30645	45.82	574	286	13	18	569	7	577	2.00E-135	479
	Cotton_A_30643	45.47	574	288	12	18	569	7	577	2.00E-135	479
	Cotton_A_06597	45.88	558	287	7	19	569	7	556	2.00E-134	476
	Cotton_A_20282	45.78	557	287	9	19	569	9	556	8.00E-134	474
	Cotton_A_10403	42.76	566	301	9	18	569	9	565	4.00E-133	471
	Cotton_A_26217	44.68	555	263	8	21	569	19	535	1.00E-132	470
	Cotton_A_04517	45.18	560	289	9	19	569	10	560	4.00E-132	468
	Cotton_A_00335	45.89	547	281	7	30	569	20	558	4.00E-132	468
	Cotton_A_37880	45.63	561	284	11	22	569	16	568	5.00E-132	468
	Cotton_A_32213	45.34	558	291	8	18	569	6	555	4.00E-130	461
	Cotton_A_04526	45.54	560	277	12	19	569	8	548	2.00E-128	456
	Cotton_A_13553	46	537	275	7	39	569	53	580	2.00E-128	456
	Cotton_A_35771	42.86	546	295	7	35	569	25	564	2.00E-126	449
	Cotton_A_30034	41.99	562	309	7	22	569	12	570	4.00E-126	448
	Cotton_A_30035	44.21	527	276	8	42	552	1	525	1.00E-125	447
	Cotton_A_20043	46.87	495	252	7	42	527	1	493	3.00E-122	435
	Cotton_A_22687	42.72	529	285	8	40	552	6	532	1.00E-121	433
	Cotton_A_04522	46.14	518	261	9	19	527	8	516	3.00E-120	429
	Cotton_A_14417	43.44	541	292	6	38	569	33	568	4.00E-120	428
	Cotton_A_25874	43.43	548	296	6	31	569	14	556	3.00E-119	426

	Cotton_A_26221	41.35	532	225	10	42	569	1	449	6.00E-107	384
	Cotton_A_04519	44.98	418	212	9	41	448	156	565	6.00E-90	328
	Cotton_A_04527	41.33	300	155	5	22	321	14	292	3.00E-57	219
	Cotton_A_04521	38.8	299	159	6	19	317	14	288	5.00E-55	212
	Cotton_A_00379	46.8	250	124	5	328	569	20	268	5.00E-55	212
	Cotton_A_04515	39.66	295	163	5	22	315	14	294	2.00E-54	210
	Cotton_A_04523	39.67	300	160	4	22	321	14	292	2.00E-54	210
	Cotton_A_20741	39.27	303	162	6	19	321	13	293	3.00E-54	209
	Cotton_A_41171	32.14	560	330	18	31	559	21	561	3.00E-52	203
	Cotton_A_05112	29.04	582	309	24	16	547	9	536	6.00E-48	189
	Cotton_A_04520	45.92	196	102	2	19	214	14	205	2.00E-46	183
	Cotton_A_29170	27.03	566	371	16	18	569	12	549	1.00E-33	141
	Cotton_A_04677	26.49	521	332	17	20	529	11	491	4.00E-33	139
	Cotton_A_30797	27.74	584	333	18	19	569	4	531	7.00E-32	135
	Cotton_A_16611	26.39	557	368	16	24	565	16	545	2.00E-30	130
	Cotton_A_17325	26.67	540	311	17	21	534	15	495	2.00E-29	127
	Cotton_A_11229	31.02	303	179	9	11	288	4	301	5.00E-29	125
	Cotton_A_11229	49.57	117	53	2	449	559	453	569	1.00E-23	108
	Cotton_A_25524	26.25	522	342	17	15	529	6	491	9.00E-29	125
	Cotton_A_28552	26.34	505	288	17	57	534	48	495	2.00E-28	124
	Cotton_A_33725	27.22	540	300	17	31	541	25	500	3.00E-28	124
	Cotton_A_21767	27.15	523	323	19	22	529	15	494	6.00E-28	122
	Cotton_A_02040	25.23	543	314	17	18	529	9	490	1.00E-27	121
	Cotton_A_32443	26.95	501	280	18	57	529	50	492	3.00E-27	120
	Cotton_A_13811	60.92	87	28	1	98	184	1	81	3.00E-27	120
	Cotton_A_33724	25.59	547	312	18	25	541	14	495	5.00E-27	119
	Cotton_A_03574	27.27	539	345	16	40	565	27	531	6.00E-27	119
	Cotton_A_02508	24.44	581	362	19	15	569	7	536	4.00E-26	116
	Cotton_A_29696	26.04	503	292	16	57	534	48	495	2.00E-25	114
	Cotton_A_25793	26.92	494	293	15	55	529	34	478	6.00E-25	112
	Cotton_A_14657	37.96	216	108	5	222	433	1	194	7.00E-24	108
	Cotton_A_02502	25.29	510	307	16	55	538	46	507	4.00E-23	106
	Cotton_A_02504	25.22	575	337	22	19	558	9	525	7.00E-23	105
	Cotton_A_04525	40.27	149	76	3	173	321	8	143	9.00E-23	105
	Cotton_A_02507	25.22	575	337	22	19	558	9	525	1.00E-22	105
	Cotton_A_02505	25.24	515	301	18	55	538	28	489	1.00E-22	104
	Cotton_A_27307	25.24	515	301	18	55	538	30	491	2.00E-22	104
	Cotton_A_02506	25.05	515	302	18	55	538	28	489	2.00E-22	104
	Cotton_A_36377	24.71	518	300	19	55	538	30	491	1.00E-21	101
	Cotton_A_31751	24.67	535	351	17	50	562	42	546	1.00E-19	95.1
	Cotton_A_29171	29.36	235	161	4	63	295	2	233	7.00E-19	92.4
	Cotton_A_16427	26.51	249	168	4	55	299	33	270	2.00E-13	73.9
	Cotton_A_16999	27.04	196	136	5	15	207	7	198	5.00E-11	66.2
**AtLAC15**	Cotton_A_04513	57.51	506	202	9	51	548	12	512	3.00E-157	551
	Cotton_A_31477	48.66	524	257	7	28	546	1	517	2.00E-140	496
	Cotton_A_13820	47.63	527	262	9	28	546	1	521	9.00E-137	484
	Cotton_A_13818	46.9	533	269	9	22	546	25	551	1.00E-136	483
	Cotton_A_15837	47.64	529	260	10	28	546	1	522	1.00E-135	480
	Cotton_A_04178	47.36	530	269	9	22	546	35	559	3.00E-135	479
	Cotton_A_24290	47.17	530	270	8	22	546	35	559	2.00E-134	476
	Cotton_A_30033	46.77	541	269	9	23	553	26	557	1.00E-130	463
	Cotton_A_04517	45.59	533	271	9	21	546	23	543	1.00E-129	460
	Cotton_A_13817	44.65	533	258	10	22	546	25	528	1.00E-127	453
	Cotton_A_04514	46.62	532	266	10	21	546	27	546	2.00E-127	452
	Cotton_A_12054	42.53	576	282	12	20	553	36	604	3.00E-126	449
	Cotton_A_10403	46.37	537	277	8	21	553	26	555	6.00E-125	444
	Cotton_A_07013	44.77	545	285	8	20	553	30	569	1.00E-124	443
	Cotton_A_00902	45.02	542	285	8	20	553	30	566	6.00E-124	441
	Cotton_A_00882	45.69	534	272	10	24	553	34	553	7.00E-124	441
	Cotton_A_22687	44.49	535	271	10	28	546	8	532	1.00E-123	440
	Cotton_A_30646	45.42	546	276	13	20	553	32	567	2.00E-123	439
	Cotton_A_19723	45.79	535	272	10	23	553	33	553	3.00E-123	439
	Cotton_A_30645	45.69	545	276	12	20	553	32	567	4.00E-123	438
	Cotton_A_00905	44.1	542	290	8	20	553	30	566	5.00E-122	435
	Cotton_A_37880	45.44	537	273	13	19	546	25	550	7.00E-122	434
	Cotton_A_30035	44.19	534	274	10	28	546	1	525	7.00E-122	434
	Cotton_A_00947	43.73	542	292	8	20	553	30	566	7.00E-122	434
	Cotton_A_30643	44.87	546	279	12	20	553	32	567	8.00E-122	434
	Cotton_A_35771	44.17	532	276	10	24	546	28	547	2.00E-121	433
	Cotton_A_17036	45.8	524	266	10	34	553	69	578	7.00E-121	431
	Cotton_A_06597	43.78	539	286	10	19	553	21	546	4.00E-120	428

	Cotton_A_30034	41.43	531	298	7	24	546	28	553	2.00E-117	420
	Cotton_A_04526	44.36	532	267	11	22	546	22	531	1.00E-116	417
	Cotton_A_05572	43.23	539	289	10	19	553	21	546	2.00E-116	416
	Cotton_A_12917	43.41	539	286	11	19	553	23	546	1.00E-115	414
	Cotton_A_20282	43.23	539	286	11	19	553	24	546	4.00E-115	412
	Cotton_A_04522	45.29	510	260	9	22	524	22	519	1.00E-114	410
	Cotton_A_00335	44.51	537	281	10	21	553	25	548	4.00E-114	408
	Cotton_A_25874	44.63	531	276	8	24	547	21	540	8.00E-114	407
	Cotton_A_32213	43.74	535	282	10	23	553	26	545	2.00E-113	406
	Cotton_A_13553	44.47	524	272	11	34	553	62	570	5.00E-113	405
	Cotton_A_14417	43.5	531	282	8	24	547	33	552	6.00E-112	401
	Cotton_A_20043	44.2	500	266	9	28	521	1	493	3.00E-111	399
	Cotton_A_26217	42.51	534	259	11	24	553	36	525	9.00E-109	390
	Cotton_A_26221	36.79	530	240	9	28	553	1	439	2.00E-93	340
	Cotton_A_04519	41.9	401	219	6	28	422	157	549	4.00E-88	322
	Cotton_A_04523	38.68	287	156	4	22	308	28	294	9.00E-53	204
	Cotton_A_20741	40	285	151	6	22	306	29	293	1.00E-52	204
	Cotton_A_04527	38.68	287	156	5	22	308	28	294	9.00E-52	201
	Cotton_A_41171	31.54	558	324	20	24	550	28	558	2.00E-51	200
	Cotton_A_04521	39.46	261	144	4	22	282	28	274	6.00E-51	198
	Cotton_A_04520	49.16	179	91	0	21	199	27	205	1.00E-50	197
	Cotton_A_04515	37.54	285	158	5	23	307	29	293	7.00E-50	195
	Cotton_A_11229	31.43	544	317	20	37	550	49	566	2.00E-49	194
	Cotton_A_05112	29.2	548	316	18	23	541	32	536	7.00E-49	192
	Cotton_A_00379	45.49	266	128	10	297	553	1	258	2.00E-46	184
	Cotton_A_29170	26.84	503	326	19	36	528	44	514	1.00E-32	138
	Cotton_A_29171	27.83	503	313	18	49	539	2	466	2.00E-32	137
	Cotton_A_03574	25.43	519	334	14	26	531	27	505	6.00E-32	135
	Cotton_A_16611	27.55	519	329	14	24	528	30	515	7.00E-32	135
	Cotton_A_25524	26.49	487	309	16	43	523	48	491	2.00E-29	127
	Cotton_A_17325	27.38	504	322	16	43	543	48	510	4.00E-29	126
	Cotton_A_30797	28.19	518	320	19	22	535	29	498	2.00E-28	124
	Cotton_A_32443	27.05	499	316	18	43	536	50	505	5.00E-28	122
	Cotton_A_04677	27.04	503	291	18	40	523	46	491	6.00E-28	122
	Cotton_A_28552	26.61	496	324	14	43	537	48	504	4.00E-27	119
	Cotton_A_14657	35.62	219	112	7	207	421	1	194	4.00E-27	119
	Cotton_A_21767	24.95	497	329	14	43	536	52	507	9.00E-27	118
	Cotton_A_29696	26.85	499	317	18	43	536	48	503	3.00E-25	113
	Cotton_A_25793	25.05	527	303	16	24	523	17	478	6.00E-24	109
	Cotton_A_31751	24.9	526	337	14	24	528	30	518	1.00E-23	108
	Cotton_A_02040	24.67	531	344	16	23	545	30	512	2.00E-21	100
	Cotton_A_13811	51.95	77	37	0	85	161	2	78	2.00E-20	97.8
	Cotton_A_33725	26.92	520	324	19	24	535	29	500	3.00E-20	97.1
	Cotton_A_02508	24.35	497	324	17	43	529	48	502	4.00E-20	96.7
	Cotton_A_33724	22.92	541	341	17	23	545	28	510	1.00E-19	94.7
	Cotton_A_04525	35.9	156	86	4	153	308	4	145	4.00E-19	93.2
	Cotton_A_02506	22.75	501	340	17	40	532	28	489	5.00E-18	89.7
	Cotton_A_02505	22.36	501	342	17	40	532	28	489	1.00E-17	88.6
	Cotton_A_27307	22.36	501	342	17	40	532	30	491	1.00E-17	88.6
	Cotton_A_02504	24.5	502	330	18	40	532	46	507	1.00E-17	88.6
	Cotton_A_02507	24.5	502	330	18	40	532	46	507	2.00E-17	87.4
	Cotton_A_02502	22.7	511	328	18	40	532	46	507	4.00E-17	86.7
	Cotton_A_36377	23.61	504	332	19	40	532	30	491	2.00E-16	84.3
	Cotton_A_16427	26.83	287	190	8	24	306	16	286	5.00E-16	83.2
**AtLAC16**	Cotton_A_20282	73.77	530	124	5	1	523	35	556	0	788
	Cotton_A_12917	71.7	530	136	5	1	523	34	556	0	783
	Cotton_A_06597	70.92	533	137	6	1	523	32	556	0	767
	Cotton_A_05572	69.36	532	147	5	1	523	32	556	0	751
	Cotton_A_32213	63.58	530	179	4	1	523	33	555	0	707
	Cotton_A_00335	67.29	532	158	5	1	523	34	558	0	706
	Cotton_A_13553	63.21	530	181	4	1	523	58	580	0	701
	Cotton_A_00882	60.38	530	197	4	1	523	40	563	0	677
	Cotton_A_17036	59.43	530	202	4	1	523	65	588	0	667
	Cotton_A_19723	59.81	530	200	4	1	523	40	563	0	659
	Cotton_A_00905	56.22	539	218	7	1	523	40	576	7.00E-177	617
	Cotton_A_07013	55.54	542	220	7	1	523	40	579	7.00E-177	617
	Cotton_A_26217	56.79	523	197	4	1	523	42	535	4.00E-176	614
	Cotton_A_00902	56.11	540	217	8	1	523	40	576	2.00E-175	612
	Cotton_A_00947	55.66	539	221	7	1	523	40	576	5.00E-174	607
	Cotton_A_12054	54.02	572	211	9	1	523	46	614	4.00E-173	604
	Cotton_A_30645	52.31	541	235	8	1	523	42	577	3.00E-164	575

	Cotton_A_30646	52.31	541	235	8	1	523	42	577	1.00E-163	573
	Cotton_A_30643	51.76	541	238	8	1	523	42	577	1.00E-161	566
	Cotton_A_13820	49.26	540	251	7	2	523	4	538	1.00E-154	543
	Cotton_A_13818	49.26	540	251	7	2	523	34	568	1.00E-154	543
	Cotton_A_31477	50.19	538	245	7	2	523	4	534	8.00E-154	540
	Cotton_A_15837	49.72	541	248	7	2	523	4	539	4.00E-152	534
	Cotton_A_24290	49.91	535	245	7	6	523	48	576	1.00E-149	526
	Cotton_A_30033	50.28	535	246	5	5	523	37	567	3.00E-149	525
	Cotton_A_04178	49.72	535	246	7	6	523	48	576	4.00E-149	525
	Cotton_A_26221	51.82	523	176	7	1	523	3	449	4.00E-149	525
	Cotton_A_20043	52.52	497	214	8	1	481	3	493	1.00E-147	520
	Cotton_A_13817	47.78	540	236	8	2	523	34	545	5.00E-144	508
	Cotton_A_14417	46.74	537	257	7	5	523	43	568	2.00E-138	489
	Cotton_A_35771	49.25	532	250	7	6	523	39	564	7.00E-138	487
	Cotton_A_25874	46.08	536	260	7	6	523	32	556	1.00E-137	486
	Cotton_A_30034	47.57	534	262	6	6	523	39	570	2.00E-131	466
	Cotton_A_30035	46.96	526	252	8	3	506	5	525	4.00E-131	465
	Cotton_A_22687	46.01	526	253	8	5	506	14	532	1.00E-127	453
	Cotton_A_04514	42.7	534	287	9	2	523	37	563	2.00E-125	446
	Cotton_A_04513	46.42	517	261	5	22	523	12	527	1.00E-124	443
	Cotton_A_04517	42.83	530	285	7	6	523	37	560	2.00E-124	442
	Cotton_A_37880	42.8	535	288	9	2	523	39	568	3.00E-120	429
	Cotton_A_10403	40.49	536	297	7	3	523	37	565	1.00E-117	420
	Cotton_A_04526	41.01	534	287	9	2	523	31	548	2.00E-117	419
	Cotton_A_04522	41.62	495	271	7	2	484	31	519	2.00E-109	393
	Cotton_A_04519	39.17	411	231	9	2	400	160	563	3.00E-82	303
	Cotton_A_00379	54.44	248	104	3	283	523	23	268	4.00E-77	285
	Cotton_A_41171	31.7	530	302	16	8	501	41	546	4.00E-60	229
	Cotton_A_11229	31.77	532	299	18	8	501	49	554	1.00E-58	224
	Cotton_A_05112	31.44	528	291	19	8	501	46	536	6.00E-54	208
	Cotton_A_20741	43.62	243	131	3	2	244	38	274	1.00E-51	201
	Cotton_A_04523	43.21	243	132	3	5	247	40	276	5.00E-51	199
	Cotton_A_04527	42.8	243	133	3	5	247	40	276	1.00E-50	197
	Cotton_A_04515	42.57	249	136	4	5	253	40	281	2.00E-50	197
	Cotton_A_04521	40.83	240	136	3	5	244	40	273	4.00E-49	192
	Cotton_A_14657	47.66	214	92	3	171	384	1	194	1.00E-47	187
	Cotton_A_14657	34.59	159	67	4	365	523	115	236	4.00E-13	73.2
	Cotton_A_17325	29.75	521	313	17	16	523	50	530	2.00E-43	173
	Cotton_A_21767	29.52	481	290	15	14	483	52	494	6.00E-43	172
	Cotton_A_04520	43.27	171	90	1	2	165	37	207	3.00E-41	166
	Cotton_A_32443	29.61	483	287	16	14	483	50	492	4.00E-41	166
	Cotton_A_29171	27.99	518	330	14	20	523	2	490	1.00E-40	164
	Cotton_A_28552	28.54	522	322	17	14	523	48	530	1.00E-39	161
	Cotton_A_04677	28.63	482	294	14	14	483	48	491	4.00E-39	159
	Cotton_A_29696	28.95	487	295	16	14	488	48	495	2.00E-38	157
	Cotton_A_30797	28.71	526	309	19	13	521	49	525	3.00E-37	153
	Cotton_A_29170	26.93	531	336	15	7	516	44	543	8.00E-37	151
	Cotton_A_25524	27.7	527	321	16	14	523	48	531	7.00E-36	148
	Cotton_A_33725	29.17	528	309	18	11	521	48	527	1.00E-35	148
	Cotton_A_02040	27.86	499	291	17	11	489	47	496	2.00E-35	147
	Cotton_A_25793	29.96	494	285	17	7	483	29	478	8.00E-35	145
	Cotton_A_02508	27.07	543	309	18	14	523	48	536	1.00E-34	144
	Cotton_A_03574	25.9	529	343	14	11	523	41	536	2.00E-33	140
	Cotton_A_02505	26.51	498	302	18	14	489	30	485	7.00E-33	138
	Cotton_A_27307	26.51	498	302	18	14	489	32	487	7.00E-33	138
	Cotton_A_02506	26.51	498	302	18	14	489	30	485	1.00E-32	137
	Cotton_A_33724	28.28	495	290	17	11	487	45	492	2.00E-32	137
	Cotton_A_02502	26.01	496	307	17	14	489	48	503	3.00E-32	137
	Cotton_A_16611	26.54	535	341	18	11	523	46	550	5.00E-32	135
	Cotton_A_36377	25.66	491	315	18	14	489	32	487	2.00E-30	130
	Cotton_A_02504	26.46	495	306	20	14	489	48	503	3.00E-29	127
	Cotton_A_02507	26.46	495	306	20	14	489	48	503	4.00E-29	126
	Cotton_A_31751	25.63	515	312	17	7	488	42	518	6.00E-29	125
	Cotton_A_13811	61.84	76	29	0	50	125	3	78	2.00E-24	110
	Cotton_A_16427	32.81	253	150	7	7	249	28	270	2.00E-23	107
	Cotton_A_04525	37.59	133	77	3	121	253	6	132	1.00E-17	87.8
	Cotton_A_17000	29.51	288	168	12	201	483	1	258	7.00E-13	72.4
**AtLAC17**	Cotton_A_07013	81.93	559	96	3	18	576	25	578	0	927
	Cotton_A_00902	79.96	559	104	4	18	576	25	575	0	916
	Cotton_A_00905	79.25	559	108	4	18	576	25	575	0	914
	Cotton_A_00947	78.89	559	110	3	18	576	25	575	0	899

Cotton_A_30646	73.56	556	136	3	22	576	31	576	0	836
Cotton_A_30645	73.38	556	137	3	22	576	31	576	0	834
Cotton_A_30643	73.51	555	136	3	22	575	31	575	0	833
Cotton_A_12054	65.87	586	162	4	22	576	35	613	0	774
Cotton_A_20043	72.13	506	127	5	31	535	1	493	0	726
Cotton_A_06597	58.7	552	208	5	25	576	24	555	0	635
Cotton_A_05572	58.15	552	211	5	25	576	24	555	0	633
Cotton_A_17036	58.21	548	204	7	30	575	62	586	3.00E-179	625
Cotton_A_00882	56.81	558	218	7	19	575	26	561	6.00E-179	624
Cotton_A_12917	57.4	554	214	7	23	576	24	555	6.00E-178	620
Cotton_A_19723	55.56	558	225	6	19	575	26	561	4.00E-176	614
Cotton_A_20282	56.5	554	218	6	23	576	25	555	1.00E-175	613
Cotton_A_13553	56.1	549	219	6	28	576	53	579	2.00E-175	612
Cotton_A_00335	57.61	552	214	5	25	576	26	557	3.00E-173	605
Cotton_A_32213	53.44	552	235	5	25	576	25	554	1.00E-168	590
Cotton_A_26217	53.57	560	205	8	18	575	27	533	5.00E-162	568
Cotton_A_24290	51.8	556	247	9	26	576	36	575	2.00E-151	532
Cotton_A_13818	50.81	559	253	12	23	575	24	566	8.00E-151	530
Cotton_A_13820	51	551	249	11	31	575	1	536	1.00E-150	530
Cotton_A_04178	50.99	557	240	9	31	576	41	575	2.00E-149	526
Cotton_A_15837	51.27	550	250	10	31	575	1	537	1.00E-147	520
Cotton_A_31477	50	550	252	10	31	575	1	532	2.00E-147	519
Cotton_A_30033	48.84	559	258	9	26	575	26	565	8.00E-145	510
Cotton_A_13817	48.84	559	241	13	23	575	24	543	2.00E-139	492
Cotton_A_26221	47.53	547	185	9	31	575	1	447	6.00E-139	491
Cotton_A_25874	45.66	565	280	9	18	576	12	555	6.00E-136	481
Cotton_A_14417	45.57	564	280	9	19	576	25	567	6.00E-134	474
Cotton_A_35771	46.13	555	275	7	27	576	28	563	6.00E-132	468
Cotton_A_30035	47.22	540	260	7	31	560	1	525	4.00E-129	458
Cotton_A_30034	45.85	554	284	5	27	576	28	569	3.00E-128	456
Cotton_A_22687	46.03	541	267	7	30	560	7	532	1.00E-126	450
Cotton_A_04513	48.58	527	255	8	54	576	12	526	1.00E-125	447
Cotton_A_04514	42.13	553	299	7	26	576	29	562	2.00E-122	436
Cotton_A_37880	39.02	551	317	9	24	572	29	562	6.00E-108	388
Cotton_A_04517	38.38	555	318	8	26	576	25	559	2.00E-107	386
Cotton_A_10403	38.49	556	318	9	25	575	27	563	3.00E-106	382
Cotton_A_04526	37.95	556	311	11	25	576	22	547	1.00E-103	374
Cotton_A_04522	39.77	518	288	9	25	538	22	519	1.00E-100	364
Cotton_A_00379	69.82	275	74	2	302	576	2	267	2.00E-100	363
Cotton_A_04519	37.12	431	246	8	30	456	156	565	5.00E-79	292
Cotton_A_41171	32.67	551	313	19	25	551	26	542	9.00E-56	214
Cotton_A_11229	29.98	557	320	19	25	551	34	550	8.00E-51	198
Cotton_A_05112	28.39	560	312	19	24	551	30	532	5.00E-47	186
Cotton_A_04521	36.62	284	153	7	26	306	29	288	1.00E-45	181
Cotton_A_20741	34.16	281	164	4	26	306	30	289	4.00E-44	176
Cotton_A_04527	35.63	261	153	4	26	286	29	274	1.00E-43	174
Cotton_A_04515	35.5	262	154	4	26	287	29	275	1.00E-43	174
Cotton_A_04523	34.52	281	163	4	26	306	29	288	4.00E-43	172
Cotton_A_14657	45.06	233	89	7	210	441	1	195	2.00E-42	171
Cotton_A_04520	39.33	178	108	0	25	202	28	205	1.00E-39	161
Cotton_A_29171	27.07	532	333	17	52	574	2	487	3.00E-32	136
Cotton_A_16611	27.9	534	335	17	27	551	30	522	8.00E-30	129
Cotton_A_03574	26.36	516	329	15	29	537	25	496	3.00E-27	120
Cotton_A_29170	25.05	543	353	18	39	570	44	543	6.00E-27	119
Cotton_A_17325	27.55	265	173	6	25	285	27	276	3.00E-25	113
Cotton_A_13811	60.26	78	31	0	87	164	1	78	3.00E-25	113
Cotton_A_21767	27.55	265	173	6	25	285	31	280	2.00E-23	107
Cotton_A_32443	26.04	265	177	6	25	285	29	278	5.00E-23	106
Cotton_A_28552	27.72	267	178	6	25	286	27	283	6.00E-23	105
Cotton_A_02508	24.05	553	369	17	27	575	29	534	7.00E-23	105
Cotton_A_30797	25.87	286	190	6	25	307	29	295	8.00E-23	105
Cotton_A_04677	25	252	178	4	25	275	27	268	1.00E-22	104
Cotton_A_29696	26.12	268	183	6	25	290	27	281	1.00E-22	104
Cotton_A_16427	28.68	265	178	5	27	290	16	270	3.00E-22	103
Cotton_A_25524	25.1	247	174	4	25	270	27	263	1.00E-21	102
Cotton_A_02505	24.12	514	316	17	46	543	30	485	1.00E-21	101
Cotton_A_27307	24.12	514	316	17	46	543	32	487	1.00E-21	101
Cotton_A_02506	24.26	507	324	18	46	543	30	485	2.00E-20	97.8
Cotton_A_25793	25.65	269	187	5	25	292	15	271	3.00E-20	97.1
Cotton_A_33724	25.1	522	328	16	26	541	28	492	3.00E-20	97.1
Cotton_A_02502	23.52	506	329	15	46	543	48	503	3.00E-20	97.1
Cotton_A_02504	24.28	519	347	16	46	562	48	522	5.00E-20	96.3

	Cotton_A_02507	24.28	519	347	16	46	562	48	522	7.00E-20	95.9
	Cotton_A_36377	23.53	493	333	15	46	537	32	481	7.00E-19	92.4
	Cotton_A_02040	25.67	261	179	6	26	285	30	276	1.00E-18	92
	Cotton_A_31751	25.48	522	326	19	39	542	42	518	4.00E-18	90.1
	Cotton_A_33725	27.3	282	186	6	27	307	32	295	1.00E-16	85.1
	Cotton_A_04525	34.48	145	82	3	162	306	8	139	2.00E-16	84.7

Supplementary Table S1: Details of BlastP similarity search perform to identify the cotton laccases using Arabidopsis laccase proteins as query. After the validation with conserve domain analysis, protein identified as laccases are highlighted with red text.

Query id	Subject id	% identity	alignment length	mismatches	gap openings	q. start	q. end	s. start	s. end	e-value	bit score
AtLAC01	Gorai.006G171500	50.9	558	265	3	25	581	34	583	2.00E-163	573
	Gorai.013G025500	52.42	557	251	6	28	581	31	576	5.00E-163	571
	Gorai.013G021400	52.42	557	251	6	28	581	31	576	6.00E-163	571
	Gorai.011G101300	51.6	562	247	8	22	581	24	562	1.00E-161	566
	Gorai.009G103200	52.42	557	254	6	28	581	31	579	1.00E-161	566
	Gorai.002G257100	52.51	558	248	6	28	581	29	573	8.00E-161	564
	Gorai.007G376800	49.82	560	268	5	25	581	34	583	1.00E-160	563
	Gorai.007G376600	52.93	563	250	9	23	581	19	570	2.00E-159	559
	Gorai.003G129800	51.7	559	251	8	28	581	33	577	1.00E-158	556
	Gorai.013G036200	51.8	556	247	6	26	581	29	563	1.00E-158	556
	Gorai.003G096600	50.18	558	257	6	24	581	27	563	2.00E-158	556
	Gorai.003G129700	52.06	559	249	8	28	581	33	577	2.00E-158	555
	Gorai.013G263200	50.89	562	256	7	24	581	32	577	5.00E-157	551
	Gorai.012G111900	47.94	557	266	8	27	581	22	556	1.00E-148	523
	Gorai.009G093800	49.2	563	260	9	21	581	18	556	6.00E-148	521
	Gorai.011G279600	49.73	555	253	8	29	581	24	554	7.00E-148	521
	Gorai.007G378200	47.57	555	267	8	29	581	24	556	7.00E-147	517
	Gorai.003G124600	48.85	563	261	10	21	581	19	556	3.00E-144	508
	Gorai.002G261500	47.94	559	265	8	26	581	23	558	1.00E-143	506
	Gorai.010G194300	48.32	565	271	7	23	581	19	568	1.00E-143	506
	Gorai.010G194600	48.32	565	271	7	23	581	19	568	4.00E-143	505
	Gorai.003G150200	50	566	259	13	23	581	28	576	5.00E-143	504
	Gorai.009G321900	48.67	563	263	9	21	581	17	555	2.00E-142	503
	Gorai.004G234200	50.09	557	251	11	33	581	39	576	7.00E-140	494
	Gorai.011G290000	48.93	562	261	12	29	581	25	569	3.00E-139	492
	Gorai.012G110000	46.59	558	270	9	33	581	29	567	1.00E-133	473
	Gorai.008G126500	44.27	567	287	9	22	581	28	572	2.00E-128	456
	Gorai.009G260400	46.31	555	282	6	30	581	32	573	2.00E-128	456
	Gorai.002G148200	46.58	556	269	10	33	581	30	564	3.00E-127	452
	Gorai.007G110500	43.84	568	288	10	22	581	30	574	8.00E-126	447
	Gorai.009G103600	43.67	561	237	7	21	579	25	508	1.00E-121	433
	Gorai.012G109700	44.12	553	278	11	25	564	22	556	3.00E-118	422
	Gorai.012G109900	44.11	560	283	9	33	581	41	581	2.00E-117	420
	Gorai.012G109500	43.2	544	280	10	33	564	30	556	1.00E-116	417
	Gorai.009G261300	40.93	562	307	9	25	581	18	559	9.00E-112	400
	Gorai.010G130100	42.16	555	299	10	31	581	32	568	1.00E-111	400
	Gorai.009G260600	41.31	564	305	10	22	581	12	553	1.00E-110	397
	Gorai.013G027600	48.72	431	198	7	151	581	2	409	2.00E-108	390
	Gorai.007G205400	45.05	495	241	10	84	564	21	498	5.00E-108	389
	Gorai.009G260800	40.35	565	305	11	25	581	20	560	2.00E-107	386
	Gorai.009G261000	40.57	562	309	11	24	581	23	563	6.00E-107	385
	Gorai.009G261100	40	555	308	10	31	581	30	563	1.00E-106	384
	Gorai.009G261500	40.5	563	300	13	25	581	18	551	2.00E-105	380
	Gorai.005G076600	38.2	555	322	7	31	581	29	566	2.00E-105	380
	Gorai.003G129500	45.31	490	203	10	28	512	33	462	9.00E-102	367
	Gorai.013G036000	53.09	307	134	3	26	325	29	332	8.00E-89	325
	Gorai.013G036000	58.86	158	64	1	424	581	300	456	1.00E-45	181
	Gorai.009G323100	42.28	395	190	4	29	419	4	364	2.00E-84	310
	Gorai.010G194200	51.99	277	129	2	35	309	4	278	8.00E-81	298
	Gorai.010G194200	52.03	148	66	2	438	581	267	413	7.00E-32	135
	Gorai.010G194400	42.58	364	179	4	23	386	19	352	1.00E-76	284
	Gorai.008G103600	31.67	581	328	21	22	571	19	561	1.00E-51	201
	Gorai.003G045100	31.3	575	324	21	29	571	39	574	2.00E-50	197
	Gorai.004G227900	29.44	591	324	22	23	571	25	564	7.00E-49	192
	Gorai.012G109600	47.49	179	90	2	40	216	37	213	3.00E-45	180
	Gorai.009G260700	33.91	289	167	6	24	310	23	289	6.00E-42	169
	Gorai.009G261200	35.69	269	155	6	24	290	23	275	8.00E-42	168
	Gorai.009G261600	33.9	292	169	6	24	313	23	292	8.00E-42	168
	Gorai.009G261400	33.56	292	170	6	24	313	23	292	1.00E-40	164
	Gorai.009G287100	33.21	271	163	5	22	290	22	276	6.00E-39	159
	Gorai.009G309400	26.41	549	347	18	43	581	44	545	1.00E-38	158
	Gorai.008G236600	27.03	566	353	22	31	581	30	550	1.00E-34	144
	Gorai.001G048300	24.3	572	357	18	33	581	27	545	8.00E-32	135
	Gorai.001G102100	25	536	348	15	31	556	32	523	9.00E-32	135
	Gorai.006G212500	27.99	568	346	19	31	581	30	551	1.00E-31	134
	Gorai.013G027700	53.33	105	49	0	22	126	25	129	2.00E-31	134
	Gorai.009G179300	25.57	528	329	16	26	545	24	495	2.00E-29	127
	Gorai.008G073000	25.24	527	292	21	47	547	47	497	3.00E-29	127

	Gorai.002G202300	25.19	528	328	18	29	547	31	500	7.00E-29	125
	Gorai.002G173600	25.56	536	325	16	28	547	26	503	7.00E-29	125
	Gorai.002G120000	24.52	522	327	15	29	541	27	490	2.00E-28	124
	Gorai.009G268100	25.34	517	329	18	29	541	27	490	3.00E-28	123
	Gorai.008G189200	24.95	533	311	18	29	541	34	497	4.00E-28	123
	Gorai.009G309500	23.15	553	359	19	43	578	44	547	6.00E-28	122
	Gorai.008G038600	24.53	534	330	18	26	547	26	498	9.00E-28	122
	Gorai.010G181000	24.86	523	329	16	29	543	27	493	3.00E-27	120
	Gorai.007G052900	26.17	535	308	20	31	547	31	496	3.00E-27	120
	Gorai.007G209700	25.58	520	288	18	47	541	48	493	5.00E-27	119
	Gorai.013G245000	24.44	532	330	15	31	547	29	503	1.00E-26	118
	Gorai.013G244900	25.79	535	325	15	28	547	26	503	4.00E-26	116
	Gorai.013G244800	24.44	532	330	15	31	547	29	503	1.00E-25	114
	Gorai.009G189900	27.37	285	187	8	29	310	27	294	3.00E-24	110
	Gorai.007G209900	26.67	510	293	18	47	541	48	491	1.00E-23	108
	Gorai.004G098200	23.93	514	310	15	50	547	39	487	4.00E-23	106
	Gorai.011G104400	24.85	507	301	18	47	541	42	480	7.00E-22	102
	Gorai.009G194300	24.91	281	195	7	31	309	32	298	1.00E-21	102
	Gorai.013G244700	25.71	280	197	5	31	309	29	298	3.00E-20	97.1
**AtLAC02**	Gorai.003G129800	73.54	567	148	2	8	573	12	577	0	866
	Gorai.003G129700	72.77	573	153	3	2	573	7	577	0	860
	Gorai.009G103200	71.63	571	154	3	9	573	11	579	0	849
	Gorai.013G263200	72.13	567	152	6	9	573	15	577	0	840
	Gorai.013G021400	72.55	550	146	3	27	573	29	576	0	836
	Gorai.013G025500	71.45	550	152	3	27	573	29	576	0	823
	Gorai.007G376600	68.17	553	170	3	25	573	20	570	0	811
	Gorai.002G257100	67.67	566	177	3	9	573	13	573	0	808
	Gorai.006G171500	65.61	570	190	3	8	573	16	583	0	781
	Gorai.007G376800	66.85	555	178	3	23	573	31	583	0	772
	Gorai.009G103600	59.71	546	154	4	26	571	29	508	0	638
	Gorai.003G129500	64.66	498	128	6	8	504	12	462	0	636
	Gorai.003G124600	55.85	573	234	8	1	573	3	556	7.00E-180	627
	Gorai.012G111900	55.92	574	234	8	1	573	1	556	4.00E-179	625
	Gorai.009G093800	55.65	575	234	10	1	573	1	556	7.00E-179	624
	Gorai.011G279600	54.1	573	244	7	1	573	1	554	3.00E-178	622
	Gorai.007G378200	55.22	565	236	8	9	573	9	556	7.00E-178	620
	Gorai.003G096600	52.69	577	255	9	1	573	1	563	1.00E-175	613
	Gorai.011G101300	53.62	567	243	9	9	573	14	562	5.00E-174	607
	Gorai.009G321900	52.45	572	253	7	2	573	3	555	8.00E-171	597
	Gorai.002G261500	54.45	573	246	7	1	573	1	558	2.00E-169	592
	Gorai.013G036200	53.36	551	242	8	24	573	27	563	2.00E-168	588
	Gorai.010G194300	51.74	576	249	10	12	573	8	568	2.00E-163	573
	Gorai.010G194600	51.22	576	252	10	12	573	8	568	4.00E-162	568
	Gorai.003G150200	51.47	577	249	10	9	573	19	576	1.00E-159	560
	Gorai.011G290000	51.04	576	253	9	13	573	8	569	2.00E-158	556
	Gorai.004G234200	51.91	549	250	7	31	573	36	576	6.00E-158	554
	Gorai.012G110000	48.56	556	272	7	24	573	20	567	8.00E-148	521
	Gorai.002G148200	49.45	548	262	6	31	573	27	564	9.00E-144	507
	Gorai.012G109900	46.41	571	294	6	11	573	15	581	4.00E-135	478
	Gorai.009G260400	49.73	549	263	8	31	573	32	573	6.00E-135	478
	Gorai.012G109500	46.79	560	277	8	11	556	4	556	2.00E-134	476
	Gorai.008G126500	46.5	557	276	11	25	573	30	572	3.00E-133	472
	Gorai.012G109700	45.44	559	286	7	11	556	4	556	3.00E-133	472
	Gorai.007G110500	44.14	580	296	11	9	573	8	574	3.00E-129	459
	Gorai.009G260600	40.81	571	317	9	5	573	2	553	2.00E-120	429
	Gorai.009G261300	39.76	576	327	9	1	573	1	559	4.00E-119	425
	Gorai.009G261100	41.94	546	303	8	31	573	29	563	1.00E-118	423
	Gorai.013G027600	51.78	421	189	7	154	573	2	409	2.00E-118	422
	Gorai.009G261500	40.14	578	314	13	1	573	1	551	3.00E-118	422
	Gorai.009G261000	40.66	578	317	11	1	573	7	563	5.00E-118	421
	Gorai.009G260800	39.79	578	323	12	1	573	3	560	7.00E-116	414
	Gorai.010G130100	38.26	575	337	9	2	573	9	568	2.00E-112	403
	Gorai.005G076600	38.88	553	316	11	30	573	27	566	3.00E-105	379
	Gorai.007G205400	42.8	514	247	13	69	556	6	498	8.00E-105	378
	Gorai.009G323100	48.85	391	165	7	27	411	1	362	2.00E-101	366
	Gorai.013G036000	59.04	271	108	2	24	294	27	294	4.00E-96	349
	Gorai.013G036000	57.05	156	66	1	419	573	301	456	7.00E-46	182
	Gorai.010G194200	57.04	277	116	2	36	311	4	278	3.00E-92	336
	Gorai.010G194200	55.45	110	48	1	464	573	305	413	2.00E-34	144
	Gorai.010G194400	45.82	371	171	7	12	378	8	352	9.00E-87	318
	Gorai.003G045100	30.43	575	328	19	9	547	17	555	7.00E-55	212

	Gorai.008G103600	30.99	555	318	18	30	552	26	547	2.00E-53	207
	Gorai.012G109600	49.29	211	102	2	11	217	4	213	6.00E-53	205
	Gorai.004G227900	31.3	575	317	20	11	547	11	545	6.00E-53	205
	Gorai.009G260700	37.36	273	156	5	20	292	18	275	9.00E-49	191
	Gorai.009G261200	36.23	276	155	5	20	292	18	275	1.00E-47	187
	Gorai.009G261400	35.62	292	167	5	20	311	18	288	4.00E-47	186
	Gorai.009G287100	36.46	288	162	5	24	311	23	289	2.00E-46	184
	Gorai.009G261600	35.57	298	171	6	20	317	18	294	1.00E-45	181
	Gorai.009G309400	27.76	562	349	18	27	573	26	545	2.00E-41	167
	Gorai.013G027700	57.02	121	50	1	7	127	11	129	4.00E-37	153
	Gorai.008G236600	26.55	565	348	16	32	573	30	550	1.00E-35	148
	Gorai.009G309500	25.55	544	353	15	44	573	44	549	2.00E-35	147
	Gorai.009G268100	26.13	532	346	16	8	538	10	495	4.00E-35	146
	Gorai.001G048300	25.18	564	355	16	34	573	25	545	3.00E-33	140
	Gorai.006G212500	26.55	565	349	16	32	573	30	551	9.00E-33	138
	Gorai.002G120000	26.08	533	351	16	8	538	4	495	5.00E-32	136
	Gorai.010G181000	25.49	506	334	13	30	533	27	491	1.00E-31	134
	Gorai.002G202300	25.15	505	336	13	30	533	31	494	1.00E-31	134
	Gorai.009G179300	25	520	319	15	30	533	27	491	8.00E-31	132
	Gorai.008G038600	25.44	507	332	16	30	533	29	492	6.00E-30	129
	Gorai.008G073000	24.77	533	314	17	30	539	29	497	3.00E-29	127
	Gorai.009G189900	25.84	534	345	18	11	538	7	495	4.00E-29	126
	Gorai.013G245000	24.77	533	365	16	9	539	5	503	2.00E-28	124
	Gorai.013G244800	24.44	532	366	16	10	539	6	503	3.00E-27	120
	Gorai.007G052900	26.02	488	315	14	48	533	47	490	1.00E-26	118
	Gorai.008G189200	25.2	508	332	14	30	533	34	497	2.00E-26	117
	Gorai.001G102100	24.76	517	346	16	32	538	32	515	3.00E-26	117
	Gorai.007G209700	26.48	506	324	14	32	533	32	493	7.00E-26	115
	Gorai.009G194300	28.72	282	185	6	32	307	32	303	7.00E-24	109
	Gorai.002G173600	23.84	537	369	17	6	539	4	503	1.00E-23	108
	Gorai.007G209900	23.98	513	336	14	31	537	31	495	9.00E-23	105
	Gorai.013G244700	23.12	532	341	16	10	539	6	471	2.00E-22	103
	Gorai.013G244900	23.28	537	367	18	9	539	6	503	2.00E-21	100
	Gorai.004G098200	24.75	497	318	16	51	539	39	487	5.00E-20	96.7
	Gorai.003G096200	29.82	171	74	1	24	194	17	141	4.00E-19	93.2
	Gorai.011G104400	25.59	297	200	9	1	296	1	277	2.00E-16	84.7
**AtLAC03**	Gorai.003G150200	77.21	544	121	2	28	570	35	576	0	885
	Gorai.004G234200	74.59	543	137	1	28	570	35	576	0	863
	Gorai.011G290000	68.07	548	171	3	26	570	23	569	0	779
	Gorai.010G194600	66	547	183	3	26	570	23	568	0	763
	Gorai.010G194300	65.81	547	184	3	26	570	23	568	0	762
	Gorai.006G171500	48.63	547	272	4	30	570	40	583	5.00E-164	574
	Gorai.011G101300	51.01	545	248	5	30	570	33	562	9.00E-164	573
	Gorai.007G376800	49.36	547	268	5	30	570	40	583	1.00E-163	573
	Gorai.013G021400	51.18	549	254	9	30	570	34	576	1.00E-162	570
	Gorai.012G110000	51.36	551	248	6	30	570	27	567	1.00E-162	570
	Gorai.013G036200	52.84	545	238	5	30	570	34	563	1.00E-162	570
	Gorai.009G103200	51.27	550	255	8	30	570	34	579	5.00E-162	568
	Gorai.003G096600	51.93	545	243	6	30	570	34	563	4.00E-161	565
	Gorai.012G111900	49.72	545	256	5	30	570	26	556	1.00E-160	563
	Gorai.003G129700	51.82	550	248	9	30	570	36	577	3.00E-160	562
	Gorai.013G263200	51.64	548	249	7	30	570	39	577	3.00E-160	562
	Gorai.013G025500	50.82	549	256	9	30	570	34	576	4.00E-160	561
	Gorai.003G129800	51.55	549	251	8	30	570	36	577	2.00E-159	559
	Gorai.009G093800	50.28	545	251	7	30	570	28	556	5.00E-159	558
	Gorai.002G148200	51.99	552	239	8	30	570	28	564	6.00E-159	557
	Gorai.007G378200	49.72	545	256	5	30	570	26	556	5.00E-158	554
	Gorai.007G376600	50	548	263	7	30	570	27	570	2.00E-157	553
	Gorai.002G257100	51.3	540	248	9	39	570	41	573	9.00E-157	550
	Gorai.003G124600	49.72	545	253	7	30	570	29	556	5.00E-154	541
	Gorai.012G109900	51.36	553	247	8	30	570	39	581	7.00E-154	541
	Gorai.002G261500	50.64	545	251	5	30	570	28	558	1.00E-153	540
	Gorai.009G260400	48.55	550	265	8	30	570	33	573	3.00E-151	532
	Gorai.012G109700	50.83	541	237	8	30	553	28	556	5.00E-151	531
	Gorai.008G126500	47.9	547	268	7	30	570	37	572	3.00E-149	525
	Gorai.007G110500	48.45	547	265	7	30	570	39	574	4.00E-149	525
	Gorai.011G279600	47.16	545	268	6	30	570	26	554	1.00E-148	523
	Gorai.012G109500	49.26	540	247	7	30	553	28	556	2.00E-148	523
	Gorai.009G321900	46.42	545	272	6	30	570	27	555	8.00E-144	507
	Gorai.009G260600	44	550	290	5	26	570	17	553	1.00E-134	477
	Gorai.009G261300	42.47	551	296	7	28	570	22	559	2.00E-132	469

	Gorai.009G323100	60.31	383	126	5	30	410	6	364	1.00E-128	457
	Gorai.009G260800	41.45	550	302	5	28	570	24	560	1.00E-128	456
	Gorai.009G261500	41.85	552	290	9	28	570	22	551	1.00E-128	456
	Gorai.009G261100	41.95	553	294	10	28	570	28	563	2.00E-128	456
	Gorai.005G076600	41.98	555	299	9	26	570	25	566	2.00E-126	449
	Gorai.009G103600	43.38	544	233	7	30	568	35	508	2.00E-126	449
	Gorai.009G261000	41.49	552	298	7	28	570	28	563	2.00E-126	449
	Gorai.010G130100	41.27	550	305	8	28	570	30	568	6.00E-125	444
	Gorai.007G205400	44.69	499	237	8	84	559	22	504	1.00E-119	427
	Gorai.013G027600	50.35	423	191	6	152	570	2	409	1.00E-117	420
	Gorai.010G194200	68.73	275	85	1	34	307	4	278	1.00E-113	407
	Gorai.010G194200	67.65	136	43	1	435	570	279	413	6.00E-54	209
	Gorai.003G129500	47.61	481	189	12	30	501	36	462	7.00E-112	401
	Gorai.010G194400	56.2	347	131	5	26	371	23	349	1.00E-107	387
	Gorai.013G036000	56.65	263	114	0	30	292	34	296	2.00E-87	320
	Gorai.013G036000	57.32	157	67	0	414	570	300	456	1.00E-51	201
	Gorai.003G045100	32.16	538	301	16	43	548	54	559	5.00E-61	232
	Gorai.004G227900	30.5	541	304	19	43	548	46	549	1.00E-60	231
	Gorai.008G103600	32.65	533	305	15	43	548	41	546	2.00E-60	230
	Gorai.012G109600	55.06	178	80	0	30	207	28	205	4.00E-54	209
	Gorai.009G260700	38.35	279	156	5	30	305	30	295	1.00E-50	198
	Gorai.009G287100	36.49	285	162	6	26	310	27	292	3.00E-50	196
	Gorai.009G261400	38.49	278	155	5	28	302	28	292	5.00E-49	192
	Gorai.009G261200	38.08	260	148	3	28	287	28	274	6.00E-49	192
	Gorai.009G261600	36.14	285	163	5	28	312	28	293	2.00E-47	187
	Gorai.009G309400	25.66	534	360	11	42	570	44	545	4.00E-41	166
	Gorai.009G179300	28.46	499	315	15	34	530	33	491	8.00E-41	165
	Gorai.001G048300	28.65	534	337	19	49	570	44	545	9.00E-41	165
	Gorai.010G181000	29.07	492	305	16	42	530	41	491	4.00E-40	163
	Gorai.008G236600	28.57	525	331	17	30	541	30	523	6.00E-39	159
	Gorai.009G309500	26.25	541	362	16	37	570	39	549	7.00E-38	155
	Gorai.009G268100	27.68	495	303	14	49	535	48	495	5.00E-37	152
	Gorai.006G212500	28.44	524	334	17	30	541	30	524	2.00E-36	150
	Gorai.002G120000	28.1	484	305	13	49	530	48	490	1.00E-35	148
	Gorai.001G102100	28.11	523	323	19	34	541	39	523	2.00E-35	147
	Gorai.013G245000	26.16	497	328	15	47	539	46	507	9.00E-35	145
	Gorai.013G244800	26.16	497	328	15	47	539	46	507	1.00E-34	144
	Gorai.002G173600	25.35	493	330	14	47	536	46	503	1.00E-34	144
	Gorai.007G052900	26.94	490	307	16	46	530	47	490	4.00E-34	143
	Gorai.008G073000	26.38	527	338	15	49	570	50	531	4.00E-34	143
	Gorai.008G038600	26.64	488	313	13	46	530	47	492	9.00E-33	138
	Gorai.008G189200	27.88	520	285	17	34	530	45	497	2.00E-32	137
	Gorai.004G098200	26.51	498	314	15	49	539	39	491	6.00E-32	135
	Gorai.011G104400	26.83	492	300	17	46	530	42	480	7.00E-32	135
	Gorai.013G244900	24.95	493	332	13	47	536	46	503	2.00E-31	134
	Gorai.007G209900	27	500	298	17	46	532	48	493	2.00E-31	134
	Gorai.002G202300	26.92	494	304	15	46	530	49	494	5.00E-31	132
	Gorai.013G244700	25.2	496	302	16	47	539	46	475	8.00E-31	132
	Gorai.007G209700	26.69	532	337	15	46	570	48	533	3.00E-30	130
	Gorai.009G189900	31.7	265	162	6	49	310	48	296	3.00E-29	127
	Gorai.013G027700	53.13	96	45	0	30	125	34	129	1.00E-26	118
	Gorai.009G194300	29.77	262	171	6	30	288	32	283	2.00E-23	107
	Gorai.006G269000	25.93	270	169	7	52	304	51	306	1.00E-17	88.2
**AtLAC04**	Gorai.007G378200	80.22	541	106	1	19	558	16	556	0	907
	Gorai.012G111900	79.3	541	111	1	19	558	16	556	0	896
	Gorai.003G124600	78.7	540	113	1	19	558	19	556	0	885
	Gorai.009G093800	76.89	541	122	2	19	558	18	556	0	874
	Gorai.002G261500	75.42	541	132	1	19	558	18	558	0	829
	Gorai.009G321900	65.95	555	186	2	5	558	3	555	0	760
	Gorai.011G279600	64.5	555	194	2	5	558	2	554	0	749
	Gorai.011G101300	62.22	540	200	3	21	558	25	562	0	726
	Gorai.003G096600	61.55	541	204	3	20	558	25	563	0	719
	Gorai.013G036200	61.11	540	206	3	21	558	26	563	0	710
	Gorai.006G171500	59.49	553	210	5	20	558	31	583	0	682
	Gorai.007G376800	59.31	553	211	5	20	558	31	583	0	682
	Gorai.013G021400	58.62	551	215	5	21	558	26	576	0	664
	Gorai.009G103200	57.4	554	220	5	21	558	26	579	0	662
	Gorai.013G025500	58.08	551	218	5	21	558	26	576	0	655
	Gorai.013G263200	56.35	543	228	5	25	558	35	577	0	644
	Gorai.007G376600	55.68	546	228	5	27	558	25	570	0	633
	Gorai.003G129700	55.72	551	232	6	20	558	27	577	5.00E-180	627

Gorai.003G129800	55.17	551	235	6	20	558	27	577	2.00E-179	625
Gorai.002G257100	55.37	549	233	6	22	558	25	573	3.00E-177	618
Gorai.010G194300	50.99	553	251	7	22	558	20	568	2.00E-164	576
Gorai.010G194600	50.81	553	252	7	22	558	20	568	6.00E-164	574
Gorai.004G234200	50.83	543	257	5	26	558	34	576	2.00E-159	559
Gorai.011G290000	50.55	550	251	7	26	558	24	569	5.00E-159	558
Gorai.003G150200	49.91	551	258	6	22	558	30	576	4.00E-157	551
Gorai.009G103600	50.46	543	205	6	19	556	25	508	3.00E-153	538
Gorai.012G110000	49.27	548	266	6	22	558	21	567	1.00E-150	530
Gorai.008G126500	48.18	550	267	6	21	558	29	572	9.00E-149	523
Gorai.007G110500	47.64	550	270	6	21	558	31	574	3.00E-147	519
Gorai.013G027600	59.02	410	164	3	151	558	2	409	2.00E-145	513
Gorai.002G148200	46.86	542	281	4	24	558	23	564	2.00E-140	496
Gorai.009G260400	47.54	549	277	6	21	558	25	573	3.00E-139	492
Gorai.012G109900	46.91	550	278	8	22	558	33	581	7.00E-136	481
Gorai.009G260600	44.13	537	295	5	26	558	18	553	1.00E-135	479
Gorai.009G261100	43.78	539	295	7	26	558	27	563	5.00E-134	474
Gorai.009G261000	43.97	539	294	7	26	558	27	563	8.00E-133	471
Gorai.009G261300	43.15	540	299	6	26	558	21	559	2.00E-132	469
Gorai.012G109500	45.42	535	276	6	23	541	22	556	7.00E-132	468
Gorai.009G261500	43.04	539	293	7	26	558	21	551	2.00E-131	466
Gorai.012G109700	45.71	536	274	7	22	541	22	556	6.00E-131	464
Gorai.009G260800	43.15	540	298	5	26	558	23	560	2.00E-129	459
Gorai.010G130100	41.83	545	300	8	26	558	29	568	6.00E-122	434
Gorai.005G076600	39.53	549	314	8	23	558	23	566	1.00E-121	433
Gorai.007G205400	46.08	510	236	10	66	547	6	504	2.00E-121	433
Gorai.003G129500	48.12	478	198	8	20	489	27	462	1.00E-119	427
Gorai.013G036000	62.27	273	102	1	21	292	26	298	2.00E-106	383
Gorai.013G036000	68.99	158	48	1	402	558	299	456	7.00E-64	241
Gorai.009G323100	43.83	381	184	7	26	397	3	362	9.00E-89	324
Gorai.010G194200	53.51	271	124	2	38	306	9	279	4.00E-84	309
Gorai.010G194200	62.99	127	46	1	432	558	288	413	1.00E-45	181
Gorai.010G194400	42.37	354	173	6	22	367	20	350	9.00E-74	275
Gorai.008G103600	32.35	541	323	15	23	536	22	546	8.00E-62	235
Gorai.003G045100	31.08	547	322	15	23	536	35	559	1.00E-59	227
Gorai.004G227900	31.68	543	320	18	24	536	28	549	1.00E-54	211
Gorai.009G260700	39.05	274	153	4	20	292	21	281	8.00E-52	201
Gorai.009G261200	39.85	266	146	5	22	286	23	275	2.00E-51	200
Gorai.009G268100	28.15	540	358	12	20	558	20	530	8.00E-51	198
Gorai.009G287100	39.62	260	143	4	28	286	30	276	8.00E-50	195
Gorai.009G261400	37.12	264	152	4	22	284	23	273	1.00E-47	187
Gorai.002G120000	27.04	540	364	12	20	558	20	530	1.00E-46	184
Gorai.002G202300	26.77	538	364	12	22	558	26	534	5.00E-46	182
Gorai.012G109600	48.28	174	89	1	38	210	37	210	9.00E-46	181
Gorai.009G261600	36.74	264	153	5	22	284	23	273	2.00E-45	180
Gorai.009G309400	26.88	532	345	15	41	558	44	545	2.00E-44	177
Gorai.009G189900	26.47	544	362	12	20	558	20	530	2.00E-44	177
Gorai.008G038600	26.78	519	346	13	23	538	25	512	5.00E-44	176
Gorai.009G179300	28	550	341	17	23	558	23	531	2.00E-42	170
Gorai.013G027700	65.05	103	36	0	21	123	26	128	1.00E-40	164
Gorai.010G181000	26.9	539	361	13	23	558	23	531	1.00E-40	164
Gorai.008G073000	26.07	514	333	12	20	524	22	497	3.00E-40	163
Gorai.008G189200	26.73	550	347	13	23	558	30	537	4.00E-39	159
Gorai.009G309500	25.38	532	357	12	41	558	44	549	5.00E-39	159
Gorai.007G052900	26.88	532	333	16	22	540	24	512	5.00E-39	159
Gorai.007G209900	27.24	514	318	15	22	522	25	495	6.00E-36	149
Gorai.007G209700	28.06	499	313	13	29	518	32	493	2.00E-35	147
Gorai.011G104400	26.69	502	323	14	22	518	19	480	4.00E-35	146
Gorai.001G048300	25.19	524	357	14	48	558	44	545	6.00E-35	145
Gorai.008G236600	26.08	556	350	20	29	558	30	550	9.00E-35	145
Gorai.006G212500	25.99	554	354	18	29	558	30	551	2.00E-33	141
Gorai.001G102100	24.56	513	340	14	29	523	32	515	5.00E-31	132
Gorai.013G245000	25.64	511	329	16	29	524	29	503	2.00E-29	127
Gorai.002G173600	24.9	506	339	16	29	524	29	503	1.00E-28	124
Gorai.013G244800	25.44	511	330	16	29	524	29	503	4.00E-28	123
Gorai.013G244900	24.81	528	357	16	29	548	29	524	3.00E-27	120
Gorai.003G096200	36.42	173	63	2	20	191	15	141	5.00E-26	116
Gorai.004G098200	25.93	486	314	13	48	524	39	487	3.00E-25	114
Gorai.013G244700	23.35	501	321	13	29	524	29	471	6.00E-24	109
Gorai.009G194300	30.42	263	170	7	29	287	32	285	1.00E-23	108
Gorai.006G269000	24.18	273	173	9	51	304	51	308	7.00E-13	72.4

**AtLAC05**	Gorai.010G194300	79.12	570	111	2	11	580	7	568	0	951
	Gorai.010G194600	79.44	569	109	2	12	580	8	568	0	951
	Gorai.011G290000	77.14	573	122	3	10	580	4	569	0	924
	Gorai.003G150200	66.26	575	183	5	6	580	13	576	0	781
	Gorai.004G234200	67.03	552	172	4	29	580	35	576	0	775
	Gorai.012G110000	51.79	558	246	4	30	580	26	567	2.00E-173	605
	Gorai.006G171500	49.32	592	269	11	2	580	10	583	3.00E-168	588
	Gorai.002G148200	52.42	557	238	6	31	580	28	564	4.00E-167	585
	Gorai.003G096600	49.74	577	262	7	8	580	11	563	5.00E-166	581
	Gorai.007G376800	49.23	583	273	10	8	580	14	583	8.00E-166	580
	Gorai.003G129700	50.25	593	261	11	4	580	3	577	2.00E-165	579
	Gorai.013G021400	50.9	558	253	9	30	580	33	576	2.00E-165	579
	Gorai.003G129800	50.59	593	259	12	4	580	3	577	3.00E-165	578
	Gorai.012G109900	50.86	582	262	7	8	580	15	581	2.00E-164	576
	Gorai.011G101300	48.63	582	268	8	3	580	8	562	1.00E-163	573
	Gorai.013G263200	50.63	557	252	8	30	580	38	577	4.00E-163	572
	Gorai.013G025500	50.62	561	250	11	30	580	33	576	6.00E-163	571
	Gorai.009G103200	49.05	581	277	10	7	580	11	579	9.00E-163	570
	Gorai.013G036200	50.9	554	244	6	31	580	34	563	4.00E-161	565
	Gorai.012G111900	49.73	555	252	6	30	580	25	556	1.00E-160	563
	Gorai.002G257100	49.91	581	264	11	8	580	12	573	1.00E-160	563
	Gorai.007G378200	48.53	579	269	7	6	580	3	556	3.00E-160	561
	Gorai.012G109500	48.95	570	260	8	8	563	4	556	2.00E-159	559
	Gorai.012G109700	49.3	570	258	8	8	563	4	556	7.00E-159	557
	Gorai.007G376600	47.87	587	282	11	1	580	1	570	2.00E-158	555
	Gorai.009G093800	47.66	577	272	8	8	580	6	556	3.00E-158	555
	Gorai.003G124600	48.08	574	267	8	11	580	10	556	1.00E-157	553
	Gorai.009G323100	68.29	391	94	2	29	419	4	364	2.00E-155	546
	Gorai.008G126500	46.77	588	289	6	1	580	1	572	4.00E-155	545
	Gorai.009G260400	46.8	579	287	5	6	580	12	573	1.00E-154	543
	Gorai.007G110500	46.76	586	286	6	4	580	6	574	5.00E-154	541
	Gorai.011G279600	47.5	581	271	9	7	580	1	554	7.00E-154	541
	Gorai.002G261500	47.93	580	274	7	5	580	3	558	9.00E-151	530
	Gorai.009G321900	44.73	579	287	7	10	580	2	555	2.00E-145	513
	Gorai.010G194400	68.72	374	88	4	10	383	9	353	9.00E-144	507
	Gorai.010G194200	80.94	278	53	0	35	312	4	281	2.00E-136	483
	Gorai.010G194200	84.56	136	20	1	445	580	279	413	9.00E-66	248
	Gorai.009G260600	41.81	574	302	10	14	580	5	553	6.00E-130	461
	Gorai.009G103600	42.58	573	244	9	11	578	16	508	3.00E-128	455
	Gorai.009G261100	41.35	561	299	9	27	580	26	563	9.00E-128	454
	Gorai.009G260800	40	575	316	8	11	580	10	560	3.00E-127	452
	Gorai.009G261500	39.55	574	314	9	13	580	5	551	6.00E-126	447
	Gorai.009G261300	39.83	575	319	9	13	580	5	559	1.00E-125	447
	Gorai.007G205400	46.42	517	242	10	68	568	6	503	3.00E-125	445
	Gorai.009G261000	39.66	590	319	11	1	580	1	563	1.00E-124	444
	Gorai.005G076600	38.67	587	332	11	1	580	1	566	1.00E-123	440
	Gorai.013G027600	50	432	188	7	153	580	2	409	4.00E-119	425
	Gorai.010G130100	39.79	583	312	13	8	580	15	568	1.00E-118	423
	Gorai.003G129500	45.96	520	201	14	8	511	7	462	4.00E-115	412
	Gorai.013G036000	52.09	263	126	0	31	293	34	296	6.00E-85	311
	Gorai.013G036000	58.23	158	65	1	423	580	300	456	3.00E-51	200
	Gorai.008G103600	31.2	548	302	19	44	558	41	546	8.00E-59	225
	Gorai.004G227900	28.55	585	335	20	11	558	11	549	2.00E-58	224
	Gorai.003G045100	31.23	586	325	21	7	558	18	559	3.00E-58	223
	Gorai.012G109600	51.74	201	96	1	8	207	4	204	6.00E-57	218
	Gorai.009G261200	37.15	288	167	5	1	288	1	274	1.00E-52	204
	Gorai.009G260700	35.28	309	184	5	1	307	1	295	2.00E-51	200
	Gorai.009G287100	35.44	285	164	4	25	309	25	289	1.00E-50	197
	Gorai.009G261400	36.6	306	178	5	1	304	1	292	7.00E-50	195
	Gorai.009G261600	35.05	311	181	6	1	311	1	290	1.00E-48	191
	Gorai.009G309500	24.7	579	392	14	8	580	9	549	1.00E-35	148
	Gorai.010G181000	26.52	543	344	17	1	540	1	491	2.00E-34	144
	Gorai.009G268100	25.17	576	367	14	13	580	11	530	3.00E-33	140
	Gorai.008G073000	25.56	583	368	17	1	577	7	529	2.00E-32	137
	Gorai.009G179300	25.46	542	349	14	3	540	1	491	3.00E-32	137
	Gorai.001G048300	23.23	564	374	17	32	580	26	545	8.00E-31	132
	Gorai.002G120000	24.48	580	374	14	7	580	9	530	9.00E-31	132
	Gorai.007G052900	26.1	502	311	14	50	546	50	496	2.00E-30	130
	Gorai.009G189900	26.57	508	315	14	43	545	41	495	5.00E-30	129
	Gorai.008G189200	23.88	536	353	13	9	540	13	497	8.00E-30	129
	Gorai.013G027700	48.74	119	58	1	7	125	13	128	9.00E-30	128
	Gorai.008G236600	24.6	565	374	16	1	555	1	523	3.00E-29	127

	Gorai.009G309400	24.03	541	369	13	43	580	44	545	3.00E-29	127
	Gorai.006G212500	25.13	577	369	21	1	561	1	530	3.00E-29	126
	Gorai.007G209900	24.49	543	350	14	7	544	8	495	3.00E-28	123
	Gorai.011G104400	24.77	533	340	17	11	540	6	480	4.00E-27	119
	Gorai.001G102100	24.91	558	332	18	31	561	32	529	2.00E-26	117
	Gorai.008G038600	24.9	502	321	16	43	540	43	492	1.00E-25	114
	Gorai.002G202300	23.65	499	331	12	43	540	45	494	2.00E-25	114
	Gorai.013G245000	23.56	505	336	14	50	549	48	507	3.00E-25	113
	Gorai.009G194300	23.96	551	340	19	3	540	7	491	8.00E-25	112
	Gorai.013G244800	23.37	505	337	14	50	549	48	507	7.00E-24	109
	Gorai.002G173600	22.2	536	366	13	50	580	48	537	1.00E-23	108
	Gorai.007G209700	24.39	537	346	13	50	580	51	533	6.00E-22	102
	Gorai.004G098200	23.37	505	330	17	50	549	39	491	5.00E-21	99.8
	Gorai.013G244900	20.35	575	413	11	7	580	7	537	1.00E-20	98.6
	Gorai.013G244700	25.76	264	185	5	50	312	48	301	2.00E-17	87.8
	Gorai.006G269000	23.53	255	162	9	53	289	51	290	1.00E-13	75.1
**AtLAC06**	Gorai.007G110500	70.48	542	160	0	28	569	33	574	0	837
	Gorai.008G126500	69.56	542	165	0	28	569	31	572	0	827
	Gorai.007G205400	52.64	492	193	5	88	553	22	499	2.00E-153	539
	Gorai.006G171500	44.86	555	282	9	31	569	37	583	2.00E-152	536
	Gorai.007G376800	44.15	564	291	8	22	569	28	583	7.00E-152	534
	Gorai.013G263200	44.71	577	302	6	3	569	8	577	9.00E-150	527
	Gorai.011G290000	46.1	551	274	5	34	569	27	569	4.00E-149	525
	Gorai.010G194600	45.64	550	287	5	29	569	22	568	9.00E-149	524
	Gorai.010G194300	45.45	550	288	5	29	569	22	568	3.00E-148	522
	Gorai.012G111900	45.72	549	281	5	27	569	19	556	5.00E-148	521
	Gorai.013G021400	44.86	555	281	9	31	569	31	576	6.00E-148	521
	Gorai.004G234200	45.97	546	281	6	33	569	36	576	7.00E-148	521
	Gorai.003G150200	47.37	551	274	8	29	569	32	576	9.00E-148	520
	Gorai.009G103200	44.56	561	277	10	31	569	31	579	1.00E-147	520
	Gorai.012G110000	45.54	549	288	5	29	569	22	567	1.00E-147	520
	Gorai.007G378200	44.63	549	287	5	27	569	19	556	6.00E-147	518
	Gorai.011G101300	46.08	549	274	10	29	569	28	562	1.00E-146	516
	Gorai.003G129800	46.03	554	275	11	31	569	33	577	1.00E-146	516
	Gorai.003G096600	46.45	549	272	10	29	569	29	563	5.00E-146	514
	Gorai.013G025500	44.14	555	285	9	31	569	31	576	5.00E-146	514
	Gorai.013G036200	46.35	548	274	9	29	569	29	563	7.00E-146	514
	Gorai.002G261500	46.45	549	277	5	27	569	21	558	2.00E-145	513
	Gorai.003G129700	45.75	553	278	9	31	569	33	577	1.00E-144	510
	Gorai.002G257100	44.23	563	292	9	21	569	19	573	2.00E-144	509
	Gorai.009G093800	45.59	544	277	6	32	569	26	556	5.00E-144	508
	Gorai.011G279600	44.85	544	281	6	32	569	24	554	5.00E-142	501
	Gorai.007G376600	44.36	559	285	10	28	569	21	570	2.00E-141	499
	Gorai.003G124600	44.63	549	284	7	27	569	22	556	3.00E-140	496
	Gorai.002G148200	44.57	543	288	5	34	569	28	564	2.00E-135	479
	Gorai.009G321900	43.17	549	293	6	27	569	20	555	4.00E-135	478
	Gorai.009G260400	44.26	549	285	6	34	569	33	573	7.00E-133	471
	Gorai.012G109900	43.94	553	297	6	27	569	32	581	6.00E-131	464
	Gorai.012G109500	43.04	539	291	6	27	552	21	556	1.00E-129	460
	Gorai.012G109700	43.04	539	291	6	27	552	21	556	3.00E-128	456
	Gorai.009G260600	41.23	553	298	9	29	569	16	553	9.00E-128	454
	Gorai.009G261300	41.71	549	294	12	34	569	24	559	2.00E-126	449
	Gorai.009G261500	40.65	551	299	10	29	569	19	551	1.00E-122	437
	Gorai.009G260800	38.77	552	315	8	29	569	21	560	3.00E-121	432
	Gorai.009G261000	39.6	553	308	10	29	569	25	563	6.00E-119	424
	Gorai.005G076600	39.43	558	316	8	24	569	19	566	7.00E-119	424
	Gorai.009G261100	38.73	550	317	8	29	569	25	563	7.00E-118	421
	Gorai.009G103600	38.72	545	266	8	27	567	28	508	4.00E-115	412
	Gorai.010G130100	39.17	554	312	11	29	569	27	568	9.00E-114	407
	Gorai.013G027600	43.6	422	216	10	156	569	2	409	2.00E-97	353
	Gorai.003G129500	41.15	486	220	12	31	507	33	461	4.00E-97	352
	Gorai.009G323100	42.82	376	197	3	32	406	4	362	2.00E-84	310
	Gorai.013G036000	51.87	268	129	0	29	296	29	296	8.00E-83	305
	Gorai.013G036000	46.3	162	82	3	410	569	298	456	5.00E-41	166
	Gorai.010G194200	46.79	280	149	0	37	316	3	282	4.00E-77	286
	Gorai.010G194200	48.1	158	69	3	424	569	257	413	7.00E-39	159
	Gorai.010G194400	39.48	347	185	4	29	369	22	349	2.00E-72	270
	Gorai.008G103600	30	560	334	16	27	553	21	555	1.00E-60	231
	Gorai.004G227900	28.93	553	332	18	27	547	26	549	8.00E-58	221
	Gorai.012G109600	45.79	190	103	0	27	216	21	210	4.00E-50	196
	Gorai.009G261400	37.98	258	147	3	29	286	25	269	5.00E-49	192

	Gorai.009G260700	36.19	268	158	3	29	296	25	279	8.00E-49	191
	Gorai.009G261600	37.21	258	149	3	29	286	25	269	4.00E-47	186
	Gorai.009G261200	35.61	264	157	3	29	292	25	275	6.00E-47	185
	Gorai.009G287100	32.96	267	166	3	32	298	29	282	5.00E-43	172
	Gorai.009G309500	25.28	534	361	13	46	569	44	549	1.00E-40	164
	Gorai.009G268100	26.06	545	342	16	32	562	27	524	2.00E-39	160
	Gorai.008G236600	27.57	515	332	14	33	534	29	515	3.00E-39	160
	Gorai.001G048300	26.04	553	362	13	33	569	24	545	5.00E-39	159
	Gorai.009G179300	25.9	502	331	11	32	529	27	491	9.00E-39	158
	Gorai.008G073000	26.47	544	353	13	32	569	29	531	7.00E-38	155
	Gorai.002G120000	26.25	522	329	11	32	542	27	503	9.00E-38	155
	Gorai.009G309400	25.66	565	351	20	24	567	21	537	2.00E-37	154
	Gorai.009G189900	25.76	524	329	14	32	542	27	503	1.00E-36	151
	Gorai.001G102100	28.29	516	325	17	33	534	31	515	1.00E-36	151
	Gorai.006G212500	26.89	517	334	14	33	534	29	516	8.00E-36	148
	Gorai.008G189200	25.14	521	336	12	32	542	34	510	2.00E-35	147
	Gorai.010G181000	25.1	506	330	12	32	529	27	491	4.00E-35	146
	Gorai.002G202300	24.07	540	318	13	32	542	31	507	7.00E-33	139
	Gorai.008G038600	24.33	522	339	15	32	542	29	505	1.00E-32	138
	Gorai.003G045100	30.9	288	169	8	21	285	28	308	1.00E-32	138
	Gorai.003G045100	37.08	178	101	4	384	553	394	568	5.00E-24	109
	Gorai.007G052900	25.24	523	329	14	21	529	16	490	9.00E-31	132
	Gorai.011G104400	24.47	519	337	14	21	531	11	482	3.00E-30	130
	Gorai.002G173600	25	508	342	13	28	529	23	497	5.00E-30	129
	Gorai.013G245000	25.15	517	349	15	28	538	23	507	3.00E-29	126
	Gorai.013G027700	55	100	45	0	29	128	29	128	4.00E-29	126
	Gorai.007G209900	24.9	510	321	15	33	529	31	491	3.00E-28	123
	Gorai.013G244800	25.1	518	348	15	28	538	23	507	4.00E-28	123
	Gorai.007G209700	27.86	524	309	20	33	541	31	500	5.00E-28	122
	Gorai.009G194300	25.24	511	338	16	28	534	26	496	7.00E-27	119
	Gorai.013G244900	24.36	505	349	13	28	529	23	497	3.00E-26	117
	Gorai.004G098200	24.59	484	317	14	53	529	39	481	3.00E-23	107
	Gorai.013G244700	26.67	255	181	4	28	281	23	272	4.00E-20	96.7
	Gorai.006G269000	27.1	262	165	7	56	306	51	297	4.00E-16	83.6
**AtLAC07**	Gorai.002G148200	65.28	527	176	4	26	550	26	547	0	724
	Gorai.012G110000	62.83	530	188	3	26	550	25	550	0	721
	Gorai.012G109700	60.82	536	194	6	26	550	26	556	0	672
	Gorai.012G109900	61.77	531	194	4	26	550	37	564	0	667
	Gorai.012G109500	60.07	536	198	6	26	550	26	556	0	666
	Gorai.010G194300	50.65	535	244	7	27	550	26	551	8.00E-158	553
	Gorai.011G290000	50.75	536	243	8	27	550	26	552	5.00E-157	551
	Gorai.010G194600	50.28	535	246	7	27	550	26	551	2.00E-156	549
	Gorai.003G129800	50.19	534	246	7	28	550	36	560	3.00E-150	528
	Gorai.006G171500	48.88	534	255	7	28	550	40	566	4.00E-150	528
	Gorai.003G096600	50.1	525	250	7	27	550	33	546	4.00E-150	528
	Gorai.003G150200	49.53	533	249	8	28	550	37	559	3.00E-149	525
	Gorai.013G263200	49.72	531	250	7	28	550	39	560	8.00E-149	524
	Gorai.007G376800	48.78	533	257	5	28	550	40	566	2.00E-148	522
	Gorai.003G129700	49.34	531	255	5	28	550	36	560	2.00E-148	522
	Gorai.004G234200	48.78	533	253	8	28	550	37	559	7.00E-147	517
	Gorai.011G101300	48.66	524	259	6	27	550	32	545	1.00E-145	513
	Gorai.013G021400	48.5	532	259	5	28	550	34	559	8.00E-145	511
	Gorai.007G376600	47.75	534	261	6	28	550	27	553	2.00E-144	509
	Gorai.002G257100	48.68	532	257	7	28	550	32	556	7.00E-143	504
	Gorai.013G025500	47.57	534	261	6	28	550	34	559	7.00E-143	504
	Gorai.009G103200	46.73	535	267	5	28	550	34	562	9.00E-143	504
	Gorai.013G036200	47.52	524	265	5	27	550	33	546	2.00E-141	499
	Gorai.009G260400	45.85	530	276	5	27	550	32	556	2.00E-139	492
	Gorai.009G093800	47.61	523	263	6	28	550	28	539	5.00E-138	488
	Gorai.003G124600	47.04	523	265	6	28	550	29	539	3.00E-136	482
	Gorai.012G111900	47.16	528	260	7	28	550	26	539	5.00E-135	478
	Gorai.011G279600	46.83	521	266	5	30	550	28	537	8.00E-135	477
	Gorai.007G378200	46.59	528	263	7	28	550	26	539	1.00E-134	477
	Gorai.009G321900	45.51	523	274	5	28	550	27	538	5.00E-131	465
	Gorai.008G126500	43.53	533	277	9	28	550	37	555	2.00E-128	456
	Gorai.007G110500	43.5	531	280	8	28	550	39	557	6.00E-127	451
	Gorai.002G261500	45.75	529	268	7	27	550	27	541	4.00E-126	448
	Gorai.009G103600	43.77	530	220	6	28	550	35	493	9.00E-120	427
	Gorai.009G260600	42.42	528	287	11	28	550	21	536	2.00E-117	420
	Gorai.009G261300	41.54	532	291	11	27	550	23	542	7.00E-113	404
	Gorai.009G261500	40.15	528	294	11	28	550	24	534	9.00E-109	391

	Gorai.009G260800	38.78	526	311	7	28	550	26	543	2.00E-108	390
	Gorai.009G261100	38.9	527	308	10	28	550	30	546	3.00E-108	389
	Gorai.007G205400	43	493	243	11	81	550	21	498	4.00E-108	389
	Gorai.013G027600	50	402	189	7	150	550	2	392	1.00E-107	387
	Gorai.009G261000	39.28	527	306	8	28	550	30	546	2.00E-106	383
	Gorai.005G076600	38.65	533	305	10	28	550	29	549	3.00E-106	382
	Gorai.003G129500	44.01	484	201	10	28	498	36	462	1.00E-102	370
	Gorai.010G130100	38.87	530	306	11	28	550	32	550	1.00E-101	367
	Gorai.009G323100	45.55	382	175	5	27	400	5	361	2.00E-92	336
	Gorai.010G194200	55.23	277	123	1	31	307	3	278	4.00E-91	332
	Gorai.010G194200	45.75	153	64	3	415	550	246	396	3.00E-35	146
	Gorai.013G036000	54.51	266	120	1	27	292	33	297	4.00E-85	312
	Gorai.013G036000	51.37	146	67	1	405	550	298	439	2.00E-40	164
	Gorai.010G194400	44.16	351	169	6	27	374	26	352	3.00E-77	286
	Gorai.012G109600	63.04	184	68	0	26	209	26	209	2.00E-72	270
	Gorai.008G103600	32.23	543	321	18	27	545	27	546	5.00E-62	236
	Gorai.004G227900	30.04	536	314	16	41	546	46	550	3.00E-56	216
	Gorai.003G045100	30.9	534	312	18	41	545	54	559	5.00E-56	215
	Gorai.009G261400	37.64	263	150	4	28	290	30	278	3.00E-51	199
	Gorai.009G261200	36.92	260	150	4	28	287	30	275	1.00E-50	198
	Gorai.009G260700	35.36	280	160	5	28	307	30	288	2.00E-50	197
	Gorai.009G287100	38.57	280	151	7	28	307	31	289	4.00E-50	196
	Gorai.009G261600	37.6	258	147	4	28	285	30	273	6.00E-50	195
	Gorai.009G309500	28.32	505	318	19	34	527	38	509	8.00E-39	158
	Gorai.001G048300	26.81	511	329	15	30	527	27	505	3.00E-35	147
	Gorai.009G309400	26.59	519	333	17	40	547	44	525	2.00E-34	144
	Gorai.009G268100	27.09	502	324	12	28	527	29	490	8.00E-34	142
	Gorai.009G179300	27.38	504	319	17	29	527	30	491	2.00E-33	140
	Gorai.008G236600	27.57	515	328	16	26	527	28	510	3.00E-32	137
	Gorai.002G120000	27.09	491	314	14	40	527	41	490	4.00E-32	136
	Gorai.008G073000	26.65	514	322	16	28	533	31	497	6.00E-32	135
	Gorai.007G209700	28.46	506	312	15	28	527	32	493	2.00E-31	134
	Gorai.006G212500	27.57	515	329	16	26	527	28	511	3.00E-31	133
	Gorai.009G189900	25.87	491	320	13	40	527	41	490	4.00E-31	133
	Gorai.010G181000	26.39	504	324	15	29	527	30	491	5.00E-31	132
	Gorai.002G202300	25.55	501	329	13	30	527	35	494	7.00E-31	132
	Gorai.001G102100	25.39	516	336	14	26	527	30	510	6.00E-30	129
	Gorai.008G189200	25.5	502	332	11	28	527	36	497	7.00E-30	129
	Gorai.008G038600	24.29	490	329	11	40	527	43	492	1.00E-29	128
	Gorai.002G173600	25.56	493	322	17	48	533	49	503	5.00E-28	123
	Gorai.013G245000	24.69	490	330	17	48	533	49	503	1.00E-26	118
	Gorai.009G194300	27.14	490	308	16	42	527	47	491	2.00E-26	117
	Gorai.007G052900	25.44	507	322	14	29	527	32	490	3.00E-26	117
	Gorai.013G244800	24.69	490	330	17	48	533	49	503	7.00E-26	115
	Gorai.007G209900	24.65	507	328	14	28	527	32	491	3.00E-25	113
	Gorai.013G027700	47.42	97	51	0	27	123	33	129	7.00E-24	108
	Gorai.011G104400	24.31	506	326	13	28	527	26	480	2.00E-23	107
	Gorai.004G098200	25.71	490	318	16	48	533	40	487	3.00E-23	107
	Gorai.013G244900	24.54	489	332	16	48	533	49	503	3.00E-23	107
	Gorai.013G244700	23.06	490	306	17	48	533	49	471	8.00E-22	102
	Gorai.003G096200	32.47	154	58	1	37	190	34	141	3.00E-19	94
	Gorai.006G269000	28.51	249	162	6	46	285	47	288	9.00E-19	92.4
	Gorai.004G016000	24.19	401	266	15	137	533	8	374	2.00E-14	78.2
**AtLAC08**	Gorai.002G148200	58.19	531	210	6	25	549	23	547	0	647
	Gorai.012G109500	54.38	548	235	6	14	549	12	556	0	645
	Gorai.012G109700	56.11	565	233	6	14	566	12	573	0	644
	Gorai.012G110000	55.78	536	229	3	20	549	17	550	0	644
	Gorai.012G109900	55.05	545	233	6	14	549	23	564	0	632
	Gorai.010G194300	46.93	554	268	9	12	549	8	551	8.00E-147	517
	Gorai.010G194600	46.03	554	273	8	12	549	8	551	2.00E-145	513
	Gorai.003G150200	47.19	551	266	8	13	549	20	559	4.00E-142	501
	Gorai.011G290000	45.03	553	278	7	13	549	10	552	5.00E-141	498
	Gorai.004G234200	47.4	557	268	8	7	549	14	559	6.00E-140	494
	Gorai.003G096600	44.4	545	282	7	12	549	16	546	8.00E-140	494
	Gorai.013G036200	46.72	533	265	6	23	549	27	546	7.00E-139	491
	Gorai.011G101300	45.97	546	272	9	13	549	14	545	2.00E-138	489
	Gorai.006G171500	46.06	558	272	11	12	549	18	566	4.00E-138	488
	Gorai.007G376800	44.39	570	281	8	8	549	5	566	1.00E-135	480
	Gorai.013G021400	46.86	525	257	7	39	549	43	559	1.00E-134	477
	Gorai.013G025500	46.29	525	260	7	39	549	43	559	8.00E-134	474
	Gorai.012G111900	45.02	542	280	7	14	549	10	539	9.00E-134	474

	Gorai.007G378200	45.14	545	275	9	14	549	10	539	1.00E-133	473
	Gorai.009G093800	43.83	543	285	9	13	549	11	539	1.00E-132	470
	Gorai.009G103200	43.6	555	288	7	12	549	16	562	3.00E-132	469
	Gorai.013G263200	43.21	567	294	9	3	549	2	560	3.00E-132	469
	Gorai.009G321900	44.59	545	278	7	15	549	8	538	7.00E-131	464
	Gorai.011G279600	45.04	544	274	10	17	549	8	537	3.00E-130	462
	Gorai.003G124600	45.42	546	272	13	15	549	9	539	6.00E-130	461
	Gorai.003G129800	44.82	560	275	11	13	549	12	560	3.00E-129	459
	Gorai.003G129700	44.88	557	279	11	13	549	12	560	3.00E-129	459
	Gorai.002G257100	43.11	559	294	9	7	549	6	556	5.00E-129	458
	Gorai.007G376600	42.78	554	292	8	12	549	9	553	7.00E-128	454
	Gorai.009G260400	42.34	548	300	6	12	549	15	556	2.00E-126	449
	Gorai.002G261500	45.7	523	263	8	36	549	31	541	3.00E-124	442
	Gorai.007G110500	41.56	527	289	7	32	549	41	557	7.00E-118	421
	Gorai.008G126500	40.99	527	292	7	32	549	39	555	1.00E-116	417
	Gorai.009G260600	40	540	296	9	22	549	13	536	1.00E-111	400
	Gorai.009G260800	40.18	565	315	10	13	568	12	562	2.00E-108	389
	Gorai.009G103600	39.78	543	254	6	13	549	18	493	5.00E-108	388
	Gorai.009G261300	39.34	549	310	11	13	549	5	542	5.00E-108	388
	Gorai.009G261100	38.95	534	305	8	25	549	25	546	2.00E-107	387
	Gorai.009G261000	38.83	546	310	10	15	549	14	546	2.00E-104	377
	Gorai.010G130100	38.48	551	313	9	13	549	12	550	3.00E-104	376
	Gorai.009G261500	38.5	548	308	11	13	549	5	534	4.00E-104	375
	Gorai.007G205400	39.53	511	263	9	67	549	6	498	7.00E-103	371
	Gorai.005G076600	39.34	577	324	12	15	575	9	575	1.00E-102	370
	Gorai.013G027600	43.95	405	206	7	152	549	2	392	6.00E-99	358
	Gorai.003G129500	40.56	498	236	10	13	497	12	462	4.00E-92	335
	Gorai.009G323100	42.71	384	185	7	27	399	3	362	3.00E-79	293
	Gorai.013G036000	49.29	280	134	2	23	294	27	306	7.00E-78	288
	Gorai.013G036000	47.53	162	78	2	390	549	283	439	5.00E-40	162
	Gorai.010G194200	52.65	264	119	3	33	290	3	266	4.00E-75	279
	Gorai.010G194200	48.85	131	66	1	419	549	267	396	2.00E-35	147
	Gorai.010G194400	40.66	364	185	7	12	365	8	350	2.00E-70	263
	Gorai.008G103600	31.44	563	306	17	24	544	22	546	1.00E-65	247
	Gorai.003G045100	32	575	314	18	13	544	19	559	3.00E-64	243
	Gorai.004G227900	29.79	574	324	19	13	544	13	549	1.00E-58	224
	Gorai.012G109600	50.75	199	92	2	14	206	12	210	1.00E-57	221
	Gorai.009G261200	37.91	277	160	4	7	282	10	275	1.00E-48	191
	Gorai.009G260700	37.55	277	161	4	7	282	10	275	1.00E-48	191
	Gorai.009G261400	38.46	273	156	4	7	278	10	271	2.00E-48	191
	Gorai.009G287100	39.2	250	143	4	34	282	35	276	3.00E-47	187
	Gorai.009G261600	37.36	273	159	4	7	278	10	271	3.00E-46	183
	Gorai.008G073000	29.26	499	291	14	41	526	42	491	2.00E-39	160
	Gorai.009G309500	26.34	543	344	16	1	526	6	509	3.00E-38	157
	Gorai.009G309400	26.29	502	315	16	41	526	43	505	2.00E-36	150
	Gorai.009G268100	27.69	502	300	16	39	526	38	490	1.00E-34	145
	Gorai.009G179300	25.62	527	335	18	12	526	10	491	1.00E-34	144
	Gorai.002G202300	27.42	496	309	17	39	526	42	494	9.00E-34	142
	Gorai.008G038600	27.11	498	304	17	41	526	42	492	2.00E-33	141
	Gorai.002G120000	26.64	503	304	16	39	526	38	490	1.00E-32	138
	Gorai.001G048300	25.53	521	310	16	37	526	32	505	2.00E-32	137
	Gorai.007G209700	27.1	524	319	13	17	526	19	493	5.00E-32	136
	Gorai.008G236600	26.13	532	339	14	14	526	14	510	5.00E-32	136
	Gorai.010G181000	25.9	498	315	16	39	526	38	491	1.00E-31	135
	Gorai.009G189900	28	500	297	17	41	526	40	490	2.00E-31	134
	Gorai.008G189200	26.69	502	305	15	39	526	45	497	3.00E-31	134
	Gorai.007G052900	27.03	492	301	15	45	526	47	490	4.00E-30	130
	Gorai.009G194300	26.91	524	325	17	12	526	17	491	6.00E-30	129
	Gorai.001G102100	26.38	508	320	17	37	526	39	510	8.00E-30	129
	Gorai.006G212500	26.82	537	330	17	14	526	14	511	2.00E-29	127
	Gorai.007G209900	26.42	492	311	13	42	526	44	491	3.00E-29	127
	Gorai.011G104400	25.56	493	302	14	45	526	42	480	3.00E-27	120
	Gorai.002G173600	26.18	489	305	20	52	526	51	497	6.00E-24	109
	Gorai.013G027700	42.74	117	63	1	13	125	13	129	4.00E-23	106
	Gorai.013G245000	25.3	498	317	20	52	535	51	507	5.00E-22	102
	Gorai.013G244800	25.3	498	317	20	52	535	51	507	3.00E-21	100
	Gorai.013G244700	24.1	498	291	19	52	535	51	475	2.00E-19	94.4
	Gorai.013G244900	24.85	491	309	18	52	526	51	497	3.00E-19	94
	Gorai.004G098200	22.71	458	293	17	82	526	72	481	2.00E-16	84.3
	Gorai.006G269000	27.8	241	155	7	52	278	51	286	2.00E-12	71.6
**AtLAC09**	Gorai.012G110000	54.95	555	240	3	20	568	17	567	2.00E-179	626

Gorai.012G109500	55.82	550	226	6	14	551	12	556	4.00E-179	624
Gorai.002G148200	58.54	533	207	5	42	568	40	564	6.00E-178	620
Gorai.012G109700	56.61	567	229	6	14	568	12	573	5.00E-177	617
Gorai.012G109900	55.67	564	236	6	14	568	23	581	3.00E-173	605
Gorai.010G194300	46.25	573	280	9	12	568	8	568	4.00E-141	498
Gorai.010G194600	45.38	573	285	8	12	568	8	568	9.00E-140	494
Gorai.006G171500	46.06	545	267	9	40	568	50	583	9.00E-138	487
Gorai.004G234200	48.8	541	250	8	42	568	49	576	2.00E-136	483
Gorai.011G290000	46.04	543	265	7	42	568	39	569	2.00E-136	483
Gorai.003G150200	46.32	570	279	7	13	568	20	576	3.00E-135	479
Gorai.003G096600	44.23	563	293	6	12	568	16	563	6.00E-135	478
Gorai.013G263200	46.94	539	266	9	40	568	49	577	2.00E-134	476
Gorai.007G376800	45.4	544	272	7	40	568	50	583	2.00E-133	473
Gorai.011G101300	44.86	564	288	9	13	568	14	562	4.00E-133	472
Gorai.007G378200	44.72	568	294	8	7	568	3	556	6.00E-132	468
Gorai.013G036200	45.11	552	282	7	23	568	27	563	7.00E-132	468
Gorai.013G021400	46.22	543	268	7	40	568	44	576	2.00E-131	466
Gorai.013G025500	45.86	543	270	7	40	568	44	576	6.00E-131	464
Gorai.009G093800	44.47	533	274	7	42	568	40	556	8.00E-131	464
Gorai.012G111900	44.39	561	292	7	14	568	10	556	1.00E-130	464
Gorai.009G321900	45.37	562	285	9	13	568	10	555	2.00E-130	462
Gorai.002G257100	45.94	542	270	9	40	568	42	573	7.00E-130	461
Gorai.003G129800	47.05	542	264	9	40	568	46	577	9.00E-130	461
Gorai.003G129700	46.85	540	264	9	42	568	48	577	1.00E-129	460
Gorai.009G103200	45.42	546	271	7	40	568	44	579	2.00E-129	459
Gorai.007G376600	44.5	573	291	9	12	568	9	570	3.00E-129	459
Gorai.011G279600	45.23	535	271	7	40	568	36	554	3.00E-127	452
Gorai.003G124600	43.06	562	297	8	13	568	12	556	7.00E-127	451
Gorai.002G261500	43.62	564	296	9	12	568	10	558	4.00E-122	435
Gorai.009G260400	43.74	567	301	7	12	568	15	573	5.00E-121	431
Gorai.007G110500	42.57	538	288	7	40	568	49	574	1.00E-118	424
Gorai.008G126500	41.42	536	293	7	42	568	49	572	3.00E-116	416
Gorai.009G261300	40.56	567	314	10	13	568	5	559	2.00E-109	393
Gorai.009G260600	40.54	555	308	9	22	568	13	553	9.00E-109	391
Gorai.009G261100	40.52	538	297	9	40	568	40	563	2.00E-107	386
Gorai.005G076600	38.75	578	328	10	15	577	9	575	3.00E-106	382
Gorai.009G260800	40.39	567	313	10	13	570	12	562	4.00E-106	382
Gorai.009G261500	40.04	567	309	12	13	568	5	551	1.00E-105	380
Gorai.009G261000	40.48	541	293	11	40	568	40	563	4.00E-104	375
Gorai.009G103600	40.68	531	240	6	42	566	47	508	2.00E-102	370
Gorai.010G130100	39.26	540	306	9	40	569	42	569	6.00E-101	365
Gorai.007G205400	40.68	499	251	8	83	554	21	501	3.00E-99	359
Gorai.013G027600	44.44	423	214	6	152	568	2	409	1.00E-94	343
Gorai.003G129500	42.89	464	214	8	40	499	46	462	4.00E-88	322
Gorai.009G323100	43.78	370	172	7	42	400	18	362	7.00E-73	271
Gorai.010G194200	54.12	255	111	3	42	290	12	266	5.00E-70	262
Gorai.010G194200	43.2	169	81	3	414	568	246	413	2.00E-37	154
Gorai.013G036000	48.15	270	134	1	23	286	27	296	2.00E-67	253
Gorai.013G036000	47.22	180	87	3	391	568	283	456	1.00E-42	171
Gorai.010G194400	40.6	367	186	7	12	368	8	352	5.00E-64	242
Gorai.008G103600	32.5	563	296	20	40	560	38	558	9.00E-62	234
Gorai.003G045100	32.14	588	317	22	14	560	25	571	4.00E-60	229
Gorai.004G227900	30.19	593	325	21	13	560	13	561	3.00E-57	220
Gorai.012G109600	52.76	199	88	2	14	206	12	210	2.00E-55	213
Gorai.009G261200	35.91	298	170	5	8	304	11	288	1.00E-43	174
Gorai.009G261400	37.64	271	151	4	40	309	40	293	4.00E-43	173
Gorai.009G287100	37.64	263	143	5	40	289	41	295	1.00E-42	171
Gorai.009G260700	35.87	276	165	4	8	282	11	275	1.00E-42	171
Gorai.009G261600	37.13	272	153	4	40	310	40	294	3.00E-41	167
Gorai.009G309400	27.22	540	344	16	41	568	43	545	7.00E-38	155
Gorai.008G073000	27.31	542	332	16	41	570	42	533	9.00E-36	148
Gorai.009G309500	26.01	569	376	16	12	568	14	549	8.00E-35	145
Gorai.009G179300	26.76	512	317	18	41	541	40	504	9.00E-32	135
Gorai.007G052900	25.93	536	332	16	46	568	47	530	8.00E-31	132
Gorai.002G120000	25.42	539	347	14	41	570	40	532	5.00E-30	129
Gorai.009G268100	26.3	540	341	14	41	570	40	532	7.00E-30	129
Gorai.009G189900	26.67	540	339	15	41	570	40	532	2.00E-29	127
Gorai.011G104400	25.46	538	331	16	46	570	42	522	1.00E-28	124
Gorai.002G202300	26.34	543	337	18	41	570	44	536	3.00E-28	123
Gorai.007G209700	26.14	547	333	14	41	570	43	535	4.00E-28	123
Gorai.008G189200	26.49	502	304	15	41	528	47	497	4.00E-28	123
Gorai.007G209900	24.86	535	351	11	42	570	44	533	5.00E-28	123

	Gorai.008G038600	25.93	513	319	17	41	541	42	505	6.00E-28	122
	Gorai.010G181000	26.61	511	319	17	41	541	40	504	6.00E-28	122
	Gorai.001G102100	26.6	564	357	19	40	585	42	566	7.00E-26	115
	Gorai.008G236600	25.77	551	347	17	40	568	40	550	1.00E-25	114
	Gorai.001G048300	24.29	560	344	16	40	568	35	545	5.00E-25	112
	Gorai.006G212500	25.86	553	340	19	40	565	40	549	5.00E-24	109
	Gorai.002G173600	25.05	499	304	17	50	528	49	497	9.00E-24	108
	Gorai.009G194300	25.13	561	329	21	40	570	42	541	1.00E-23	108
	Gorai.013G027700	39.84	123	70	1	7	125	7	129	4.00E-22	103
	Gorai.013G245000	25.54	505	313	19	50	537	49	507	1.00E-21	101
	Gorai.013G244800	25.86	495	305	18	50	528	49	497	4.00E-21	100
	Gorai.013G244900	25	496	308	17	50	528	49	497	3.00E-20	97.1
	Gorai.013G244700	25.05	495	277	19	50	528	49	465	1.00E-17	88.6
	Gorai.004G098200	23.63	491	314	18	50	528	40	481	1.00E-16	85.1
**AtLAC10**	Gorai.007G378200	75.99	554	131	2	5	558	5	556	0	854
	Gorai.012G111900	75.99	558	132	2	1	558	1	556	0	851
	Gorai.009G093800	73.83	554	142	2	5	558	6	556	0	833
	Gorai.003G124600	72.38	554	149	3	5	558	7	556	0	815
	Gorai.002G261500	72.02	554	154	1	5	558	6	558	0	813
	Gorai.009G321900	66.24	551	183	2	8	558	8	555	0	753
	Gorai.011G279600	65.41	558	189	3	1	558	1	554	0	743
	Gorai.011G101300	60.36	550	212	4	10	558	18	562	0	684
	Gorai.003G096600	61.61	547	204	4	13	558	22	563	0	684
	Gorai.013G036200	60.34	537	209	3	23	558	30	563	0	671
	Gorai.006G171500	55.69	571	238	6	1	558	15	583	0	634
	Gorai.009G103200	55.24	563	238	5	10	558	17	579	3.00E-180	628
	Gorai.007G376800	56.55	550	225	5	22	558	35	583	8.00E-180	627
	Gorai.013G021400	56.44	551	227	5	20	558	27	576	8.00E-180	627
	Gorai.013G025500	55.17	551	234	5	20	558	27	576	1.00E-176	616
	Gorai.003G129800	53.48	574	247	8	4	558	5	577	9.00E-175	610
	Gorai.013G263200	53.53	566	247	6	7	558	14	577	4.00E-174	608
	Gorai.002G257100	54.46	549	238	6	21	558	26	573	5.00E-173	604
	Gorai.003G129700	53.43	569	249	7	5	558	10	577	1.00E-172	603
	Gorai.007G376600	52.57	565	254	5	7	558	7	570	2.00E-172	602
	Gorai.010G194300	50.35	570	261	6	3	558	7	568	1.00E-161	566
	Gorai.010G194600	50.35	570	261	6	3	558	7	568	2.00E-161	565
	Gorai.011G290000	49.03	567	269	6	8	558	7	569	4.00E-157	551
	Gorai.003G150200	49.2	565	267	7	7	558	19	576	3.00E-153	538
	Gorai.008G126500	47.97	567	272	5	9	558	12	572	2.00E-152	535
	Gorai.012G110000	51.1	546	258	3	22	558	22	567	3.00E-152	535
	Gorai.004G234200	50.55	548	253	7	24	558	34	576	4.00E-151	531
	Gorai.007G110500	47.82	573	278	5	1	558	8	574	9.00E-151	530
	Gorai.009G103600	48.74	556	221	7	5	556	13	508	1.00E-146	516
	Gorai.013G027600	59.61	411	162	3	149	558	2	409	2.00E-141	499
	Gorai.009G260400	47.47	573	286	5	1	558	1	573	3.00E-140	495
	Gorai.002G148200	47.04	540	281	2	24	558	25	564	3.00E-139	492
	Gorai.012G109900	44.82	569	297	7	6	558	14	581	6.00E-132	468
	Gorai.012G109500	45.41	555	283	7	6	541	3	556	1.00E-130	463
	Gorai.009G261100	43.7	540	296	6	24	558	27	563	2.00E-130	462
	Gorai.009G261000	43.09	557	305	7	7	558	14	563	3.00E-129	459
	Gorai.009G260600	41.8	555	316	4	6	558	4	553	9.00E-129	457
	Gorai.009G261300	42.75	559	310	6	5	558	6	559	1.00E-128	457
	Gorai.012G109700	44.22	554	291	6	6	541	3	556	2.00E-128	456
	Gorai.009G261500	42.11	558	307	6	5	558	6	551	2.00E-126	449
	Gorai.009G260800	42.29	558	313	5	5	558	8	560	2.00E-125	446
	Gorai.005G076600	40.21	562	326	7	4	558	8	566	3.00E-123	439
	Gorai.007G205400	45.34	494	234	8	80	547	21	504	2.00E-119	426
	Gorai.010G130100	40.39	562	320	9	7	558	12	568	3.00E-112	402
	Gorai.003G129500	45.67	508	204	12	4	489	5	462	5.00E-112	402
	Gorai.013G036000	62.5	272	102	0	23	294	30	301	7.00E-100	361
	Gorai.013G036000	67.92	159	50	1	401	558	298	456	2.00E-61	233
	Gorai.009G323100	43.34	383	189	5	22	397	1	362	3.00E-86	316
	Gorai.010G194200	53.82	275	127	0	35	309	8	282	2.00E-83	307
	Gorai.010G194200	66.93	127	41	1	432	558	288	413	3.00E-47	186
	Gorai.010G194400	41.08	370	185	5	3	366	7	349	2.00E-71	267
	Gorai.008G103600	33.63	553	304	19	20	536	21	546	3.00E-62	236
	Gorai.004G227900	30.89	560	338	16	5	536	11	549	8.00E-54	208
	Gorai.009G261200	39.78	279	151	5	7	285	14	275	1.00E-48	191
	Gorai.012G109600	44.71	208	111	1	6	209	3	210	2.00E-48	191
	Gorai.009G268100	28	550	360	12	10	558	16	530	6.00E-48	189
	Gorai.009G260700	37.41	278	158	4	8	285	14	275	2.00E-47	187

	Gorai.009G287100	40.93	259	140	3	27	285	31	276	4.00E-47	186
	Gorai.009G261400	38.85	260	146	4	24	283	27	273	4.00E-44	176
	Gorai.002G120000	27.29	535	357	11	25	558	27	530	1.00E-43	174
	Gorai.009G309400	27.36	552	372	13	13	558	17	545	2.00E-43	173
	Gorai.009G261600	37.69	260	149	4	24	283	27	273	7.00E-43	172
	Gorai.002G202300	26.92	561	362	17	5	558	15	534	9.00E-43	171
	Gorai.007G052900	27.92	523	327	13	27	540	31	512	3.00E-42	170
	Gorai.009G179300	27.27	517	336	13	11	518	6	491	7.00E-42	169
	Gorai.009G189900	26.6	515	346	11	25	538	27	510	1.00E-41	168
	Gorai.008G038600	26.83	518	341	13	25	538	29	512	8.00E-41	165
	Gorai.010G181000	27.33	516	333	13	13	518	8	491	4.00E-40	163
	Gorai.007G209900	27.6	500	322	11	27	522	32	495	1.00E-39	161
	Gorai.008G236600	25.73	579	365	17	7	558	10	550	6.00E-39	159
	Gorai.009G309500	27.51	567	364	17	8	558	14	549	6.00E-39	159
	Gorai.008G073000	26.88	532	328	14	7	524	13	497	1.00E-38	158
	Gorai.008G189200	26.77	523	331	14	8	518	15	497	6.00E-37	152
	Gorai.006G212500	27.01	548	358	17	27	558	30	551	4.00E-36	149
	Gorai.011G104400	27.35	501	309	17	27	518	26	480	4.00E-36	149
	Gorai.007G209700	28.29	502	310	12	27	518	32	493	5.00E-36	149
	Gorai.013G027700	61.34	119	44	1	3	121	12	128	1.00E-34	144
	Gorai.001G048300	25.94	532	351	17	43	558	41	545	1.00E-34	144
	Gorai.001G102100	26.71	513	331	16	27	523	32	515	5.00E-33	139
	Gorai.009G194300	25.67	561	335	17	27	558	32	539	7.00E-33	139
	Gorai.003G045100	34.21	304	170	9	6	283	15	314	8.00E-33	138
	Gorai.003G045100	37.89	161	96	2	380	536	399	559	2.00E-27	120
	Gorai.002G173600	25.63	558	368	18	4	549	3	525	7.00E-32	135
	Gorai.013G245000	25.14	529	353	15	7	524	7	503	7.00E-31	132
	Gorai.013G244900	26.16	516	331	16	46	549	48	525	3.00E-30	130
	Gorai.013G244800	25.05	507	339	14	27	524	29	503	2.00E-29	127
	Gorai.004G098200	25.1	482	325	13	46	524	39	487	4.00E-26	116
	Gorai.013G244700	24	500	321	13	27	524	29	471	2.00E-25	114
	Gorai.003G096200	37.08	178	63	3	13	189	12	141	6.00E-19	92.8
**AtLAC11**	Gorai.003G096600	80.71	539	104	0	19	557	25	563	0	935
	Gorai.011G101300	79.18	538	112	0	20	557	25	562	0	924
	Gorai.013G036200	81.04	538	102	0	20	557	26	563	0	920
	Gorai.009G093800	64.19	539	192	1	19	557	19	556	0	739
	Gorai.007G378200	62.15	539	201	2	21	557	19	556	0	725
	Gorai.003G124600	62.76	537	198	2	21	557	22	556	0	724
	Gorai.012G111900	61.78	539	203	2	21	557	19	556	0	719
	Gorai.011G279600	59.59	537	216	1	21	557	19	554	0	687
	Gorai.013G027600	78.43	408	88	0	150	557	2	409	0	687
	Gorai.002G261500	60.3	539	211	2	21	557	21	558	0	678
	Gorai.009G321900	59.37	539	218	1	19	557	18	555	0	672
	Gorai.006G171500	58.68	547	210	8	26	557	38	583	0	671
	Gorai.013G021400	59.42	552	209	8	20	557	26	576	0	671
	Gorai.007G376800	58.14	547	213	7	26	557	38	583	0	670
	Gorai.009G103200	58.2	555	214	8	20	557	26	579	0	667
	Gorai.013G025500	58.15	552	216	8	20	557	26	576	0	665
	Gorai.013G263200	56.23	546	226	6	23	557	34	577	0	642
	Gorai.003G129800	56.96	546	219	9	26	557	34	577	0	632
	Gorai.003G129700	56.59	546	221	9	26	557	34	577	7.00E-180	627
	Gorai.007G376600	54.48	547	233	6	26	557	25	570	2.00E-177	619
	Gorai.002G257100	55.45	550	229	8	22	557	26	573	4.00E-177	618
	Gorai.010G194300	51.27	552	249	6	22	557	21	568	2.00E-169	592
	Gorai.010G194600	51.45	552	248	6	22	557	21	568	2.00E-169	592
	Gorai.003G150200	51	551	252	5	21	557	30	576	2.00E-167	585
	Gorai.011G290000	51.26	554	249	6	21	557	20	569	1.00E-166	583
	Gorai.004G234200	50.82	547	251	5	25	557	34	576	6.00E-165	577
	Gorai.012G110000	49.36	547	266	5	22	557	21	567	8.00E-159	557
	Gorai.007G110500	49.55	551	258	8	20	557	31	574	5.00E-155	545
	Gorai.008G126500	49.55	553	255	11	20	557	29	572	5.00E-154	541
	Gorai.009G103600	50.55	542	202	7	20	555	27	508	4.00E-151	531
	Gorai.002G148200	48.52	542	272	4	23	557	23	564	2.00E-148	523
	Gorai.009G260400	48.82	551	271	5	18	557	23	573	2.00E-147	520
	Gorai.012G109900	47.27	550	277	6	21	557	32	581	1.00E-141	500
	Gorai.012G109500	46.08	536	273	4	21	540	21	556	3.00E-141	499
	Gorai.012G109700	46.27	536	272	5	21	540	21	556	3.00E-141	499
	Gorai.013G036000	81.39	274	51	0	20	293	26	299	2.00E-138	489
	Gorai.013G036000	81.13	159	30	0	399	557	298	456	3.00E-77	286
	Gorai.009G260600	43.87	538	299	2	23	557	16	553	1.00E-136	483
	Gorai.009G261300	43.81	541	298	4	23	557	19	559	3.00E-135	478

	Gorai.009G261100	42.49	539	306	3	23	557	25	563	5.00E-133	471
	Gorai.009G260800	43.52	540	300	3	23	557	21	560	7.00E-133	471
	Gorai.009G261000	43.07	541	300	3	23	557	25	563	2.00E-131	466
	Gorai.009G261500	42.59	540	298	4	23	557	19	551	2.00E-131	466
	Gorai.005G076600	42.2	545	307	6	21	557	22	566	3.00E-131	465
	Gorai.010G130100	41.58	546	304	8	23	557	27	568	5.00E-126	448
	Gorai.003G129500	50.84	474	177	10	26	488	34	462	1.00E-125	447
	Gorai.007G205400	47.84	487	220	8	81	541	21	499	1.00E-125	446
	Gorai.009G323100	46.48	383	170	6	25	395	3	362	4.00E-97	352
	Gorai.010G194200	53.99	276	126	1	31	305	3	278	2.00E-89	326
	Gorai.010G194200	59.84	127	50	1	431	557	288	413	6.00E-44	175
	Gorai.010G194400	44.63	354	166	5	21	367	20	350	9.00E-85	311
	Gorai.003G045100	35.45	550	300	15	19	535	32	559	1.00E-74	277
	Gorai.008G103600	33.09	547	313	14	21	535	21	546	6.00E-71	265
	Gorai.004G227900	30.66	548	321	19	23	536	28	550	2.00E-63	240
	Gorai.009G260700	37.27	271	157	3	23	293	25	282	1.00E-51	201
	Gorai.012G109600	47.62	189	99	0	21	209	21	209	6.00E-51	199
	Gorai.009G261200	37.88	264	151	4	23	286	25	275	8.00E-50	195
	Gorai.009G261400	37.02	262	152	3	23	284	25	273	2.00E-48	191
	Gorai.009G287100	35.97	278	159	4	28	305	31	289	4.00E-48	189
	Gorai.013G027700	77.88	104	23	0	20	123	26	129	1.00E-47	187
	Gorai.009G261600	37.4	262	151	4	23	284	25	273	1.00E-46	184
	Gorai.009G268100	29.41	544	332	18	26	557	27	530	2.00E-46	184
	Gorai.009G309400	28.01	532	331	14	44	557	48	545	9.00E-46	181
	Gorai.002G120000	30.59	523	311	18	47	557	48	530	2.00E-44	177
	Gorai.009G309500	29.01	493	315	15	44	524	48	517	2.00E-44	177
	Gorai.009G179300	28.87	523	321	19	47	557	48	531	2.00E-43	174
	Gorai.002G202300	28.29	516	332	15	47	557	52	534	3.00E-43	173
	Gorai.010G181000	28.87	523	321	19	47	557	48	531	2.00E-41	167
	Gorai.008G038600	27.6	500	316	16	47	537	50	512	2.00E-41	167
	Gorai.009G189900	28.49	523	322	17	47	557	48	530	2.00E-40	163
	Gorai.008G073000	26.99	515	313	14	44	541	47	515	4.00E-40	162
	Gorai.008G236600	27.84	546	353	17	28	557	30	550	5.00E-40	162
	Gorai.001G048300	27	537	337	15	44	557	41	545	7.00E-40	162
	Gorai.007G052900	29.61	510	301	18	44	539	47	512	8.00E-39	158
	Gorai.007G209700	28.46	506	318	13	44	541	48	517	3.00E-38	157
	Gorai.006G212500	27.66	546	355	16	28	557	30	551	5.00E-38	155
	Gorai.008G189200	27.94	544	340	16	26	557	34	537	6.00E-38	155
	Gorai.003G096200	45.11	184	50	3	19	201	15	148	1.00E-37	154
	Gorai.007G209900	29.31	505	305	17	28	521	32	495	6.00E-37	152
	Gorai.011G104400	27.52	505	325	14	17	517	13	480	1.00E-36	151
	Gorai.001G102100	28.44	545	335	16	28	550	32	543	1.00E-36	151
	Gorai.009G194300	28.63	503	303	16	28	517	32	491	6.00E-35	145
	Gorai.002G173600	27.52	487	312	16	47	523	48	503	7.00E-33	139
	Gorai.013G245000	26.13	486	320	14	47	523	48	503	1.00E-32	138
	Gorai.013G244800	26.13	486	320	14	47	523	48	503	1.00E-31	135
	Gorai.013G244900	28.48	488	306	17	47	523	48	503	7.00E-29	125
	Gorai.013G244700	25.47	483	295	13	47	523	48	471	2.00E-27	121
	Gorai.004G098200	26.39	485	313	15	47	523	39	487	1.00E-26	118
**AtLAC12**	Gorai.010G194300	80.29	548	101	2	24	565	22	568	0	937
	Gorai.010G194600	79.74	548	104	2	24	565	22	568	0	931
	Gorai.011G290000	78.69	549	109	2	24	565	22	569	0	915
	Gorai.004G234200	66.85	546	172	4	26	565	34	576	0	769
	Gorai.003G150200	66.12	549	175	5	24	565	32	576	0	758
	Gorai.012G110000	52.46	549	251	3	24	565	22	567	4.00E-173	605
	Gorai.006G171500	53.35	553	237	9	27	565	38	583	2.00E-172	602
	Gorai.003G096600	53.3	546	240	6	24	565	29	563	2.00E-172	602
	Gorai.002G148200	53.92	549	239	5	24	565	23	564	2.00E-169	592
	Gorai.011G101300	52.2	546	246	6	24	565	28	562	2.00E-169	592
	Gorai.013G036200	53.66	546	238	6	24	565	29	563	9.00E-169	590
	Gorai.007G376800	52.62	553	241	8	27	565	38	583	3.00E-166	582
	Gorai.012G111900	52.02	544	247	5	26	565	23	556	5.00E-166	581
	Gorai.007G378200	51.65	544	249	5	26	565	23	556	6.00E-166	580
	Gorai.003G129700	53.36	551	238	10	27	565	34	577	2.00E-165	578
	Gorai.013G021400	52.17	552	244	10	27	565	32	576	6.00E-165	577
	Gorai.013G263200	52.55	548	244	8	27	565	37	577	1.00E-164	576
	Gorai.012G109700	53.16	538	235	6	24	548	23	556	3.00E-164	575
	Gorai.002G257100	52.71	554	243	9	24	565	27	573	1.00E-163	573
	Gorai.003G129800	53.18	551	239	10	27	565	34	577	1.00E-163	573
	Gorai.009G093800	51.47	544	248	6	26	565	25	556	3.00E-163	572
	Gorai.012G109500	52.04	538	241	6	24	548	23	556	4.00E-163	571

	Gorai.012G109900	53.26	552	244	6	24	565	34	581	5.00E-163	571
	Gorai.007G376600	51.08	556	251	8	24	565	22	570	7.00E-163	570
	Gorai.003G124600	51.47	544	247	7	26	565	26	556	6.00E-162	567
	Gorai.011G279600	51.28	546	250	5	24	565	21	554	5.00E-161	564
	Gorai.009G103200	50.45	555	252	9	27	565	32	579	5.00E-160	561
	Gorai.013G025500	50.63	555	248	10	27	565	32	576	9.00E-160	560
	Gorai.002G261500	51.65	546	250	5	24	565	23	558	7.00E-159	557
	Gorai.009G323100	69.29	381	96	2	24	402	1	362	2.00E-158	555
	Gorai.009G260400	51.09	548	256	4	26	565	30	573	8.00E-158	553
	Gorai.008G126500	49.35	541	269	3	27	565	35	572	6.00E-157	551
	Gorai.007G110500	50.09	539	264	3	29	565	39	574	1.00E-155	546
	Gorai.009G321900	49.27	546	261	6	24	565	22	555	4.00E-155	545
	Gorai.010G194400	70.11	348	79	3	24	365	22	350	3.00E-140	495
	Gorai.010G194200	82.01	278	50	0	33	310	4	281	5.00E-139	491
	Gorai.010G194200	85.29	136	19	1	430	565	279	413	7.00E-68	255
	Gorai.009G260600	44.71	548	287	6	24	565	16	553	2.00E-137	486
	Gorai.009G261100	44.28	551	286	9	24	565	25	563	4.00E-135	478
	Gorai.009G260800	43.33	547	298	6	24	565	21	560	6.00E-135	478
	Gorai.009G261300	43.43	548	297	7	24	565	19	559	2.00E-134	476
	Gorai.005G076600	42.57	552	298	10	24	565	24	566	8.00E-134	474
	Gorai.009G261500	42.78	547	294	7	24	565	19	551	2.00E-132	469
	Gorai.009G261000	43.09	550	294	7	24	565	25	563	2.00E-132	469
	Gorai.009G103600	45.02	542	227	7	27	563	33	508	4.00E-129	459
	Gorai.010G130100	42.57	552	297	11	24	565	27	568	1.00E-128	457
	Gorai.013G027600	53.7	419	179	6	151	565	2	409	5.00E-128	454
	Gorai.007G205400	47.31	501	217	9	82	553	21	503	2.00E-123	439
	Gorai.003G129500	47.92	480	189	12	27	496	34	462	8.00E-113	404
	Gorai.013G036000	54.85	268	121	0	24	291	29	296	2.00E-89	327
	Gorai.013G036000	56.96	158	67	1	408	565	300	456	4.00E-51	199
	Gorai.008G103600	32.5	560	302	21	24	543	23	546	2.00E-64	244
	Gorai.004G227900	31.52	552	316	19	24	543	28	549	4.00E-64	243
	Gorai.003G045100	32.44	558	305	21	24	543	36	559	3.00E-62	236
	Gorai.012G109600	56.76	185	80	0	24	208	23	207	3.00E-60	229
	Gorai.009G260700	38.41	289	158	4	24	312	25	293	4.00E-56	216
	Gorai.009G261400	39.79	289	154	5	24	312	25	293	5.00E-56	215
	Gorai.009G261200	38.97	290	155	6	24	312	25	293	6.00E-56	215
	Gorai.009G261600	38.28	290	159	5	24	313	25	294	9.00E-55	211
	Gorai.009G287100	37.32	284	158	4	28	311	30	293	4.00E-52	202
	Gorai.009G309500	27.27	539	355	18	36	565	39	549	2.00E-43	174
	Gorai.001G048300	25.63	554	360	17	30	565	26	545	9.00E-40	162
	Gorai.007G052900	28	525	333	14	45	565	47	530	2.00E-38	157
	Gorai.010G181000	28.13	487	312	13	41	525	41	491	4.00E-38	156
	Gorai.009G179300	27.78	504	317	15	25	525	32	491	1.00E-37	154
	Gorai.009G268100	28.34	494	301	14	41	525	41	490	2.00E-37	154
	Gorai.002G120000	28.16	490	311	13	45	531	45	496	2.00E-37	154
	Gorai.009G309400	25.14	529	365	12	41	565	44	545	8.00E-37	152
	Gorai.008G073000	25.81	492	319	12	45	531	47	497	4.00E-36	149
	Gorai.009G189900	28.28	495	310	14	41	530	41	495	5.00E-35	146
	Gorai.007G209900	26.7	528	344	13	41	565	44	531	7.00E-35	145
	Gorai.008G236600	26.34	505	340	14	29	525	30	510	7.00E-34	142
	Gorai.006G212500	27.22	507	334	16	29	525	30	511	8.00E-34	142
	Gorai.011G104400	28.22	489	293	19	45	525	42	480	3.00E-31	133
	Gorai.002G202300	26.38	489	317	14	41	525	45	494	8.00E-31	132
	Gorai.008G038600	25.99	531	346	17	41	565	43	532	8.00E-31	132
	Gorai.008G189200	25.67	483	320	11	45	525	52	497	2.00E-30	130
	Gorai.001G102100	25.05	511	347	14	29	530	32	515	2.00E-29	127
	Gorai.013G245000	23.41	487	341	12	47	531	47	503	5.00E-29	126
	Gorai.007G209700	25.87	487	314	12	45	525	48	493	5.00E-29	126
	Gorai.013G027700	52.48	101	48	0	24	124	29	129	1.00E-27	122
	Gorai.013G244800	23	487	343	12	47	531	47	503	2.00E-27	121
	Gorai.002G173600	23.08	481	338	11	47	525	47	497	4.00E-27	120
	Gorai.009G194300	24.6	500	334	13	29	525	32	491	4.00E-27	119
	Gorai.004G098200	24.07	482	325	14	47	525	38	481	1.00E-24	111
	Gorai.013G244900	23.24	482	336	14	47	525	47	497	1.00E-23	108
	Gorai.013G244700	24.82	274	195	5	47	319	47	310	7.00E-18	89.4
	Gorai.006G269000	24.8	254	160	7	51	287	51	290	3.00E-14	77
**AtLAC13**	Gorai.003G150200	74.33	561	140	4	9	569	20	576	0	800
	Gorai.004G234200	74.73	562	138	4	8	569	19	576	0	782
	Gorai.010G194300	67.08	565	178	7	8	569	9	568	0	708
	Gorai.010G194600	66.73	565	180	7	8	569	9	568	0	707
	Gorai.011G290000	65.9	566	186	6	7	569	8	569	0	696

Gorai.006G171500	48.62	578	279	9	4	569	12	583	1.00E-146	517
Gorai.007G376800	49.64	550	265	7	26	569	40	583	5.00E-143	504
Gorai.012G110000	48.93	562	268	7	16	569	17	567	1.00E-140	497
Gorai.013G036200	50.63	555	252	8	19	569	27	563	3.00E-140	495
Gorai.011G101300	49.03	567	267	9	7	569	14	562	5.00E-140	494
Gorai.003G096600	49.21	573	266	9	4	569	9	563	2.00E-139	493
Gorai.012G111900	48.67	565	271	9	8	569	8	556	3.00E-138	489
Gorai.007G378200	47.44	586	261	9	1	569	1	556	7.00E-138	488
Gorai.009G103200	47.14	577	285	10	6	569	10	579	4.00E-137	485
Gorai.013G263200	49.64	550	260	8	26	569	39	577	4.00E-137	485
Gorai.003G129700	50.27	551	258	9	26	569	36	577	5.00E-137	484
Gorai.012G109900	48.89	583	273	12	1	569	10	581	5.00E-137	484
Gorai.003G129800	50.27	551	258	9	26	569	36	577	7.00E-137	484
Gorai.002G148200	48.83	557	262	10	21	569	23	564	1.00E-136	483
Gorai.013G021400	47.82	550	274	8	26	569	34	576	1.00E-135	479
Gorai.013G025500	47.82	550	274	8	26	569	34	576	2.00E-134	476
Gorai.007G376600	47.63	569	283	10	8	569	10	570	6.00E-134	474
Gorai.002G257100	47.89	570	279	10	9	569	13	573	2.00E-133	473
Gorai.008G126500	46.2	552	275	8	25	569	36	572	3.00E-133	472
Gorai.011G279600	46.64	566	279	10	8	569	8	554	2.00E-131	466
Gorai.009G093800	47.78	563	279	9	7	569	9	556	2.00E-131	466
Gorai.007G110500	45.65	552	278	8	25	569	38	574	3.00E-131	465
Gorai.003G124600	45.61	570	286	9	4	569	7	556	3.00E-131	465
Gorai.009G260400	47.91	549	273	7	26	569	33	573	4.00E-131	465
Gorai.012G109700	48.83	557	259	10	10	552	12	556	3.00E-130	462
Gorai.012G109500	46.32	557	273	10	10	552	12	556	5.00E-130	461
Gorai.002G261500	47.72	570	277	8	4	569	6	558	1.00E-129	460
Gorai.009G321900	44.62	567	291	10	7	569	8	555	2.00E-124	443
Gorai.009G260600	43.01	572	300	10	4	569	2	553	2.00E-118	423
Gorai.009G261300	42.63	570	305	9	7	569	5	559	5.00E-118	421
Gorai.009G323100	59.44	392	127	7	21	408	1	364	6.00E-118	421
Gorai.009G261500	41.58	570	303	10	7	569	5	551	5.00E-113	405
Gorai.009G261100	41.16	554	306	6	21	569	25	563	9.00E-113	404
Gorai.009G260800	40.14	558	313	8	18	569	18	560	2.00E-112	403
Gorai.009G261000	41.26	555	304	7	21	569	25	563	1.00E-111	400
Gorai.010G130100	40	575	315	10	8	569	11	568	4.00E-111	399
Gorai.009G103600	42.5	560	253	11	8	567	18	508	6.00E-111	398
Gorai.010G194400	59.4	367	125	6	8	373	9	352	5.00E-109	392
Gorai.005G076600	39.08	568	330	9	7	569	10	566	3.00E-108	389
Gorai.010G194200	72.58	248	66	2	30	277	4	249	6.00E-107	385
Gorai.010G194200	66.18	136	45	1	434	569	279	413	5.00E-52	202
Gorai.007G205400	43.82	502	240	11	80	558	22	504	1.00E-96	350
Gorai.013G027600	48.94	425	196	8	149	569	2	409	6.00E-96	348
Gorai.003G129500	43.87	481	210	12	26	500	36	462	1.00E-89	327
Gorai.013G036000	54.44	259	116	2	19	277	27	283	2.00E-79	293
Gorai.013G036000	56.03	141	62	0	429	569	316	456	6.00E-45	179
Gorai.008G103600	30.69	554	331	16	19	547	21	546	1.00E-54	211
Gorai.012G109600	49.25	201	100	2	10	210	12	210	5.00E-53	205
Gorai.009G260700	40.23	266	144	6	10	275	14	264	3.00E-52	203
Gorai.009G261200	39.25	265	146	6	10	274	14	263	5.00E-51	199
Gorai.009G261400	40.3	268	143	7	8	275	14	264	6.00E-51	199
Gorai.009G261600	39.55	268	145	7	8	275	14	264	2.00E-50	197
Gorai.009G287100	38.98	254	140	7	22	275	27	265	5.00E-48	189
Gorai.004G227900	30.79	302	165	10	8	277	11	300	6.00E-31	132
Gorai.004G227900	45.45	110	56	2	441	547	441	549	9.00E-24	108
Gorai.003G045100	29.93	294	168	8	10	277	25	306	3.00E-29	127
Gorai.003G045100	48.18	110	54	1	441	547	450	559	4.00E-26	116
Gorai.013G027700	49.15	118	57	1	7	121	12	129	5.00E-28	122
Gorai.009G268100	30.77	273	172	7	7	276	10	268	3.00E-27	120
Gorai.009G189900	31.23	269	168	7	11	276	14	268	7.00E-26	115
Gorai.009G309500	28.67	279	184	8	6	275	7	279	2.00E-25	114
Gorai.009G179300	30.51	272	170	8	8	275	11	267	2.00E-25	114
Gorai.002G120000	33.62	235	139	7	45	276	48	268	2.00E-24	111
Gorai.007G052900	32.48	274	166	8	8	277	11	269	5.00E-24	109
Gorai.010G181000	30.32	277	172	8	9	277	6	269	5.00E-24	109
Gorai.009G309400	29.1	268	184	6	10	275	16	279	9.00E-24	108
Gorai.008G189200	27.47	273	183	6	7	276	15	275	6.00E-23	105
Gorai.002G202300	29.54	281	175	9	1	275	8	271	4.00E-22	103
Gorai.008G236600	30.07	276	181	7	7	275	10	280	3.00E-21	100
Gorai.008G038600	30.47	233	147	6	45	275	50	269	2.00E-20	97.8
Gorai.008G073000	27.8	277	174	9	4	272	7	265	3.00E-20	97.1
Gorai.006G212500	29.96	277	181	7	7	275	10	281	7.00E-20	95.9

	Gorai.011G104400	30.77	273	168	9	10	277	8	264	8.00E-20	95.5
	Gorai.007G209900	29.67	273	171	7	10	277	14	270	2.00E-19	94
	Gorai.001G048300	29.18	257	171	5	25	275	24	275	2.00E-18	90.9
	Gorai.001G102100	27.38	263	168	7	25	275	31	282	5.00E-17	86.3
	Gorai.013G244900	28.81	236	156	8	45	277	48	274	8.00E-17	85.5
	Gorai.002G173600	26.92	234	163	6	45	277	48	274	3.00E-16	84
	Gorai.013G245000	26.5	234	164	6	45	277	48	274	5.00E-16	83.2
	Gorai.013G244700	26.5	234	164	6	45	277	48	274	6.00E-16	82.8
	Gorai.013G244800	26.5	234	164	6	45	277	48	274	7.00E-16	82.4
	Gorai.009G194300	25.6	250	173	5	25	273	31	268	1.00E-14	78.6
	Gorai.007G209700	27.78	288	169	9	1	272	1	265	2.00E-14	78.2
	Gorai.004G098200	25.83	240	158	5	45	277	39	265	3.00E-14	77
	Gorai.006G269000	24.8	246	149	8	48	275	51	278	2.00E-13	74.7
**AtLAC14**	Gorai.009G260400	55.26	570	240	6	13	569	6	573	3.00E-165	578
	Gorai.011G290000	49.91	573	268	6	12	569	1	569	1.00E-158	556
	Gorai.010G194300	50.35	564	261	7	20	569	10	568	6.00E-157	551
	Gorai.010G194600	50.18	564	262	7	20	569	10	568	1.00E-156	550
	Gorai.004G234200	52.2	546	247	7	35	569	34	576	6.00E-154	541
	Gorai.003G150200	50.89	562	261	8	19	569	19	576	3.00E-153	538
	Gorai.006G171500	46.06	571	288	10	15	569	17	583	9.00E-146	513
	Gorai.003G096600	48.29	555	273	7	21	569	17	563	2.00E-145	513
	Gorai.007G376800	46.79	560	278	9	26	569	28	583	8.00E-145	510
	Gorai.011G101300	48.39	558	272	8	20	569	13	562	8.00E-144	507
	Gorai.013G036200	48.44	545	267	7	31	569	27	563	1.00E-141	500
	Gorai.002G257100	47.54	570	273	11	18	569	12	573	3.00E-141	499
	Gorai.013G021400	46.98	547	273	10	37	569	33	576	6.00E-141	498
	Gorai.013G025500	46.73	550	270	10	37	569	33	576	1.00E-140	496
	Gorai.009G103200	45.94	579	289	13	11	569	5	579	2.00E-140	496
	Gorai.009G260600	46.77	558	281	8	19	569	5	553	3.00E-139	492
	Gorai.009G261100	47.71	547	268	10	33	569	25	563	5.00E-139	491
	Gorai.013G263200	44.33	573	302	8	12	569	7	577	9.00E-138	487
	Gorai.003G129800	45.69	580	290	12	12	569	1	577	2.00E-137	486
	Gorai.007G376600	45.42	568	291	10	18	569	6	570	3.00E-137	485
	Gorai.009G261000	46.17	561	281	9	19	569	14	563	1.00E-136	483
	Gorai.009G093800	46.42	558	286	7	17	569	7	556	2.00E-136	483
	Gorai.012G110000	43.38	551	297	6	31	569	20	567	2.00E-136	482
	Gorai.003G129700	45.47	574	288	12	18	569	7	577	1.00E-135	480
	Gorai.007G378200	46.06	558	283	8	22	569	7	556	2.00E-134	476
	Gorai.010G130100	46.43	560	281	11	22	569	16	568	9.00E-134	474
	Gorai.003G124600	45.78	557	287	9	19	569	9	556	1.00E-133	473
	Gorai.009G261300	45.63	561	286	10	19	569	8	559	2.00E-133	473
	Gorai.012G111900	45.2	562	292	8	16	569	3	556	2.00E-133	473
	Gorai.009G261500	45.71	560	279	10	19	569	8	551	1.00E-132	470
	Gorai.009G260800	45.36	560	288	9	19	569	10	560	2.00E-132	469
	Gorai.011G279600	45.63	561	288	8	15	569	5	554	7.00E-132	468
	Gorai.002G261500	45.7	547	282	7	30	569	20	558	1.00E-131	467
	Gorai.005G076600	44.17	566	293	11	19	569	9	566	1.00E-130	463
	Gorai.009G321900	45.16	558	292	8	18	569	6	555	1.00E-130	463
	Gorai.012G109500	43.69	547	290	8	22	552	12	556	2.00E-129	459
	Gorai.012G109700	43.69	547	290	8	22	552	12	556	4.00E-127	452
	Gorai.002G148200	42.52	548	298	7	33	569	23	564	1.00E-126	450
	Gorai.012G109900	42.17	562	308	7	22	569	23	581	2.00E-125	446
	Gorai.007G110500	43.44	541	292	6	38	569	39	574	6.00E-120	428
	Gorai.008G126500	43.43	548	296	6	31	569	30	572	3.00E-119	426
	Gorai.009G103600	38.86	561	272	8	15	567	11	508	3.00E-107	386
	Gorai.013G027600	47.12	416	206	7	160	569	2	409	4.00E-103	372
	Gorai.007G205400	40.89	494	250	8	91	555	21	501	3.00E-101	365
	Gorai.003G129500	41.43	502	229	13	18	500	7	462	6.00E-91	332
	Gorai.009G323100	51.79	307	146	2	33	338	1	306	2.00E-88	323
	Gorai.010G194200	55.6	277	122	1	41	317	3	278	1.00E-86	317
	Gorai.010G194200	51.18	127	61	1	443	569	288	413	1.00E-36	151
	Gorai.010G194400	44.6	361	174	3	20	375	10	349	2.00E-82	303
	Gorai.013G036000	49.08	271	137	1	31	301	27	296	3.00E-77	286
	Gorai.013G036000	52.83	159	73	1	413	569	298	456	1.00E-46	184
	Gorai.009G261200	40.47	299	154	6	19	317	14	288	2.00E-57	221
	Gorai.009G260700	40.34	295	161	5	22	315	14	294	1.00E-56	218
	Gorai.009G261400	42.65	279	146	4	22	300	14	278	9.00E-56	214
	Gorai.009G261600	41.73	278	148	4	22	299	14	277	2.00E-55	214
	Gorai.012G109600	49.5	200	100	1	22	221	12	210	9.00E-54	208
	Gorai.009G287100	40.14	284	149	6	34	317	27	289	7.00E-52	202
	Gorai.008G103600	32.14	557	334	17	31	559	21	561	8.00E-52	202

	Gorai.004G227900	29.97	584	314	25	16	547	9	549	3.00E-51	200
	Gorai.009G309500	27.59	569	364	19	18	569	12	549	1.00E-32	138
	Gorai.009G179300	26.97	519	332	18	20	529	11	491	9.00E-32	135
	Gorai.008G073000	27.74	584	333	18	19	569	4	531	4.00E-31	133
	Gorai.008G236600	26.15	543	359	17	38	565	30	545	4.00E-30	129
	Gorai.006G212500	25.67	557	373	17	24	565	16	546	9.00E-30	128
	Gorai.009G268100	27.38	504	284	16	57	534	48	495	2.00E-29	127
	Gorai.002G202300	27.26	532	329	19	13	529	6	494	8.00E-29	125
	Gorai.002G120000	26.34	505	288	17	57	534	48	495	9.00E-29	125
	Gorai.003G045100	32.01	303	176	9	11	288	9	306	1.00E-28	124
	Gorai.003G045100	49.17	120	55	2	446	559	455	574	2.00E-23	107
	Gorai.010G181000	26.25	522	342	17	15	529	6	491	2.00E-28	124
	Gorai.007G209900	25.63	554	317	19	18	541	10	498	2.00E-28	124
	Gorai.007G209700	27.22	540	300	17	31	541	25	500	2.00E-28	124
	Gorai.007G052900	25.23	543	314	17	18	529	9	490	7.00E-28	122
	Gorai.011G104400	26.4	534	340	16	15	542	2	488	1.00E-27	121
	Gorai.001G048300	26.94	542	352	19	40	565	27	540	4.00E-27	119
	Gorai.008G038600	28.45	485	293	16	57	529	50	492	9.00E-27	118
	Gorai.009G189900	25.84	503	293	15	57	534	48	495	2.00E-25	114
	Gorai.008G189200	25.8	531	326	15	18	529	16	497	2.00E-25	114
	Gorai.001G102100	25	528	353	14	50	562	44	543	3.00E-24	110
	Gorai.013G245000	25.83	515	298	18	55	538	46	507	4.00E-24	110
	Gorai.002G173600	25.23	551	322	20	19	535	9	503	6.00E-24	109
	Gorai.013G244800	25.54	513	298	18	57	538	48	507	6.00E-23	106
	Gorai.013G027700	45.69	116	63	0	18	133	14	129	7.00E-23	105
	Gorai.013G244900	25.26	586	340	23	8	558	3	525	1.00E-22	104
	Gorai.013G244700	25.97	489	298	16	55	538	46	475	1.00E-21	101
	Gorai.009G309400	29.03	248	171	4	50	295	44	288	2.00E-20	97.8
	Gorai.009G309400	33.33	132	80	3	440	569	420	545	5.00E-11	66.2
	Gorai.004G098200	24.61	512	297	19	57	538	39	491	7.00E-20	95.9
	Gorai.009G194300	27.31	249	166	4	55	299	49	286	1.00E-14	79
**AtLAC15**	Gorai.009G260400	58.57	531	209	8	24	548	33	558	2.00E-171	598
	Gorai.010G194300	47.28	533	267	9	22	546	25	551	2.00E-137	486
	Gorai.011G290000	47.57	534	265	9	22	546	25	552	3.00E-137	485
	Gorai.010G194600	46.9	533	269	9	22	546	25	551	8.00E-137	484
	Gorai.004G234200	46.98	530	271	8	22	546	35	559	3.00E-134	475
	Gorai.003G150200	46.98	530	271	9	22	546	35	559	7.00E-134	474
	Gorai.006G171500	45.32	545	280	11	20	553	36	573	6.00E-132	468
	Gorai.012G110000	46.4	541	271	9	23	553	26	557	1.00E-131	467
	Gorai.009G260800	45.97	533	269	9	21	546	23	543	2.00E-130	462
	Gorai.007G376800	44.67	544	285	9	20	553	36	573	9.00E-130	460
	Gorai.009G260600	46.98	530	264	9	22	546	19	536	3.00E-129	459
	Gorai.012G109500	44.9	539	271	10	24	546	28	556	8.00E-128	454
	Gorai.009G261300	46.15	533	267	10	22	546	22	542	4.00E-127	452
	Gorai.002G257100	46.4	541	272	11	23	553	31	563	5.00E-126	448
	Gorai.009G261100	45.39	542	272	11	21	553	27	553	9.00E-126	447
	Gorai.013G021400	44.67	544	284	9	20	553	30	566	1.00E-124	444
	Gorai.009G261000	45.49	543	270	11	21	553	27	553	1.00E-124	443
	Gorai.013G263200	44.26	540	288	9	20	553	35	567	2.00E-124	443
	Gorai.009G103200	44.77	545	285	8	20	553	30	569	2.00E-124	442
	Gorai.003G129700	45.79	546	274	13	20	553	32	567	3.00E-124	442
	Gorai.012G109700	44.34	539	274	10	24	546	28	556	3.00E-124	442
	Gorai.003G129800	45.77	544	277	12	20	553	32	567	3.00E-124	442
	Gorai.003G096600	45.13	534	275	10	24	553	34	553	7.00E-124	441
	Gorai.009G261500	45.49	532	264	10	22	546	22	534	1.00E-123	441
	Gorai.013G025500	44.83	542	286	8	20	553	30	566	1.00E-123	440
	Gorai.005G076600	45.91	538	279	8	21	553	26	556	1.00E-123	440
	Gorai.002G148200	44.36	532	275	10	24	546	28	547	7.00E-123	437
	Gorai.010G130100	45.52	536	274	12	19	546	25	550	8.00E-123	437
	Gorai.013G036200	45.42	535	274	10	23	553	33	553	6.00E-122	434
	Gorai.007G376600	44.38	543	282	11	23	553	26	560	7.00E-122	434
	Gorai.011G101300	45.13	534	275	10	24	553	33	552	7.00E-121	431
	Gorai.012G111900	43.23	539	289	10	19	553	21	546	5.00E-119	425
	Gorai.012G109900	41.43	531	298	7	24	546	39	564	1.00E-117	421
	Gorai.011G279600	44.9	539	278	11	19	553	21	544	2.00E-116	416
	Gorai.007G378200	43.04	539	290	10	19	553	21	546	2.00E-116	416
	Gorai.003G124600	43.23	539	286	11	19	553	24	546	8.00E-115	411
	Gorai.009G093800	43.23	539	287	11	19	553	23	546	1.00E-114	410
	Gorai.002G261500	44.69	537	280	10	21	553	25	548	1.00E-114	410
	Gorai.008G126500	44.04	545	278	9	19	547	23	556	4.00E-114	409
	Gorai.009G321900	43.74	535	282	10	23	553	26	545	4.00E-114	408

	Gorai.007G110500	44.07	531	279	8	24	547	39	558	6.00E-113	405
	Gorai.007G205400	41.4	500	251	11	77	551	21	503	3.00E-96	349
	Gorai.009G103600	39	541	252	9	20	553	31	500	2.00E-95	347
	Gorai.009G323100	45.41	381	181	5	22	397	4	362	7.00E-92	335
	Gorai.013G027600	46	413	205	10	145	553	1	399	4.00E-89	325
	Gorai.010G194200	52.21	272	128	2	35	305	11	281	4.00E-80	296
	Gorai.010G194200	48.39	124	58	4	423	546	279	396	3.00E-22	103
	Gorai.003G129500	40.08	484	228	16	20	494	32	462	9.00E-80	294
	Gorai.010G194400	40.52	348	184	6	22	366	25	352	8.00E-73	271
	Gorai.013G036000	48.3	265	136	1	23	287	33	296	9.00E-68	254
	Gorai.013G036000	48.37	153	75	4	401	553	298	446	2.00E-29	127
	Gorai.009G261400	39.65	285	152	5	22	306	28	292	2.00E-53	207
	Gorai.009G261200	40.61	261	141	4	22	282	28	274	9.00E-53	205
	Gorai.009G260700	38.25	285	156	5	23	307	29	293	5.00E-52	202
	Gorai.009G261600	38.33	287	157	5	22	308	28	294	1.00E-51	201
	Gorai.008G103600	31.72	558	323	19	24	550	28	558	2.00E-51	201
	Gorai.004G227900	29.82	550	323	19	23	541	32	549	6.00E-51	199
	Gorai.009G287100	41	261	140	4	22	282	29	275	1.00E-50	198
	Gorai.003G045100	32.48	545	310	22	37	550	54	571	2.00E-49	194
	Gorai.012G109600	47.25	182	96	0	24	205	28	209	2.00E-46	184
	Gorai.009G309400	27.13	516	326	18	36	539	44	521	4.00E-33	139
	Gorai.001G048300	25.63	519	342	14	26	531	27	514	4.00E-32	136
	Gorai.009G309500	27.13	505	322	20	36	528	44	514	4.00E-32	136
	Gorai.006G212500	27.31	520	330	15	24	528	30	516	7.00E-30	129
	Gorai.010G181000	26.28	487	310	16	43	523	48	491	1.00E-29	128
	Gorai.009G268100	27.58	504	321	16	43	543	48	510	2.00E-29	127
	Gorai.008G038600	26.65	499	318	17	43	536	50	505	3.00E-28	123
	Gorai.008G236600	25.29	514	347	11	24	528	30	515	3.00E-28	123
	Gorai.001G102100	25.43	519	338	14	24	528	32	515	5.00E-28	122
	Gorai.008G073000	27.99	518	321	19	22	535	29	498	6.00E-28	122
	Gorai.009G179300	27.04	503	291	18	40	523	46	491	1.00E-27	121
	Gorai.002G120000	27.6	500	314	16	43	537	48	504	1.00E-27	121
	Gorai.002G202300	24.95	497	329	13	43	536	52	507	6.00E-27	119
	Gorai.009G189900	26.8	500	318	18	43	537	48	504	5.00E-25	112
	Gorai.008G189200	24.91	534	295	17	24	523	36	497	4.00E-24	109
	Gorai.007G052900	24.71	526	350	14	23	545	30	512	2.00E-21	101
	Gorai.013G027700	49.41	85	43	0	24	108	34	118	1.00E-20	98.6
	Gorai.007G209900	23.29	541	339	17	23	545	31	513	1.00E-20	98.2
	Gorai.007G209700	26.54	520	326	19	24	535	29	500	4.00E-19	93.6
	Gorai.011G104400	22.41	522	339	17	23	536	25	488	1.00E-18	91.3
	Gorai.013G245000	22.75	501	340	17	40	532	46	507	9.00E-18	89
	Gorai.013G244800	22.38	496	343	15	43	532	48	507	1.00E-17	88.6
	Gorai.013G244900	24.15	501	333	16	40	532	46	507	8.00E-17	85.9
	Gorai.009G194300	27.65	264	175	7	24	283	32	283	2.00E-16	84.7
	Gorai.002G173600	23.95	501	334	18	40	532	46	507	3.00E-16	84
	Gorai.004G098200	20.93	497	342	13	43	532	39	491	6.00E-15	79.3
	Gorai.013G244700	24.28	276	195	7	40	313	46	309	3.00E-13	73.9
	Gorai.006G269000	24.9	245	172	7	46	283	51	290	5.00E-13	73.2
**AtLAC16**	Gorai.003G124600	73.58	530	125	5	1	523	35	556	0	786
	Gorai.009G093800	72.08	530	134	5	1	523	34	556	0	786
	Gorai.012G111900	70.54	533	139	6	1	523	32	556	0	764
	Gorai.007G378200	69.61	533	144	6	1	523	32	556	0	751
	Gorai.009G321900	63.58	530	179	4	1	523	33	555	0	707
	Gorai.002G261500	67.29	532	158	5	1	523	34	558	0	705
	Gorai.011G279600	63.58	530	179	4	1	523	32	554	0	704
	Gorai.003G096600	59.81	530	200	4	1	523	40	563	0	671
	Gorai.011G101300	59.43	530	202	4	1	523	39	562	0	665
	Gorai.013G036200	59.06	530	204	4	1	523	40	563	0	653
	Gorai.007G376800	57.12	541	211	8	1	523	46	583	0	630
	Gorai.006G171500	58.04	541	206	8	1	523	46	583	2.00E-180	629
	Gorai.013G021400	57.51	539	211	7	1	523	40	576	2.00E-178	622
	Gorai.009G103200	55.9	542	218	7	1	523	40	579	2.00E-177	619
	Gorai.013G025500	56.48	540	215	8	1	523	40	576	2.00E-176	615
	Gorai.002G257100	53.92	536	230	5	3	523	40	573	6.00E-170	594
	Gorai.007G376600	52.79	538	235	5	3	523	35	570	5.00E-169	591
	Gorai.013G263200	53.43	539	229	8	1	523	45	577	2.00E-167	585
	Gorai.003G129800	52.32	539	238	7	1	523	42	577	6.00E-164	574
	Gorai.003G129700	52.51	537	236	7	3	523	44	577	1.00E-163	573
	Gorai.010G194600	49.07	540	252	7	2	523	34	568	5.00E-154	541
	Gorai.010G194300	49.26	540	251	7	2	523	34	568	6.00E-154	541
	Gorai.011G290000	49.72	541	248	7	2	523	34	569	2.00E-152	536

	Gorai.012G110000	50.09	535	247	5	5	523	37	567	2.00E-149	525
	Gorai.004G234200	49.72	535	246	7	6	523	48	576	3.00E-149	525
	Gorai.003G150200	49.72	535	246	7	6	523	48	576	7.00E-149	524
	Gorai.013G027600	58.94	414	164	3	110	523	2	409	1.00E-145	513
	Gorai.009G103600	48.02	529	206	7	1	521	41	508	6.00E-141	498
	Gorai.007G110500	46.93	537	256	7	5	523	49	574	2.00E-139	492
	Gorai.002G148200	48.87	532	252	7	6	523	39	564	2.00E-138	489
	Gorai.008G126500	46.46	536	258	7	6	523	48	572	7.00E-138	487
	Gorai.009G260400	48.87	532	258	4	5	523	43	573	9.00E-136	480
	Gorai.012G109900	47.75	534	261	6	6	523	50	581	1.00E-131	466
	Gorai.012G109700	46.56	524	253	8	5	506	38	556	1.00E-128	457
	Gorai.012G109500	46.58	526	250	8	5	506	38	556	5.00E-128	454
	Gorai.009G260600	42.59	533	288	8	2	523	28	553	7.00E-126	447
	Gorai.009G260800	42.7	534	288	7	2	523	33	560	2.00E-125	446
	Gorai.009G261300	42.24	535	290	8	2	523	31	559	7.00E-125	444
	Gorai.009G261100	42.13	534	290	9	2	523	37	563	8.00E-125	444
	Gorai.009G261000	42.51	534	288	9	2	523	37	563	3.00E-124	442
	Gorai.009G261500	42.24	535	282	10	2	523	31	551	8.00E-123	437
	Gorai.010G130100	42.8	535	288	9	2	523	39	568	1.00E-120	430
	Gorai.005G076600	40.53	533	302	7	3	523	37	566	9.00E-116	414
	Gorai.007G205400	43.79	507	251	8	32	512	6	504	8.00E-112	400
	Gorai.003G129500	45.32	470	192	9	1	454	42	462	1.00E-107	387
	Gorai.013G036000	60.08	258	95	2	1	251	40	296	8.00E-93	338
	Gorai.013G036000	65.63	160	54	1	364	523	298	456	4.00E-61	232
	Gorai.009G323100	43.09	376	171	6	2	360	13	362	2.00E-81	300
	Gorai.010G194200	49.63	272	130	1	2	266	7	278	8.00E-79	291
	Gorai.010G194200	60.63	127	49	1	397	523	288	413	1.00E-44	178
	Gorai.010G194400	43.07	339	161	5	2	331	34	349	1.00E-72	271
	Gorai.008G103600	31.5	527	307	15	8	501	41	546	1.00E-59	228
	Gorai.003G045100	31.95	532	298	18	8	501	54	559	2.00E-58	224
	Gorai.004G227900	32.33	529	298	20	8	501	46	549	3.00E-56	216
	Gorai.009G287100	44.03	243	130	3	2	244	38	274	2.00E-52	204
	Gorai.009G260700	42.97	249	135	4	5	253	40	281	4.00E-52	202
	Gorai.009G261200	42.5	240	132	2	5	244	40	273	2.00E-51	201
	Gorai.009G261400	42.17	249	137	4	5	253	40	281	3.00E-50	196
	Gorai.009G261600	41.37	249	139	4	5	253	40	281	7.00E-48	188
	Gorai.012G109600	46.51	172	85	1	5	169	38	209	2.00E-43	173
	Gorai.009G268100	29.75	521	313	17	16	523	50	530	3.00E-43	173
	Gorai.002G202300	29.52	481	290	15	14	483	52	494	5.00E-43	172
	Gorai.009G309400	28.25	531	338	14	7	523	44	545	3.00E-42	170
	Gorai.008G038600	28.9	481	293	14	14	483	50	492	2.00E-40	164
	Gorai.002G120000	29.92	488	291	17	14	489	48	496	3.00E-40	163
	Gorai.009G309500	27.63	532	331	16	7	516	44	543	8.00E-39	158
	Gorai.009G189900	28.95	487	295	16	14	488	48	495	2.00E-38	157
	Gorai.009G179300	29.07	485	288	16	14	483	48	491	4.00E-38	156
	Gorai.010G181000	27.51	527	322	15	14	523	48	531	2.00E-36	150
	Gorai.007G052900	27.86	499	291	17	11	489	47	496	5.00E-36	149
	Gorai.008G073000	28.33	526	311	19	13	521	49	525	8.00E-36	148
	Gorai.007G209700	28.79	528	311	18	11	521	48	527	1.00E-34	144
	Gorai.001G048300	25.9	529	352	13	11	523	41	545	2.00E-34	144
	Gorai.008G189200	29.55	494	287	17	7	483	48	497	2.00E-34	143
	Gorai.008G236600	26.68	536	339	20	11	523	46	550	8.00E-34	142
	Gorai.001G102100	27.66	517	294	17	7	488	44	515	1.00E-33	141
	Gorai.007G209900	28.4	500	291	17	7	487	44	495	3.00E-33	140
	Gorai.013G245000	26.1	498	304	18	14	489	48	503	3.00E-33	139
	Gorai.013G244800	25.9	498	305	18	14	489	48	503	5.00E-32	135
	Gorai.002G173600	25.51	494	312	17	14	489	48	503	8.00E-32	135
	Gorai.011G104400	28.04	485	291	17	11	483	42	480	1.00E-31	134
	Gorai.006G212500	27.05	536	338	20	11	523	46	551	7.00E-31	132
	Gorai.013G244900	25.4	500	305	18	14	489	48	503	4.00E-29	126
	Gorai.013G027700	61.11	90	28	1	1	83	40	129	1.00E-28	125
	Gorai.013G244700	25.25	491	285	17	14	489	48	471	3.00E-28	123
	Gorai.004G098200	25.7	498	299	18	14	489	39	487	1.00E-25	114
	Gorai.003G096200	37.33	150	55	2	1	150	31	141	1.00E-24	111
	Gorai.009G194300	32.81	253	150	7	7	249	44	286	4.00E-23	106
**AtLAC17**	Gorai.009G103200	81.93	559	96	3	18	576	25	578	0	924
	Gorai.013G021400	81.04	559	98	3	18	576	25	575	0	920
	Gorai.013G025500	79.43	559	107	3	18	576	25	575	0	910
	Gorai.003G129800	73.74	556	135	3	22	576	31	576	0	840
	Gorai.003G129700	73.02	556	139	3	22	576	31	576	0	835
	Gorai.013G263200	72.48	556	139	5	22	576	34	576	0	818

Gorai.002G257100	71.27	557	151	2	20	576	25	572	0	807
Gorai.007G376600	69.66	557	162	3	20	576	20	569	0	806
Gorai.006G171500	70.45	555	157	3	22	576	35	582	0	801
Gorai.007G376800	70.09	555	159	3	22	576	35	582	0	791
Gorai.009G103600	62.97	559	132	6	17	575	25	508	0	662
Gorai.012G111900	58.88	552	207	5	25	576	24	555	0	638
Gorai.007G378200	57.61	552	214	5	25	576	24	555	0	631
Gorai.003G096600	57.5	560	213	7	18	575	25	561	1.00E-180	630
Gorai.011G101300	57.25	559	214	7	19	575	25	560	9.00E-180	627
Gorai.011G279600	56.52	552	218	6	25	576	24	553	8.00E-179	624
Gorai.009G093800	57.58	554	213	7	23	576	24	555	1.00E-178	623
Gorai.003G124600	56.86	554	216	6	23	576	25	555	3.00E-177	618
Gorai.013G036200	54.66	558	230	6	19	575	26	561	2.00E-173	606
Gorai.002G261500	57.61	552	214	5	25	576	26	557	4.00E-173	605
Gorai.003G129500	64.75	488	115	6	22	508	31	462	1.00E-170	596
Gorai.009G321900	53.44	552	235	5	25	576	25	554	2.00E-168	589
Gorai.004G234200	51.9	553	251	7	26	576	36	575	1.00E-152	536
Gorai.010G194300	50.63	559	254	12	23	575	24	566	2.00E-151	533
Gorai.010G194600	50.27	559	256	12	23	575	24	566	4.00E-150	528
Gorai.011G290000	50.63	559	258	10	22	575	22	567	2.00E-148	523
Gorai.003G150200	50.63	557	242	9	31	576	41	575	5.00E-148	521
Gorai.012G110000	48.84	559	258	9	26	575	26	565	2.00E-144	509
Gorai.008G126500	46.28	564	276	9	19	576	29	571	9.00E-138	487
Gorai.009G260400	48.4	562	274	8	18	576	24	572	1.00E-135	480
Gorai.007G110500	45.74	564	279	9	19	576	31	573	8.00E-135	477
Gorai.002G148200	46.21	554	272	8	30	576	29	563	2.00E-132	469
Gorai.012G109500	46.88	544	264	8	27	560	28	556	5.00E-129	458
Gorai.012G109900	46.03	554	283	5	27	576	39	580	1.00E-128	456
Gorai.012G109700	46.07	547	270	7	24	560	25	556	5.00E-128	455
Gorai.013G027600	55.84	428	166	7	149	575	2	407	9.00E-123	437
Gorai.009G260600	41.59	553	301	8	26	576	20	552	3.00E-119	426
Gorai.007G205400	46.34	505	230	11	80	565	21	503	2.00E-118	423
Gorai.009G261300	39.75	556	312	8	25	576	22	558	9.00E-115	410
Gorai.009G261100	40.18	555	307	7	26	576	29	562	3.00E-113	405
Gorai.009G261000	40.54	555	305	8	26	576	29	562	1.00E-111	400
Gorai.009G261500	39.21	556	307	10	25	576	22	550	6.00E-111	398
Gorai.010G130100	38.66	551	319	8	24	572	29	562	2.00E-108	389
Gorai.009G260800	38.56	555	317	8	26	576	25	559	6.00E-108	388
Gorai.005G076600	38.78	557	316	10	25	575	27	564	2.00E-105	380
Gorai.013G036000	57.56	271	113	1	19	289	26	294	3.00E-98	356
Gorai.013G036000	65.16	155	53	1	422	575	300	454	4.00E-48	189
Gorai.009G323100	46.23	398	174	8	22	415	1	362	4.00E-96	348
Gorai.010G194200	53.24	278	127	2	30	306	3	278	2.00E-88	323
Gorai.010G194200	31.27	371	213	9	212	575	76	411	9.00E-33	138
Gorai.010G194400	44.13	358	166	8	23	378	24	349	1.00E-79	294
Gorai.008G103600	32.18	550	317	19	25	551	26	542	1.00E-55	214
Gorai.004G227900	29.59	561	317	20	24	551	30	545	2.00E-51	200
Gorai.003G045100	30.42	549	328	18	25	551	39	555	2.00E-51	200
Gorai.012G109600	50.57	174	86	0	36	209	37	210	2.00E-49	194
Gorai.009G261200	35.82	282	160	7	25	306	28	288	2.00E-45	181
Gorai.009G260700	35.88	262	153	4	26	287	29	275	3.00E-45	179
Gorai.009G261400	34.88	281	162	5	26	306	29	288	2.00E-43	174
Gorai.009G287100	35.5	262	154	4	26	287	30	276	2.00E-43	173
Gorai.009G261600	36.02	261	152	5	26	286	29	274	2.00E-41	167
Gorai.013G027700	58.65	104	43	0	19	122	26	129	1.00E-35	147
Gorai.009G309400	27.34	545	341	17	39	574	44	542	8.00E-35	145
Gorai.008G236600	27.85	535	334	17	27	551	30	522	1.00E-30	131
Gorai.009G309500	26.87	547	338	23	39	570	44	543	4.00E-28	123
Gorai.006G212500	28.04	535	334	18	27	551	30	523	8.00E-28	122
Gorai.001G048300	25.97	516	340	14	29	537	25	505	8.00E-28	122
Gorai.009G268100	27.55	265	173	6	25	285	27	276	4.00E-25	113
Gorai.001G102100	26.19	527	335	19	27	542	32	515	3.00E-24	110
Gorai.002G202300	27.92	265	172	6	25	285	31	280	1.00E-23	108
Gorai.002G120000	27.72	267	178	6	25	286	27	283	4.00E-23	106
Gorai.009G194300	28.68	265	178	5	27	290	32	286	6.00E-23	105
Gorai.008G038600	26.04	265	177	6	25	285	29	278	1.00E-22	105
Gorai.009G179300	24.81	262	184	5	25	285	27	276	1.00E-22	105
Gorai.009G189900	26.22	267	184	6	25	290	27	281	1.00E-22	104
Gorai.013G244800	24.12	514	316	17	46	543	48	503	4.00E-22	103
Gorai.013G245000	23.93	514	317	17	46	543	48	503	4.00E-22	103
Gorai.010G181000	25.1	247	174	4	25	270	27	263	5.00E-22	102
Gorai.008G073000	25.52	286	191	6	25	307	29	295	1.00E-21	101

	Gorai.003G096200	31.4	172	71	2	19	189	16	141	1.00E-20	98.6
	Gorai.002G173600	23.09	511	325	14	46	543	48	503	6.00E-20	96.3
	Gorai.007G209900	24.71	522	330	16	26	541	31	495	6.00E-20	95.9
	Gorai.013G244900	24.75	497	322	17	46	537	48	497	2.00E-19	94.4
	Gorai.008G189200	25.74	272	183	6	25	292	34	290	3.00E-19	94
	Gorai.007G052900	26.23	244	165	6	43	285	47	276	4.00E-19	93.2
	Gorai.011G104400	24.9	261	181	6	26	285	25	271	7.00E-17	85.9
	Gorai.013G244700	24.82	274	189	8	46	317	48	306	1.00E-16	85.5
	Gorai.007G209700	26.24	282	189	6	27	307	32	295	7.00E-16	82.8
	Gorai.004G098200	25.48	263	182	8	46	306	39	289	7.00E-14	75.9

Supplementary Table S1: Details of BlastP similarity search perform to identify the cotton laccases using Arabidopsis laccase proteins as query.
After the validation with conserve domain analysis, protein identified as laccases are highlighted with red text.

Query id	Subject id	% identity	alignmen	mismatch	gap open	q. start	q. end	s. start	s. end	e-value	bit score
AtLAC01	Gh_D13G0231	52.96	557	248	6	28	581	31	576	6.00E-164	575
	Gh_A13G0216	51.89	557	254	6	28	581	31	576	3.00E-163	573
	Gh_D10G0895	51.96	562	245	8	22	581	24	562	3.00E-163	572
	Gh_A09G1445	50.36	558	268	3	25	581	34	583	5.00E-163	572
	Gh_D09G1454	50.72	558	266	3	25	581	34	583	6.00E-163	572
	Gh_A05G0849	52.42	557	254	6	28	581	31	579	1.00E-162	571
	Gh_A10G0858	51.96	562	245	8	22	581	24	562	2.00E-162	570
	Gh_D13G0194	52.24	557	252	6	28	581	31	576	5.00E-162	568
	Gh_D05G3888	52.6	557	253	6	28	581	31	579	1.00E-161	567
	Gh_D11G3307	49.82	560	268	5	25	581	34	583	1.00E-160	564
	Gh_A11G2922	49.46	560	270	5	25	581	34	583	1.00E-159	561
	Gh_D01G2166	52.33	558	249	6	28	581	29	573	3.00E-159	559
	Gh_D03G1181	51.7	559	251	8	28	581	33	577	2.00E-158	556
	Gh_A13G1977	51.78	562	251	7	24	581	32	577	4.00E-158	556
	Gh_D03G1180	51.88	559	250	8	28	581	33	577	8.00E-158	555
	Gh_D11G3305	52.58	563	251	9	23	581	19	569	1.00E-157	554
	Gh_A11G2920	52.4	563	253	9	23	581	19	570	4.00E-157	552
	Gh_A01G1905	52.15	558	250	7	28	581	29	573	4.00E-157	552
	Gh_A03G2082	51.16	559	254	8	28	581	33	577	1.00E-156	551
	Gh_D13G2373	50.71	562	257	7	24	581	32	577	6.00E-156	548
	Gh_A13G0237	49.64	562	258	8	22	581	25	563	1.00E-155	547
	Gh_D13G0253	49.47	562	259	8	22	581	25	563	2.00E-154	543
	Gh_A03G0583	49.19	559	259	8	24	581	27	561	2.00E-151	534
	Gh_A13G0179	47.4	557	231	7	28	581	31	528	7.00E-150	528
	Gh_D04G1243	48.29	557	264	8	27	581	22	556	5.00E-149	525
	Gh_D03G0865	48.84	559	257	9	24	581	27	557	2.00E-147	520
	Gh_D05G0888	49.02	563	261	9	21	581	18	556	3.00E-147	519
	Gh_A05G0758	48.85	563	262	9	21	581	18	556	9.00E-147	518
	Gh_A11G2936	47.76	557	267	8	27	581	22	556	9.00E-147	518
	Gh_D11G3322	47.57	555	267	8	29	581	24	556	2.00E-146	516
	Gh_D03G1128	48.85	563	261	10	21	581	19	556	4.00E-144	509
	Gh_A06G1415	48.32	565	271	7	23	581	19	568	2.00E-143	506
	Gh_A05G2622	49.11	564	259	10	21	581	17	555	4.00E-143	506
	Gh_D06G1762	48.32	565	271	7	23	581	19	568	6.00E-143	505
	Gh_D03G1367	50	566	259	13	23	581	28	576	1.00E-142	504
	Gh_D01G2209	47.58	559	267	8	26	581	23	558	2.00E-142	504
	Gh_A01G1948	47.76	559	266	8	26	581	23	558	2.00E-142	503
	Gh_D10G2466	46.83	583	256	9	29	581	24	582	2.00E-142	503
	Gh_D05G2912	48.49	563	264	9	21	581	17	555	8.00E-142	501
	Gh_A03G0417	48.31	563	264	10	21	581	19	556	9.00E-142	501
	Gh_A03G2057	49.12	566	264	12	23	581	28	576	2.00E-140	496
	Gh_A06G1413	47.28	569	271	7	23	581	19	568	3.00E-140	496
	Gh_D10G2461	48.93	562	261	12	29	581	25	569	2.00E-139	493
	Gh_A10G2140	48.04	560	269	8	29	581	25	569	2.00E-138	490
	Gh_A08G2350	49.82	558	252	12	33	581	39	577	4.00E-138	489
	Gh_D08G2159	50	558	251	12	33	581	39	577	6.00E-138	488
	Gh_D04G1224	46.59	558	270	9	33	581	29	567	2.00E-133	473
	Gh_A04G0744	46.59	558	270	9	33	581	29	567	3.00E-133	473
	Gh_A03G2084	48.64	516	233	8	28	539	33	520	7.00E-130	462
	Gh_D05G2353	46.13	555	288	7	30	581	32	578	3.00E-128	456
	Gh_A05G2099	45.95	555	285	6	30	581	32	574	5.00E-128	456
	Gh_A13G2215	46.58	556	269	10	33	581	30	564	2.00E-126	450
	Gh_A05G0853	44.76	563	233	9	21	581	25	511	2.00E-126	450
	Gh_D11G1042	43.66	568	289	10	22	581	30	574	2.00E-125	447
	Gh_D13G2524	46.4	556	270	10	33	581	30	564	2.00E-125	447
	Gh_A12G1019	42.78	589	285	10	23	581	29	595	1.00E-123	441
	Gh_D12G1138	42.54	590	287	10	22	581	28	595	2.00E-123	441
	Gh_A05G2631	44.77	554	252	10	35	581	4	510	6.00E-121	432
	Gh_D11G1874	44.61	538	278	10	34	564	13	537	3.00E-118	423
	Gh_D04G1223	44.5	555	288	8	33	581	41	581	1.00E-117	421

	Gh_D04G1221	44.04	554	277	12	25	564	22	556	3.00E-117	420
	Gh_A04G0743	44.31	562	282	11	33	581	30	573	7.00E-117	419
	Gh_Sca005020G01	41.89	561	267	9	21	579	25	528	6.00E-114	409
	Gh_A06G0997	42.11	551	297	8	35	581	36	568	1.00E-111	401
	Gh_D06G1188	42.16	555	299	10	31	581	32	568	2.00E-111	400
	Gh_D05G2354	41.49	564	304	10	22	581	21	562	1.00E-110	398
	Gh_D05G2356	40.57	562	309	9	25	581	18	559	1.00E-109	394
	Gh_A02G0638	38.45	554	321	6	31	581	29	565	3.00E-108	390
	Gh_A05G2115	40.07	564	309	12	24	581	23	563	5.00E-107	386
	Gh_A05G2113	39.79	563	313	10	25	581	18	560	7.00E-106	382
	Gh_A05G2117	39.65	565	309	11	25	581	20	560	1.00E-105	381
	Gh_A05G2116	40.54	555	305	11	31	581	30	563	1.00E-105	381
	Gh_D03G1177	45.06	506	207	11	31	528	34	476	2.00E-105	380
	Gh_A13G2103	49.07	428	184	7	167	581	90	496	1.00E-104	378
	Gh_A13G2103	46.77	62	33	0	26	87	29	90	2.00E-11	68.9
	Gh_D02G0685	37.84	555	324	7	31	581	29	566	1.00E-103	375
	Gh_A13G2102	42.3	513	220	8	26	538	29	465	2.00E-100	364
	Gh_A05G2100	41	522	282	11	22	539	21	520	1.00E-94	345
	Gh_A13G0218	52.82	284	129	3	28	308	31	312	2.00E-89	327
	Gh_A13G0218	51.56	128	60	1	412	539	309	434	1.00E-21	102
	Gh_D13G2551	56.88	269	115	1	26	294	29	296	6.00E-89	326
	Gh_D13G2551	59.49	158	63	1	424	581	300	456	2.00E-46	184
	Gh_D06G2354	46.28	376	185	5	210	581	11	373	5.00E-79	293
	Gh_D05G2921	40.62	389	193	4	35	419	4	358	6.00E-79	293
	Gh_A11G0894	40.88	362	191	6	25	383	27	368	3.00E-78	290
	Gh_D06G1759	42.58	364	179	4	23	386	19	352	2.00E-75	281
	Gh_A11G1716	50.18	277	129	6	55	326	1	273	1.00E-68	258
	Gh_D13G0232	46.83	331	157	5	210	539	12	324	5.00E-68	256
	Gh_D13G0328	55.98	184	81	0	26	209	29	212	8.00E-58	222
	Gh_D13G0328	65.81	117	40	0	465	581	211	327	2.00E-34	144
	Gh_D12G0913	31.5	581	328	21	22	571	19	560	8.00E-51	199
	Gh_A12G0840	31.33	581	330	21	22	571	19	561	2.00E-50	198
	Gh_A02G1272	31.45	585	310	23	29	571	39	574	2.00E-50	197
	Gh_D03G0412	31.3	575	324	21	29	571	39	574	5.00E-50	196
	Gh_A08G1751	29.27	591	325	22	23	571	25	564	8.00E-49	192
	Gh_D08G2100	29.27	591	325	22	23	571	25	564	4.00E-48	190
	Gh_A06G1412	52.75	182	83	2	401	581	1	180	5.00E-42	170
	Gh_D05G2355	35.32	269	156	6	24	290	23	275	3.00E-41	167
	Gh_A05G2114	35.32	269	156	6	24	290	23	275	3.00E-41	167
**AtLAC02**	Gh_D03G1181	73.72	567	147	2	8	573	12	577	0	866
	Gh_A03G2082	72.84	567	152	2	8	573	12	577	0	858
	Gh_D03G1180	72.43	573	155	3	2	573	7	577	0	857
	Gh_A05G0849	71.28	571	156	3	9	573	11	579	0	845
	Gh_A13G1977	72.15	571	153	6	5	573	11	577	0	845
	Gh_D05G3888	71.45	571	155	3	9	573	11	579	0	843
	Gh_D13G2373	72.13	567	152	6	9	573	15	577	0	842
	Gh_D13G0194	71	569	159	4	8	573	11	576	0	838
	Gh_D13G0231	72	550	149	3	27	573	29	576	0	830
	Gh_A13G0216	71.27	550	153	3	27	573	29	576	0	829
	Gh_A11G2920	68.17	553	170	3	25	573	20	570	0	809
	Gh_D01G2166	67.67	566	177	3	9	573	13	573	0	808
	Gh_A01G1905	66.55	565	187	2	10	573	10	573	0	793
	Gh_D11G3305	67.09	553	175	4	25	573	20	569	0	791
	Gh_D09G1454	65.44	570	191	3	8	573	16	583	0	779
	Gh_A09G1445	65.57	575	190	4	5	573	11	583	0	777
	Gh_D11G3307	66.85	555	178	3	23	573	31	583	0	773
	Gh_A11G2922	66.13	555	182	3	23	573	31	583	0	766
	Gh_A13G0179	64.91	550	140	4	27	573	29	528	0	752
	Gh_A03G2084	69.9	525	141	5	8	531	12	520	0	738
	Gh_A05G0853	58.76	548	161	5	26	573	29	511	0	633
	Gh_D03G1128	55.67	573	235	8	1	573	3	556	8.00E-179	624
	Gh_D04G1243	56.54	566	227	8	9	573	9	556	1.00E-178	624
	Gh_D05G0888	55.65	575	234	10	1	573	1	556	2.00E-178	623

Gh_A05G0758	55.65	575	234	10	1	573	1	556	3.00E-178	623
Gh_D11G3322	55.93	565	232	8	9	573	9	556	3.00E-178	623
Gh_A03G0417	55.75	574	233	9	1	573	3	556	8.00E-178	621
Gh_A11G2936	54.69	565	239	7	9	573	9	556	2.00E-175	613
Gh_A13G0237	53.52	568	248	8	7	573	11	563	2.00E-175	613
Gh_D10G0895	53.97	567	241	9	9	573	14	562	3.00E-175	613
Gh_D03G1177	62.28	509	137	6	22	525	23	481	5.00E-175	612
Gh_D13G0253	53.35	568	249	8	7	573	11	563	1.00E-174	611
Gh_A10G0858	53.53	566	245	8	9	573	14	562	2.00E-174	609
Gh_Sca005020G01	57.87	553	173	7	26	571	29	528	8.00E-174	608
Gh_D10G2466	51.58	601	244	8	1	573	1	582	2.00E-173	607
Gh_A05G2622	52.62	572	252	7	2	573	3	555	8.00E-171	598
Gh_D05G2912	52.27	572	254	7	2	573	3	555	4.00E-170	595
Gh_D01G2209	54.45	573	246	7	1	573	1	558	9.00E-170	594
Gh_A03G0583	52.27	572	251	11	7	573	7	561	6.00E-169	592
Gh_A01G1948	54.45	573	246	7	1	573	1	558	8.00E-169	591
Gh_D03G0865	51.75	572	250	12	7	573	7	557	3.00E-164	576
Gh_D06G1762	51.39	576	251	10	12	573	8	568	4.00E-162	569
Gh_A06G1415	51.22	576	252	10	12	573	8	568	9.00E-162	568
Gh_A06G1413	51.05	570	262	9	12	573	8	568	1.00E-159	561
Gh_D03G1367	51.3	577	250	10	9	573	19	576	3.00E-159	559
Gh_A03G2057	51.47	577	249	10	9	573	19	576	1.00E-158	558
Gh_A08G2350	50.44	573	269	8	8	573	13	577	5.00E-158	555
Gh_D10G2461	51.23	570	261	8	13	573	8	569	1.00E-157	554
Gh_D08G2159	51.45	550	252	8	31	573	36	577	4.00E-155	545
Gh_A10G2140	50	576	270	9	7	573	3	569	5.00E-155	545
Gh_D04G1224	48.56	556	272	7	24	573	20	567	1.00E-147	521
Gh_A04G0744	48.56	556	272	7	24	573	20	567	8.00E-147	518
Gh_A13G2215	49.64	548	261	6	31	573	27	564	3.00E-143	506
Gh_D13G2524	49.37	553	264	7	26	573	23	564	2.00E-142	503
Gh_A05G2631	49.18	551	223	11	36	573	4	510	4.00E-141	499
Gh_A04G0743	47.14	577	284	8	11	573	4	573	2.00E-137	487
Gh_A13G0218	75.34	292	72	0	27	318	29	320	2.00E-136	484
Gh_A13G0218	69.84	126	38	0	406	531	309	434	9.00E-43	172
Gh_D04G1223	46.23	571	295	6	11	573	15	581	4.00E-135	479
Gh_A05G2099	49.64	550	263	8	31	573	32	574	7.00E-134	475
Gh_D04G1221	45.26	559	287	7	11	556	4	556	2.00E-132	470
Gh_D05G2353	48.56	554	267	8	31	573	32	578	3.00E-132	470
Gh_D11G1042	43.97	580	297	11	9	573	8	574	8.00E-129	458
Gh_D12G1138	44.66	580	276	12	25	573	30	595	8.00E-129	458
Gh_A12G1019	44.05	597	288	13	8	573	14	595	1.00E-128	457
Gh_D05G2354	41.11	574	317	9	2	573	8	562	3.00E-122	436
Gh_D11G1874	43.89	540	268	11	36	556	14	537	8.00E-119	425
Gh_D05G2356	39.17	577	329	9	1	573	1	559	3.00E-118	423
Gh_A05G2113	39.86	577	326	10	1	573	1	560	3.00E-117	419
Gh_A05G2115	39.76	576	325	9	1	573	7	563	4.00E-117	419
Gh_A05G2116	41.06	548	305	10	31	573	29	563	2.00E-116	417
Gh_D13G0232	66.36	324	95	4	211	531	12	324	4.00E-114	409
Gh_A05G2117	38.82	577	330	11	1	573	3	560	2.00E-113	407
Gh_D06G1188	38.26	575	337	9	2	573	9	568	4.00E-112	403
Gh_A06G0997	38.02	576	337	10	2	573	9	568	7.00E-111	399
Gh_A13G2102	43.75	528	221	12	5	530	12	465	2.00E-108	390
Gh_A02G0638	39.09	573	325	11	9	573	9	565	3.00E-108	390
Gh_A05G2100	40.6	532	295	10	2	531	8	520	7.00E-108	389
Gh_A13G2103	49.52	420	185	7	168	573	90	496	2.00E-105	380
Gh_A13G2103	49.41	85	36	3	5	88	12	90	2.00E-14	79
Gh_D02G0685	38.7	553	317	11	30	573	27	566	9.00E-104	375
Gh_D05G2921	48.43	382	162	7	36	411	4	356	2.00E-97	354
Gh_D13G2551	55.59	286	124	2	9	294	12	294	3.00E-95	347
Gh_D13G2551	57.05	156	66	1	419	573	301	456	4.00E-45	180
Gh_D06G2354	49.34	377	163	9	211	573	11	373	1.00E-88	325
Gh_D06G1759	45.55	371	172	7	12	378	8	352	1.00E-85	315
Gh_A11G0894	44.63	354	188	4	22	375	23	368	1.00E-83	308

	Gh_D13G0328	60.96	187	72	1	24	210	27	212	9.00E-68	255
	Gh_D13G0328	62.73	110	41	0	464	573	218	327	1.00E-40	165
	Gh_A11G1716	54.55	220	100	0	57	276	2	221	2.00E-67	254
	Gh_D12G0913	31.34	552	319	17	30	552	26	546	4.00E-54	210
	Gh_Sca030590G01	73.2	153	39	2	380	531	1	152	8.00E-54	209
	Gh_D03G0412	30.09	575	330	19	9	547	17	555	1.00E-53	209
	Gh_A08G1751	31.3	591	331	21	11	563	11	564	3.00E-53	207
	Gh_A02G1272	29.49	573	335	17	9	547	18	555	4.00E-53	207
	Gh_D08G2100	31.64	572	319	20	11	547	11	545	5.00E-53	206
	Gh_A12G0840	30.95	559	313	20	30	552	26	547	1.00E-52	205
	Gh_D04G1220	50.26	191	90	2	11	197	4	193	8.00E-50	196
	Gh_D05G2355	36.59	276	154	5	20	292	18	275	9.00E-48	189
	Gh_A05G2114	36.59	276	154	5	20	292	18	275	1.00E-47	189
**AtLAC03**	Gh_A03G2057	76.61	543	126	1	28	570	35	576	0	885
	Gh_D03G1367	77.02	544	122	2	28	570	35	576	0	884
	Gh_A08G2350	74.63	544	136	2	28	570	35	577	0	857
	Gh_D08G2159	74.08	544	139	2	28	570	35	577	0	854
	Gh_A10G2140	68.25	548	170	3	26	570	23	569	0	783
	Gh_D10G2461	68.43	548	169	3	26	570	23	569	0	781
	Gh_A06G1415	66.36	547	181	3	26	570	23	568	0	767
	Gh_D06G1762	66.18	547	182	3	26	570	23	568	0	766
	Gh_A06G1413	65.63	547	185	3	26	570	23	568	0	759
	Gh_A05G2631	62.34	539	169	5	34	570	4	510	0	685
	Gh_D10G0895	51.56	545	245	5	30	570	33	562	2.00E-165	580
	Gh_A10G0858	51.56	545	245	5	30	570	33	562	1.00E-164	577
	Gh_A09G1445	48.45	547	273	4	30	570	40	583	1.00E-163	574
	Gh_A13G0237	51.93	545	243	6	30	570	34	563	2.00E-163	573
	Gh_D09G1454	48.45	547	273	4	30	570	40	583	3.00E-163	573
	Gh_D13G0194	51.37	549	253	9	30	570	34	576	5.00E-163	572
	Gh_D13G0253	51.56	545	245	6	30	570	34	563	7.00E-163	572
	Gh_A11G2922	48.99	547	270	5	30	570	40	583	8.00E-163	571
	Gh_D11G3307	48.99	547	270	5	30	570	40	583	1.00E-162	571
	Gh_A13G1977	52.01	548	247	7	30	570	39	577	1.00E-162	570
	Gh_D05G3888	51.45	550	254	8	30	570	34	579	2.00E-162	570
	Gh_D04G1224	51.36	551	248	6	30	570	27	567	2.00E-162	570
	Gh_D13G0231	51.37	549	253	9	30	570	34	576	2.00E-161	566
	Gh_A05G0849	51.09	550	256	8	30	570	34	579	4.00E-161	565
	Gh_D13G2373	51.82	548	248	7	30	570	39	577	1.00E-160	564
	Gh_A13G0216	50.09	549	260	9	30	570	34	576	3.00E-160	562
	Gh_D04G1243	49.72	545	256	5	30	570	26	556	4.00E-160	562
	Gh_A04G0744	50.45	551	253	6	30	570	27	567	8.00E-160	561
	Gh_D03G1180	51.45	550	250	9	30	570	36	577	4.00E-159	559
	Gh_D05G0888	50.28	545	251	7	30	570	28	556	4.00E-158	555
	Gh_A13G2215	52.17	552	238	8	30	570	28	564	6.00E-158	555
	Gh_D11G3322	49.91	545	255	5	30	570	26	556	7.00E-158	555
	Gh_D03G1181	51.09	550	252	9	30	570	36	577	2.00E-157	553
	Gh_A05G0758	49.72	545	254	7	30	570	28	556	6.00E-157	551
	Gh_D13G2524	51.63	552	241	8	30	570	28	564	1.00E-156	551
	Gh_D01G2166	51.3	540	248	9	39	570	41	573	1.00E-156	550
	Gh_A11G2936	50.37	534	247	5	41	570	37	556	2.00E-156	550
	Gh_A11G2920	49.45	548	266	7	30	570	27	570	5.00E-156	548
	Gh_A03G2082	50.55	550	255	9	30	570	36	577	1.00E-155	547
	Gh_A03G0583	51.28	546	243	8	30	570	34	561	2.00E-155	546
	Gh_D03G1128	49.91	545	252	7	30	570	29	556	1.00E-154	544
	Gh_D04G1223	51.81	552	246	7	30	570	39	581	1.00E-154	544
	Gh_A03G0417	49.54	545	254	7	30	570	29	556	9.00E-154	541
	Gh_D11G3305	49.82	548	263	8	30	570	27	569	1.00E-153	541
	Gh_D01G2209	50.46	545	252	5	30	570	28	558	2.00E-153	540
	Gh_A01G1905	50.93	540	250	9	39	570	41	573	3.00E-153	540
	Gh_A01G1948	50.64	545	251	6	30	570	28	558	6.00E-153	538
	Gh_A04G0743	50.27	557	250	7	30	570	28	573	7.00E-153	538
	Gh_A05G2099	48.55	550	266	8	30	570	33	574	3.00E-152	536
	Gh_D03G0865	50.92	546	241	9	30	570	34	557	6.00E-152	535

	Gh_D04G1221	50.46	541	239	8	30	553	28	556	3.00E-149	526
	Gh_D05G2353	48.55	550	270	8	30	570	33	578	6.00E-149	525
	Gh_D11G1042	48.26	547	266	7	30	570	39	574	9.00E-149	525
	Gh_D10G2466	45.72	573	263	8	30	570	26	582	2.00E-146	517
	Gh_D12G1138	45.61	570	270	8	30	570	37	595	2.00E-144	510
	Gh_A12G1019	45.61	570	270	8	30	570	37	595	2.00E-144	510
	Gh_A05G2622	46.61	545	271	6	30	570	27	555	1.00E-143	507
	Gh_D05G2912	46.24	545	273	6	30	570	27	555	3.00E-143	506
	Gh_A13G0179	46.27	549	233	10	30	570	34	528	2.00E-137	487
	Gh_A03G2084	49.8	508	223	11	30	528	36	520	2.00E-135	480
	Gh_D06G2354	63.84	365	129	3	208	570	10	373	2.00E-134	477
	Gh_D05G2354	44	550	290	5	26	570	26	562	3.00E-134	476
	Gh_D11G1874	44.13	537	272	7	39	559	19	543	1.00E-131	468
	Gh_A02G0638	42.42	554	297	8	26	570	25	565	6.00E-130	462
	Gh_D05G2356	41.74	551	300	7	28	570	22	559	7.00E-130	462
	Gh_A05G0853	44.14	546	231	8	30	570	35	511	4.00E-129	459
	Gh_A05G2113	41.49	552	301	7	28	570	22	560	4.00E-128	456
	Gh_A05G2117	41.56	551	300	7	28	570	24	560	7.00E-127	452
	Gh_D02G0685	41.98	555	299	9	26	570	25	566	2.00E-126	450
	Gh_A05G2115	41.41	553	297	10	28	570	28	563	8.00E-126	448
	Gh_D06G1188	41.27	550	305	8	28	570	30	568	1.00E-124	444
	Gh_A06G0997	40.91	550	307	8	28	570	30	568	5.00E-124	442
	Gh_A05G2116	41.12	552	300	7	28	570	28	563	6.00E-124	442
	Gh_D05G2921	58.16	380	133	5	33	410	3	358	4.00E-122	436
	Gh_Sca005020G01	42.81	549	249	10	30	568	35	528	4.00E-121	432
	Gh_A05G2100	43.68	506	267	5	28	528	28	520	1.00E-118	424
	Gh_D03G1177	45.56	496	201	12	39	522	43	481	4.00E-111	399
	Gh_A13G2103	49.76	422	180	6	166	570	90	496	1.00E-107	388
	Gh_D06G1759	56.48	347	130	5	26	371	23	349	2.00E-107	387
	Gh_A13G2102	43.03	502	212	7	30	527	34	465	5.00E-105	379
	Gh_D13G2551	55.89	263	116	0	30	292	34	296	8.00E-87	319
	Gh_D13G2551	58.6	157	65	0	414	570	300	456	9.00E-53	206
	Gh_A13G0218	51.17	299	140	3	30	326	34	328	2.00E-86	317
	Gh_A13G0218	51.18	127	60	2	403	528	309	434	1.00E-32	138
	Gh_A11G0894	43.44	343	186	2	30	371	33	368	4.00E-83	306
	Gh_A06G1412	70	180	54	0	391	570	1	180	3.00E-75	280
	Gh_D13G0232	46.79	327	151	10	210	528	13	324	2.00E-74	277
	Gh_A11G1716	47.45	255	134	0	55	309	2	256	6.00E-64	243
	Gh_D08G2100	30.49	551	310	20	43	557	46	559	1.00E-60	232
	Gh_D03G0412	31.97	538	302	16	43	548	54	559	2.00E-60	231
	Gh_A08G1751	30.87	541	302	20	43	548	46	549	2.00E-60	231
	Gh_A02G1272	31.78	538	303	16	43	548	54	559	4.00E-60	230
	Gh_D12G0913	33.15	537	296	19	43	548	41	545	1.00E-59	228
	Gh_A12G0840	32.9	538	297	19	43	548	41	546	3.00E-59	227
	Gh_D13G0328	55.62	178	79	0	30	207	34	211	8.00E-57	219
	Gh_D13G0328	59.69	129	52	0	442	570	199	327	1.00E-43	175
	Gh_A11G0895	56.28	183	74	3	391	570	1	180	4.00E-53	207
	Gh_D04G1220	55.09	167	75	0	30	196	28	194	8.00E-51	199
	Gh_D05G2589	36.84	285	161	6	26	310	27	292	2.00E-50	198
	Gh_A05G2114	39.23	260	145	4	28	287	28	274	7.00E-50	196
**AtLAC04**	Gh_D11G3322	80.22	541	106	1	19	558	16	556	0	909
	Gh_A11G2936	79.67	541	109	1	19	558	16	556	0	903
	Gh_D04G1243	79.11	541	112	1	19	558	16	556	0	894
	Gh_D03G1128	78.33	540	115	1	19	558	19	556	0	884
	Gh_A03G0417	78.15	540	116	1	19	558	19	556	0	878
	Gh_D05G0888	76.89	541	122	2	19	558	18	556	0	873
	Gh_A05G0758	76.52	541	124	2	19	558	18	556	0	867
	Gh_A01G1948	75.97	541	129	1	19	558	18	558	0	832
	Gh_D01G2209	75.42	541	132	1	19	558	18	558	0	829
	Gh_A05G2622	66.31	555	184	2	5	558	3	555	0	766
	Gh_D05G2912	65.95	555	186	2	5	558	3	555	0	760
	Gh_D10G2466	62.26	583	189	2	5	558	2	582	0	748
	Gh_D10G0895	62.59	540	198	3	21	558	25	562	0	731

Gh_A10G0858	62.59	540	198	3	21	558	25	562	0	729
Gh_A13G0237	61.3	540	205	3	21	558	26	563	0	716
Gh_D13G0253	60.74	540	208	3	21	558	26	563	0	710
Gh_A03G0583	61.44	542	201	5	20	558	25	561	0	706
Gh_D03G0865	60.81	541	202	5	20	558	25	557	0	696
Gh_D11G3307	59.86	553	208	5	20	558	31	583	0	686
Gh_A11G2922	59.67	553	209	5	20	558	31	583	0	684
Gh_D09G1454	59.31	553	211	5	20	558	31	583	0	681
Gh_A09G1445	58.95	553	213	5	20	558	31	583	0	678
Gh_D13G0194	58.62	551	215	5	21	558	26	576	0	664
Gh_A13G0216	58.08	551	218	5	21	558	26	576	0	662
Gh_A05G0849	57.04	554	222	5	21	558	26	579	0	661
Gh_D13G0231	58.62	551	215	5	21	558	26	576	0	661
Gh_D05G3888	57.4	554	220	5	21	558	26	579	0	660
Gh_D13G2373	56.35	543	228	5	25	558	35	577	0	645
Gh_A13G1977	56.17	543	229	5	25	558	35	577	0	640
Gh_A11G2920	56.23	546	225	5	27	558	25	570	5.00E-180	628
Gh_D03G1180	55.35	551	234	6	20	558	27	577	5.00E-178	622
Gh_D03G1181	54.81	551	237	6	20	558	27	577	1.00E-177	620
Gh_D01G2166	55.56	549	232	6	22	558	25	573	1.00E-177	620
Gh_A03G2082	54.95	546	234	6	25	558	32	577	5.00E-177	618
Gh_D11G3305	55.31	546	229	6	27	558	25	569	2.00E-175	613
Gh_A01G1905	55.19	549	234	6	22	558	25	573	1.00E-174	610
Gh_A06G1415	50.99	553	251	7	22	558	20	568	3.00E-164	576
Gh_D06G1762	50.81	553	252	7	22	558	20	568	9.00E-164	574
Gh_A06G1413	50.63	553	253	7	22	558	20	568	5.00E-163	572
Gh_A13G0179	52.81	551	199	7	21	558	26	528	7.00E-163	571
Gh_A10G2140	50.73	550	250	7	26	558	24	569	3.00E-159	559
Gh_D10G2461	50.55	550	251	7	26	558	24	569	6.00E-159	558
Gh_D08G2159	50.55	544	258	6	26	558	34	577	3.00E-157	553
Gh_A03G2057	50.27	551	256	6	22	558	30	576	9.00E-157	551
Gh_A08G2350	50.37	544	259	6	26	558	34	577	1.00E-156	550
Gh_D03G1367	49.73	551	259	6	22	558	30	576	1.00E-156	550
Gh_A05G0853	50.09	545	209	7	19	558	25	511	2.00E-154	543
Gh_A03G2084	53.37	504	218	5	20	516	27	520	5.00E-154	542
Gh_D04G1224	49.27	548	266	6	22	558	21	567	2.00E-150	530
Gh_A04G0744	49.27	548	266	6	22	558	21	567	3.00E-150	530
Gh_D11G1042	47.45	550	271	6	21	558	31	574	6.00E-147	518
Gh_D12G1138	46.07	573	268	7	21	558	29	595	4.00E-144	509
Gh_A12G1019	46.21	567	264	7	27	558	35	595	2.00E-142	503
Gh_A05G2631	48.97	533	229	8	38	558	9	510	1.00E-141	501
Gh_Sca005020G01	46.28	551	236	6	19	556	25	528	7.00E-140	495
Gh_A13G2215	47.04	540	279	4	26	558	25	564	1.00E-139	494
Gh_D13G2524	46.68	542	282	4	24	558	23	564	1.00E-139	494
Gh_A05G2099	46.73	550	281	6	21	558	25	574	2.00E-139	493
Gh_A13G2103	57.93	416	158	4	158	558	83	496	5.00E-139	492
Gh_A13G2103	55.38	65	29	0	21	85	26	90	2.00E-16	85.5
Gh_A04G0743	47.1	552	276	6	23	558	22	573	9.00E-139	491
Gh_D05G2353	46.39	554	281	6	21	558	25	578	1.00E-137	488
Gh_D11G1874	47.04	540	253	8	32	547	13	543	8.00E-137	485
Gh_D04G1223	46.91	550	278	8	22	558	33	581	1.00E-135	481
Gh_D05G2354	44.32	537	294	5	26	558	27	562	2.00E-135	480
Gh_A13G2102	50.3	497	188	6	21	515	26	465	1.00E-134	478
Gh_A05G2116	44.53	539	291	7	26	558	27	563	1.00E-134	478
Gh_A05G2115	44.4	545	291	9	22	558	23	563	4.00E-134	476
Gh_A05G2113	42.41	540	304	5	26	558	21	560	1.00E-130	464
Gh_D04G1221	45.34	536	276	7	22	541	22	556	2.00E-129	460
Gh_A05G2117	42.96	540	299	5	26	558	23	560	8.00E-129	458
Gh_D05G2356	42.04	540	305	6	26	558	21	559	8.00E-129	458
Gh_A02G0638	40.55	545	313	7	23	558	23	565	1.00E-125	447
Gh_D06G1188	41.83	545	300	8	26	558	29	568	1.00E-121	434
Gh_D02G0685	39.53	549	314	8	23	558	23	566	1.00E-121	434
Gh_D03G1177	46.48	497	204	8	29	511	34	482	9.00E-121	431

	Gh_A06G0997	41.14	542	308	7	26	558	29	568	2.00E-120	431
	Gh_A05G2100	43.64	495	274	5	26	516	27	520	1.00E-118	424
	Gh_D13G2551	61.9	273	103	1	21	292	26	298	3.00E-105	380
	Gh_D13G2551	68.99	158	48	1	402	558	299	456	6.00E-64	243
	Gh_A13G0218	59.59	292	113	3	21	307	26	317	2.00E-100	364
	Gh_A13G0218	58.73	126	51	1	392	516	309	434	2.00E-38	157
	Gh_D06G2354	49.59	363	164	6	211	558	15	373	2.00E-95	347
	Gh_A11G0894	48.29	350	175	3	21	368	25	370	3.00E-91	333
	Gh_D05G2921	43.63	369	178	7	38	397	9	356	1.00E-85	315
	Gh_D13G0232	51.25	320	135	5	209	516	14	324	8.00E-82	302
	Gh_D06G1759	42.37	354	173	6	22	367	20	350	3.00E-73	273
	Gh_A11G1716	56.3	238	102	2	54	290	2	238	8.00E-72	269
	Gh_D13G0328	56.25	224	89	3	21	244	26	240	1.00E-71	268
	Gh_D13G0328	71.9	121	34	0	438	558	207	327	3.00E-50	197
	Gh_A12G0840	32.16	541	324	15	23	536	22	546	2.00E-61	234
	Gh_D12G0913	31.84	537	330	14	23	536	22	545	1.00E-60	232
	Gh_A02G1272	31.85	540	327	14	23	536	35	559	2.00E-60	231
	Gh_D03G0412	31.25	544	325	14	23	536	35	559	7.00E-59	226
	Gh_A06G1412	58.89	180	72	2	381	558	1	180	1.00E-58	225
	Gh_A08G1751	31.68	543	320	18	24	536	28	549	1.00E-54	212
	Gh_D08G2100	31.68	543	320	18	24	536	28	549	2.00E-54	211
	Gh_D05G2355	39.93	268	147	5	20	286	21	275	2.00E-51	201
	Gh_D05G2420	27.96	540	359	12	20	558	20	530	2.00E-50	198
	Gh_A05G2165	28.07	538	357	12	22	558	22	530	2.00E-50	198
**AtLAC05**	Gh_A06G1415	79.26	569	110	2	12	580	8	568	0	952
	Gh_D06G1762	79.12	570	111	2	11	580	7	568	0	951
	Gh_A06G1413	78.77	570	113	2	11	580	7	568	0	947
	Gh_A10G2140	76.72	580	124	3	1	580	1	569	0	929
	Gh_D10G2461	76.72	580	124	3	1	580	1	569	0	928
	Gh_A05G2631	72.71	546	110	3	35	580	4	510	0	816
	Gh_A03G2057	65.74	575	186	5	6	580	13	576	0	781
	Gh_D03G1367	66.09	575	184	5	6	580	13	576	0	780
	Gh_D08G2159	66.55	553	174	5	29	580	35	577	0	767
	Gh_A08G2350	66	553	177	5	29	580	35	577	0	762
	Gh_D06G2354	81.13	371	62	2	210	580	11	373	2.00E-177	620
	Gh_D04G1224	51.79	558	246	4	30	580	26	567	4.00E-173	605
	Gh_A04G0744	51.43	558	248	4	30	580	26	567	2.00E-171	600
	Gh_A13G0237	50.87	578	253	8	7	580	13	563	4.00E-170	595
	Gh_D13G0253	50.17	578	257	8	7	580	13	563	8.00E-169	591
	Gh_A09G1445	48.9	593	270	12	2	580	10	583	5.00E-168	588
	Gh_D09G1454	49.16	592	270	11	2	580	10	583	1.00E-167	587
	Gh_A13G2215	52.78	557	236	6	31	580	28	564	1.00E-166	584
	Gh_D13G0194	49.57	585	271	11	4	580	8	576	1.00E-165	581
	Gh_A13G1977	51.71	557	246	8	30	580	38	577	4.00E-165	579
	Gh_D13G2524	52.06	557	240	6	31	580	28	564	4.00E-165	579
	Gh_D10G0895	48.97	582	266	8	3	580	8	562	4.00E-165	579
	Gh_D03G1181	50.59	593	259	12	4	580	3	577	7.00E-165	578
	Gh_A11G2922	49.32	584	271	10	8	580	14	583	7.00E-165	578
	Gh_D03G1180	50.25	593	261	11	4	580	3	577	8.00E-165	578
	Gh_D11G3307	48.97	584	273	10	8	580	14	583	1.00E-164	577
	Gh_D04G1223	50.95	581	263	6	8	580	15	581	1.00E-164	577
	Gh_A13G0216	50.9	558	253	10	30	580	33	576	3.00E-164	576
	Gh_A10G0858	48.8	582	267	8	3	580	8	562	3.00E-164	576
	Gh_D13G0231	50.72	558	254	10	30	580	33	576	1.00E-163	573
	Gh_D13G2373	50.63	557	252	8	30	580	38	577	6.00E-163	572
	Gh_D05G3888	49.23	581	276	10	7	580	11	579	7.00E-163	572
	Gh_A03G2082	50.08	593	262	12	4	580	3	577	9.00E-163	571
	Gh_A05G0849	48.71	581	279	10	7	580	11	579	1.00E-161	567
	Gh_D04G1243	50.09	555	250	6	30	580	25	556	3.00E-161	566
	Gh_A04G0743	49.23	587	267	8	8	580	4	573	8.00E-161	565
	Gh_A03G0583	49.31	578	261	9	8	580	11	561	3.00E-160	563
	Gh_D01G2166	49.74	581	265	11	8	580	12	573	7.00E-160	561
	Gh_D11G3322	48.53	579	269	7	6	580	3	556	1.00E-159	561

	Gh_A11G2920	48.72	587	277	11	1	580	1	570	1.00E-159	560
	Gh_D03G1128	48.26	574	266	8	11	580	10	556	2.00E-158	556
	Gh_A11G2936	48.01	579	272	7	6	580	3	556	7.00E-158	555
	Gh_A01G1905	48.88	581	270	10	8	580	12	573	1.00E-157	554
	Gh_D04G1221	48.77	570	261	8	8	563	4	556	4.00E-157	552
	Gh_A03G0417	47.74	574	269	8	11	580	10	556	1.00E-156	551
	Gh_D05G0888	47.49	577	273	8	8	580	6	556	1.00E-156	551
	Gh_D03G0865	48.62	578	261	10	8	580	11	557	4.00E-156	549
	Gh_A05G0758	47.14	577	275	8	8	580	6	556	8.00E-156	548
	Gh_D11G3305	47.87	587	281	12	1	580	1	569	1.00E-154	544
	Gh_A05G2099	46.93	586	293	5	1	580	1	574	4.00E-154	542
	Gh_D11G1042	46.76	586	286	6	4	580	6	574	1.00E-153	541
	Gh_D05G2353	46.8	579	292	5	6	580	12	578	3.00E-152	536
	Gh_D12G1138	44.84	611	290	7	1	580	1	595	1.00E-150	531
	Gh_D01G2209	47.59	580	276	7	5	580	3	558	4.00E-150	529
	Gh_A01G1948	47.41	580	277	7	5	580	3	558	7.00E-150	528
	Gh_A12G1019	44.84	611	290	7	1	580	1	595	1.00E-149	528
	Gh_D10G2466	45.48	609	270	10	7	580	1	582	1.00E-149	527
	Gh_D05G2921	67.1	386	97	2	34	419	3	358	2.00E-148	523
	Gh_A05G2622	44.56	579	288	7	10	580	2	555	5.00E-145	512
	Gh_D05G2912	44.56	579	288	7	10	580	2	555	6.00E-145	512
	Gh_D06G1759	68.72	374	88	4	10	383	9	353	2.00E-143	506
	Gh_A03G2084	49.82	548	224	14	8	538	7	520	6.00E-142	502
	Gh_A13G0179	46.42	558	230	10	30	580	33	528	8.00E-140	495
	Gh_D11G1874	45.86	543	271	7	35	568	14	542	5.00E-136	482
	Gh_D05G2354	41.29	574	309	9	13	580	11	562	6.00E-130	462
	Gh_A05G0853	43.85	561	232	10	25	580	29	511	2.00E-129	461
	Gh_A02G0638	39.83	580	319	10	7	580	10	565	3.00E-127	453
	Gh_A05G2117	39.9	574	319	8	13	580	7	560	7.00E-127	452
	Gh_A05G2115	40.63	576	311	10	13	580	11	563	3.00E-126	449
	Gh_D05G2356	39.65	575	320	9	13	580	5	559	5.00E-125	446
	Gh_Sca005020G01	41.36	573	271	10	11	578	16	528	2.00E-124	444
	Gh_A05G2113	39.06	576	323	9	13	580	5	560	6.00E-123	439
	Gh_D02G0685	39.22	566	317	10	22	580	21	566	1.00E-122	438
	Gh_A05G2116	38.93	560	314	8	27	580	26	563	7.00E-121	432
	Gh_D06G1188	39.79	583	312	13	8	580	15	568	2.00E-118	423
	Gh_A06G0997	39.79	583	312	13	8	580	15	568	4.00E-118	423
	Gh_D03G1177	43.74	535	220	14	7	527	9	476	2.00E-115	414
	Gh_A05G2100	40.79	532	287	9	13	538	11	520	2.00E-115	414
	Gh_A13G2103	48.26	431	182	7	167	580	90	496	7.00E-106	382
	Gh_A13G2102	40.56	535	233	9	7	537	12	465	1.00E-102	371
	Gh_A11G0894	43.24	377	199	2	4	379	6	368	2.00E-89	328
	Gh_A06G1412	85.64	181	25	1	400	580	1	180	3.00E-89	327
	Gh_A13G0218	52.67	281	130	3	30	308	33	312	1.00E-87	321
	Gh_A13G0218	47.06	153	74	3	391	538	284	434	2.00E-31	135
	Gh_D13G2551	48.78	287	145	1	7	293	12	296	2.00E-84	311
	Gh_D13G2551	58.23	158	65	1	423	580	300	456	3.00E-51	200
	Gh_D13G0232	46.31	339	146	12	210	538	12	324	3.00E-73	273
	Gh_A11G1716	47.86	257	133	1	55	311	1	256	3.00E-65	247
	Gh_A12G0840	31.76	551	295	20	44	558	41	546	3.00E-59	227
	Gh_D12G0913	31.28	553	294	20	44	558	41	545	4.00E-59	227
	Gh_A08G1751	28.89	585	333	21	11	558	11	549	4.00E-58	223
	Gh_D08G2100	28.89	585	333	21	11	558	11	549	4.00E-58	223
	Gh_D03G0412	31.06	586	326	21	7	558	18	559	1.00E-57	222
	Gh_D13G0328	50.54	184	91	0	31	214	34	217	5.00E-57	219
	Gh_D13G0328	62.39	117	44	0	464	580	211	327	5.00E-41	166
	Gh_A02G1272	30.94	585	328	21	7	558	18	559	8.00E-57	219
	Gh_A11G0895	55.19	183	77	3	400	580	1	180	5.00E-53	206
	Gh_D04G1220	50.79	191	93	1	8	197	4	194	2.00E-52	204
	Gh_A05G2114	35.58	312	174	6	1	309	1	288	1.00E-51	202
	Gh_D05G2355	35.92	309	177	6	1	309	1	288	4.00E-51	200
**AtLAC06**	Gh_D11G1042	70.3	542	161	0	28	569	33	574	0	836
	Gh_A12G1019	65.84	565	170	1	28	569	31	595	0	806

Gh_D12G1138	66.19	565	168	1	28	569	31	595	0	805
Gh_D11G1874	53.17	536	222	5	37	553	13	538	1.00E-170	597
Gh_A11G0894	69.45	347	106	0	28	374	27	373	3.00E-154	543
Gh_A09G1445	44.86	555	282	8	31	569	37	583	9.00E-153	538
Gh_D11G3307	44.33	564	290	8	22	569	28	583	8.00E-152	535
Gh_D09G1454	44.68	555	283	9	31	569	37	583	3.00E-151	533
Gh_A11G2922	43.97	564	292	8	22	569	28	583	5.00E-150	528
Gh_D13G2373	44.71	577	302	6	3	569	8	577	1.00E-149	527
Gh_D10G2461	46.28	551	273	5	34	569	27	569	3.00E-149	526
Gh_D04G1243	46.08	549	279	5	27	569	19	556	6.00E-149	525
Gh_A13G1977	45.63	561	286	9	20	569	25	577	3.00E-148	523
Gh_A10G2140	45.92	551	275	5	34	569	27	569	5.00E-148	522
Gh_D05G3888	44.74	561	276	10	31	569	31	579	7.00E-148	521
Gh_A06G1415	45.45	550	288	5	29	569	22	568	1.00E-147	521
Gh_D06G1762	45.64	550	287	5	29	569	22	568	1.00E-147	520
Gh_A05G0849	44.46	560	279	10	31	569	31	579	2.00E-147	520
Gh_D04G1224	45.54	549	288	5	29	569	22	567	2.00E-147	520
Gh_D03G1367	47.19	551	275	8	29	569	32	576	3.00E-147	520
Gh_A06G1413	45.45	550	288	5	29	569	22	568	5.00E-147	518
Gh_A04G0744	44.81	549	292	5	29	569	22	567	6.00E-147	518
Gh_A13G0216	43.96	555	286	9	31	569	31	576	7.00E-147	518
Gh_A03G2057	46.92	552	275	8	29	569	32	576	8.00E-147	518
Gh_D11G3322	44.99	549	285	5	27	569	19	556	8.00E-147	518
Gh_D13G0231	44.5	555	283	9	31	569	31	576	1.00E-146	518
Gh_D13G0194	44.5	555	283	9	31	569	31	576	1.00E-146	518
Gh_D10G0895	46.08	549	274	10	29	569	28	562	3.00E-146	516
Gh_A10G0858	46.08	549	274	10	29	569	28	562	2.00E-145	513
Gh_D08G2159	45.52	547	283	7	33	569	36	577	6.00E-145	512
Gh_D03G1181	45.21	553	281	9	31	569	33	577	1.00E-144	511
Gh_A01G1948	46.27	549	278	5	27	569	21	558	1.00E-144	511
Gh_A11G2936	44.44	549	288	5	27	569	19	556	2.00E-144	510
Gh_A08G2350	45.62	548	281	8	33	569	36	577	2.00E-144	510
Gh_D01G2209	45.9	549	280	5	27	569	21	558	2.00E-144	510
Gh_A05G0758	45.4	544	278	6	32	569	26	556	3.00E-144	509
Gh_D03G1180	45.57	553	279	9	31	569	33	577	4.00E-144	509
Gh_D13G0253	45.7	547	279	7	29	569	29	563	5.00E-144	509
Gh_A13G0237	45.89	547	278	7	29	569	29	563	9.00E-144	508
Gh_D01G2166	44.05	563	293	9	21	569	19	573	2.00E-143	506
Gh_A01G1905	44.4	563	291	9	21	569	19	573	4.00E-143	506
Gh_D05G0888	45.4	544	278	6	32	569	26	556	5.00E-143	505
Gh_A03G2082	44.48	553	285	9	31	569	33	577	9.00E-142	501
Gh_A11G2920	43.73	558	290	8	28	569	21	570	9.00E-141	498
Gh_A03G0583	45.82	550	272	12	29	569	29	561	8.00E-140	495
Gh_A03G0417	44.63	549	284	7	27	569	22	556	1.00E-139	494
Gh_D03G1128	44.44	549	285	7	27	569	22	556	1.00E-139	494
Gh_D10G2466	43.01	572	279	7	32	569	24	582	6.00E-138	488
Gh_D11G3305	43.83	559	287	11	28	569	21	569	2.00E-137	487
Gh_D03G0865	44.73	550	274	12	29	569	29	557	4.00E-135	479
Gh_A05G2622	42.81	549	295	6	27	569	20	555	8.00E-135	478
Gh_D05G2912	42.99	549	294	6	27	569	20	555	2.00E-134	477
Gh_D13G2524	44.38	543	289	5	34	569	28	564	3.00E-134	476
Gh_A04G0743	43.71	556	297	6	27	569	21	573	6.00E-134	475
Gh_A13G2215	44.57	543	288	5	34	569	28	564	9.00E-134	475
Gh_A05G2099	44.36	550	284	6	34	569	33	574	2.00E-133	474
Gh_A05G2631	43.87	538	267	5	37	569	3	510	4.00E-132	469
Gh_D05G2353	43.68	554	286	6	34	569	33	578	2.00E-131	467
Gh_D04G1223	44.12	553	296	6	27	569	32	581	2.00E-130	464
Gh_D05G2354	41.41	553	297	9	29	569	25	562	8.00E-128	455
Gh_D04G1221	42.86	539	292	6	27	552	21	556	6.00E-127	452
Gh_D05G2356	41.19	556	297	13	29	569	19	559	7.00E-125	445
Gh_A05G2113	41.11	557	297	13	29	569	19	560	5.00E-124	442
Gh_A13G0179	41.41	553	255	10	31	569	31	528	5.00E-124	442
Gh_A05G0853	40.22	547	260	12	27	569	28	511	6.00E-121	432

	Gh_A05G2117	38.18	550	321	7	29	569	21	560	1.00E-120	431
	Gh_A02G0638	39.36	559	318	7	22	569	17	565	2.00E-119	427
	Gh_D02G0685	39.25	558	317	8	24	569	19	566	8.00E-118	422
	Gh_A05G2115	38.22	552	317	8	29	569	25	563	4.00E-117	419
	Gh_A05G2116	38.52	553	314	10	29	569	25	563	7.00E-117	419
	Gh_A03G2084	43.31	508	257	11	31	527	33	520	1.00E-116	418
	Gh_A06G0997	38.81	554	314	11	29	569	27	568	3.00E-114	409
	Gh_D06G1188	39.17	554	312	11	29	569	27	568	2.00E-113	407
	Gh_A05G2100	40.75	508	282	7	29	528	25	521	1.00E-110	398
	Gh_Sca005020G01	35.37	557	288	9	27	567	28	528	2.00E-104	377
	Gh_D03G1177	39.6	500	226	13	34	516	36	476	2.00E-97	354
	Gh_A11G1716	57.87	254	106	1	59	312	2	254	4.00E-90	330
	Gh_D06G2354	45.89	353	179	5	226	569	24	373	1.00E-89	328
	Gh_A13G2103	40.95	420	215	10	170	569	90	496	5.00E-89	326
	Gh_A13G0218	42.94	361	177	4	31	363	31	390	8.00E-85	312
	Gh_A13G0218	41.54	130	69	3	401	527	309	434	8.00E-26	116
	Gh_D13G2551	45.09	346	181	4	29	366	29	373	1.00E-83	308
	Gh_D13G2551	46.91	162	81	3	410	569	298	456	4.00E-41	167
	Gh_A13G2102	41.98	393	210	7	140	526	85	465	7.00E-83	305
	Gh_D05G2921	41.62	370	198	3	38	406	4	356	5.00E-80	296
	Gh_A11G0895	72.78	180	49	0	390	569	1	180	6.00E-75	279
	Gh_D06G1759	39.19	347	186	4	29	369	22	349	8.00E-71	265
	Gh_D12G0913	30.74	566	321	19	27	553	21	554	2.00E-60	231
	Gh_A12G0840	30.73	563	326	18	27	553	21	555	6.00E-60	229
	Gh_D13G0232	36.86	331	175	10	213	527	12	324	8.00E-60	229
	Gh_A08G1751	28.78	556	329	19	27	547	26	549	1.00E-57	221
	Gh_D08G2100	28.96	556	328	19	27	547	26	549	2.00E-57	221
	Gh_D13G0328	53.8	184	85	0	29	212	29	212	7.00E-56	216
	Gh_D13G0328	49.25	134	66	1	438	569	194	327	2.00E-37	155
	Gh_A05G2112	38.37	258	146	3	29	286	25	269	8.00E-50	196
	Gh_D05G2357	37.98	258	147	3	29	286	25	269	8.00E-49	192
	Gh_A06G1412	48.09	183	89	3	390	569	1	180	8.00E-49	192
	Gh_D04G1220	47.98	173	90	0	27	199	21	193	9.00E-48	189
	Gh_D05G2355	35.61	264	157	3	29	292	25	275	1.00E-46	185
	Gh_A05G2114	35.98	264	156	3	29	292	25	275	2.00E-46	184
**AtLAC07**	Gh_A04G0744	63.21	530	186	3	26	550	25	550	0	724
	Gh_A13G2215	65.65	527	174	4	26	550	26	547	0	723
	Gh_D04G1224	62.83	530	188	3	26	550	25	550	0	721
	Gh_D13G2524	64.9	527	178	4	26	550	26	547	0	716
	Gh_A04G0743	61.19	536	192	5	26	550	26	556	0	676
	Gh_D04G1221	60.63	536	195	5	26	550	26	556	0	669
	Gh_D04G1223	61.77	531	194	4	26	550	37	564	0	668
	Gh_A06G1415	50.65	535	244	7	27	550	26	551	1.00E-157	554
	Gh_D10G2461	51.12	536	241	8	27	550	26	552	3.00E-157	553
	Gh_D06G1762	50.65	535	244	7	27	550	26	551	3.00E-157	553
	Gh_A10G2140	50.93	536	242	8	27	550	26	552	2.00E-156	550
	Gh_A06G1413	50.09	535	247	7	27	550	26	551	8.00E-156	548
	Gh_A13G0237	50.67	525	247	7	27	550	33	546	1.00E-150	531
	Gh_A09G1445	49.06	534	254	8	28	550	40	566	3.00E-150	530
	Gh_D13G0253	50.67	525	247	7	27	550	33	546	3.00E-150	529
	Gh_D09G1454	48.88	534	255	7	28	550	40	566	5.00E-150	529
	Gh_D11G3307	49.16	533	255	5	28	550	40	566	2.00E-149	526
	Gh_D13G2373	49.72	531	250	7	28	550	39	560	5.00E-149	525
	Gh_A11G2922	48.97	533	256	5	28	550	40	566	8.00E-149	525
	Gh_A03G2057	49.53	533	249	8	28	550	37	559	1.00E-148	524
	Gh_D03G1367	49.34	533	250	8	28	550	37	559	1.00E-148	524
	Gh_A13G1977	49.72	531	250	7	28	550	39	560	4.00E-148	522
	Gh_D03G1180	49.72	533	250	6	28	550	36	560	7.00E-148	521
	Gh_D10G0895	49.24	524	256	6	27	550	32	545	2.00E-147	520
	Gh_D03G1181	49.63	534	249	7	28	550	36	560	4.00E-147	519
	Gh_A10G0858	49.05	524	257	6	27	550	32	545	7.00E-147	518
	Gh_A03G0583	49.62	526	249	9	27	550	33	544	2.00E-145	513
	Gh_A03G2082	48.88	534	253	7	28	550	36	560	2.00E-145	513

Gh_A08G2350	48.69	534	253	9	28	550	37	560	2.00E-144	510
Gh_D08G2159	48.31	534	255	9	28	550	37	560	3.00E-144	509
Gh_D13G0194	48.12	532	261	5	28	550	34	559	1.00E-143	508
Gh_A11G2920	47.94	534	260	6	28	550	27	553	3.00E-143	506
Gh_A05G0849	47.29	535	264	5	28	550	34	562	3.00E-143	506
Gh_D05G3888	46.92	535	266	5	28	550	34	562	3.00E-143	506
Gh_D01G2166	48.68	532	257	7	28	550	32	556	9.00E-143	504
Gh_A13G0216	46.8	532	268	5	28	550	34	559	3.00E-142	503
Gh_D03G0865	49.24	526	247	10	27	550	33	540	7.00E-142	501
Gh_D13G0231	47.19	534	263	6	28	550	34	559	8.00E-142	501
Gh_A05G2631	48.39	529	224	10	32	550	4	493	3.00E-139	493
Gh_A01G1905	47.74	532	262	7	28	550	32	556	5.00E-139	492
Gh_D11G3305	46.82	534	265	7	28	550	27	552	7.00E-139	491
Gh_A05G2099	46.14	531	274	5	27	550	32	557	3.00E-138	489
Gh_D05G0888	47.42	523	264	6	28	550	28	539	4.00E-137	486
Gh_D05G2353	45.61	535	275	6	27	550	32	561	1.00E-136	484
Gh_D03G1128	47.23	523	264	6	28	550	29	539	1.00E-136	484
Gh_A05G0758	47.04	523	266	6	28	550	28	539	2.00E-136	483
Gh_D04G1243	47.35	528	259	7	28	550	26	539	8.00E-136	481
Gh_A11G2936	46.97	528	261	7	28	550	26	539	4.00E-135	479
Gh_D11G3322	46.97	528	261	7	28	550	26	539	6.00E-135	478
Gh_A03G0417	46.85	523	266	6	28	550	29	539	9.00E-135	478
Gh_D05G2912	45.32	523	275	5	28	550	27	538	2.00E-130	463
Gh_D10G2466	44.99	549	263	7	30	550	28	565	3.00E-130	463
Gh_A05G2622	45.12	523	276	5	28	550	27	538	6.00E-130	462
Gh_A03G2084	48.43	508	229	9	28	525	36	520	2.00E-129	461
Gh_D11G1042	43.31	531	281	8	28	550	39	557	1.00E-126	451
Gh_D01G2209	45.94	529	267	7	27	550	27	541	8.00E-126	448
Gh_A01G1948	45.94	529	267	7	27	550	27	541	1.00E-125	447
Gh_D11G1874	43.66	536	275	10	31	550	13	537	1.00E-125	447
Gh_A12G1019	41.91	556	276	10	28	550	37	578	1.00E-123	441
Gh_D12G1138	41.37	556	279	10	28	550	37	578	3.00E-123	439
Gh_A05G0853	43.21	530	224	7	28	550	35	494	2.00E-117	421
Gh_D05G2354	42.23	528	288	11	28	550	30	545	8.00E-117	418
Gh_A13G0179	42.86	532	241	8	28	550	34	511	6.00E-114	409
Gh_Sca005020G01	40.82	539	243	8	28	550	35	513	3.00E-110	396
Gh_D05G2356	40.83	529	297	11	28	550	24	542	2.00E-109	394
Gh_A05G2113	40.04	532	298	11	28	550	24	543	2.00E-108	390
Gh_A05G2100	42.15	503	274	11	28	525	30	520	1.00E-107	387
Gh_A02G0638	38.91	532	304	9	28	550	29	548	1.00E-107	387
Gh_A05G2116	38.55	524	314	6	28	550	30	546	3.00E-107	386
Gh_A05G2117	38.21	526	314	7	28	550	26	543	7.00E-107	385
Gh_A05G2115	38.9	527	308	10	28	550	30	546	1.00E-105	381
Gh_D03G1177	43.7	492	205	10	37	514	43	476	1.00E-105	381
Gh_D02G0685	38.46	533	306	10	28	550	29	549	2.00E-105	380
Gh_D06G1188	38.87	530	306	11	28	550	32	550	2.00E-101	367
Gh_A06G0997	39.16	526	302	11	32	550	36	550	3.00E-100	363
Gh_A13G2102	41.16	498	228	9	27	524	33	465	5.00E-98	356
Gh_D05G2921	44.56	377	176	4	32	400	4	355	1.00E-89	328
Gh_A13G2103	43.5	400	203	6	164	550	90	479	1.00E-88	325
Gh_D06G2354	47.86	351	163	7	211	550	15	356	3.00E-87	320
Gh_A11G0894	44.83	348	184	3	28	375	33	372	3.00E-85	314
Gh_D13G2551	54.51	266	120	1	27	292	33	297	9.00E-85	312
Gh_D13G2551	51.37	146	67	1	405	550	298	439	2.00E-40	165
Gh_A13G0218	46.99	349	173	6	28	370	34	376	7.00E-83	305
Gh_A13G0218	47.37	133	60	3	396	525	309	434	8.00E-30	129
Gh_D06G1759	44.44	351	168	6	27	374	26	352	5.00E-77	286
Gh_A11G1716	50.59	255	124	1	54	308	3	255	5.00E-70	263
Gh_D04G1220	64.29	168	60	0	26	193	26	193	5.00E-66	249
Gh_D13G0232	41.77	328	163	7	209	525	14	324	2.00E-65	248
Gh_A12G0840	32.6	543	319	18	27	545	27	546	2.00E-63	241
Gh_D12G0913	32.47	542	320	17	27	545	27	545	2.00E-63	241
Gh_D13G0328	55.31	179	80	0	27	205	33	211	2.00E-57	221

	Gh_D13G0328	56.12	98	43	0	453	550	213	310	5.00E-30	130
	Gh_A08G1751	30.78	536	310	18	41	546	46	550	5.00E-56	216
	Gh_D08G2100	30.04	536	314	16	41	546	46	550	9.00E-56	216
	Gh_D03G0412	30.9	534	312	18	41	545	54	559	1.00E-55	215
	Gh_A02G1272	30.52	534	314	18	41	545	54	559	2.00E-55	214
	Gh_D05G2355	36.92	260	150	4	28	287	30	275	1.00E-50	198
	Gh_D05G2357	37.84	259	147	4	28	286	30	274	2.00E-50	197
	Gh_A05G2112	38.22	259	146	4	28	286	30	274	3.00E-50	197
	Gh_D05G2589	38.57	280	151	7	28	307	31	289	4.00E-50	197
**AtLAC08**	Gh_A04G0743	56.02	548	226	6	14	549	12	556	0	651
	Gh_D04G1224	55.78	536	229	3	20	549	17	550	0	644
	Gh_D04G1221	56.11	565	233	6	14	566	12	573	0	642
	Gh_A04G0744	55.78	536	229	3	20	549	17	550	0	642
	Gh_A13G2215	57.66	529	212	6	27	549	25	547	0	641
	Gh_D13G2524	57.82	531	212	6	25	549	23	547	0	641
	Gh_D04G1223	55.41	545	231	6	14	549	23	564	0	634
	Gh_A06G1415	46.75	554	269	9	12	549	8	551	7.00E-147	518
	Gh_D06G1762	46.39	554	271	8	12	549	8	551	2.00E-146	516
	Gh_A06G1413	46.21	554	272	9	12	549	8	551	3.00E-144	509
	Gh_A03G2057	48.09	551	261	9	13	549	20	559	8.00E-143	505
	Gh_D03G1367	47.01	551	267	8	13	549	20	559	1.00E-141	501
	Gh_D10G2461	45.21	553	277	7	13	549	10	552	3.00E-141	499
	Gh_D10G0895	46.52	546	269	9	13	549	14	545	2.00E-140	497
	Gh_A10G2140	44.85	553	279	7	13	549	10	552	1.00E-139	494
	Gh_A10G0858	46.52	546	269	9	13	549	14	545	2.00E-139	494
	Gh_A08G2350	47.31	558	268	9	7	549	14	560	3.00E-139	493
	Gh_A09G1445	45.52	558	275	11	12	549	18	566	6.00E-138	489
	Gh_A13G0237	44.34	548	280	8	13	549	13	546	2.00E-137	487
	Gh_D09G1454	45.88	558	273	11	12	549	18	566	3.00E-137	486
	Gh_D08G2159	46.95	558	270	9	7	549	14	560	4.00E-137	486
	Gh_D13G0253	44.34	548	280	8	13	549	13	546	4.00E-137	486
	Gh_A03G0583	44.32	546	279	9	12	549	16	544	6.00E-137	485
	Gh_D13G0194	46.1	564	272	10	8	549	6	559	7.00E-136	482
	Gh_D11G3307	44.74	570	279	9	8	549	5	566	3.00E-135	479
	Gh_A13G0216	46.48	525	259	7	39	549	43	559	5.00E-134	476
	Gh_D04G1243	45.2	542	279	7	14	549	10	539	6.00E-134	475
	Gh_D13G0231	46.1	525	261	7	39	549	43	559	2.00E-133	474
	Gh_D11G3322	45.32	545	274	9	14	549	10	539	3.00E-133	473
	Gh_A11G2922	44.21	570	282	9	8	549	5	566	4.00E-133	473
	Gh_D13G2373	43.21	567	294	9	3	549	2	560	2.00E-132	470
	Gh_A11G2936	44.28	542	284	7	14	549	10	539	3.00E-132	469
	Gh_A13G1977	43.21	567	294	8	3	549	2	560	5.00E-132	469
	Gh_D05G3888	43.6	555	288	7	12	549	16	562	7.00E-132	468
	Gh_A05G0849	43.78	555	287	7	12	549	16	562	7.00E-132	468
	Gh_D03G0865	43.59	546	279	10	12	549	16	540	3.00E-131	466
	Gh_A05G2622	44.63	549	279	8	12	549	4	538	3.00E-131	466
	Gh_D05G0888	43.65	543	286	9	13	549	11	539	7.00E-131	465
	Gh_A05G0758	44.32	546	279	12	15	549	8	539	1.00E-130	464
	Gh_D05G2912	44.4	545	279	7	15	549	8	538	3.00E-130	463
	Gh_D03G1128	45.34	547	271	13	15	549	9	539	4.00E-130	462
	Gh_D03G1180	44.82	560	275	11	13	549	12	560	7.00E-129	458
	Gh_A11G2920	42.96	554	291	8	12	549	9	553	1.00E-128	457
	Gh_D01G2166	43.11	559	294	9	7	549	6	556	2.00E-128	457
	Gh_D03G1181	44.74	561	274	12	13	549	12	560	3.00E-128	456
	Gh_A03G0417	45.05	546	274	13	15	549	9	539	4.00E-128	456
	Gh_A05G2631	45.83	528	238	9	33	549	3	493	1.00E-127	454
	Gh_A05G2099	42.88	548	296	6	13	549	16	557	1.00E-127	454
	Gh_D10G2466	43.48	575	273	10	13	549	5	565	2.00E-127	454
	Gh_A01G1905	43.19	551	290	8	12	549	16	556	2.00E-126	450
	Gh_A03G2082	46.29	525	253	11	42	549	48	560	4.00E-126	449
	Gh_D05G2353	41.95	553	300	6	12	549	15	561	8.00E-125	445
	Gh_A01G1948	46.27	523	260	8	36	549	31	541	2.00E-124	444
	Gh_D01G2209	45.89	523	262	8	36	549	31	541	5.00E-124	442

	Gh_D11G3305	42.06	554	295	9	12	549	9	552	6.00E-124	442
	Gh_D11G1042	41.37	527	290	7	32	549	41	557	2.00E-117	420
	Gh_D11G1874	40.78	537	284	9	34	549	14	537	1.00E-113	408
	Gh_A03G2084	42.14	541	252	12	13	524	12	520	7.00E-113	405
	Gh_A12G1019	39.09	550	293	8	32	549	39	578	8.00E-112	402
	Gh_D12G1138	39.27	550	292	8	32	549	39	578	8.00E-112	402
	Gh_D05G2354	39.75	551	302	10	13	549	11	545	9.00E-112	402
	Gh_A05G0853	41.75	515	232	7	39	549	44	494	4.00E-110	396
	Gh_A13G0179	41	522	238	8	42	549	46	511	2.00E-108	390
	Gh_A05G2115	39.27	545	309	9	15	549	14	546	9.00E-108	388
	Gh_A05G2117	38.8	567	325	9	13	568	7	562	2.00E-106	384
	Gh_D05G2356	38.98	549	312	11	13	549	5	542	6.00E-106	382
	Gh_A02G0638	39.86	577	322	11	12	573	6	572	6.00E-106	382
	Gh_A05G2116	39.25	535	302	10	25	549	25	546	8.00E-105	379
	Gh_D06G1188	38.48	551	313	9	13	549	12	550	5.00E-104	376
	Gh_A05G2113	38.55	550	314	12	13	549	5	543	8.00E-104	375
	Gh_A06G0997	38.11	551	315	9	13	549	12	550	1.00E-103	375
	Gh_A05G2100	39.54	526	288	10	13	524	11	520	4.00E-103	373
	Gh_D02G0685	39.34	577	324	12	15	575	9	575	2.00E-102	370
	Gh_Sca005020G01	36.96	552	277	8	13	549	18	513	5.00E-96	349
	Gh_A13G2102	39.81	520	235	10	13	523	15	465	6.00E-95	345
	Gh_D03G1177	39.66	527	236	14	14	514	7	477	7.00E-93	339
	Gh_A13G2103	40.43	460	236	10	114	549	34	479	9.00E-89	325
	Gh_D06G2354	44.32	352	177	5	207	549	15	356	5.00E-86	316
	Gh_D13G2551	48	300	143	3	8	294	7	306	2.00E-78	291
	Gh_D13G2551	48.15	162	77	2	390	549	283	439	5.00E-41	166
	Gh_D05G2921	42.44	377	182	7	34	399	4	356	1.00E-75	281
	Gh_A11G0894	43.44	343	183	3	32	368	35	372	5.00E-75	280
	Gh_A13G0218	51.94	258	116	3	39	288	43	300	9.00E-71	265
	Gh_A13G0218	47.69	130	62	2	395	524	311	434	3.00E-29	127
	Gh_D06G1759	40.66	364	185	7	12	365	8	350	6.00E-70	263
	Gh_D13G0232	41.46	328	167	6	205	524	14	324	8.00E-67	252
	Gh_A12G0840	32.31	554	313	17	24	544	22	546	2.00E-66	251
	Gh_D03G0412	32	575	314	18	13	544	19	559	6.00E-64	243
	Gh_D12G0913	31.44	563	305	18	24	544	22	545	7.00E-64	243
	Gh_A02G1272	31.62	563	325	17	14	544	25	559	2.00E-63	241
	Gh_A08G1751	29.79	574	324	19	13	544	13	549	1.00E-58	225
	Gh_D08G2100	29.79	574	324	19	13	544	13	549	2.00E-58	224
	Gh_A11G1716	46.12	258	131	3	56	307	3	258	4.00E-58	223
	Gh_D04G1220	51.37	183	83	2	14	190	12	194	1.00E-52	205
	Gh_A05G2112	38.46	273	156	4	7	278	10	271	4.00E-49	193
	Gh_D05G2355	37.55	277	161	4	7	282	10	275	6.00E-49	193
	Gh_D13G0328	45.07	213	107	2	23	229	27	235	2.00E-48	191
	Gh_D13G0328	53.47	101	47	0	449	549	210	310	2.00E-30	131
	Gh_D05G2357	38.1	273	157	4	7	278	10	271	1.00E-47	189
	Gh_D05G2589	39.6	250	142	4	34	282	35	276	2.00E-47	188
**AtLAC09**	Gh_A04G0743	56.44	567	230	6	14	568	12	573	5.00E-180	628
	Gh_A04G0744	54.95	555	240	3	20	568	17	567	2.00E-179	626
	Gh_D04G1224	54.95	555	240	3	20	568	17	567	3.00E-179	626
	Gh_A13G2215	58.72	533	206	5	42	568	40	564	2.00E-177	620
	Gh_D13G2524	58.54	533	207	5	42	568	40	564	5.00E-177	619
	Gh_D04G1221	56.44	567	230	6	14	568	12	573	6.00E-176	615
	Gh_D04G1223	56.03	564	234	6	14	568	23	581	1.00E-173	607
	Gh_A06G1415	46.07	573	281	9	12	568	8	568	2.00E-141	500
	Gh_D06G1762	45.72	573	283	8	12	568	8	568	1.00E-140	497
	Gh_A06G1413	45.55	573	284	9	12	568	8	568	2.00E-138	490
	Gh_A09G1445	45.5	545	270	9	40	568	50	583	1.00E-137	487
	Gh_D09G1454	45.87	545	268	9	40	568	50	583	9.00E-137	484
	Gh_D10G2461	46.22	543	264	7	42	568	39	569	2.00E-136	483
	Gh_A03G2057	47.19	570	274	8	13	568	20	576	4.00E-136	483
	Gh_A10G2140	46.22	543	264	7	42	568	39	569	9.00E-136	481
	Gh_A08G2350	48.71	542	250	9	42	568	49	577	1.00E-135	481
	Gh_D10G0895	45.39	564	285	9	13	568	14	562	4.00E-135	479

Gh_D13G2373	46.94	539	266	9	40	568	49	577	1.00E-134	478
Gh_D03G1367	46.14	570	280	7	13	568	20	576	1.00E-134	478
Gh_A10G0858	45.39	564	285	9	13	568	14	562	3.00E-134	476
Gh_D08G2159	48.34	542	252	9	42	568	49	577	7.00E-134	475
Gh_A13G1977	46.94	539	266	9	40	568	49	577	9.00E-134	474
Gh_D13G0253	44.35	566	290	7	13	568	13	563	9.00E-133	471
Gh_A13G0237	44.17	566	291	7	13	568	13	563	1.00E-132	471
Gh_D11G3307	44.85	544	275	6	40	568	50	583	2.00E-132	470
Gh_D11G3322	44.72	568	294	8	7	568	3	556	1.00E-131	468
Gh_A03G0583	43.97	564	291	8	12	568	16	561	2.00E-131	467
Gh_D13G0194	46.22	543	268	7	40	568	44	576	2.00E-131	467
Gh_A13G0216	46.04	543	269	7	40	568	44	576	3.00E-131	466
Gh_D04G1243	44.56	561	291	7	14	568	10	556	3.00E-131	466
Gh_A11G2922	44.49	544	277	6	40	568	50	583	5.00E-131	466
Gh_A11G2936	44.37	568	296	8	7	568	3	556	2.00E-130	464
Gh_D13G0231	45.67	543	271	7	40	568	44	576	5.00E-130	462
Gh_D05G0888	42.88	562	299	7	13	568	11	556	6.00E-130	462
Gh_D05G2912	45.2	562	286	9	13	568	10	555	1.00E-129	461
Gh_A05G0758	42.43	568	305	7	7	568	5	556	1.00E-129	461
Gh_A05G0849	45.6	546	270	7	40	568	44	579	2.00E-129	460
Gh_A05G2622	45.07	568	285	10	12	568	4	555	3.00E-129	460
Gh_D01G2166	45.94	542	270	8	40	568	42	573	3.00E-129	460
Gh_D03G1181	47.15	543	262	10	40	568	46	577	3.00E-129	459
Gh_D05G3888	45.42	546	271	7	40	568	44	579	4.00E-129	459
Gh_A11G2920	44.5	573	291	9	12	568	9	570	6.00E-129	459
Gh_D03G1180	46.85	540	264	9	42	568	48	577	8.00E-129	458
Gh_A03G2082	46.59	543	265	10	40	568	46	577	3.00E-127	453
Gh_D03G1128	43.06	562	297	8	13	568	12	556	8.00E-127	452
Gh_D03G0865	43.44	564	290	9	12	568	16	557	1.00E-126	451
Gh_D10G2466	44.23	563	264	9	40	568	36	582	3.00E-125	446
Gh_A05G2631	46.1	538	240	9	42	568	12	510	5.00E-125	446
Gh_A01G1905	45.02	542	275	8	40	568	42	573	5.00E-125	446
Gh_A03G0417	42.7	562	299	8	13	568	12	556	8.00E-125	445
Gh_D11G3305	43.8	573	294	10	12	568	9	569	3.00E-124	443
Gh_D01G2209	43.62	564	296	9	12	568	10	558	3.00E-122	436
Gh_A01G1948	43.97	564	294	9	12	568	10	558	6.00E-122	436
Gh_A05G2099	43.92	567	299	7	13	568	16	574	1.00E-121	434
Gh_D05G2353	43.18	572	302	7	12	568	15	578	1.00E-119	428
Gh_D11G1042	42.57	538	288	7	40	568	49	574	2.00E-118	424
Gh_D11G1874	42.43	535	274	9	40	554	20	540	4.00E-112	403
Gh_D12G1138	39.71	559	293	8	42	568	49	595	1.00E-111	401
Gh_A12G1019	39.36	559	295	8	42	568	49	595	4.00E-110	396
Gh_D05G2354	40.46	566	313	10	13	568	11	562	3.00E-109	393
Gh_A03G2084	44.36	505	233	9	40	526	46	520	1.00E-108	391
Gh_A02G0638	39.1	578	327	9	12	575	6	572	1.00E-108	391
Gh_D05G2356	40.21	567	316	10	13	568	5	559	1.00E-107	388
Gh_A05G0853	41.62	531	240	7	42	568	47	511	7.00E-107	385
Gh_A05G2115	40.07	564	314	9	13	568	16	563	2.00E-106	384
Gh_D02G0685	38.79	580	325	11	15	577	9	575	5.00E-106	383
Gh_A05G2113	39.89	569	316	12	13	568	5	560	7.00E-106	382
Gh_A05G2116	41	539	293	10	40	568	40	563	3.00E-105	380
Gh_A13G0179	40.15	543	253	8	40	568	44	528	1.00E-104	378
Gh_A05G2117	38.49	569	326	9	13	570	7	562	2.00E-104	377
Gh_D06G1188	39.26	540	306	9	40	569	42	569	1.00E-100	365
Gh_A06G0997	39.11	542	304	10	40	569	42	569	3.00E-100	363
Gh_A05G2100	40	525	291	10	13	527	11	521	2.00E-95	348
Gh_Sca005020G01	36.97	541	266	10	42	566	47	528	2.00E-93	341
Gh_D03G1177	40.49	489	224	13	40	516	44	477	3.00E-88	323
Gh_A13G2102	38.58	521	242	10	13	525	15	465	5.00E-86	316
Gh_A13G2103	39.75	478	250	10	114	568	34	496	6.00E-85	312
Gh_D06G2354	44.2	371	186	5	207	568	15	373	9.00E-83	305
Gh_D05G2921	43.51	370	173	7	42	400	12	356	1.00E-71	268
Gh_A11G0894	44.78	335	173	3	40	368	43	371	6.00E-70	263

	Gh_D13G2551	46.55	290	144	2	8	286	7	296	7.00E-68	256
	Gh_D13G2551	47.78	180	86	3	391	568	283	456	1.00E-43	176
	Gh_A13G0218	51.75	257	116	3	40	288	44	300	3.00E-65	247
	Gh_A13G0218	48.09	131	61	2	396	526	311	434	9.00E-29	126
	Gh_D06G1759	40.6	367	186	7	12	368	8	352	2.00E-63	241
	Gh_A12G0840	33.63	556	299	21	40	560	38	558	1.00E-62	238
	Gh_D13G0232	42.12	330	164	6	205	526	14	324	1.00E-61	235
	Gh_D03G0412	32.48	588	315	22	14	560	25	571	1.00E-60	232
	Gh_D12G0913	32.5	563	295	21	40	560	38	557	4.00E-60	230
	Gh_A02G1272	32.02	584	323	21	14	560	25	571	7.00E-60	229
	Gh_A08G1751	29.9	592	328	20	13	560	13	561	9.00E-57	219
	Gh_D08G2100	30.22	579	317	20	13	546	13	549	1.00E-56	218
	Gh_A11G1716	48.55	241	117	2	56	290	3	242	2.00E-53	208
	Gh_D04G1220	54.1	183	78	2	14	190	12	194	6.00E-50	196
	Gh_A06G1412	46.24	186	94	1	383	568	1	180	8.00E-49	192
	Gh_D13G0328	42.72	213	112	2	23	229	27	235	3.00E-45	181
	Gh_D13G0328	51.69	118	57	0	451	568	210	327	1.00E-33	142
	Gh_A05G2112	36.6	306	171	6	11	310	6	294	9.00E-44	176
	Gh_D05G2355	35.57	298	171	5	8	304	11	288	3.00E-43	174
	Gh_D05G2589	38.02	263	142	5	40	289	41	295	7.00E-43	172
**AtLAC10**	Gh_D04G1243	75.99	558	132	2	1	558	1	556	0	852
	Gh_D11G3322	75.99	554	131	2	5	558	5	556	0	851
	Gh_A11G2936	75.95	553	131	2	6	558	6	556	0	849
	Gh_D05G0888	73.83	554	142	2	5	558	6	556	0	833
	Gh_A05G0758	73.29	554	145	2	5	558	6	556	0	827
	Gh_D03G1128	72.02	554	151	3	5	558	7	556	0	812
	Gh_A01G1948	72.2	554	153	1	5	558	6	558	0	812
	Gh_D01G2209	71.66	554	156	1	5	558	6	558	0	812
	Gh_A03G0417	71.66	554	153	3	5	558	7	556	0	807
	Gh_A05G2622	66.42	551	182	2	8	558	8	555	0	753
	Gh_D05G2912	66.06	551	184	2	8	558	8	555	0	752
	Gh_D10G2466	63.82	586	180	5	1	558	1	582	0	741
	Gh_A13G0237	61.4	557	209	4	3	558	12	563	0	689
	Gh_D10G0895	60.73	550	210	4	10	558	18	562	0	689
	Gh_A10G0858	60.73	550	210	4	10	558	18	562	0	687
	Gh_D13G0253	60.86	557	212	4	3	558	12	563	0	686
	Gh_A03G0583	61.68	548	200	6	13	558	22	561	0	668
	Gh_D03G0865	60.95	548	200	8	13	558	22	557	0	657
	Gh_D09G1454	55.52	571	239	6	1	558	15	583	0	633
	Gh_A09G1445	54.82	571	243	6	1	558	15	583	0	631
	Gh_A05G0849	55.42	563	237	5	10	558	17	579	8.00E-180	628
	Gh_D11G3307	56.91	550	223	5	22	558	35	583	8.00E-180	627
	Gh_D13G0194	55.38	567	237	6	7	558	11	576	1.00E-179	627
	Gh_A11G2922	56.73	550	224	5	22	558	35	583	3.00E-179	626
	Gh_D05G3888	55.24	563	238	5	10	558	17	579	3.00E-179	625
	Gh_A13G0216	55.54	551	232	5	20	558	27	576	3.00E-178	622
	Gh_D13G0231	55.35	551	233	5	20	558	27	576	1.00E-177	620
	Gh_D13G2373	53.53	566	247	6	7	558	14	577	6.00E-174	608
	Gh_D03G1181	53.31	574	248	8	4	558	5	577	2.00E-173	607
	Gh_D03G1180	53.95	569	246	7	5	558	10	577	2.00E-173	606
	Gh_A03G2082	53.14	574	249	8	4	558	5	577	1.00E-172	603
	Gh_A11G2920	52.92	565	252	5	7	558	7	570	2.00E-172	603
	Gh_A13G1977	53	566	250	6	7	558	14	577	3.00E-172	603
	Gh_D01G2166	54.46	549	238	6	21	558	26	573	6.00E-172	602
	Gh_A01G1905	52.93	563	251	7	7	558	14	573	4.00E-168	589
	Gh_D11G3305	51.5	565	259	6	7	558	7	569	3.00E-166	582
	Gh_A06G1415	50.7	570	259	6	3	558	7	568	2.00E-162	570
	Gh_D06G1762	50.35	570	261	6	3	558	7	568	3.00E-161	566
	Gh_A06G1413	50.35	570	261	6	3	558	7	568	3.00E-160	562
	Gh_A10G2140	49.56	569	267	6	6	558	5	569	2.00E-158	556
	Gh_D10G2461	48.86	569	271	6	6	558	5	569	2.00E-157	553
	Gh_A03G2057	49.91	565	263	8	7	558	19	576	2.00E-153	540
	Gh_D03G1367	49.38	565	266	7	7	558	19	576	3.00E-153	540

	Gh_D04G1224	51.1	546	258	3	22	558	22	567	5.00E-152	535
	Gh_A04G0744	50.92	546	259	3	22	558	22	567	8.00E-152	535
	Gh_A13G0179	50.27	551	213	6	20	558	27	528	1.00E-151	534
	Gh_D11G1042	47.64	573	279	5	1	558	8	574	2.00E-150	530
	Gh_A08G2350	50.46	549	253	8	24	558	34	577	5.00E-150	528
	Gh_D08G2159	50.46	549	253	8	24	558	34	577	3.00E-149	526
	Gh_A03G2084	52.08	528	226	8	4	516	5	520	3.00E-148	523
	Gh_A05G0853	49.27	550	216	7	13	558	21	511	1.00E-147	521
	Gh_D12G1138	45.76	590	274	6	9	558	12	595	3.00E-147	519
	Gh_A12G1019	45.93	590	273	6	9	558	12	595	3.00E-146	516
	Gh_A13G2215	48.25	543	270	4	24	558	25	564	4.00E-140	496
	Gh_A05G2099	47.4	576	283	6	1	558	1	574	8.00E-140	494
	Gh_D13G2524	47.04	540	281	2	24	558	25	564	2.00E-138	490
	Gh_D05G2353	46.89	578	287	5	1	558	1	578	4.00E-138	489
	Gh_A05G2631	47.84	533	237	6	36	558	9	510	1.00E-136	484
	Gh_A04G0743	45.01	571	296	6	6	558	3	573	3.00E-134	476
	Gh_D11G1874	46	537	265	5	30	547	13	543	4.00E-133	473
	Gh_A13G2102	50	510	194	6	7	515	16	465	7.00E-133	471
	Gh_A13G2103	57.56	410	157	4	163	558	90	496	2.00E-132	470
	Gh_A13G2103	50.65	77	36	1	7	83	16	90	4.00E-11	67.8
	Gh_D04G1223	44.99	569	296	7	6	558	14	581	6.00E-132	469
	Gh_Sca005020G01	44.13	562	258	7	5	556	13	528	7.00E-131	465
	Gh_A05G2115	43.83	559	302	7	5	558	12	563	1.00E-129	461
	Gh_A05G2116	43.15	540	299	6	24	558	27	563	2.00E-128	456
	Gh_D05G2354	41.73	556	317	4	5	558	12	562	3.00E-128	456
	Gh_D04G1221	44.22	554	291	6	6	541	3	556	1.00E-126	451
	Gh_A05G2113	42.14	560	313	6	5	558	6	560	3.00E-126	449
	Gh_A05G2117	42.11	558	314	5	5	558	8	560	3.00E-125	446
	Gh_D05G2356	41.68	559	316	6	5	558	6	559	8.00E-125	445
	Gh_A02G0638	40.21	562	327	6	3	558	7	565	3.00E-123	439
	Gh_D02G0685	40.21	562	326	7	4	558	8	566	4.00E-122	436
	Gh_D03G1177	44.13	528	225	11	4	511	5	482	6.00E-113	405
	Gh_D06G1188	40.39	562	320	9	7	558	12	568	5.00E-112	402
	Gh_A06G0997	39.57	561	326	9	7	558	12	568	2.00E-111	400
	Gh_A05G2100	40.47	514	299	4	5	516	12	520	2.00E-109	394
	Gh_D13G2551	59.73	293	114	2	4	294	11	301	3.00E-100	363
	Gh_D13G2551	67.92	159	50	1	401	558	298	456	1.00E-61	235
	Gh_D06G2354	49.04	363	167	5	210	558	15	373	5.00E-96	349
	Gh_A11G0894	48.99	347	173	1	22	368	28	370	3.00E-91	333
	Gh_A13G0218	57.19	292	120	3	20	307	27	317	4.00E-91	333
	Gh_A13G0218	58.73	126	51	1	392	516	309	434	3.00E-39	160
	Gh_D05G2921	42.82	369	183	5	36	397	9	356	2.00E-82	304
	Gh_D13G0232	47.35	321	147	6	208	516	14	324	5.00E-77	286
	Gh_D06G1759	41.08	370	185	5	3	366	7	349	6.00E-71	266
	Gh_D13G0328	57.01	221	85	2	23	243	30	240	3.00E-66	250
	Gh_D13G0328	65	140	47	1	421	558	188	327	9.00E-49	192
	Gh_A11G1716	48.63	255	130	1	52	306	2	255	2.00E-63	241
	Gh_D12G0913	33.64	544	315	16	20	536	21	545	6.00E-62	236
	Gh_A06G1412	61.11	180	68	2	381	558	1	180	3.00E-60	230
	Gh_A08G1751	30.89	560	338	16	5	536	11	549	2.00E-53	208
	Gh_D08G2100	30.89	560	338	16	5	536	11	549	2.00E-53	207
	Gh_A11G0895	52.75	182	80	2	381	558	1	180	1.00E-50	198
	Gh_D05G2355	38.85	278	154	5	8	285	14	275	3.00E-48	191
	Gh_A05G2165	28.04	535	353	11	25	558	27	530	1.00E-47	188
	Gh_D05G2589	40.93	259	140	3	27	285	31	276	1.00E-47	188
	Gh_D04G1220	47.64	191	96	1	6	192	3	193	2.00E-47	188
	Gh_D05G2420	27.82	550	361	12	10	558	16	530	2.00E-47	188
**AtLAC11**	Gh_D10G0895	79.18	538	112	0	20	557	25	562	0	924
	Gh_A10G0858	78.81	538	114	0	20	557	25	562	0	920
	Gh_A03G0583	80.19	540	103	2	19	557	25	561	0	917
	Gh_A13G0237	79	538	113	0	20	557	26	563	0	917
	Gh_D13G0253	77.7	538	120	0	20	557	26	563	0	907
	Gh_D03G0865	79.26	540	104	3	19	557	25	557	0	904

Gh_D05G0888	64.01	539	193	1	19	557	19	556	0	738
Gh_A05G0758	63.64	539	195	1	19	557	19	556	0	733
Gh_D03G1128	62.57	537	199	2	21	557	22	556	0	723
Gh_D11G3322	62.34	539	200	2	21	557	19	556	0	723
Gh_D04G1243	61.97	539	202	2	21	557	19	556	0	721
Gh_A03G0417	62.2	537	201	2	21	557	22	556	0	718
Gh_A11G2936	61.6	539	204	2	21	557	19	556	0	715
Gh_D10G2466	57.5	567	208	3	21	557	19	582	0	679
Gh_D01G2209	60.3	539	211	2	21	557	21	558	0	678
Gh_A01G1948	60.3	539	211	2	21	557	21	558	0	677
Gh_D11G3307	58.68	547	210	8	26	557	38	583	0	674
Gh_A11G2922	58.32	547	212	8	26	557	38	583	0	671
Gh_D05G2912	59.18	539	219	1	19	557	18	555	0	671
Gh_D09G1454	58.5	547	211	8	26	557	38	583	0	670
Gh_A05G2622	59.3	543	212	3	19	557	18	555	0	669
Gh_A13G0216	58.33	552	215	8	20	557	26	576	0	669
Gh_D05G3888	58.38	555	213	8	20	557	26	579	0	669
Gh_A09G1445	58.14	547	213	8	26	557	38	583	0	668
Gh_D13G0194	59.06	552	211	8	20	557	26	576	0	667
Gh_A05G0849	58.02	555	215	8	20	557	26	579	0	665
Gh_D13G0231	58.33	552	215	8	20	557	26	576	0	664
Gh_A13G2102	68.28	495	102	4	20	514	26	465	0	651
Gh_A13G2103	76.87	415	83	1	156	557	82	496	0	645
Gh_A13G2103	81.54	65	12	0	20	84	26	90	8.00E-27	119
Gh_D13G2373	56.54	543	223	6	26	557	37	577	0	642
Gh_A13G1977	56.27	542	226	5	26	557	37	577	0	635
Gh_D03G1181	57.14	546	218	9	26	557	34	577	0	633
Gh_A03G2082	56.41	546	222	9	26	557	34	577	1.00E-179	627
Gh_D03G1180	56.78	546	220	9	26	557	34	577	2.00E-179	626
Gh_A11G2920	54.93	548	229	7	26	557	25	570	1.00E-177	620
Gh_D01G2166	55.64	550	228	8	22	557	26	573	1.00E-177	620
Gh_A01G1905	54.91	550	232	8	22	557	26	573	4.00E-174	609
Gh_D11G3305	53.83	548	234	8	26	557	25	569	8.00E-172	601
Gh_A06G1415	51.45	552	248	6	22	557	21	568	2.00E-169	593
Gh_D06G1762	51.27	552	249	6	22	557	21	568	6.00E-169	592
Gh_A03G2057	51.09	550	251	5	22	557	31	576	1.00E-167	587
Gh_D03G1367	51.18	551	251	5	21	557	30	576	2.00E-167	586
Gh_D10G2461	51.62	554	247	6	21	557	20	569	3.00E-167	586
Gh_A10G2140	51.99	554	245	6	21	557	20	569	7.00E-167	585
Gh_A06G1413	50.91	552	251	6	22	557	21	568	3.00E-166	582
Gh_A13G0179	52.9	552	197	10	20	557	26	528	1.00E-162	570
Gh_A08G2350	50.36	548	253	6	25	557	34	577	2.00E-162	570
Gh_D08G2159	50.36	548	253	6	25	557	34	577	3.00E-162	569
Gh_A04G0744	49.91	547	263	5	22	557	21	567	1.00E-158	557
Gh_D04G1224	49.36	547	266	5	22	557	21	567	1.00E-158	557
Gh_A03G2084	56.55	504	188	12	26	515	34	520	3.00E-157	553
Gh_D11G1042	49.36	551	259	8	20	557	31	574	1.00E-154	543
Gh_A05G0853	50.74	544	203	8	20	557	27	511	4.00E-152	536
Gh_D12G1138	47.57	576	255	12	20	557	29	595	5.00E-150	529
Gh_A05G2099	48.91	552	270	5	18	557	23	574	2.00E-148	523
Gh_A12G1019	47.47	573	254	12	23	557	32	595	2.00E-148	523
Gh_A05G2631	49.26	540	229	10	31	557	3	510	1.00E-147	521
Gh_D13G2524	48.15	542	274	4	23	557	23	564	1.00E-146	518
Gh_D05G2353	48.38	556	271	5	18	557	23	578	4.00E-146	516
Gh_A13G2215	47.96	540	274	4	25	557	25	564	1.00E-145	514
Gh_A04G0743	46.84	553	278	5	21	557	21	573	1.00E-143	508
Gh_Sca005020G01	47.83	552	222	9	20	555	27	528	3.00E-142	503
Gh_D11G1874	47.74	530	254	7	31	541	13	538	2.00E-141	500
Gh_D04G1223	47.09	550	278	6	21	557	32	581	6.00E-141	498
Gh_D04G1221	45.71	536	275	5	21	540	21	556	2.00E-139	494
Gh_D13G2551	81.39	274	51	0	20	293	26	299	7.00E-139	491
Gh_D13G2551	82.39	159	28	0	399	557	298	456	3.00E-78	290
Gh_D05G2354	43.68	538	300	2	23	557	25	562	7.00E-136	481

	Gh_A02G0638	42.65	544	305	5	21	557	22	565	8.00E-134	474
	Gh_D05G2356	43.07	541	302	4	23	557	19	559	2.00E-133	473
	Gh_A05G2113	43.17	542	301	4	23	557	19	560	1.00E-132	471
	Gh_A05G2115	42.59	540	306	3	22	557	24	563	6.00E-132	469
	Gh_A05G2117	42.96	540	303	3	23	557	21	560	1.00E-131	468
	Gh_D02G0685	42.2	545	307	6	21	557	22	566	2.00E-131	466
	Gh_A05G2116	42.12	539	308	3	23	557	25	563	1.00E-130	464
	Gh_D06G1188	41.58	546	304	8	23	557	27	568	8.00E-126	448
	Gh_A06G0997	41.39	546	305	8	23	557	27	568	5.00E-125	446
	Gh_D03G1177	47.8	500	193	11	28	510	34	482	9.00E-125	445
	Gh_A05G2100	42.94	496	280	2	23	515	25	520	2.00E-118	424
	Gh_A13G0218	58.54	287	117	2	20	304	26	312	2.00E-101	367
	Gh_A13G0218	62.99	127	45	2	390	515	309	434	3.00E-39	160
	Gh_D06G2354	50.41	367	162	6	207	557	11	373	5.00E-101	366
	Gh_A11G0894	50	350	170	2	20	368	25	370	4.00E-98	356
	Gh_D13G0328	59.03	310	108	5	20	320	26	325	4.00E-95	346
	Gh_D13G0328	76.64	137	32	0	421	557	191	327	1.00E-57	222
	Gh_D05G2921	45.48	376	170	6	32	395	4	356	4.00E-92	336
	Gh_D13G0232	53.42	322	126	9	208	515	13	324	2.00E-87	321
	Gh_D06G1759	44.07	354	168	5	21	367	20	350	1.00E-83	308
	Gh_A02G1272	35.22	548	304	15	19	535	32	559	8.00E-75	279
	Gh_D03G0412	35.27	550	301	15	19	535	32	559	1.00E-73	275
	Gh_A11G1716	53.33	255	117	2	52	305	1	254	1.00E-71	268
	Gh_A12G0840	32.54	544	322	14	21	536	21	547	2.00E-70	264
	Gh_D12G0913	33.27	550	307	16	21	535	21	545	2.00E-69	261
	Gh_D08G2100	30.23	569	333	20	23	557	28	566	1.00E-63	241
	Gh_A08G1751	30.84	548	320	19	23	536	28	550	2.00E-63	241
	Gh_A06G1412	56.11	180	78	1	379	557	1	180	2.00E-57	221
	Gh_D05G2355	38.26	264	150	4	23	286	25	275	2.00E-50	198
	Gh_A05G2112	38.17	262	149	3	23	284	25	273	3.00E-50	197
	Gh_A05G2114	38.26	264	150	4	23	286	25	275	3.00E-50	197
**AtLAC12**	Gh_D06G1762	80.11	548	102	2	24	565	22	568	0	936
	Gh_A06G1415	79.74	548	104	2	24	565	22	568	0	934
	Gh_A06G1413	79.56	548	105	2	24	565	22	568	0	928
	Gh_D10G2461	79.05	549	107	2	24	565	22	569	0	917
	Gh_A10G2140	78.69	549	109	2	24	565	22	569	0	914
	Gh_A05G2631	74.39	535	107	3	33	565	4	510	0	830
	Gh_D08G2159	66.73	547	172	5	26	565	34	577	0	764
	Gh_A03G2057	65.94	549	176	5	24	565	32	576	0	760
	Gh_D03G1367	65.94	549	176	5	24	565	32	576	0	757
	Gh_A08G2350	66.24	548	173	6	26	565	34	577	0	757
	Gh_A13G0237	54.95	546	231	6	24	565	29	563	3.00E-177	619
	Gh_D13G0253	54.58	546	233	6	24	565	29	563	7.00E-177	618
	Gh_D04G1224	52.46	549	251	3	24	565	22	567	6.00E-173	605
	Gh_D06G2354	79.67	364	67	2	208	565	11	373	7.00E-173	605
	Gh_D09G1454	53.16	553	238	9	27	565	38	583	6.00E-172	601
	Gh_A04G0744	52.28	549	252	3	24	565	22	567	1.00E-171	600
	Gh_A09G1445	52.62	553	241	9	27	565	38	583	2.00E-171	600
	Gh_D10G0895	52.56	546	244	6	24	565	28	562	8.00E-171	598
	Gh_A10G0858	52.38	546	245	6	24	565	28	562	8.00E-170	594
	Gh_A13G2215	54.2	548	237	5	25	565	24	564	3.00E-169	593
	Gh_A04G0743	53.33	555	242	6	24	565	23	573	3.00E-168	589
	Gh_D13G2524	53.55	549	241	5	24	565	23	564	2.00E-167	587
	Gh_A13G1977	53.65	548	238	8	27	565	37	577	2.00E-167	586
	Gh_D04G1243	52.39	544	245	5	26	565	23	556	1.00E-166	584
	Gh_A03G0583	52.47	547	241	8	24	565	29	561	3.00E-166	582
	Gh_D11G3307	52.62	553	241	9	27	565	38	583	9.00E-166	581
	Gh_A11G2922	52.44	553	242	9	27	565	38	583	3.00E-165	579
	Gh_D11G3322	51.47	544	250	5	26	565	23	556	8.00E-165	578
	Gh_D03G1180	53.54	551	237	10	27	565	34	577	1.00E-164	577
	Gh_D13G2373	52.55	548	244	8	27	565	37	577	1.00E-164	577
	Gh_D13G0194	51.99	552	245	10	27	565	32	576	4.00E-164	575
	Gh_A11G2920	51.7	559	243	9	24	565	22	570	8.00E-164	574

Gh_D04G1223	53.54	551	244	5	24	565	34	581	1.00E-163	574
Gh_D03G1181	53.18	551	239	10	27	565	34	577	2.00E-163	573
Gh_D01G2166	52.35	554	245	9	24	565	27	573	7.00E-163	571
Gh_A11G2936	50.92	544	253	5	26	565	23	556	9.00E-163	571
Gh_D03G1128	51.65	544	246	7	26	565	26	556	2.00E-162	570
Gh_A03G2082	52.81	551	241	10	27	565	34	577	2.00E-162	570
Gh_D03G0865	52.1	547	239	9	24	565	29	557	2.00E-162	570
Gh_A13G0216	51.09	552	250	9	27	565	32	576	3.00E-162	569
Gh_D04G1221	52.23	538	240	6	24	548	23	556	9.00E-162	568
Gh_A03G0417	51.29	544	248	7	26	565	26	556	2.00E-161	567
Gh_D05G0888	51.1	544	250	6	26	565	25	556	2.00E-161	566
Gh_A05G0758	50.92	544	251	6	26	565	25	556	4.00E-161	566
Gh_D13G0231	50.91	552	251	9	27	565	32	576	5.00E-161	565
Gh_A01G1905	52.17	554	246	9	24	565	27	573	5.00E-161	565
Gh_D05G3888	50.63	555	251	9	27	565	32	579	4.00E-160	562
Gh_A05G0849	50.27	555	253	9	27	565	32	579	2.00E-159	560
Gh_D10G2466	49.48	574	246	6	24	565	21	582	1.00E-158	557
Gh_A01G1948	51.47	546	251	5	24	565	23	558	3.00E-158	556
Gh_D01G2209	51.28	546	252	5	24	565	23	558	4.00E-158	555
Gh_D11G3305	50.9	556	251	9	24	565	22	569	4.00E-158	555
Gh_A05G2099	51	549	256	4	26	565	30	574	6.00E-158	555
Gh_D11G1042	50.09	539	264	3	29	565	39	574	2.00E-155	546
Gh_D05G2353	49.37	553	263	4	26	565	30	578	4.00E-155	545
Gh_D05G2912	49.08	546	262	6	24	565	22	555	9.00E-155	544
Gh_A05G2622	49.08	546	262	6	24	565	22	555	9.00E-155	544
Gh_A12G1019	47.34	564	269	4	27	565	35	595	7.00E-152	535
Gh_D12G1138	46.81	564	272	4	27	565	35	595	2.00E-151	533
Gh_D05G2921	68.36	373	97	2	32	402	3	356	3.00E-151	533
Gh_A03G2084	52.47	507	211	12	27	523	34	520	2.00E-141	500
Gh_D06G1759	70.11	348	79	3	24	365	22	350	5.00E-140	495
Gh_D05G2354	44.71	548	287	6	24	565	25	562	7.00E-137	485
Gh_D11G1874	46.96	543	252	8	33	553	14	542	1.00E-136	484
Gh_A02G0638	43.38	551	294	9	24	565	24	565	2.00E-136	483
Gh_A13G0179	46.56	552	227	11	27	565	32	528	5.00E-136	482
Gh_A05G2117	42.96	547	300	6	24	565	21	560	7.00E-134	475
Gh_D02G0685	42.39	552	299	10	24	565	24	566	7.00E-133	472
Gh_A05G2115	43.01	551	293	8	24	565	25	563	8.00E-133	471
Gh_D05G2356	43.07	548	299	7	24	565	19	559	1.00E-132	471
Gh_A05G2113	42.5	553	296	7	24	565	19	560	3.00E-130	463
Gh_A05G0853	45.04	544	229	8	27	565	33	511	4.00E-130	462
Gh_A05G2116	41.92	551	299	8	24	565	25	563	4.00E-129	459
Gh_D06G1188	42.57	552	297	11	24	565	27	568	2.00E-128	457
Gh_A06G0997	42.86	553	294	12	24	565	27	568	2.00E-127	454
Gh_Sca005020G01	43.01	551	245	9	27	563	33	528	2.00E-124	444
Gh_A05G2100	44.66	506	264	6	24	523	25	520	1.00E-122	437
Gh_A13G2102	45.73	503	203	8	24	522	29	465	2.00E-113	407
Gh_D03G1177	46.25	506	199	14	24	512	27	476	7.00E-113	405
Gh_A13G2103	50.72	418	178	7	165	565	90	496	1.00E-110	397
Gh_A06G1412	85.64	181	25	1	385	565	1	180	1.00E-90	331
Gh_D13G2551	54.48	268	122	0	24	291	29	296	8.00E-89	325
Gh_D13G2551	57.59	158	66	1	408	565	300	456	1.00E-51	202
Gh_A11G0894	45.77	343	186	0	27	369	31	373	2.00E-88	324
Gh_A13G0218	51.17	299	143	2	27	323	32	329	4.00E-88	323
Gh_A13G0218	52.34	128	58	2	397	523	309	434	5.00E-32	137
Gh_D13G0232	45.78	332	145	11	208	523	12	324	5.00E-72	270
Gh_A11G1716	47.88	259	134	1	53	311	1	258	1.00E-65	248
Gh_D08G2100	31.49	562	322	20	24	552	28	559	4.00E-64	243
Gh_D12G0913	32.38	559	303	20	24	543	23	545	4.00E-64	243
Gh_A08G1751	31.52	552	316	19	24	543	28	549	7.00E-64	242
Gh_A12G0840	31.83	553	315	19	24	543	23	546	3.00E-63	240
Gh_D03G0412	32.26	558	306	21	24	543	36	559	2.00E-61	234
Gh_D13G0328	53.23	201	90	1	24	224	29	225	3.00E-61	234
Gh_D13G0328	61.54	117	45	0	449	565	211	327	2.00E-41	168

	Gh_A02G1272	32.08	558	307	21	24	543	36	559	9.00E-61	232
	Gh_D05G2355	38.97	290	155	6	24	312	25	293	7.00E-56	216
	Gh_D04G1220	57.31	171	73	0	24	194	23	193	7.00E-56	216
	Gh_A05G2114	38.97	290	155	6	24	312	25	293	1.00E-55	215
	Gh_A11G0895	57.92	183	72	3	385	565	1	180	5.00E-55	213
**AtLAC13**	Gh_A03G2057	74.33	561	140	4	9	569	20	576	0	806
	Gh_D03G1367	74.15	561	141	4	9	569	20	576	0	798
	Gh_A08G2350	74.78	563	137	5	8	569	19	577	0	781
	Gh_D08G2159	74.25	563	140	5	8	569	19	577	0	775
	Gh_A06G1415	67.08	565	178	7	8	569	9	568	0	708
	Gh_D06G1762	66.9	565	179	7	8	569	9	568	0	707
	Gh_A10G2140	65.62	573	189	7	1	569	1	569	0	702
	Gh_A06G1413	66.55	565	181	7	8	569	9	568	0	701
	Gh_D10G2461	65.62	573	189	7	1	569	1	569	0	697
	Gh_A05G2631	61.88	543	168	8	30	569	4	510	7.00E-169	591
	Gh_A09G1445	48.27	578	281	9	4	569	12	583	2.00E-146	516
	Gh_D09G1454	48.44	578	280	9	4	569	12	583	6.00E-146	515
	Gh_A13G0237	50.18	570	259	10	7	569	12	563	1.00E-142	504
	Gh_D11G3307	49.45	550	266	7	26	569	40	583	2.00E-142	503
	Gh_D13G0253	50	570	260	10	7	569	12	563	4.00E-142	503
	Gh_A11G2922	49.45	550	266	7	26	569	40	583	4.00E-142	502
	Gh_D10G0895	49.56	567	264	9	7	569	14	562	5.00E-141	499
	Gh_D04G1224	48.93	562	268	7	16	569	17	567	2.00E-140	497
	Gh_A10G0858	49.38	567	265	9	7	569	14	562	7.00E-140	495
	Gh_A04G0744	47.86	562	274	7	16	569	17	567	9.00E-139	491
	Gh_A13G1977	50	550	258	8	26	569	39	577	5.00E-138	489
	Gh_D04G1223	49.48	582	271	11	1	569	10	581	8.00E-138	488
	Gh_D11G3322	48.33	569	281	8	1	569	1	556	1.00E-137	488
	Gh_D13G2373	49.82	550	259	8	26	569	39	577	1.00E-137	487
	Gh_D04G1243	48.5	565	272	9	8	569	8	556	2.00E-137	486
	Gh_D05G3888	47.31	577	284	10	6	569	10	579	3.00E-137	486
	Gh_A05G0849	46.97	577	286	10	6	569	10	579	8.00E-137	484
	Gh_A13G2215	49.09	552	258	10	26	569	28	564	5.00E-136	482
	Gh_A11G2936	47.1	586	263	9	1	569	1	556	7.00E-136	481
	Gh_D03G1180	49.73	551	261	9	26	569	36	577	2.00E-135	480
	Gh_D13G0194	46.83	568	288	9	8	569	17	576	2.00E-135	480
	Gh_A13G0216	47.27	550	277	8	26	569	34	576	8.00E-135	478
	Gh_D13G0231	48	550	273	8	26	569	34	576	1.00E-134	478
	Gh_D03G1181	49.73	551	261	9	26	569	36	577	1.00E-134	477
	Gh_D13G2524	48.29	557	265	10	21	569	23	564	2.00E-134	477
	Gh_A04G0743	49.13	574	266	10	10	569	12	573	5.00E-134	476
	Gh_A03G0583	48.78	574	265	11	4	569	9	561	6.00E-134	475
	Gh_D01G2166	47.89	570	279	10	9	569	13	573	4.00E-133	473
	Gh_A03G2082	49.18	551	264	9	26	569	36	577	1.00E-132	471
	Gh_A11G2920	47.45	569	284	10	8	569	10	570	8.00E-132	468
	Gh_A03G0417	45.61	570	286	9	4	569	7	556	1.00E-131	467
	Gh_D03G1128	45.61	570	286	9	4	569	7	556	5.00E-131	465
	Gh_D11G1042	45.65	552	278	8	25	569	38	574	7.00E-131	465
	Gh_A05G2099	47.36	549	277	7	26	569	33	574	1.00E-130	464
	Gh_D05G0888	47.78	563	279	9	7	569	9	556	3.00E-130	463
	Gh_D03G0865	46.1	577	272	12	4	569	9	557	3.00E-130	463
	Gh_A05G0758	47.25	563	282	9	7	569	9	556	4.00E-130	462
	Gh_A12G1019	42.88	597	293	9	7	569	13	595	6.00E-130	462
	Gh_D05G2353	47.55	551	277	7	26	569	33	578	6.00E-130	462
	Gh_A01G1905	46.01	576	295	9	1	569	7	573	3.00E-129	460
	Gh_D11G3305	47.1	569	285	10	8	569	10	569	4.00E-129	459
	Gh_A01G1948	47.2	572	281	8	2	569	4	558	5.00E-129	459
	Gh_D01G2209	47.2	572	281	8	2	569	4	558	6.00E-129	459
	Gh_D10G2466	45.12	594	275	11	8	569	8	582	7.00E-129	458
	Gh_D12G1138	43.73	574	280	8	25	569	36	595	9.00E-129	458
	Gh_D04G1221	48.29	557	262	10	10	552	12	556	3.00E-128	456
	Gh_A05G2622	44.56	570	287	10	7	569	8	555	9.00E-125	445
	Gh_D05G2912	44.44	567	292	10	7	569	8	555	4.00E-124	442

	Gh_D05G2354	43.06	569	301	9	7	569	11	562	3.00E-118	423
	Gh_D05G2356	42.11	570	308	9	7	569	5	559	3.00E-116	416
	Gh_A05G2113	40.46	571	317	10	7	569	5	560	2.00E-114	410
	Gh_A03G2084	46.85	508	241	11	26	527	36	520	2.00E-113	407
	Gh_A05G2117	39.48	575	327	8	1	569	1	560	5.00E-113	405
	Gh_D06G2354	65.57	366	120	5	207	569	11	373	1.00E-112	405
	Gh_D05G2921	58.33	384	128	7	29	408	3	358	3.00E-111	400
	Gh_D06G1188	40	575	315	10	8	569	11	568	7.00E-111	399
	Gh_A05G2115	40.18	570	317	8	7	569	11	563	1.00E-110	398
	Gh_A06G0997	39.83	575	316	10	8	569	11	568	2.00E-110	397
	Gh_A13G0179	41.56	551	259	10	26	569	34	528	4.00E-110	396
	Gh_A05G0853	42.26	549	242	9	25	569	34	511	5.00E-110	395
	Gh_D11G1874	42.99	542	284	10	30	558	14	543	1.00E-109	395
	Gh_A02G0638	39.2	574	330	9	4	569	3	565	2.00E-109	394
	Gh_D02G0685	39.08	568	330	9	7	569	10	566	2.00E-109	394
	Gh_A05G2116	40.72	555	307	7	21	569	25	563	3.00E-109	393
	Gh_D06G1759	59.67	367	124	6	8	373	9	352	2.00E-107	387
	Gh_A05G2100	42.5	527	280	9	7	527	11	520	4.00E-103	373
	Gh_Sca005020G01	40.64	566	275	11	8	567	18	528	3.00E-100	363
	Gh_D03G1177	43.46	497	213	12	35	521	43	481	3.00E-88	323
	Gh_A13G2103	46.81	423	192	7	164	569	90	496	1.00E-85	315
	Gh_A13G2103	43.42	76	43	0	7	82	15	90	2.00E-11	68.6
	Gh_D13G2551	53.31	272	124	3	7	277	14	283	4.00E-80	296
	Gh_D13G2551	56.74	141	61	0	429	569	316	456	2.00E-45	181
	Gh_A13G2102	47.41	405	191	8	126	526	79	465	7.00E-80	296
	Gh_A13G0218	52.03	246	116	2	26	271	34	277	2.00E-74	278
	Gh_A13G0218	59.77	87	35	0	441	527	348	434	1.00E-27	122
	Gh_A11G0894	41.83	349	187	4	25	369	32	368	2.00E-72	271
	Gh_A06G1412	66.85	181	59	1	389	569	1	180	3.00E-60	230
	Gh_D13G0328	43.98	266	141	4	15	276	23	284	2.00E-56	218
	Gh_D13G0328	58.47	118	49	0	452	569	210	327	9.00E-41	166
	Gh_A11G1716	49.11	224	112	2	51	274	2	223	3.00E-56	217
	Gh_A12G0840	29.82	560	328	16	19	547	21	546	2.00E-55	214
	Gh_D13G0232	44.65	327	161	7	207	527	12	324	3.00E-55	214
	Gh_D12G0913	29.46	560	329	17	19	547	21	545	3.00E-54	210
	Gh_A05G2114	40.38	265	143	6	10	274	14	263	1.00E-52	205
	Gh_D05G2355	39.62	265	145	6	10	274	14	263	6.00E-51	199
	Gh_A05G2112	40.67	268	142	7	8	275	14	264	7.00E-51	199
	Gh_D05G2357	40.3	268	143	7	8	275	14	264	5.00E-50	196
	Gh_D04G1220	50.27	185	90	2	10	194	12	194	6.00E-49	193
	Gh_D05G2589	39.37	254	139	7	22	275	27	265	4.00E-48	190
	Gh_A05G2328	38.58	254	141	7	22	275	27	265	2.00E-47	187
**AtLAC14**	Gh_A05G2099	55.17	571	240	6	13	569	6	574	3.00E-164	576
	Gh_D05G2353	54.09	575	244	6	13	569	6	578	6.00E-163	572
	Gh_D10G2461	50.26	573	266	7	12	569	1	569	7.00E-159	558
	Gh_A10G2140	50.09	573	267	7	12	569	1	569	2.00E-158	557
	Gh_A06G1415	50.53	564	261	6	20	569	9	568	3.00E-157	553
	Gh_D06G1762	50.35	564	261	7	20	569	10	568	2.00E-156	550
	Gh_A06G1413	50.62	565	259	7	20	569	9	568	4.00E-156	549
	Gh_D08G2159	52.29	547	246	8	35	569	34	577	5.00E-153	539
	Gh_D03G1367	50.89	562	261	8	19	569	19	576	6.00E-153	538
	Gh_A03G2057	50.71	562	262	8	19	569	19	576	2.00E-152	536
	Gh_A08G2350	52.29	547	246	8	35	569	34	577	3.00E-152	536
	Gh_A09G1445	45.71	571	290	10	15	569	17	583	5.00E-146	515
	Gh_D09G1454	46.06	571	288	10	15	569	17	583	8.00E-146	514
	Gh_D13G0253	47.31	558	280	7	18	569	14	563	8.00E-145	511
	Gh_D10G0895	48.57	558	271	8	20	569	13	562	2.00E-144	510
	Gh_A13G0237	47.49	558	279	7	18	569	14	563	8.00E-144	508
	Gh_D11G3307	46.61	560	279	10	26	569	28	583	1.00E-143	508
	Gh_A10G0858	48.39	558	272	8	20	569	13	562	3.00E-143	506
	Gh_A11G2922	45.5	578	288	11	15	569	10	583	7.00E-143	505
	Gh_D01G2166	47.63	569	274	10	18	569	12	573	3.00E-141	499
	Gh_D13G0194	46.28	564	285	11	20	569	17	576	5.00E-141	499

Gh_D13G0231	46.91	550	269	10	37	569	33	576	7.00E-141	498
Gh_A05G0849	45.94	579	289	13	11	569	5	579	2.00E-140	497
Gh_A13G0216	46.55	550	271	10	37	569	33	576	2.00E-140	497
Gh_A03G0583	47.57	555	275	7	21	569	17	561	4.00E-140	496
Gh_A01G1905	47.79	565	279	10	18	569	12	573	5.00E-140	495
Gh_D05G3888	45.94	579	289	13	11	569	5	579	6.00E-140	495
Gh_D05G2354	46.54	563	286	8	16	569	6	562	1.00E-139	494
Gh_D03G0865	47.66	556	269	10	21	569	17	557	1.00E-137	488
Gh_D13G2373	44.33	573	302	9	12	569	7	577	2.00E-137	487
Gh_A05G2115	46.52	561	279	10	19	569	14	563	2.00E-137	486
Gh_A13G1977	46.49	542	279	7	37	569	38	577	1.00E-136	484
Gh_D03G1181	45.52	580	291	12	12	569	1	577	2.00E-136	484
Gh_A11G2920	45.42	568	291	10	18	569	6	570	3.00E-136	483
Gh_A05G2116	46.98	547	272	9	33	569	25	563	4.00E-136	482
Gh_D04G1224	43.38	551	297	6	31	569	20	567	4.00E-136	482
Gh_D03G1180	45.47	574	288	12	18	569	7	577	9.00E-136	481
Gh_D05G0888	46.42	558	286	7	17	569	7	556	1.00E-135	481
Gh_A05G0758	46.24	558	287	7	17	569	7	556	1.00E-135	481
Gh_A11G2936	46.35	561	283	8	19	569	4	556	2.00E-135	480
Gh_A03G2082	45.3	574	289	13	18	569	7	577	6.00E-135	478
Gh_A04G0744	43.01	551	299	6	31	569	20	567	7.00E-135	478
Gh_D11G3322	46.24	558	282	8	22	569	7	556	2.00E-134	477
Gh_A05G2631	47.57	536	246	6	41	569	3	510	2.00E-134	477
Gh_D04G1243	45.37	562	291	8	16	569	3	556	4.00E-134	476
Gh_A02G0638	44.6	565	292	9	18	569	9	565	6.00E-134	475
Gh_A03G0417	45.78	557	287	9	19	569	9	556	8.00E-134	475
Gh_D03G1128	45.78	557	287	9	19	569	9	556	1.00E-133	474
Gh_D06G1188	46.43	560	281	11	22	569	16	568	2.00E-133	474
Gh_A05G2113	46.09	562	283	10	19	569	8	560	2.00E-133	474
Gh_D11G3305	45.42	568	290	11	18	569	6	569	2.00E-132	470
Gh_A06G0997	45.63	561	284	11	22	569	16	568	1.00E-131	468
Gh_A05G2117	45	560	290	9	19	569	10	560	1.00E-131	468
Gh_D01G2209	45.7	547	282	7	30	569	20	558	1.00E-131	467
Gh_D05G2356	45.28	561	288	10	19	569	8	559	3.00E-131	466
Gh_A01G1948	45.7	547	282	7	30	569	20	558	8.00E-131	465
Gh_A04G0743	43.62	564	300	8	22	569	12	573	2.00E-130	464
Gh_A05G2622	45.34	558	291	8	18	569	6	555	3.00E-130	463
Gh_D05G2912	45.16	558	292	8	18	569	6	555	4.00E-130	462
Gh_D02G0685	44.17	566	293	11	19	569	9	566	1.00E-129	461
Gh_D10G2466	43.8	589	286	9	15	569	5	582	7.00E-129	458
Gh_D13G2524	42.7	548	297	7	33	569	23	564	3.00E-126	450
Gh_D04G1221	43.14	547	293	8	22	552	12	556	5.00E-126	449
Gh_D04G1223	42.53	562	306	7	22	569	23	581	2.00E-125	447
Gh_A13G2215	42.6	547	297	7	34	569	24	564	4.00E-125	446
Gh_A05G2100	45.49	521	269	8	16	527	6	520	1.00E-121	434
Gh_D11G1042	43.44	541	292	6	38	569	39	574	1.00E-119	427
Gh_A03G2084	43.67	529	264	11	18	527	7	520	5.00E-116	416
Gh_D12G1138	41.68	571	296	7	31	569	30	595	1.00E-114	411
Gh_A13G0179	41.47	545	258	11	37	569	33	528	3.00E-114	410
Gh_A12G1019	40.85	585	309	7	17	569	16	595	8.00E-114	408
Gh_D11G1874	41.08	538	284	9	41	555	13	540	2.00E-113	407
Gh_A05G0853	40.33	553	260	8	25	569	21	511	3.00E-111	400
Gh_Sca005020G01	36.08	571	297	11	15	569	11	529	1.00E-97	355
Gh_A13G2102	41.59	517	231	10	18	526	12	465	2.00E-93	341
Gh_D03G1177	40.23	527	240	14	18	521	7	481	5.00E-92	336
Gh_A13G2103	46.27	415	196	8	174	569	90	496	1.00E-91	334
Gh_D06G2354	48.09	366	173	5	218	569	11	373	2.00E-89	328
Gh_D05G2921	50.67	298	145	2	42	338	4	300	7.00E-83	306
Gh_D06G1759	44.88	361	173	3	20	375	10	349	3.00E-82	303
Gh_D13G2551	47.26	292	152	2	11	301	6	296	2.00E-78	291
Gh_D13G2551	53.46	159	72	1	413	569	298	456	6.00E-48	189
Gh_A13G0218	44.78	335	177	6	37	365	33	365	5.00E-77	286
Gh_A13G0218	53.17	126	55	3	405	527	310	434	1.00E-31	135

	Gh_A11G0894	42.44	344	194	3	38	380	33	373	1.00E-74	278
	Gh_D13G0232	44.04	327	152	10	218	527	12	324	7.00E-64	243
	Gh_A11G1716	44.62	260	142	2	63	322	2	259	5.00E-57	220
	Gh_A05G2112	41.33	300	155	5	22	321	14	292	2.00E-56	218
	Gh_D05G2355	40.2	296	156	5	22	317	14	288	4.00E-56	216
	Gh_D05G2357	42.45	278	146	4	22	299	14	277	9.00E-56	216
	Gh_A05G2114	39.13	299	158	6	19	317	14	288	2.00E-55	214
	Gh_A05G2328	39.44	284	151	5	34	317	27	289	8.00E-53	206
	Gh_D05G2589	40.49	284	148	6	34	317	27	289	8.00E-53	206
	Gh_A12G0840	31.96	560	331	18	31	559	21	561	2.00E-52	204
	Gh_D12G0913	32.26	558	331	18	31	559	21	560	7.00E-51	199
	Gh_D08G2100	29.21	606	329	25	16	569	9	566	7.00E-51	199
	Gh_A08G1751	29.97	584	314	25	16	547	9	549	8.00E-51	199
	Gh_D13G0328	46.57	204	104	2	31	234	27	225	7.00E-50	196
	Gh_D13G0328	55.93	118	52	0	452	569	210	327	2.00E-37	155
	Gh_A05G2101	37.63	295	154	5	22	315	14	279	3.00E-49	194
**AtLAC15**	Gh_A05G2099	58.46	532	209	9	24	548	33	559	4.00E-170	595
	Gh_D05G2353	57.84	536	210	9	24	548	33	563	8.00E-169	591
	Gh_A06G1415	47.28	533	267	9	22	546	25	551	2.00E-137	486
	Gh_D10G2461	47.29	535	265	10	22	546	25	552	7.00E-137	485
	Gh_D06G1762	47.09	533	268	9	22	546	25	551	1.00E-136	484
	Gh_A10G2140	47.48	535	264	10	22	546	25	552	2.00E-136	483
	Gh_A06G1413	46.72	533	270	9	22	546	25	551	2.00E-135	480
	Gh_D03G1367	47.17	530	270	9	22	546	35	559	6.00E-134	475
	Gh_A03G2057	47.17	530	270	9	22	546	35	559	1.00E-133	474
	Gh_D08G2159	46.7	531	272	9	22	546	35	560	3.00E-132	469
	Gh_A08G2350	47.27	531	269	10	22	546	35	560	5.00E-132	469
	Gh_A09G1445	45.14	545	281	11	20	553	36	573	1.00E-131	468
	Gh_D04G1224	46.4	541	271	9	23	553	26	557	2.00E-131	467
	Gh_D09G1454	45.14	545	281	11	20	553	36	573	3.00E-131	466
	Gh_A04G0744	46.58	541	270	9	23	553	26	557	9.00E-130	461
	Gh_D05G2354	47.17	530	263	9	22	546	28	545	2.00E-129	460
	Gh_A05G2117	45.49	532	271	9	22	546	24	543	7.00E-129	458
	Gh_D11G3307	44.3	544	287	9	20	553	36	573	2.00E-128	457
	Gh_A11G2922	44.3	544	287	9	20	553	36	573	6.00E-128	455
	Gh_A05G2115	45.94	542	269	12	21	553	27	553	1.00E-126	451
	Gh_D01G2166	46.21	541	273	11	23	553	31	563	8.00E-126	448
	Gh_A02G0638	46.64	536	275	8	22	553	27	555	9.00E-126	448
	Gh_A13G1977	44.44	540	287	9	20	553	35	567	1.00E-125	447
	Gh_D05G3888	44.95	545	284	8	20	553	30	569	2.00E-125	447
	Gh_A13G0237	45.88	534	271	10	24	553	34	553	9.00E-125	445
	Gh_A01G1905	46.21	541	273	11	23	553	31	563	1.00E-124	444
	Gh_D13G0194	44.49	544	285	9	20	553	30	566	2.00E-124	444
	Gh_D13G2373	44.26	540	288	9	20	553	35	567	3.00E-124	443
	Gh_A05G2113	45.69	534	269	10	22	546	22	543	3.00E-124	443
	Gh_D13G0253	45.88	534	271	10	24	553	34	553	6.00E-124	442
	Gh_D03G1181	45.59	544	278	12	20	553	32	567	6.00E-124	442
	Gh_A05G0849	44.77	545	285	8	20	553	30	569	7.00E-124	442
	Gh_D05G2356	45.4	533	271	10	22	546	22	542	1.00E-123	441
	Gh_A05G2631	45.42	524	247	8	28	546	4	493	1.00E-123	441
	Gh_D04G1221	44.16	539	275	10	24	546	28	556	2.00E-123	441
	Gh_D13G0231	44.65	542	287	8	20	553	30	566	2.00E-123	440
	Gh_A05G2116	45.62	537	266	11	21	547	27	547	2.00E-123	440
	Gh_D03G1180	45.42	546	276	13	20	553	32	567	4.00E-123	439
	Gh_A13G0216	44.46	542	288	8	20	553	30	566	5.00E-123	439
	Gh_A03G2082	45.24	546	277	13	20	553	32	567	6.00E-123	439
	Gh_D13G2524	44.36	532	275	10	24	546	28	547	1.00E-122	438
	Gh_D06G1188	45.52	536	274	12	19	546	25	550	2.00E-122	437
	Gh_D10G0895	45.51	534	273	10	24	553	33	552	3.00E-122	436
	Gh_A13G2215	44.36	532	275	10	24	546	28	547	7.00E-122	435
	Gh_A04G0743	43.49	538	280	10	24	546	28	556	1.00E-121	434
	Gh_A06G0997	45.44	537	273	13	19	546	25	550	2.00E-121	434
	Gh_D02G0685	45.54	538	281	8	21	553	26	556	3.00E-121	433

	Gh_A10G0858	45.32	534	274	10	24	553	33	552	3.00E-121	433
	Gh_D04G1243	43.78	539	286	10	19	553	21	546	3.00E-120	430
	Gh_A11G2920	44.18	541	286	10	23	553	26	560	1.00E-119	427
	Gh_A03G0583	44.86	535	273	12	24	553	34	551	3.00E-118	423
	Gh_D04G1223	41.46	533	295	9	24	546	39	564	9.00E-118	421
	Gh_D11G3305	43.46	543	286	12	23	553	26	559	7.00E-117	419
	Gh_D11G3322	43.41	539	288	10	19	553	21	546	1.00E-116	418
	Gh_A11G2936	43.23	539	289	10	19	553	21	546	4.00E-116	416
	Gh_D03G0865	44.49	535	271	13	24	553	34	547	1.00E-115	414
	Gh_A05G0758	43.41	539	286	11	19	553	23	546	1.00E-115	414
	Gh_D03G1128	43.23	539	286	11	19	553	24	546	5.00E-115	412
	Gh_A05G2100	45.74	505	257	9	22	521	28	520	7.00E-115	412
	Gh_D05G0888	43.23	539	287	11	19	553	23	546	3.00E-114	410
	Gh_D01G2209	44.32	537	282	10	21	553	25	548	5.00E-114	409
	Gh_A03G0417	43.04	539	287	11	19	553	24	546	6.00E-114	409
	Gh_A05G2622	43.74	535	282	10	23	553	26	545	1.00E-113	407
	Gh_A01G1948	44.32	537	282	10	21	553	25	548	2.00E-113	407
	Gh_D05G2912	43.55	535	283	10	23	553	26	545	2.00E-113	407
	Gh_D11G1042	43.88	531	280	8	24	547	39	558	2.00E-112	404
	Gh_D10G2466	42.86	567	277	12	19	553	21	572	9.00E-112	401
	Gh_A13G0179	40.41	542	262	8	20	553	30	518	1.00E-111	401
	Gh_D12G1138	42.43	568	277	11	19	547	23	579	9.00E-111	398
	Gh_A12G1019	42.6	554	277	10	24	547	37	579	6.00E-110	395
	Gh_D11G1874	41.44	543	287	10	27	551	13	542	5.00E-106	382
	Gh_A03G2084	42.69	513	259	14	20	521	32	520	2.00E-99	361
	Gh_A05G0853	39.33	539	254	10	20	553	31	501	9.00E-97	352
	Gh_D05G2921	44.8	375	180	5	28	397	4	356	3.00E-87	320
	Gh_Sca005020G01	37.16	549	271	13	20	553	31	520	4.00E-86	317
	Gh_A13G2103	44.28	411	198	11	160	553	90	486	5.00E-80	296
	Gh_D06G2354	45.33	353	179	9	202	546	10	356	2.00E-78	291
	Gh_D03G1177	39.39	495	230	15	34	515	44	481	7.00E-78	289
	Gh_A13G2102	38.45	502	236	12	23	520	33	465	2.00E-77	288
	Gh_D06G1759	40.8	348	183	6	22	366	25	352	2.00E-72	271
	Gh_A13G0218	47.16	282	148	1	20	300	30	311	3.00E-72	270
	Gh_A13G0218	44.62	130	66	5	393	521	310	434	8.00E-14	76.6
	Gh_A11G0894	41.64	341	193	3	24	363	33	368	5.00E-70	263
	Gh_D13G2551	47.92	265	137	1	23	287	33	296	6.00E-67	253
	Gh_D13G2551	49.67	153	73	4	401	553	298	446	7.00E-30	129
	Gh_D13G0232	42.38	328	167	9	202	521	11	324	2.00E-55	214
	Gh_A11G1716	43.36	256	144	1	49	304	2	256	3.00E-55	214
	Gh_A05G2328	40	285	151	6	22	306	29	293	3.00E-52	204
	Gh_D05G2589	41.38	261	139	4	22	282	29	275	7.00E-52	202
	Gh_D12G0913	31.72	558	322	19	24	550	28	557	2.00E-51	201
	Gh_A12G0840	31.66	559	324	20	23	550	27	558	4.00E-51	200
	Gh_A05G2112	38.68	287	156	5	22	308	28	294	4.00E-51	200
	Gh_A05G2114	39.46	261	144	4	22	282	28	274	4.00E-51	200
	Gh_D05G2355	40	260	142	4	23	282	29	274	4.00E-51	200
	Gh_D05G2357	38.33	287	157	5	22	308	28	294	7.00E-51	199
	Gh_D08G2100	29.82	550	323	19	23	541	32	549	1.00E-50	198
	Gh_A08G1751	29.82	550	323	19	23	541	32	549	2.00E-50	198
	Gh_A05G2101	36.49	285	146	5	23	307	29	278	1.00E-46	185
**AtLAC16**	Gh_A03G0417	73.58	530	125	5	1	523	35	556	0	785
	Gh_D05G0888	71.89	530	135	5	1	523	34	556	0	785
	Gh_D03G1128	73.21	530	127	5	1	523	35	556	0	781
	Gh_A05G0758	71.51	530	137	5	1	523	34	556	0	780
	Gh_D04G1243	70.92	533	137	6	1	523	32	556	0	768
	Gh_D11G3322	68.8	532	150	5	1	523	32	556	0	751
	Gh_A11G2936	69.36	532	147	5	1	523	32	556	0	750
	Gh_A05G2622	63.58	530	179	4	1	523	33	555	0	708
	Gh_D05G2912	63.58	530	179	4	1	523	33	555	0	706
	Gh_A01G1948	67.67	532	156	5	1	523	34	558	0	705
	Gh_D01G2209	67.11	532	159	5	1	523	34	558	0	704
	Gh_D10G2466	60.57	558	178	5	1	523	32	582	0	692

Gh_A13G0237	60.38	530	197	4	1	523	40	563	0	676
Gh_D13G0253	60.38	530	197	4	1	523	40	563	0	675
Gh_A03G0583	60.42	528	198	5	1	523	40	561	0	671
Gh_D10G0895	59.81	530	200	4	1	523	39	562	0	670
Gh_A10G0858	59.62	530	201	4	1	523	39	562	0	667
Gh_D03G0865	59.47	528	199	6	1	523	40	557	0	653
Gh_D11G3307	57.49	541	209	8	1	523	46	583	3.00E-180	629
Gh_D09G1454	57.86	541	207	8	1	523	46	583	9.00E-180	627
Gh_A11G2922	57.12	541	211	8	1	523	46	583	3.00E-179	625
Gh_A09G1445	57.12	541	211	8	1	523	46	583	2.00E-178	623
Gh_D13G0194	57.33	539	212	7	1	523	40	576	1.00E-177	620
Gh_A13G0216	56.22	539	218	7	1	523	40	576	7.00E-177	618
Gh_A05G0849	55.54	542	220	7	1	523	40	579	3.00E-176	615
Gh_D13G0231	56.4	539	217	7	1	523	40	576	4.00E-176	615
Gh_D05G3888	55.72	542	219	7	1	523	40	579	3.00E-175	612
Gh_D01G2166	54.1	536	229	6	3	523	40	573	2.00E-170	596
Gh_A11G2920	53.16	538	233	5	3	523	35	570	1.00E-168	590
Gh_A13G1977	53.62	539	228	8	1	523	45	577	2.00E-167	586
Gh_D13G2373	53.43	539	229	8	1	523	45	577	3.00E-167	586
Gh_A01G1905	53.17	536	234	5	3	523	40	573	7.00E-167	584
Gh_D11G3305	51.67	538	240	6	3	523	35	569	5.00E-164	575
Gh_D03G1180	52.88	539	231	8	3	523	44	577	1.00E-163	573
Gh_D03G1181	52.13	539	239	7	1	523	42	577	3.00E-163	572
Gh_A03G2082	51.94	541	237	8	1	523	42	577	2.00E-162	570
Gh_D06G1762	49.44	540	250	7	2	523	34	568	1.00E-154	544
Gh_A06G1415	49.26	540	251	7	2	523	34	568	3.00E-154	543
Gh_D10G2461	49.72	541	248	7	2	523	34	569	6.00E-152	535
Gh_A10G2140	49.72	541	248	7	2	523	34	569	2.00E-151	533
Gh_A06G1413	48.89	540	253	7	2	523	34	568	2.00E-151	533
Gh_A13G0179	52.13	539	192	8	1	523	40	528	4.00E-151	532
Gh_D04G1224	50.09	535	247	5	5	523	37	567	4.00E-149	525
Gh_D03G1367	49.91	535	245	7	6	523	48	576	7.00E-149	525
Gh_A04G0744	50.09	535	247	5	5	523	37	567	3.00E-148	523
Gh_A08G2350	49.63	536	246	8	6	523	48	577	9.00E-148	521
Gh_A03G2057	49.53	535	247	7	6	523	48	576	9.00E-148	521
Gh_D08G2159	49.44	536	247	8	6	523	48	577	3.00E-147	519
Gh_A05G0853	48.78	531	204	9	1	523	41	511	3.00E-143	506
Gh_A05G2631	48.4	531	238	7	2	523	7	510	3.00E-141	499
Gh_D11G1042	46.74	537	257	7	5	523	49	574	6.00E-139	491
Gh_A13G2215	49.25	532	250	7	6	523	39	564	8.00E-138	488
Gh_A03G2084	50.1	499	211	10	1	481	42	520	7.00E-137	485
Gh_D13G2524	48.5	532	254	7	6	523	39	564	1.00E-135	481
Gh_A05G2099	48.78	533	258	5	5	523	43	574	1.00E-134	478
Gh_A04G0743	47.87	541	255	8	5	523	38	573	6.00E-134	475
Gh_D05G2353	47.86	537	261	5	5	523	43	578	3.00E-133	473
Gh_D12G1138	44.36	559	259	8	6	523	48	595	4.00E-133	473
Gh_A12G1019	44.01	559	261	8	6	523	48	595	2.00E-132	470
Gh_D04G1223	47.75	534	261	6	6	523	50	581	8.00E-132	468
Gh_A13G2102	49.58	480	188	4	1	480	40	465	1.00E-130	464
Gh_A13G2103	55.83	412	163	4	125	523	91	496	2.00E-129	461
Gh_A13G2103	58.82	51	21	0	1	51	40	90	3.00E-12	71.2
Gh_Sca005020G01	45.69	534	231	11	1	521	41	528	4.00E-128	456
Gh_D04G1221	46.18	524	255	8	5	506	38	556	7.00E-127	452
Gh_D05G2354	42.78	533	287	8	2	523	37	562	6.00E-126	449
Gh_D11G1874	45.27	528	265	6	5	512	20	543	7.00E-126	448
Gh_D05G2356	42.06	535	291	8	2	523	31	559	8.00E-125	445
Gh_A05G2116	42.32	534	289	9	2	523	37	563	4.00E-124	442
Gh_A05G2115	42.32	534	289	9	2	523	37	563	6.00E-124	442
Gh_A05G2117	42.64	530	286	7	6	523	37	560	8.00E-124	441
Gh_A05G2113	41.6	536	293	8	2	523	31	560	2.00E-122	437
Gh_D06G1188	42.8	535	288	9	2	523	39	568	2.00E-120	430
Gh_A06G0997	42.8	535	288	9	2	523	39	568	4.00E-120	429
Gh_A02G0638	41.17	532	299	6	3	523	37	565	6.00E-119	425

	Gh_D02G0685	40.53	533	302	7	3	523	37	566	1.00E-115	414
	Gh_D03G1177	44.38	489	201	9	1	470	40	476	2.00E-111	400
	Gh_A05G2100	41.96	491	267	8	2	481	37	520	9.00E-109	392
	Gh_D06G2354	48.9	362	169	6	173	523	17	373	8.00E-94	342
	Gh_D13G2551	60.08	258	95	2	1	251	40	296	1.00E-92	338
	Gh_D13G2551	65	160	55	1	364	523	298	456	5.00E-60	229
	Gh_A13G0218	55.56	279	114	3	1	269	40	318	3.00E-88	323
	Gh_A13G0218	58.14	129	49	3	355	481	309	434	7.00E-38	156
	Gh_A11G0894	47.62	336	161	3	5	333	43	370	2.00E-83	307
	Gh_D05G2921	41.6	375	178	5	2	360	7	356	4.00E-80	296
	Gh_D13G0232	49.07	324	143	8	168	481	13	324	5.00E-79	293
	Gh_D06G1759	43.07	339	161	5	2	331	34	349	3.00E-72	270
	Gh_A11G1716	50.39	254	118	2	20	266	2	254	7.00E-67	252
	Gh_D13G0328	59.54	173	63	1	1	166	40	212	3.00E-61	234
	Gh_D13G0328	67.67	133	43	0	391	523	195	327	1.00E-50	198
	Gh_A12G0840	31.7	530	302	16	8	501	41	546	2.00E-59	228
	Gh_D12G0913	31.63	528	304	16	8	501	41	545	4.00E-59	227
	Gh_A02G1272	31.77	532	299	18	8	501	54	559	2.00E-58	224
	Gh_D03G0412	31.58	532	300	18	8	501	54	559	2.00E-57	221
	Gh_D08G2100	32.33	532	300	20	8	504	46	552	2.00E-56	218
	Gh_A08G1751	32.33	529	298	20	8	501	46	549	2.00E-56	218
	Gh_A06G1412	56.35	181	77	2	344	523	1	180	6.00E-56	216
	Gh_D05G2589	44.44	243	129	3	2	244	38	274	1.00E-52	205
	Gh_D05G2355	42.5	240	132	2	5	244	40	273	2.00E-51	201
	Gh_A05G2328	43.62	243	131	3	2	244	38	274	5.00E-51	199
**AtLAC17**	Gh_A05G0849	82.11	559	95	3	18	576	25	578	0	926
	Gh_D05G3888	81.75	559	97	3	18	576	25	578	0	920
	Gh_D13G0194	81.04	559	98	4	18	576	25	575	0	918
	Gh_D13G0231	79.79	559	105	4	18	576	25	575	0	917
	Gh_A13G0216	79.07	559	109	3	18	576	25	575	0	914
	Gh_D03G1181	73.74	556	135	3	22	576	31	576	0	838
	Gh_D03G1180	73.38	556	137	3	22	576	31	576	0	835
	Gh_A03G2082	73.38	556	137	3	22	576	31	576	0	834
	Gh_A13G0179	72.09	559	100	4	18	576	25	527	0	831
	Gh_D13G2373	72.3	556	140	5	22	576	34	576	0	816
	Gh_A11G2920	70.38	557	158	3	20	576	20	569	0	813
	Gh_A13G1977	72.3	556	140	5	22	576	34	576	0	810
	Gh_D01G2166	71.27	557	151	2	20	576	25	572	0	807
	Gh_A01G1905	70.68	556	154	2	20	575	25	571	0	800
	Gh_D09G1454	70.09	555	159	3	22	576	35	582	0	797
	Gh_A09G1445	69.55	555	162	3	22	576	35	582	0	794
	Gh_D11G3307	70.27	555	158	3	22	576	35	582	0	793
	Gh_D11G3305	68.94	557	165	4	20	576	20	568	0	793
	Gh_A11G2922	69.73	555	161	3	22	576	35	582	0	788
	Gh_A03G2084	71.26	515	122	6	22	535	31	520	0	722
	Gh_A05G0853	62.43	559	136	6	17	575	25	509	0	665
	Gh_Sca005020G01	62.21	561	153	7	17	575	25	528	0	651
	Gh_D04G1243	58.51	552	209	5	25	576	24	555	0	635
	Gh_D11G3322	57.97	552	212	5	25	576	24	555	0	634
	Gh_A11G2936	58.15	552	211	5	25	576	24	555	0	633
	Gh_D10G0895	57.6	559	212	7	19	575	25	560	0	632
	Gh_A10G0858	57.6	559	212	7	19	575	25	560	2.00E-180	630
	Gh_A05G0758	57.58	554	213	7	23	576	24	555	2.00E-178	623
	Gh_A13G0237	56.81	558	218	7	19	575	26	561	5.00E-178	622
	Gh_D05G0888	57.4	554	214	7	23	576	24	555	5.00E-178	622
	Gh_D13G0253	56.63	558	219	7	19	575	26	561	3.00E-177	619
	Gh_D03G1128	56.68	554	217	6	23	576	25	555	3.00E-176	616
	Gh_A03G0417	56.14	554	220	6	23	576	25	555	9.00E-175	611
	Gh_D10G2466	54.14	580	216	7	25	576	24	581	3.00E-174	609
	Gh_D03G1177	63.93	499	117	6	30	524	37	476	1.00E-173	607
	Gh_A03G0583	56.51	561	215	9	18	575	25	559	3.00E-173	606
	Gh_A01G1948	57.79	552	213	5	25	576	26	557	9.00E-173	604
	Gh_D01G2209	57.43	552	215	5	25	576	26	557	1.00E-172	603

Gh_D03G0865	56.15	561	213	10	18	575	25	555	2.00E-169	593
Gh_A05G2622	53.62	552	234	5	25	576	25	554	6.00E-169	592
Gh_D05G2912	53.26	552	236	5	25	576	25	554	1.00E-167	587
Gh_A06G1415	50.63	559	254	12	23	575	24	566	1.00E-150	531
Gh_D08G2159	51.44	554	253	8	26	576	36	576	6.00E-150	528
Gh_D06G1762	50.45	559	255	12	23	575	24	566	7.00E-150	528
Gh_A08G2350	51.26	554	254	8	26	576	36	576	1.00E-149	527
Gh_A13G0218	79.6	299	61	0	18	316	25	323	6.00E-149	525
Gh_A13G0218	82.54	126	22	0	410	535	309	434	4.00E-48	190
Gh_A03G2057	50.99	557	240	9	31	576	41	575	1.00E-148	524
Gh_D10G2461	50.63	559	258	10	22	575	22	567	3.00E-148	523
Gh_D03G1367	50.45	557	243	9	31	576	41	575	2.00E-147	520
Gh_A10G2140	50.63	559	258	10	22	575	22	567	2.00E-147	520
Gh_A06G1413	49.91	559	258	12	23	575	24	566	2.00E-147	520
Gh_D04G1224	48.84	559	258	9	26	575	26	565	4.00E-144	509
Gh_A04G0744	48.66	559	259	9	26	575	26	565	7.00E-144	508
Gh_A05G2099	48.22	562	276	7	18	576	24	573	3.00E-136	483
Gh_D11G1042	45.74	564	279	9	19	576	31	573	2.00E-134	477
Gh_D05G2353	47.35	566	279	8	18	576	24	577	2.00E-134	477
Gh_D11G1874	46.35	548	264	11	30	565	13	542	9.00E-133	471
Gh_A13G2215	46.31	555	274	7	27	576	28	563	1.00E-132	471
Gh_D12G1138	44.29	587	277	10	19	576	29	594	2.00E-132	471
Gh_A12G1019	43.88	588	280	10	18	576	28	594	7.00E-131	465
Gh_D13G2524	46.28	551	270	8	33	576	32	563	8.00E-131	465
Gh_D13G0232	75.45	330	64	5	206	535	12	324	3.00E-130	463
Gh_A04G0743	47.32	560	270	7	27	576	28	572	4.00E-130	462
Gh_A05G2631	46.64	551	244	11	30	575	3	508	1.00E-129	461
Gh_D04G1223	46.03	554	283	5	27	576	39	580	4.00E-128	456
Gh_D04G1221	46.25	547	269	7	24	560	25	556	3.00E-127	453
Gh_D05G2354	41.77	553	300	8	26	576	29	561	2.00E-119	427
Gh_A05G2115	40.36	555	306	8	26	576	29	562	3.00E-114	410
Gh_A13G2102	46.03	517	201	8	19	534	26	465	4.00E-113	406
Gh_A13G2103	53.4	427	163	7	163	575	90	494	4.00E-113	406
Gh_A13G2103	46.15	65	35	0	19	83	26	90	1.00E-12	72.8
Gh_A05G2113	39.68	557	312	9	25	576	22	559	9.00E-113	405
Gh_D05G2356	39.21	556	315	8	25	576	22	558	2.00E-112	404
Gh_A05G2116	40	555	308	8	26	576	29	562	2.00E-110	397
Gh_D06G1188	38.66	551	319	8	24	572	29	562	4.00E-108	389
Gh_A02G0638	38.85	556	316	9	25	575	27	563	7.00E-108	389
Gh_A06G0997	39.02	551	317	9	24	572	29	562	9.00E-108	388
Gh_A05G2117	38.2	555	319	8	26	576	25	559	1.00E-106	385
Gh_A05G2100	41.8	512	276	8	26	535	29	520	9.00E-106	382
Gh_D02G0685	38.6	557	317	10	25	575	27	564	4.00E-104	376
Gh_D13G2551	56.46	271	116	1	19	289	26	294	8.00E-97	352
Gh_D13G2551	65.16	155	53	1	422	575	300	454	3.00E-46	184
Gh_D05G2921	46.79	389	167	8	31	415	4	356	3.00E-94	343
Gh_A11G0894	44.23	364	185	5	19	381	25	371	3.00E-88	323
Gh_D06G2354	49.2	376	170	11	206	575	11	371	1.00E-82	305
Gh_D06G1759	44.13	358	166	8	23	378	24	349	4.00E-79	293
Gh_A11G1716	52.9	259	118	2	52	309	2	257	2.00E-74	277
Gh_D13G0328	54.92	193	87	0	19	211	26	218	3.00E-66	251
Gh_D13G0328	64.54	141	48	1	437	575	185	325	6.00E-40	163
Gh_D12G0913	32.97	555	305	21	25	551	26	541	2.00E-55	215
Gh_A12G0840	32.49	551	314	19	25	551	26	542	6.00E-55	213
Gh_Sca030590G01	76.32	152	35	1	385	535	1	152	7.00E-55	213
Gh_D08G2100	29.77	561	316	20	24	551	30	545	2.00E-51	201
Gh_A08G1751	29.59	561	317	20	24	551	30	545	8.00E-51	199
Gh_A02G1272	29.98	557	320	19	25	551	39	555	2.00E-50	198
Gh_D03G0412	30.24	549	329	18	25	551	39	555	2.00E-50	197
Gh_D04G1220	50.6	166	82	0	27	192	28	193	5.00E-47	186
Gh_A05G2114	36.62	284	153	7	26	306	29	288	1.00E-45	182
Gh_D05G2355	35.94	281	159	7	26	306	29	288	3.00E-45	181

Supplementary Table S2: Chromosomal distribution of all the laccase family genes identified from cotton.

S. No.	Gene Name	Gene ID	Chromosome Number	Start position	Stop position	Molecular wt. (Kda)	pI	Sub-cellular localization
G. arboreum (A-genome)								
1	LAC02_1	Cotton_A_20043	Chr8	81279651	81282485	Table 1	Table 1	Table 1
2	LAC02_2	Cotton_A_30643	Chr7	112198734	112200887			
3	LAC02_3	Cotton_A_30645	Chr7	112238136	112240289			
4	LAC02_4	Cotton_A_30646	Chr7	112301808	112303961			
5	LAC03_1	Cotton_A_04178	Chr7	78051563	78053707			
6	LAC03_2	Cotton_A_24290	Chr13	89192357	89194554			
7	LAC04_1	Cotton_A_00335	Chr2	69974070	69977957			
8	LAC04_2	Cotton_A_05572	Chr4	129106164	129108201			
9	LAC04_3	Cotton_A_06597	Chr7	27437711	27439798			
10	LAC04_4	Cotton_A_12917	Chr9	66032424	66035279			
11	LAC04_5	Cotton_A_13553	Chr10	96546298	96548750			
12	LAC04_6	Cotton_A_20282	Chr7	54244095	54246807			
13	LAC04_7	Cotton_A_29171	Chr10	47067405	47070913			
14	LAC04_8	Cotton_A_32213	Chr10	87092454	87095892			
15	LAC05_1	Cotton_A_13817	Chr8	71027959	71034475			
16	LAC05_2	Cotton_A_13818	Chr8	71068414	71070598			
17	LAC05_3	Cotton_A_13820	Chr8	71134606	71136600			
18	LAC05_4	Cotton_A_15837	Chr13	66318063	66320195			
19	LAC06_1	Cotton_A_14417	Chr4	7772175	7774365			
20	LAC06_2	Cotton_A_25874	Chr6	45584114	45586109			
21	LAC07_1	Cotton_A_22687	Chr12	86399040	86401261			
22	LAC07_2	Cotton_A_30033	Chr12	111604680	111608069			
23	LAC07_3	Cotton_A_30034	Chr12	111490687	111493668			
24	LAC07_4	Cotton_A_30035	Chr12	111394263	111396884			
25	LAC07_5	Cotton_A_35771	Chr13	539188	543296			
26	LAC11_1	Cotton_A_00882	Chr13	73503949	73506086			
27	LAC11_2	Cotton_A_17036	Chr9	56144460	56146555			
28	LAC11_3	Cotton_A_19723	Chr13	63352239	63354366			
29	LAC11_4	Cotton_A_26217	Chr7	32225217	32227325			

30	LAC11_5	Cotton_A_26221	Chr7	32087805	32092421			
31	LAC12	Cotton_A_31477	Chr10	76670719	76672888			
32	LAC14_1	Cotton_A_04514	Chr6	105156736	105159112			
33	LAC14_2	Cotton_A_04517	Chr6	105080230	105082613			
34	LAC14_3	Cotton_A_04519	Chr6	105038752	105049813			
35	LAC14_4	Cotton_A_04522	Chr6	105012062	105014213			
36	LAC14_5	Cotton_A_04526	Chr6	104947203	104949548			
37	LAC14_6	Cotton_A_10403	Chr9	69308986	69311473			
38	LAC14_7	Cotton_A_37880	Chr12	73129546	73131714			
39	LAC15	Cotton_A_04513	Chr6	105171153	105174123			
40	LAC17_1	Cotton_A_00902	Chr13	73653118	73655337			
41	LAC17_2	Cotton_A_00905	Chr13	73732088	73734303			
42	LAC17_3	Cotton_A_00947	Chr13	74192475	74194689			
43	LAC17_4	Cotton_A_07013	Chr10	88398331	88400538			
44	LAC17_5	Cotton_A_12054	Chr11	77398558	77400798			
***G. raimondii* (D-genome)**								
1	LAC02_1	Gr013G263200	Chr13	57687706	57691942	63.67	9.35	Secretory
2	LAC02_5	Gr003G129800	Chr3	38287051	38289204	63.736	9.21	Secretory
3	LAC02_6	Gr003G129700	Chr3	38235116	38237269	63.745	9.21	Secretory
4	LAC03_1	Gr003G150200	Chr3	41747074	41744911	64.283	8.89	Secretory
5	LAC03_2	Gr004G234200	Chr4	57205285	57203087	63.925	8.62	Secretory
6	LAC04_1	Gr002G261500	Chr2	62202782	62206452	61.288	7.28	Secretory
7	LAC04_2	Gr007G378200	Chr7	60866231	60868271	60.639	9.28	Secretory
8	LAC04_3	Gr012G111900	Chr12	25401214	25403301	60.778	9.33	Secretory
9	LAC04_4	Gr009G093800	Chr9	6847898	6845152	61.147	9.5	Secretory
10	LAC04_5	Gr011G279600	Chr11	61143453	61145929	60.987	8.68	Secretory
11	LAC04_6	Gr003G124600	Chr3	37186699	37183971	61.309	9.04	Secretory
12	LAC04_8	Gr009G321900	Chr9	31218190	31221933	61.31	8.06	Secretory
13	LAC05_3	Gr010G194600	Chr10	55406818	55409007	63.178	9.27	Secretory
14	LAC05_4	Gr011G290000	Chr11	62138112	62140340	62.76	6.94	Secretory
15	LAC05_5	Gr010G194200	Chr10	55212656	55214647	46.045	8.8	Non-secretory
16	LAC05_6	Gr010G194300	Chr10	55272915	55270710	63.188	9.17	Secretory
17	LAC06_1	Gr007G110500	Chr7	8404536	8406746	63.71	6.3	Secretory

18	LAC06_2	Gr008G126500	Chr8	36744467	36746510	63.719	6.96	Secretory
19	LAC06_3	Gr007G205400	Chr7	21186046	21188148	57.94	9.12	Mitochondrial
20	LAC07_2	Gr012G110000	Chr12	25016814	25020047	62.588	8.58	Secretory
21	LAC07_3	Gr012G109900	Chr12	24927474	24930488	63.895	8.78	Secretory
22	LAC07_4	Gr012G109700	Chr12	24841018	24843824	62.864	8.82	Secretory
23	LAC07_5	Gr002G148200	Chr2	28200096	28204171	62.282	8.02	Secretory
24	LAC09	Gr012G109500	Chr12	24671921	24674718	63.002	8.65	Secretory
25	LAC11_1	Gr013G027600	Chr13	2047626	2046122	45.179	8.68	Non-secretory
26	LAC11_2	Gr011G101300	Chr11	11406722	11404624	62.344	9.06	Secretory
27	LAC11_3	Gr013G036200	Chr13	2815434	2813291	62.183	8.86	Secretory
28	LAC11_4	Gr003G096600	Chr3	30023645	30025779	62.907	9.24	Secretory
29	LAC11_6	Gr013G036000	Chr13	2793278	2791152	50.511	7.73	Secretory
30	LAC14_1	Gr009G260600	Chr9	21486909	21484563	62.136	5.56	Secretory
31	LAC14_2	Gr009G260800	Chr9	21504167	21501798	63.11	6.16	Secretory
32	LAC14_3	Gr009G261000	Chr9	21530522	21528102	63.257	5.49	Secretory
33	LAC14_4	Gr009G261500	Chr9	21590611	21588241	61.584	4.8	Secretory
34	LAC14_5	Gr009G261300	Chr9	21566227	21563832	62.68	4.66	Secretory
35	LAC14_6	Gr005G076600	Chr5	8710389	8708127	65.935	5.85	Secretory
36	LAC14_7	Gr010G130100	Chr10	28224989	28227209	63.911	6.13	Secretory
37	LAC14_8	Gr009G261100	Chr9	21548569	21546803	63.371	6.43	Secretory
38	LAC15	Gr009G260400	Chr9	21482299	21473913	64.423	7.65	Secretory
39	LAC17_2	Gr013G025500	Chr13	1912277	1914496	63.462	8.83	Secretory
40	LAC17_3	Gr013G021400	Chr13	1510205	1507991	63.466	8.92	Secretory
41	LAC17_4	Gr009G103200	Chr9	7444118	7441908	64.061	9.08	Secretory
42	LAC17_5	Gr006G171500	Chr6	43067355	43069568	64.609	9.87	Secretory
43	LAC17_6	Gr007G376800	Chr7	60798694	60796617	64.772	9.86	Secretory
44	LAC17_7	Gr002G257100	Chr2	61893165	61889176	63.841	8.51	Secretory
45	LAC17_8	Gr007G376600	Chr7	60789897	60792410	63.469	8.97	Secretory
46	LAC17_9	Gr009G103600	Chr9	7497129	7499844	56.182	8.86	Secretory
***G. hirsutum* A-subgenome**								
1	LAC02_1	Gh_A13G1977	A13	79264620	79268910	63.722	9.36	Secretory
2	LAC02_2	Gh_A03G2084	scaffold518_A03	99719	105189	57.453	9.54	Secretory
3	LAC02_4	Gh_A03G2082	scaffold518_A03	1825	68065	63.778	9.35	Secretory

4	LAC03_1	Gh_A03G2057	scaffold511_A03	178235	180382	64.383	8.6	Secretory
5	LAC03_2	Gh_A08G2350	scaffold1930_A08	6846	9046	64.075	8.51	Secretory
6	LAC04_1	Gh_A01G1948	A01	99395294	99399154	61.372	7.65	Secretory
7	LAC04_2	Gh_A11G2936	A11	93159674	93161710	60.656	9.29	Secretory
8	LAC04_4	Gh_A05G0758	A05	7794137	7796989	61.063	9.54	Secretory
9	LAC04_6	Gh_A03G0417	A03	8607635	8610346	61.333	8.81	Secretory
10	LAC04_8	Gh_A05G2622	A05	39425421	39428800	61.035	8.32	Secretory
11	LAC05_2	Gh_A06G1413	A06	96545202	96608221	63.204	9.23	Secretory
12	LAC05_3	Gh_A06G1415	A06	96665317	96667521	63.206	9.32	Secretory
13	LAC05_4	Gh_A10G2140	A10	100194082	100196409	63	6.96	Secretory
14	LAC06_1	Gh_A11G0894	A11	9206275	9207783	41.539	8.04	Secretory
15	LAC06_2	Gh_A12G1019	A12	61687640	61689683	66.534	7.57	Secretory
16	LAC06_3	Gh_A11G1716	A11	26751630	26752781	30.954	9.45	Non-secretory
17	LAC07_1	Gh_A04G0743	A04	50627781	50743297	62.911	8.1	Secretory
18	LAC07_2	Gh_A04G0744	A04	50843009	50846398	62.656	8.57	Secretory
19	LAC07_5	Gh_A13G2215	scaffold3554_A13	11736	15813	62.21	7.67	Secretory
20	LAC11_1	Gh_A13G0237	A13	2768658	2770795	62.752	9.11	Secretory
21	LAC11_2	Gh_A10G0858	A10	18506385	18508484	62.206	8.74	Secretory
22	LAC11_3	Gh_A13G2102	scaffold3415_A13	168658	170534	52.11	9.05	Secretory
23	LAC11_4	Gh_A03G0583	A03	15372800	15374880	62.755	9.27	Secretory
24	LAC11_7	Gh_A13G2103	scaffold3415_A13	176544	182802	54.431	8.49	Secretory
25	LAC12	Gh_A05G2631	A05	40457256	40459435	56.408	9.48	Non-secretory
26	LAC14_1	Gh_A05G2100	A05	23711696	23714334	59.982	5.73	Secretory
27	LAC14_2	Gh_A05G2117	A05	24094935	24097318	63.078	6.83	Secretory
28	LAC14_3	Gh_A05G2116	A05	24066813	24069230	63.244	5.78	Secretory
29	LAC14_4	Gh_A05G2113	A05	24031174	24033544	62.895	4.98	Secretory
30	LAC14_6	Gh_A02G0638	A02	10108128	10110615	65.884	5.98	Secretory
31	LAC14_7	Gh_A06G0997	A06	47977469	47979639	63.909	6.25	Secretory
32	LAC14_8	Gh_A05G2115	A05	24060793	24063195	63.342	6.24	Secretory
33	LAC15	Gh_A05G2099	A05	23696963	23699162	64.59	8.24	Secretory
34	LAC17_1	Gh_A13G0218	A13	2604596	2606573	48.058	8.24	Secretory
35	LAC17_2	Gh_A13G0216	A13	2530591	2532809	63.401	8.48	Secretory
36	LAC17_3	Gh_A13G0179	A13	1981529	1983743	58.323	8.94	Secretory
37	LAC17_4	Gh_A05G0849	A05	8473281	8475491	63.977	9.01	Secretory

38	LAC17_5	Gh_A09G1445	A09	67731186	67733528	64.493	9.89	Secretory
39	LAC17_6	Gh_A11G2922	A11	93089381	93091461	64.63	9.89	Secretory
40	LAC17_7	Gh_A01G1905	A01	98990679	98995193	63.973	8.85	Secretory
41	LAC17_8	Gh_A11G2920	A11	93082651	93085155	63.445	9.02	Secretory
42	LAC17_9	Gh_A05G0853	A05	8523038	8525208	56.485	9.04	Secretory
***G. hirsutum* D-subgenome**								
1	*LAC02_1*	Gh_D13G2373	D13	59874172	59878480	63.689	9.39	Secretory
2	*LAC02_5*	Gh_D03G1181	D03	38563246	38565399	63.626	9.3	Secretory
3	*LAC02_6*	Gh_D03G1180	D03	38536987	38539552	63.729	9.27	Secretory
4	*LAC03_1*	Gh_D03G1367	D03	42244904	42247055	64.284	8.89	Secretory
5	*LAC03_2*	Gh_D08G2159	D08	61034429	61036627	63.991	8.72	Secretory
6	*LAC04_1*	Gh_D01G2209	D01	60950279	60958788	61.244	7.28	Secretory
7	*LAC04_2*	Gh_D11G3322	D11	66019363	66021401	60.656	9.23	Secretory
8	*LAC04_3*	Gh_D04G1243	D04	40647135	40649222	60.796	9.33	Secretory
9	*LAC04_4*	Gh_D05G0888	D05	7511957	7514764	64.094	9.01	Secretory
10	*LAC04_5*	Gh_D10G2466	scaffold4398_D10	17261	19737	64.335	8.73	Secretory
11	*LAC04_6*	Gh_D03G1128	D03	37433498	37436758	61.329	9.04	Secretory
12	*LAC04_8*	Gh_D05G2912	D05	33858990	33862531	61.312	8.06	Secretory
13	*LAC05_3*	Gh_D06G1762	D06	57379288	57381478	63.268	9.27	Secretory
14	*LAC05_4*	Gh_D10G2461	scaffold4395_D10	172369	174667	62.746	6.94	Secretory
15	*LAC05_5*	Gh_D06G2354	scaffold4155_D06	108	1310	63.072	5.31	Secretory
16	*LAC05_6*	Gh_D06G1759	D06	57337178	57338648	39.060	8.11	Secretory
17	*LAC06_1*	Gh_D11G1042	D11	9202928	9205138	63.697	6.3	Secretory
18	*LAC06_2*	Gh_D12G1138	D12	38326335	38328379	66.559	6.95	Secretory
19	*LAC06_3*	Gh_D11G1874	D11	21832473	21834814	61.948	8.57	Non-secretory
20	*LAC07_2*	Gh_D04G1224	D04	40228079	40231273	62.589	8.58	Secretory
21	*LAC07_3*	Gh_D04G1223	D04	40204685	40207701	63.919	8.88	Secretory
22	*LAC07_4*	Gh_D04G1221	D04	40131410	40134195	62.848	8.83	Secretory
23	*LAC07_5*	Gh_D13G2524	scaffold4697_D13	1380014	1384875	62.351	8.02	Secretory
24	***LAC11_1***	Gh_D13G0253	D13	2439918	2442055	62.829	9.22	Secretory
25	***LAC11_2***	Gh_D10G0895	D10	11525129	11527341	62.191	8.91	Secretory
26	***LAC11_3***	Gh_D13G2551	scaffold4716_D13	4631	6806	50.461	7.25	Secretory
27	***LAC11_4***	Gh_D03G0865	D03	30060064	30062170	62.427	9.17	Secretory

28	***LAC11_6***	Gh_D13G0328	D13	3297315	3299169	36.416	7.73	Secretory
29	*LAC12*	Gh_D05G2921	D05	34408572	34410175	39.787	9.18	Mitochondrial
30	***LAC14_1***	Gh_D05G2354	D05	23304099	23306467	63.072	5.31	Secretory
31	***LAC14_5***	Gh_D05G2356	D05	23393118	23395516	62.705	4.84	Secretory
32	***LAC14_6***	Gh_D02G0685	D02	9686886	9689379	66.057	5.85	Secretory
33	***LAC14_7***	Gh_D06G1188	D06	29636934	29639110	63.991	6.13	Secretory
34	*LAC15*	Gh_D05G2353	D05	23292980	23295193	64.781	7.06	Secretory
35	*LAC17_2*	Gh_D13G0231	D13	2268740	2270959	63.412	9	Secretory
36	*LAC17_3*	Gh_D13G0194	D13	1802345	1804559	63.376	9	Secretory
37	*LAC17_4*	Gh_D05G3888	scaffold4075_D05	34814	37024	61.117	9.46	Secretory
38	*LAC17_5*	Gh_D09G1454	D09	42133032	42135245	64.610	9.87	Secretory
39	*LAC17_6*	Gh_D11G3307	D11	65948893	65950972	64.766	9.87	Secretory
40	*LAC17_7*	Gh_D01G2166	D01	60605724	60609611	63.771	8.32	Secretory
41	*LAC17_8*	Gh_D11G3305	D11	65942124	65944712	63.404	9.01	Secretory
42	*LAC17_9*	Gh_Sca005020G01				58.289	8.6	Secretory

Supplementary Table S3A: Domain prediction analysis of all the identified Laccase family proteins from *G. arboreum* .

	Conserved Domain Database								InterProScan	
**Query**	**Hit type**	**PSSM-ID**	**From**	**To**	**E-Value**	**Bitscore**	**Accession**	**Short name**	**Accession**	**Short name**
GaLAC02_1	specific	259943	128	276	4.05E-83	256.371	cd13875	CuRO_2_LCC_plant	IPR001117	Multicopper oxidase, type 1
	superfamily	277509	128	276	4.05E-83	256.371	cl19115	Cupredoxin superfamily	IPR011707	Multicopper oxidase, type 3
	specific	259918	1	113	1.48E-71	225.216	cd13849	CuRO_1_LCC_plant	IPR011706	Multicopper oxidase, type 2
	superfamily	277509	1	113	1.48E-71	225.216	cl19115	Cupredoxin superfamily	IPR017761	Laccase
	superfamily	277509	380	493	1.47E-60	197.097	cl19115	Cupredoxin superfamily		
	multi-dom	274556	1	493	0	843.633	TIGR03389	laccase		
GaLAC02_2	specific	259964	423	560	1.58E-81	254.106	cd13897	CuRO_3_LCC_plant	IPR001117	Multicopper oxidase, type 1
	superfamily	277509	423	560	1.58E-81	254.106	cl19115	Cupredoxin superfamily	IPR011707	Multicopper oxidase, type 3
	specific	259943	167	315	1.24E-79	249.438	cd13875	CuRO_2_LCC_plant	IPR011706	Multicopper oxidase, type 2
	superfamily	277509	167	315	1.24E-79	249.438	cl19115	Cupredoxin superfamily	IPR017761	Laccase
	specific	259918	36	152	3.50E-71	226.372	cd13849	CuRO_1_LCC_plant		
	superfamily	277509	36	152	3.50E-71	226.372	cl19115	Cupredoxin superfamily		
	multi-dom	274556	31	577	0	930.688	TIGR03389	laccase		
GaLAC02_3	specific	259943	167	315	1.98E-80	251.364	cd13875	CuRO_2_LCC_plant	IPR001117	Multicopper oxidase, type 1
	superfamily	277509	167	315	1.98E-80	251.364	cl19115	Cupredoxin superfamily	IPR011707	Multicopper oxidase, type 3
	specific	259964	423	560	1.40E-79	249.099	cd13897	CuRO_3_LCC_plant	IPR011706	Multicopper oxidase, type 2
	superfamily	277509	423	560	1.40E-79	249.099	cl19115	Cupredoxin superfamily	IPR017761	Laccase
	specific	259918	36	152	7.84E-74	233.305	cd13849	CuRO_1_LCC_plant		
	superfamily	277509	36	152	7.84E-74	233.305	cl19115	Cupredoxin superfamily		
	multi-dom	274556	31	577	0	937.237	TIGR03389	laccase		
GaLAC02_4	specific	259964	423	560	3.42E-80	250.639	cd13897	CuRO_3_LCC_plant	IPR001117	Multicopper oxidase, type 1
	superfamily	277509	423	560	3.42E-80	250.639	cl19115	Cupredoxin superfamily	IPR011707	Multicopper oxidase, type 3
	specific	259943	167	315	1.75E-79	249.052	cd13875	CuRO_2_LCC_plant	IPR011706	Multicopper oxidase, type 2
	superfamily	277509	167	315	1.75E-79	249.052	cl19115	Cupredoxin superfamily	IPR017761	Laccase
	specific	259918	36	152	7.27E-72	227.913	cd13849	CuRO_1_LCC_plant		
	superfamily	277509	36	152	7.27E-72	227.913	cl19115	Cupredoxin superfamily		
	multi-dom	274556	31	577	0	938.392	TIGR03389	laccase		
GaLAC03_1	specific	259943	168	314	4.26E-89	274.09	cd13875	CuRO_2_LCC_plant	IPR001117	Multicopper oxidase, type 1
	superfamily	277509	168	314	4.26E-89	274.09	cl19115	Cupredoxin superfamily	IPR011707	Multicopper oxidase, type 3
	specific	259964	421	559	6.22E-81	252.565	cd13897	CuRO_3_LCC_plant	IPR011706	Multicopper oxidase, type 2
	superfamily	277509	421	559	6.22E-81	252.565	cl19115	Cupredoxin superfamily	IPR017761	Laccase
	specific	259918	37	153	6.69E-73	230.609	cd13849	CuRO_1_LCC_plant		
	superfamily	277509	37	153	6.69E-73	230.609	cl19115	Cupredoxin superfamily		

	multi-dom	274556	32	576	0	844.403	TIGR03389	laccase		
GaLAC03_2	specific	259943	169	314	4.45E-89	274.09	cd13875	CuRO_2_LCC_plant	IPR001117	Multicopper oxidase, type 1
	superfamily	277509	169	314	4.45E-89	274.09	cl19115	Cupredoxin superfamily	IPR011707	Multicopper oxidase, type 3
	specific	259964	421	559	2.94E-82	256.032	cd13897	CuRO_3_LCC_plant	IPR011706	Multicopper oxidase, type 2
	superfamily	277509	421	559	2.94E-82	256.032	cl19115	Cupredoxin superfamily	IPR017761	Laccase
	specific	259918	37	153	4.16E-72	228.683	cd13849	CuRO_1_LCC_plant		
	superfamily	277509	37	153	4.16E-72	228.683	cl19115	Cupredoxin superfamily		
	multi-dom	274556	34	576	0	850.567	TIGR03389	laccase		
GaLAC04_1	specific	259964	404	541	1.16E-80	251.41	cd13897	CuRO_3_LCC_plant	IPR001117	Multicopper oxidase, type 1
	superfamily	277509	404	541	1.16E-80	251.41	cl19115	Cupredoxin superfamily	IPR011707	Multicopper oxidase, type 3
	specific	259943	159	305	9.92E-75	236.341	cd13875	CuRO_2_LCC_plant	IPR011706	Multicopper oxidase, type 2
	superfamily	277509	159	305	9.92E-75	236.341	cl19115	Cupredoxin superfamily	IPR017761	Laccase
	specific	259918	28	144	7.46E-60	195.941	cd13849	CuRO_1_LCC_plant		
	superfamily	277509	28	144	7.46E-60	195.941	cl19115	Cupredoxin superfamily		
	multi-dom	274556	24	558	0	923.369	TIGR03389	laccase		
GaLAC04_2	specific	259964	402	539	5.92E-81	252.18	cd13897	CuRO_3_LCC_plant	IPR001117	Multicopper oxidase, type 1
	superfamily	277509	402	539	5.92E-81	252.18	cl19115	Cupredoxin superfamily	IPR011707	Multicopper oxidase, type 3
	specific	259943	158	303	6.20E-80	249.823	cd13875	CuRO_2_LCC_plant	IPR011706	Multicopper oxidase, type 2
	superfamily	277509	158	303	6.20E-80	249.823	cl19115	Cupredoxin superfamily	IPR017761	Laccase
	specific	259918	26	142	3.76E-69	220.209	cd13849	CuRO_1_LCC_plant		
	superfamily	277509	26	142	3.76E-69	220.209	cl19115	Cupredoxin superfamily		
	multi-dom	274556	21	556	0	978.453	TIGR03389	laccase		
GaLAC04_3	specific	259964	402	539	5.22E-83	257.188	cd13897	CuRO_3_LCC_plant	IPR001117	Multicopper oxidase, type 1
	superfamily	277509	402	539	5.22E-83	257.188	cl19115	Cupredoxin superfamily	IPR011707	Multicopper oxidase, type 3
	specific	259943	157	303	4.63E-79	247.512	cd13875	CuRO_2_LCC_plant	IPR011706	Multicopper oxidase, type 2
	superfamily	277509	157	303	4.63E-79	247.512	cl19115	Cupredoxin superfamily	IPR017761	Laccase
	specific	259918	26	142	8.90E-73	229.839	cd13849	CuRO_1_LCC_plant		
	superfamily	277509	26	142	8.90E-73	229.839	cl19115	Cupredoxin superfamily		
	multi-dom	274556	21	556	0	986.927	TIGR03389	laccase		
GaLAC04_4	specific	259943	161	305	6.30E-79	247.126	cd13875	CuRO_2_LCC_plant	IPR001117	Multicopper oxidase, type 1
	superfamily	277509	161	305	6.30E-79	247.126	cl19115	Cupredoxin superfamily	IPR011707	Multicopper oxidase, type 3
	specific	259964	402	539	1.02E-78	246.017	cd13897	CuRO_3_LCC_plant	IPR011706	Multicopper oxidase, type 2
	superfamily	277509	402	539	1.02E-78	246.017	cl19115	Cupredoxin superfamily	IPR017761	Laccase
	specific	259918	28	144	4.84E-72	227.913	cd13849	CuRO_1_LCC_plant		
	superfamily	277509	28	144	4.84E-72	227.913	cl19115	Cupredoxin superfamily		
	multi-dom	274556	23	556	0	976.142	TIGR03389	laccase		

GaLAC04_5	specific	259964	426	563	1.42E-76	241.395	cd13897	CuRO_3_LCC_plant	IPR001117	Multicopper oxidase, type 1
	superfamily	277509	426	563	1.42E-76	241.395	cl19115	Cupredoxin superfamily	IPR011707	Multicopper oxidase, type 3
	specific	259943	184	329	4.98E-76	240.193	cd13875	CuRO_2_LCC_plant	IPR011706	Multicopper oxidase, type 2
	superfamily	277509	184	329	4.98E-76	240.193	cl19115	Cupredoxin superfamily	IPR017761	Laccase
	specific	259918	55	168	4.43E-66	212.89	cd13849	CuRO_1_LCC_plant		
	superfamily	277509	55	168	4.43E-66	212.89	cl19115	Cupredoxin superfamily		
	multi-dom	274556	21	580	0	945.711	TIGR03389	laccase		
GaLAC04_6	specific	259964	402	539	8.98E-79	246.402	cd13897	CuRO_3_LCC_plant	IPR001117	Multicopper oxidase, type 1
	superfamily	277509	402	539	8.98E-79	246.402	cl19115	Cupredoxin superfamily	IPR011707	Multicopper oxidase, type 3
	specific	259943	162	305	4.75E-78	244.815	cd13875	CuRO_2_LCC_plant	IPR011706	Multicopper oxidase, type 2
	superfamily	277509	162	305	4.75E-78	244.815	cl19115	Cupredoxin superfamily	IPR017761	Laccase
	specific	259918	29	145	1.58E-70	224.061	cd13849	CuRO_1_LCC_plant		
	superfamily	277509	29	145	1.58E-70	224.061	cl19115	Cupredoxin superfamily		
	multi-dom	274556	24	556	0	961.889	TIGR03389	laccase		
GaLAC04_7	superfamily	277509	333	455	9.33E-62	199.963	cl19115	Cupredoxin superfamily	IPR001117	Multicopper oxidase, type 1
	superfamily	277509	106	251	1.08E-61	200.322	cl19115	Cupredoxin superfamily	IPR011707	Multicopper oxidase, type 3
	superfamily	277509	1	92	6.93E-53	176.444	cl19115	Cupredoxin superfamily	IPR011706	Multicopper oxidase, type 2
	multi-dom	274556	1	490	2.53E-63	216.913	TIGR03389	laccase		
GaLAC04_8	specific	259964	401	538	4.10E-78	244.476	cd13897	CuRO_3_LCC_plant	IPR001117	Multicopper oxidase, type 1
	superfamily	277509	401	538	4.10E-78	244.476	cl19115	Cupredoxin superfamily	IPR011707	Multicopper oxidase, type 3
	specific	259918	27	143	1.75E-68	218.668	cd13849	CuRO_1_LCC_plant	IPR011706	Multicopper oxidase, type 2
	superfamily	277509	27	143	1.75E-68	218.668	cl19115	Cupredoxin superfamily	IPR017761	Laccase
	specific	259943	159	304	3.36E-68	219.007	cd13875	CuRO_2_LCC_plant		
	superfamily	277509	159	304	3.36E-68	219.007	cl19115	Cupredoxin superfamily		
	multi-dom	274556	23	555	0	922.599	TIGR03389	laccase		
GaLAC05_1	specific	259964	390	528	1.63E-86	266.047	cd13897	CuRO_3_LCC_plant	IPR001117	Multicopper oxidase, type 1
	superfamily	277509	390	528	1.63E-86	266.047	cl19115	Cupredoxin superfamily	IPR011707	Multicopper oxidase, type 3
	specific	259918	27	143	1.37E-72	229.068	cd13849	CuRO_1_LCC_plant	IPR011706	Multicopper oxidase, type 2
	superfamily	277509	27	143	1.37E-72	229.068	cl19115	Cupredoxin superfamily	IPR017761	Laccase
	specific	259943	164	282	1.29E-71	227.867	cd13875	CuRO_2_LCC_plant		
	superfamily	277509	164	282	1.29E-71	227.867	cl19115	Cupredoxin superfamily		
	multi-dom	274556	22	545	0	867.515	TIGR03389	laccase		
GaLAC05_2	specific	259943	159	305	1.84E-88	272.164	cd13875	CuRO_2_LCC_plant	IPR001117	Multicopper oxidase, type 1
	superfamily	277509	159	305	1.84E-88	272.164	cl19115	Cupredoxin superfamily	IPR011707	Multicopper oxidase, type 3
	specific	259964	413	551	8.93E-86	264.892	cd13897	CuRO_3_LCC_plant	IPR011706	Multicopper oxidase, type 2
	superfamily	277509	413	551	8.93E-86	264.892	cl19115	Cupredoxin superfamily	IPR017761	Laccase

	specific	259918	27	143	5.76E-72	227.913	cd13849	CuRO_1_LCC_plant		
	superfamily	277509	27	143	5.76E-72	227.913	cl19115	Cupredoxin superfamily		
	multi-dom	274556	22	568	0	912.199	TIGR03389	laccase		
GaLAC05_3	specific	259943	129	275	3.79E-90	275.631	cd13875	CuRO_2_LCC_plant	IPR001117	Multicopper oxidase, type 1
	superfamily	277509	129	275	3.79E-90	275.631	cl19115	Cupredoxin superfamily	IPR011707	Multicopper oxidase, type 3
	specific	259964	383	521	1.37E-86	266.047	cd13897	CuRO_3_LCC_plant	IPR011706	Multicopper oxidase, type 2
	superfamily	277509	383	521	1.37E-86	266.047	cl19115	Cupredoxin superfamily	IPR017761	Laccase
	specific	259918	1	113	3.98E-70	222.52	cd13849	CuRO_1_LCC_plant		
	superfamily	277509	1	113	3.98E-70	222.52	cl19115	Cupredoxin superfamily		
	multi-dom	274556	1	538	0	904.495	TIGR03389	laccase		
GaLAC05_4	specific	259943	130	275	2.26E-87	268.698	cd13875	CuRO_2_LCC_plant	IPR001117	Multicopper oxidase, type 1
	superfamily	277509	130	275	2.26E-87	268.698	cl19115	Cupredoxin superfamily	IPR011707	Multicopper oxidase, type 3
	specific	259964	384	522	6.41E-85	261.81	cd13897	CuRO_3_LCC_plant	IPR011706	Multicopper oxidase, type 2
	superfamily	277509	384	522	6.41E-85	261.81	cl19115	Cupredoxin superfamily	IPR017761	Laccase
	specific	259918	1	113	2.30E-69	220.594	cd13849	CuRO_1_LCC_plant		
	superfamily	277509	1	113	2.30E-69	220.594	cl19115	Cupredoxin superfamily		
	multi-dom	274556	1	539	0	887.546	TIGR03389	laccase		
GaLAC06_1	specific	259943	164	311	2.13E-77	243.274	cd13875	CuRO_2_LCC_plant	IPR001117	Multicopper oxidase, type 1
	superfamily	277509	164	311	2.13E-77	243.274	cl19115	Cupredoxin superfamily	IPR011707	Multicopper oxidase, type 3
	specific	259964	413	551	8.73E-76	238.698	cd13897	CuRO_3_LCC_plant	IPR011706	Multicopper oxidase, type 2
	superfamily	277509	413	551	8.73E-76	238.698	cl19115	Cupredoxin superfamily	IPR017761	Laccase
	specific	259918	33	149	3.63E-66	212.89	cd13849	CuRO_1_LCC_plant		
	superfamily	277509	33	149	3.63E-66	212.89	cl19115	Cupredoxin superfamily		
	multi-dom	274556	28	568	0	743.866	TIGR03389	laccase		
GaLAC06_2	specific	259943	152	299	4.11E-76	239.808	cd13875	CuRO_2_LCC_plant	IPR001117	Multicopper oxidase, type 1
	superfamily	277509	152	299	4.11E-76	239.808	cl19115	Cupredoxin superfamily	IPR011707	Multicopper oxidase, type 3
	specific	259964	401	539	1.10E-73	232.92	cd13897	CuRO_3_LCC_plant	IPR011706	Multicopper oxidase, type 2
	superfamily	277509	401	539	1.10E-73	232.92	cl19115	Cupredoxin superfamily	IPR017761	Laccase
	specific	259918	21	137	2.47E-67	215.586	cd13849	CuRO_1_LCC_plant		
	superfamily	277509	21	137	2.47E-67	215.586	cl19115	Cupredoxin superfamily		
	multi-dom	274556	16	556	0	740.4	TIGR03389	laccase		
GaLAC07_1	specific	259943	135	286	2.28E-81	253.29	cd13875	CuRO_2_LCC_plant	IPR001117	Multicopper oxidase, type 1
	superfamily	277509	135	286	2.28E-81	253.29	cl19115	Cupredoxin superfamily	IPR011707	Multicopper oxidase, type 3
	specific	259964	391	532	1.27E-72	230.224	cd13897	CuRO_3_LCC_plant	IPR011706	Multicopper oxidase, type 2
	superfamily	277509	391	532	1.27E-72	230.224	cl19115	Cupredoxin superfamily	IPR017761	Laccase
	specific	259918	6	120	2.58E-62	202.104	cd13849	CuRO_1_LCC_plant		

	superfamily	277509	6	120	2.58E-62	202.104	cl19115	Cupredoxin superfamily		
	multi-dom	274556	8	532	0	702.265	TIGR03389	laccase		
GaLAC07_2	specific	259943	158	305	9.78E-86	265.231	cd13875	CuRO_2_LCC_plant	IPR001117	Multicopper oxidase, type 1
	superfamily	277509	158	305	9.78E-86	265.231	cl19115	Cupredoxin superfamily	IPR011707	Multicopper oxidase, type 3
	specific	259918	27	143	1.13E-70	224.446	cd13849	CuRO_1_LCC_plant	IPR011706	Multicopper oxidase, type 2
	superfamily	277509	27	143	1.13E-70	224.446	cl19115	Cupredoxin superfamily	IPR017761	Laccase
	superfamily	277509	408	550	1.61E-70	225.216	cl19115	Cupredoxin superfamily		
	multi-dom	274556	22	567	0	774.682	TIGR03389	laccase		
GaLAC07_3	specific	259964	412	553	1.57E-75	238.313	cd13897	CuRO_3_LCC_plant	IPR001117	Multicopper oxidase, type 1
	superfamily	277509	412	553	1.57E-75	238.313	cl19115	Cupredoxin superfamily	IPR011707	Multicopper oxidase, type 3
	specific	259943	159	306	1.61E-66	214.77	cd13875	CuRO_2_LCC_plant	IPR011706	Multicopper oxidase, type 2
	superfamily	277509	159	306	1.61E-66	214.77	cl19115	Cupredoxin superfamily	IPR017761	Laccase
	specific	259918	28	144	4.33E-63	204.801	cd13849	CuRO_1_LCC_plant		
	superfamily	277509	28	144	4.33E-63	204.801	cl19115	Cupredoxin superfamily		
	multi-dom	274556	23	570	0	692.635	TIGR03389	laccase		
GaLAC07_4	specific	259943	128	279	4.97E-81	252.134	cd13875	CuRO_2_LCC_plant	IPR001117	Multicopper oxidase, type 1
	superfamily	277509	128	279	4.97E-81	252.134	cl19115	Cupredoxin superfamily	IPR011707	Multicopper oxidase, type 3
	specific	259964	384	525	1.21E-76	240.239	cd13897	CuRO_3_LCC_plant	IPR011706	Multicopper oxidase, type 2
	superfamily	277509	384	525	1.21E-76	240.239	cl19115	Cupredoxin superfamily	IPR017761	Laccase
	specific	259918	1	113	1.89E-62	202.49	cd13849	CuRO_1_LCC_plant		
	superfamily	277509	1	113	1.89E-62	202.49	cl19115	Cupredoxin superfamily		
	multi-dom	274556	1	525	0	707.272	TIGR03389	laccase		
GaLAC07_5	specific	259943	159	306	2.40E-80	250.978	cd13875	CuRO_2_LCC_plant	IPR001117	Multicopper oxidase, type 1
	superfamily	277509	159	306	2.40E-80	250.978	cl19115	Cupredoxin superfamily	IPR011707	Multicopper oxidase, type 3
	specific	259918	28	144	6.18E-68	217.512	cd13849	CuRO_1_LCC_plant	IPR011706	Multicopper oxidase, type 2
	superfamily	277509	28	144	6.18E-68	217.512	cl19115	Cupredoxin superfamily	IPR017761	Laccase
	superfamily	277509	406	547	1.54E-69	222.52	cl19115	Cupredoxin superfamily		
	multi-dom	274556	24	564	0	716.902	TIGR03389	laccase		
GaLAC11_1	specific	259943	167	311	4.22E-87	268.698	cd13875	CuRO_2_LCC_plant	IPR001117	Multicopper oxidase, type 1
	superfamily	277509	167	311	4.22E-87	268.698	cl19115	Cupredoxin superfamily	IPR011707	Multicopper oxidase, type 3
	specific	259964	408	546	6.39E-84	259.884	cd13897	CuRO_3_LCC_plant	IPR011706	Multicopper oxidase, type 2
	superfamily	277509	408	546	6.39E-84	259.884	cl19115	Cupredoxin superfamily	IPR017761	Laccase
	specific	259918	34	150	6.95E-70	222.52	cd13849	CuRO_1_LCC_plant		
	superfamily	277509	34	150	6.95E-70	222.52	cl19115	Cupredoxin superfamily		
	multi-dom	274556	29	563	0	975.756	TIGR03389	laccase		
GaLAC11_2	specific	259943	192	336	1.60E-85	265.231	cd13875	CuRO_2_LCC_plant	IPR001117	Multicopper oxidase, type 1

	superfamily	277509	192	336	1.60E-85	265.231	cl19115	Cupredoxin superfamily	IPR011707	Multicopper oxidase, type 3
	specific	259964	433	571	1.79E-83	259.499	cd13897	CuRO_3_LCC_plant	IPR011706	Multicopper oxidase, type 2
	superfamily	277509	433	571	1.79E-83	259.499	cl19115	Cupredoxin superfamily	IPR017761	Laccase
	specific	259918	62	175	7.58E-71	225.601	cd13849	CuRO_1_LCC_plant		
	superfamily	277509	62	175	7.58E-71	225.601	cl19115	Cupredoxin superfamily		
	multi-dom	274556	63	588	0	980.379	TIGR03389	laccase		
GaLAC11_3	specific	259943	167	311	5.61E-88	270.624	cd13875	CuRO_2_LCC_plant	IPR001117	Multicopper oxidase, type 1
	superfamily	277509	167	311	5.61E-88	270.624	cl19115	Cupredoxin superfamily	IPR011707	Multicopper oxidase, type 3
	specific	259964	408	546	8.41E-86	264.892	cd13897	CuRO_3_LCC_plant	IPR011706	Multicopper oxidase, type 2
	superfamily	277509	408	546	8.41E-86	264.892	cl19115	Cupredoxin superfamily	IPR017761	Laccase
	specific	259918	34	150	4.06E-70	222.905	cd13849	CuRO_1_LCC_plant		
	superfamily	277509	34	150	4.06E-70	222.905	cl19115	Cupredoxin superfamily		
	multi-dom	274556	29	563	0	981.149	TIGR03389	laccase		
GaLAC11_4	specific	259943	139	283	7.92E-88	269.468	cd13875	CuRO_2_LCC_plant	IPR001117	Multicopper oxidase, type 1
	superfamily	277509	139	283	7.92E-88	269.468	cl19115	Cupredoxin superfamily	IPR011707	Multicopper oxidase, type 3
	specific	259964	380	518	3.41E-84	259.884	cd13897	CuRO_3_LCC_plant	IPR011706	Multicopper oxidase, type 2
	superfamily	277509	380	518	3.41E-84	259.884	cl19115	Cupredoxin superfamily	IPR017761	Laccase
	superfamily	277509	36	122	1.12E-43	151.643	cl19115	Cupredoxin superfamily		
	multi-dom	274556	31	535	0	902.569	TIGR03389	laccase		
GaLAC11_5	specific	259943	100	244	2.21E-86	263.305	cd13875	CuRO_2_LCC_plant	IPR001117	Multicopper oxidase, type 1
	superfamily	277509	100	244	2.21E-86	263.305	cl19115	Cupredoxin superfamily	IPR011707	Multicopper oxidase, type 3
	superfamily	277509	341	432	8.61E-45	154.34	cl19115	Cupredoxin superfamily	IPR011706	Multicopper oxidase, type 2
	superfamily	277509	1	83	2.94E-39	138.546	cl19115	Cupredoxin superfamily		
	multi-dom	274556	1	449	0	756.963	TIGR03389	laccase		
GaLAC12	specific	259943	129	275	1.42E-86	266.386	cd13875	CuRO_2_LCC_plant	IPR001117	Multicopper oxidase, type 1
	superfamily	277509	129	275	1.42E-86	266.386	cl19115	Cupredoxin superfamily	IPR011707	Multicopper oxidase, type 3
	specific	259964	379	517	1.40E-84	260.655	cd13897	CuRO_3_LCC_plant	IPR011706	Multicopper oxidase, type 2
	superfamily	277509	379	517	1.40E-84	260.655	cl19115	Cupredoxin superfamily	IPR017761	Laccase
	specific	259918	1	113	8.53E-71	224.061	cd13849	CuRO_1_LCC_plant		
	superfamily	277509	1	113	8.53E-71	224.061	cl19115	Cupredoxin superfamily		
	multi-dom	274556	1	534	0	897.561	TIGR03389	laccase		
GaLAC14_1	specific	259964	408	546	2.46E-81	253.336	cd13897	CuRO_3_LCC_plant	IPR001117	Multicopper oxidase, type 1
	superfamily	277509	408	546	2.46E-81	253.336	cl19115	Cupredoxin superfamily	IPR011707	Multicopper oxidase, type 3
	specific	259943	161	307	8.00E-73	231.333	cd13875	CuRO_2_LCC_plant	IPR011706	Multicopper oxidase, type 2
	superfamily	277509	161	307	8.00E-73	231.333	cl19115	Cupredoxin superfamily	IPR017761	Laccase
	specific	259918	30	146	3.12E-62	202.49	cd13849	CuRO_1_LCC_plant		

	superfamily	277509	30	146	3.12E-62	202.49	cl19115	Cupredoxin superfamily		
	multi-dom	274556	25	563	0	689.168	TIGR03389	laccase		
GaLAC14_2	specific	259964	405	543	6.16E-75	236.387	cd13897	CuRO_3_LCC_plant	IPR001117	Multicopper oxidase, type 1
	superfamily	277509	405	543	6.16E-75	236.387	cl19115	Cupredoxin superfamily	IPR011707	Multicopper oxidase, type 3
	specific	259943	157	303	8.87E-71	225.941	cd13875	CuRO_2_LCC_plant	IPR011706	Multicopper oxidase, type 2
	superfamily	277509	157	303	8.87E-71	225.941	cl19115	Cupredoxin superfamily	IPR017761	Laccase
	specific	259918	26	142	2.59E-61	199.793	cd13849	CuRO_1_LCC_plant		
	superfamily	277509	26	142	2.59E-61	199.793	cl19115	Cupredoxin superfamily		
	multi-dom	274556	21	560	0	678.382	TIGR03389	laccase		
GaLAC14_3	specific	259943	284	430	7.14E-72	229.407	cd13875	CuRO_2_LCC_plant	IPR001117	Multicopper oxidase, type 1
	superfamily	277509	284	430	7.14E-72	229.407	cl19115	Cupredoxin superfamily	IPR011707	Multicopper oxidase, type 3
	specific	259918	157	269	5.50E-61	199.408	cd13849	CuRO_1_LCC_plant		
	superfamily	277509	157	269	5.50E-61	199.408	cl19115	Cupredoxin superfamily		
	superfamily	277509	531	565	1.42E-08	53.0321	cl19115	Cupredoxin superfamily		
GaLAC14_4	specific	259943	155	301	4.86E-69	220.163	cd13875	CuRO_2_LCC_plant	IPR001117	Multicopper oxidase, type 1
	superfamily	277509	155	301	4.86E-69	220.163	cl19115	Cupredoxin superfamily	IPR011707	Multicopper oxidase, type 3
	specific	259918	24	140	1.19E-64	207.497	cd13849	CuRO_1_LCC_plant	IPR011706	Multicopper oxidase, type 2
	superfamily	277509	24	140	1.19E-64	207.497	cl19115	Cupredoxin superfamily	IPR017761	Laccase
	superfamily	277509	403	519	1.52E-55	184.385	cl19115	Cupredoxin superfamily		
	multi-dom	274556	19	519	0	617.136	TIGR03389	laccase		
GaLAC14_5	specific	259943	155	301	7.17E-68	217.851	cd13875	CuRO_2_LCC_plant	IPR001117	Multicopper oxidase, type 1
	superfamily	277509	155	301	7.17E-68	217.851	cl19115	Cupredoxin superfamily	IPR011707	Multicopper oxidase, type 3
	specific	259918	24	140	4.76E-65	209.423	cd13849	CuRO_1_LCC_plant	IPR011706	Multicopper oxidase, type 2
	superfamily	277509	24	140	4.76E-65	209.423	cl19115	Cupredoxin superfamily	IPR017761	Laccase
	superfamily	277509	403	531	3.90E-65	210.579	cl19115	Cupredoxin superfamily		
	multi-dom	274556	19	548	0	651.033	TIGR03389	laccase		
GaLAC14_6	specific	259918	29	145	5.46E-68	217.897	cd13849	CuRO_1_LCC_plant	IPR001117	Multicopper oxidase, type 1
	superfamily	277509	29	145	5.46E-68	217.897	cl19115	Cupredoxin superfamily	IPR011707	Multicopper oxidase, type 3
	specific	259943	160	309	9.98E-65	210.533	cd13875	CuRO_2_LCC_plant	IPR011706	Multicopper oxidase, type 2
	superfamily	277509	160	309	9.98E-65	210.533	cl19115	Cupredoxin superfamily	IPR017761	Laccase
	superfamily	277509	409	548	2.06E-62	204.03	cl19115	Cupredoxin superfamily		
	multi-dom	274556	24	565	0	659.508	TIGR03389	laccase		
GaLAC14_7	specific	259964	412	550	1.72E-78	246.017	cd13897	CuRO_3_LCC_plant	IPR001117	Multicopper oxidase, type 1
	superfamily	277509	412	550	1.72E-78	246.017	cl19115	Cupredoxin superfamily	IPR011707	Multicopper oxidase, type 3
	specific	259918	32	148	1.90E-64	208.268	cd13849	CuRO_1_LCC_plant	IPR011706	Multicopper oxidase, type 2
	superfamily	277509	32	148	1.90E-64	208.268	cl19115	Cupredoxin superfamily	IPR017761	Laccase

	specific	259943	163	308	1.88E-63	206.681	cd13875	CuRO_2_LCC_plant		
	superfamily	277509	163	308	1.88E-63	206.681	cl19115	Cupredoxin superfamily		
	multi-dom	274556	27	568	0	654.885	TIGR03389	laccase		
GaLAC15	specific	259943	116	263	1.99E-82	255.216	cd13875	CuRO_2_LCC_plant	IPR001117	Multicopper oxidase, type 1
	superfamily	277509	116	263	1.99E-82	255.216	cl19115	Cupredoxin superfamily	IPR011707	Multicopper oxidase, type 3
	specific	259964	371	510	2.00E-78	244.476	cd13897	CuRO_3_LCC_plant	IPR011706	Multicopper oxidase, type 2
	superfamily	277509	371	510	2.00E-78	244.476	cl19115	Cupredoxin superfamily	IPR017761	Laccase
	superfamily	277509	12	101	4.77E-47	160.888	cl19115	Cupredoxin superfamily		
	multi-dom	274556	12	527	0	757.348	TIGR03389	laccase		
GaLAC17_1	specific	259964	422	559	1.32E-82	256.803	cd13897	CuRO_3_LCC_plant	IPR001117	Multicopper oxidase, type 1
	superfamily	277509	422	559	1.32E-82	256.803	cl19115	Cupredoxin superfamily	IPR011707	Multicopper oxidase, type 3
	specific	259943	165	313	1.06E-81	254.83	cd13875	CuRO_2_LCC_plant	IPR011706	Multicopper oxidase, type 2
	superfamily	277509	165	313	1.06E-81	254.83	cl19115	Cupredoxin superfamily	IPR017761	Laccase
	specific	259918	34	150	4.12E-70	223.29	cd13849	CuRO_1_LCC_plant		
	superfamily	277509	34	150	4.12E-70	223.29	cl19115	Cupredoxin superfamily		
	multi-dom	274556	29	576	0	963.43	TIGR03389	laccase		
GaLAC17_2	specific	259943	165	313	1.27E-82	257.142	cd13875	CuRO_2_LCC_plant	IPR001117	Multicopper oxidase, type 1
	superfamily	277509	165	313	1.27E-82	257.142	cl19115	Cupredoxin superfamily	IPR011707	Multicopper oxidase, type 3
	specific	259964	422	559	1.43E-82	256.803	cd13897	CuRO_3_LCC_plant	IPR011706	Multicopper oxidase, type 2
	superfamily	277509	422	559	1.43E-82	256.803	cl19115	Cupredoxin superfamily	IPR017761	Laccase
	specific	259918	34	150	6.62E-70	222.905	cd13849	CuRO_1_LCC_plant		
	superfamily	277509	34	150	6.62E-70	222.905	cl19115	Cupredoxin superfamily		
	multi-dom	274556	29	576	0	957.267	TIGR03389	laccase		
GaLAC17_3	specific	259964	422	559	3.66E-81	252.951	cd13897	CuRO_3_LCC_plant	IPR001117	Multicopper oxidase, type 1
	superfamily	277509	422	559	3.66E-81	252.951	cl19115	Cupredoxin superfamily	IPR011707	Multicopper oxidase, type 3
	specific	259943	165	313	1.47E-78	246.741	cd13875	CuRO_2_LCC_plant	IPR011706	Multicopper oxidase, type 2
	superfamily	277509	165	313	1.47E-78	246.741	cl19115	Cupredoxin superfamily	IPR017761	Laccase
	specific	259918	34	150	2.29E-69	221.364	cd13849	CuRO_1_LCC_plant		
	superfamily	277509	34	150	2.29E-69	221.364	cl19115	Cupredoxin superfamily		
	multi-dom	274556	29	576	0	947.252	TIGR03389	laccase		
GaLAC17_4	specific	259964	425	562	4.56E-83	258.343	cd13897	CuRO_3_LCC_plant	IPR001117	Multicopper oxidase, type 1
	superfamily	277509	425	562	4.56E-83	258.343	cl19115	Cupredoxin superfamily	IPR011707	Multicopper oxidase, type 3
	specific	259943	165	313	2.27E-82	256.756	cd13875	CuRO_2_LCC_plant	IPR011706	Multicopper oxidase, type 2
	superfamily	277509	165	313	2.27E-82	256.756	cl19115	Cupredoxin superfamily	IPR017761	Laccase
	specific	259918	34	150	2.10E-70	224.061	cd13849	CuRO_1_LCC_plant		
	superfamily	277509	34	150	2.10E-70	224.061	cl19115	Cupredoxin superfamily		

	multi-dom	274556	29	579	0	959.963	TIGR03389	laccase		
GaLAC17_5	specific	259964	460	597	7.11E-81	253.336	cd13897	CuRO_3_LCC_plant	IPR001117	Multicopper oxidase, type 1
	superfamily	277509	460	597	7.11E-81	253.336	cl19115	Cupredoxin superfamily	IPR011707	Multicopper oxidase, type 3
	specific	259943	171	350	7.56E-78	245.586	cd13875	CuRO_2_LCC_plant	IPR011706	Multicopper oxidase, type 2
	superfamily	277509	171	350	7.56E-78	245.586	cl19115	Cupredoxin superfamily	IPR017761	Laccase
	specific	259918	40	156	6.83E-72	229.068	cd13849	CuRO_1_LCC_plant		
	superfamily	277509	40	156	6.83E-72	229.068	cl19115	Cupredoxin superfamily		
	multi-dom	274556	35	614	0	938.007	TIGR03389	laccase		

Supplementary Table S3B: Domain prediction analysis of all the identified Laccase family proteins from *G. raimondii* .

	Conserved Domain Database							InterProScan	
**Query**	**Hit type**	**From**	**To**	**E-Value**	**Bitscore**	**Accession**	**Short name**	**Accession**	**Short name**
LAC02_1	specific	170	318	9.83E-81	252.519	cd13875	CuRO_2_LCC_plant	IPR001117	Multicopper oxidase, type 1
	superfamily	170	318	9.83E-81	252.519	cl19115	Cupredoxin superfamily	IPR011707	Multicopper oxidase, type 3
	specific	422	560	3.20E-80	250.639	cd13897	CuRO_3_LCC_plant	IPR011706	Multicopper oxidase, type 2
	superfamily	422	560	3.20E-80	250.639	cl19115	Cupredoxin superfamily	IPR017761	Laccase
	specific	39	155	8.81E-73	230.224	cd13849	CuRO_1_LCC_plant		
	superfamily	39	155	8.81E-73	230.224	cl19115	Cupredoxin superfamily		
	multi-dom	36	577	0	946.481	TIGR03389	laccase		
LAC02_5	specific	423	560	5.18E-82	255.262	cd13897	CuRO_3_LCC_plant	IPR001117	Multicopper oxidase, type 1
	superfamily	423	560	5.18E-82	255.262	cl19115	Cupredoxin superfamily	IPR011707	Multicopper oxidase, type 3
	specific	167	315	6.29E-81	252.904	cd13875	CuRO_2_LCC_plant	IPR011706	Multicopper oxidase, type 2
	superfamily	167	315	6.29E-81	252.904	cl19115	Cupredoxin superfamily	IPR017761	Laccase
	specific	36	152	2.51E-73	231.765	cd13849	CuRO_1_LCC_plant		
	superfamily	36	152	2.51E-73	231.765	cl19115	Cupredoxin superfamily		
	multi-dom	31	577	0	944.941	TIGR03389	laccase		
LAC02_6	specific	423	560	1.34E-81	254.106	cd13897	CuRO_3_LCC_plant	IPR001117	Multicopper oxidase, type 1
	superfamily	423	560	1.34E-81	254.106	cl19115	Cupredoxin superfamily	IPR011707	Multicopper oxidase, type 3
	specific	167	315	4.49E-80	250.593	cd13875	CuRO_2_LCC_plant	IPR011706	Multicopper oxidase, type 2
	superfamily	167	315	4.49E-80	250.593	cl19115	Cupredoxin superfamily	IPR017761	Laccase
	specific	36	152	1.62E-71	227.142	cd13849	CuRO_1_LCC_plant		
	superfamily	36	152	1.62E-71	227.142	cl19115	Cupredoxin superfamily		
	multi-dom	31	577	0	933.385	TIGR03389	laccase		
LAC03_1	specific	168	314	2.95E-90	277.172	cd13875	CuRO_2_LCC_plant	IPR001117	Multicopper oxidase, type 1
	superfamily	168	314	2.95E-90	277.172	cl19115	Cupredoxin superfamily	IPR011707	Multicopper oxidase, type 3
	specific	421	559	5.84E-81	252.565	cd13897	CuRO_3_LCC_plant	IPR011706	Multicopper oxidase, type 2
	superfamily	421	559	5.84E-81	252.565	cl19115	Cupredoxin superfamily	IPR017761	Laccase
	specific	37	153	4.92E-72	228.298	cd13849	CuRO_1_LCC_plant		
	superfamily	37	153	4.92E-72	228.298	cl19115	Cupredoxin superfamily		
	multi-dom	32	576	0	840.937	TIGR03389	laccase		
LAC03_2	specific	169	314	1.38E-87	270.238	cd13875	CuRO_2_LCC_plant	IPR001117	Multicopper oxidase, type 1
	superfamily	169	314	1.38E-87	270.238	cl19115	Cupredoxin superfamily	IPR011707	Multicopper oxidase, type 3
	specific	421	559	1.70E-83	259.114	cd13897	CuRO_3_LCC_plant	IPR011706	Multicopper oxidase, type 2
	superfamily	421	559	1.70E-83	259.114	cl19115	Cupredoxin superfamily	IPR017761	Laccase

	specific	37	153	7.35E-72	227.913	cd13849	CuRO_1_LCC_plant		
	superfamily	37	153	7.35E-72	227.913	cl19115	Cupredoxin superfamily		
	multi-dom	34	576	0	855.574	TIGR03389	laccase		
LAC04_1	specific	404	541	6.21E-81	252.18	cd13897	CuRO_3_LCC_plant	IPR001117	Multicopper oxidase, type 1
	superfamily	404	541	6.21E-81	252.18	cl19115	Cupredoxin superfamily	IPR011707	Multicopper oxidase, type 3
	specific	159	305	7.50E-74	234.03	cd13875	CuRO_2_LCC_plant	IPR011706	Multicopper oxidase, type 2
	superfamily	159	305	7.50E-74	234.03	cl19115	Cupredoxin superfamily	IPR017761	Laccase
	specific	28	144	4.87E-60	196.326	cd13849	CuRO_1_LCC_plant		
	superfamily	28	144	4.87E-60	196.326	cl19115	Cupredoxin superfamily		
	multi-dom	24	558	0	924.525	TIGR03389	laccase		
LAC04_2	specific	402	539	1.58E-80	251.025	cd13897	CuRO_3_LCC_plant	IPR001117	Multicopper oxidase, type 1
	superfamily	402	539	1.58E-80	251.025	cl19115	Cupredoxin superfamily	IPR011707	Multicopper oxidase, type 3
	specific	158	303	7.92E-80	249.438	cd13875	CuRO_2_LCC_plant	IPR011706	Multicopper oxidase, type 2
	superfamily	158	303	7.92E-80	249.438	cl19115	Cupredoxin superfamily	IPR017761	Laccase
	specific	26	142	1.50E-69	221.364	cd13849	CuRO_1_LCC_plant		
	superfamily	26	142	1.50E-69	221.364	cl19115	Cupredoxin superfamily		
	multi-dom	21	556	0	980.764	TIGR03389	laccase		
LAC04_3	specific	402	539	4.08E-82	254.877	cd13897	CuRO_3_LCC_plant	IPR001117	Multicopper oxidase, type 1
	superfamily	402	539	4.08E-82	254.877	cl19115	Cupredoxin superfamily	IPR011707	Multicopper oxidase, type 3
	specific	157	303	5.85E-79	247.126	cd13875	CuRO_2_LCC_plant	IPR011706	Multicopper oxidase, type 2
	superfamily	157	303	5.85E-79	247.126	cl19115	Cupredoxin superfamily	IPR017761	Laccase
	specific	26	142	7.79E-72	227.527	cd13849	CuRO_1_LCC_plant		
	superfamily	26	142	7.79E-72	227.527	cl19115	Cupredoxin superfamily		
	multi-dom	21	556	0	986.927	TIGR03389	laccase		
LAC04_4	specific	402	539	2.15E-80	250.639	cd13897	CuRO_3_LCC_plant	IPR001117	Multicopper oxidase, type 1
	superfamily	402	539	2.15E-80	250.639	cl19115	Cupredoxin superfamily	IPR011707	Multicopper oxidase, type 3
	specific	161	305	4.99E-79	247.512	cd13875	CuRO_2_LCC_plant	IPR011706	Multicopper oxidase, type 2
	superfamily	161	305	4.99E-79	247.512	cl19115	Cupredoxin superfamily	IPR017761	Laccase
	specific	28	144	2.10E-72	229.068	cd13849	CuRO_1_LCC_plant		
	superfamily	28	144	2.10E-72	229.068	cl19115	Cupredoxin superfamily		
	multi-dom	23	556	0	982.305	TIGR03389	laccase		
LAC04_5	specific	400	537	2.37E-76	239.854	cd13897	CuRO_3_LCC_plant	IPR001117	Multicopper oxidase, type 1
	superfamily	400	537	2.37E-76	239.854	cl19115	Cupredoxin superfamily	IPR011707	Multicopper oxidase, type 3
	specific	158	303	7.07E-72	228.637	cd13875	CuRO_2_LCC_plant	IPR011706	Multicopper oxidase, type 2
	superfamily	158	303	7.07E-72	228.637	cl19115	Cupredoxin superfamily	IPR017761	Laccase

	specific	26	142	1.73E-67	215.971	cd13849	CuRO_1_LCC_plant		
	superfamily	26	142	1.73E-67	215.971	cl19115	Cupredoxin superfamily		
	multi-dom	21	554	0	952.259	TIGR03389	laccase		
LAC04_6	specific	162	305	5.17E-80	249.823	cd13875	CuRO_2_LCC_plant	IPR001117	Multicopper oxidase, type 1
	superfamily	162	305	5.17E-80	249.823	cl19115	Cupredoxin superfamily	IPR011707	Multicopper oxidase, type 3
	specific	402	539	7.66E-79	246.402	cd13897	CuRO_3_LCC_plant	IPR011706	Multicopper oxidase, type 2
	superfamily	402	539	7.66E-79	246.402	cl19115	Cupredoxin superfamily	IPR017761	Laccase
	specific	29	145	1.44E-70	224.061	cd13849	CuRO_1_LCC_plant		
	superfamily	29	145	1.44E-70	224.061	cl19115	Cupredoxin superfamily		
	multi-dom	24	556	0	967.282	TIGR03389	laccase		
LAC04_8	specific	401	538	5.15E-79	246.787	cd13897	CuRO_3_LCC_plant	IPR001117	Multicopper oxidase, type 1
	superfamily	401	538	5.15E-79	246.787	cl19115	Cupredoxin superfamily	IPR011707	Multicopper oxidase, type 3
	specific	159	304	9.00E-69	220.548	cd13875	CuRO_2_LCC_plant	IPR011706	Multicopper oxidase, type 2
	superfamily	159	304	9.00E-69	220.548	cl19115	Cupredoxin superfamily	IPR017761	Laccase
	specific	27	143	1.20E-68	219.053	cd13849	CuRO_1_LCC_plant		
	superfamily	27	143	1.20E-68	219.053	cl19115	Cupredoxin superfamily		
	multi-dom	23	555	0	924.525	TIGR03389	laccase		
LAC05_3	specific	159	305	1.99E-88	272.164	cd13875	CuRO_2_LCC_plant	IPR001117	Multicopper oxidase, type 1
	superfamily	159	305	1.99E-88	272.164	cl19115	Cupredoxin superfamily	IPR011707	Multicopper oxidase, type 3
	specific	413	551	1.69E-86	266.818	cd13897	CuRO_3_LCC_plant	IPR011706	Multicopper oxidase, type 2
	superfamily	413	551	1.69E-86	266.818	cl19115	Cupredoxin superfamily	IPR017761	Laccase
	specific	27	143	3.66E-72	228.683	cd13849	CuRO_1_LCC_plant		
	superfamily	27	143	3.66E-72	228.683	cl19115	Cupredoxin superfamily		
	multi-dom	22	568	0	915.665	TIGR03389	laccase		
LAC05_4	specific	160	305	2.38E-86	266.772	cd13875	CuRO_2_LCC_plant	IPR001117	Multicopper oxidase, type 1
	superfamily	160	305	2.38E-86	266.772	cl19115	Cupredoxin superfamily	IPR011707	Multicopper oxidase, type 3
	specific	414	552	4.39E-85	262.966	cd13897	CuRO_3_LCC_plant	IPR011706	Multicopper oxidase, type 2
	superfamily	414	552	4.39E-85	262.966	cl19115	Cupredoxin superfamily	IPR017761	Laccase
	specific	27	143	1.73E-71	226.757	cd13849	CuRO_1_LCC_plant		
	superfamily	27	143	1.73E-71	226.757	cl19115	Cupredoxin superfamily		
	multi-dom	22	569	0	900.643	TIGR03389	laccase		
LAC05_5	specific	132	278	1.35E-92	277.942	cd13875	CuRO_2_LCC_plant	IPR001117	Multicopper oxidase, type 1
	superfamily	132	278	1.35E-92	277.942	cl19115	Cupredoxin superfamily	IPR011707	Multicopper oxidase, type 3
	specific	3	116	9.12E-69	215.586	cd13849	CuRO_1_LCC_plant		
	superfamily	3	116	9.12E-69	215.586	cl19115	Cupredoxin superfamily		

	superfamily	266	396	1.87E-70	220.594	cl19115	Cupredoxin superfamily		
	multi-dom	4	413	0	690.324	TIGR03389	laccase		
LAC05_6	specific	159	305	3.08E-91	279.483	cd13875	CuRO_2_LCC_plant	IPR001117	Multicopper oxidase, type 1
	superfamily	159	305	3.08E-91	279.483	cl19115	Cupredoxin superfamily	IPR011707	Multicopper oxidase, type 3
	specific	413	551	2.24E-84	261.425	cd13897	CuRO_3_LCC_plant	IPR011706	Multicopper oxidase, type 2
	superfamily	413	551	2.24E-84	261.425	cl19115	Cupredoxin superfamily	IPR017761	Laccase
	specific	27	143	3.22E-72	228.683	cd13849	CuRO_1_LCC_plant		
	superfamily	27	143	3.22E-72	228.683	cl19115	Cupredoxin superfamily		
	multi-dom	22	568	0	921.829	TIGR03389	laccase		
LAC06_1	specific	170	317	4.11E-78	245.586	cd13875	CuRO_2_LCC_plant	IPR001117	Multicopper oxidase, type 1
	superfamily	170	317	4.11E-78	245.586	cl19115	Cupredoxin superfamily	IPR011707	Multicopper oxidase, type 3
	specific	419	557	1.12E-74	236.002	cd13897	CuRO_3_LCC_plant	IPR011706	Multicopper oxidase, type 2
	superfamily	419	557	1.12E-74	236.002	cl19115	Cupredoxin superfamily	IPR017761	Laccase
	specific	39	155	5.35E-66	212.505	cd13849	CuRO_1_LCC_plant		
	superfamily	39	155	5.35E-66	212.505	cl19115	Cupredoxin superfamily		
	multi-dom	34	574	0	752.341	TIGR03389	laccase		
LAC06_2	specific	168	315	2.76E-77	243.274	cd13875	CuRO_2_LCC_plant	IPR001117	Multicopper oxidase, type 1
	superfamily	168	315	2.76E-77	243.274	cl19115	Cupredoxin superfamily	IPR011707	Multicopper oxidase, type 3
	specific	417	555	1.37E-73	233.305	cd13897	CuRO_3_LCC_plant	IPR011706	Multicopper oxidase, type 2
	superfamily	417	555	1.37E-73	233.305	cl19115	Cupredoxin superfamily	IPR017761	Laccase
	specific	37	153	2.51E-67	215.971	cd13849	CuRO_1_LCC_plant		
	superfamily	37	153	2.51E-67	215.971	cl19115	Cupredoxin superfamily		
	multi-dom	32	572	0	744.252	TIGR03389	laccase		
LAC06_3	specific	99	253	1.46E-73	232.104	cd13875	CuRO_2_LCC_plant	IPR001117	Multicopper oxidase, type 1
	superfamily	99	253	1.46E-73	232.104	cl19115	Cupredoxin superfamily	IPR011707	Multicopper oxidase, type 3
	specific	359	498	1.00E-71	226.757	cd13897	CuRO_3_LCC_plant	IPR011706	Multicopper oxidase, type 2
	superfamily	359	498	1.00E-71	226.757	cl19115	Cupredoxin superfamily	IPR017761	Laccase
	superfamily	6	84	1.10E-37	134.694	cl19115	Cupredoxin superfamily		
LAC07_2	specific	158	305	1.14E-86	267.542	cd13875	CuRO_2_LCC_plant	IPR001117	Multicopper oxidase, type 1
	superfamily	158	305	1.14E-86	267.542	cl19115	Cupredoxin superfamily	IPR011707	Multicopper oxidase, type 3
	specific	27	143	1.20E-69	221.749	cd13849	CuRO_1_LCC_plant	IPR011706	Multicopper oxidase, type 2
	superfamily	27	143	1.20E-69	221.749	cl19115	Cupredoxin superfamily	IPR017761	Laccase
	superfamily	408	550	1.21E-68	220.209	cl19115	Cupredoxin superfamily		
	multi-dom	22	567	0	771.216	TIGR03389	laccase		
LAC07_3	specific	423	564	3.39E-75	237.543	cd13897	CuRO_3_LCC_plant	IPR001117	Multicopper oxidase, type 1

	superfamily	423	564	3.39E-75	237.543	cl19115	Cupredoxin superfamily	IPR011707	Multicopper oxidase, type 3
	specific	170	317	4.35E-67	216.696	cd13875	CuRO_2_LCC_plant	IPR011706	Multicopper oxidase, type 2
	superfamily	170	317	4.35E-67	216.696	cl19115	Cupredoxin superfamily	IPR017761	Laccase
	specific	39	155	8.34E-63	204.03	cd13849	CuRO_1_LCC_plant		
	superfamily	39	155	8.34E-63	204.03	cl19115	Cupredoxin superfamily		
	multi-dom	34	581	0	694.946	TIGR03389	laccase		
LAC07_4	specific	159	310	5.46E-78	245.2	cd13875	CuRO_2_LCC_plant	IPR001117	Multicopper oxidase, type 1
	superfamily	159	310	5.46E-78	245.2	cl19115	Cupredoxin superfamily	IPR011707	Multicopper oxidase, type 3
	specific	415	556	1.30E-75	238.698	cd13897	CuRO_3_LCC_plant	IPR011706	Multicopper oxidase, type 2
	superfamily	415	556	1.30E-75	238.698	cl19115	Cupredoxin superfamily	IPR017761	Laccase
	specific	28	144	3.35E-64	207.882	cd13849	CuRO_1_LCC_plant		
	superfamily	28	144	3.35E-64	207.882	cl19115	Cupredoxin superfamily		
	multi-dom	23	556	0	712.28	TIGR03389	laccase		
LAC07_5	specific	159	306	1.60E-82	256.756	cd13875	CuRO_2_LCC_plant	IPR001117	Multicopper oxidase, type 1
	superfamily	159	306	1.60E-82	256.756	cl19115	Cupredoxin superfamily	IPR011707	Multicopper oxidase, type 3
	specific	28	144	1.86E-67	215.971	cd13849	CuRO_1_LCC_plant	IPR011706	Multicopper oxidase, type 2
	superfamily	28	144	1.86E-67	215.971	cl19115	Cupredoxin superfamily	IPR017761	Laccase
	superfamily	406	547	5.96E-70	223.29	cl19115	Cupredoxin superfamily		
	multi-dom	23	564	0	718.828	TIGR03389	laccase		
LAC09	specific	159	310	1.37E-80	251.749	cd13875	CuRO_2_LCC_plant	IPR001117	Multicopper oxidase, type 1
	superfamily	159	310	1.37E-80	251.749	cl19115	Cupredoxin superfamily	IPR011707	Multicopper oxidase, type 3
	specific	415	556	3.89E-73	232.15	cd13897	CuRO_3_LCC_plant	IPR011706	Multicopper oxidase, type 2
	superfamily	415	556	3.89E-73	232.15	cl19115	Cupredoxin superfamily	IPR017761	Laccase
	specific	28	144	1.18E-65	211.734	cd13849	CuRO_1_LCC_plant		
	superfamily	28	144	1.18E-65	211.734	cl19115	Cupredoxin superfamily		
	multi-dom	23	556	0	713.821	TIGR03389	laccase		
LAC11_1	specific	13	157	1.79E-88	267.157	cd13875	CuRO_2_LCC_plant	IPR001117	Multicopper oxidase, type 1
	superfamily	13	157	1.79E-88	267.157	cl19115	Cupredoxin superfamily	IPR011706	Multicopper oxidase, type 2
	specific	254	392	1.04E-86	262.581	cd13897	CuRO_3_LCC_plant		
	superfamily	254	392	1.04E-86	262.581	cl19115	Cupredoxin superfamily		
LAC11_2	specific	166	310	5.47E-85	262.92	cd13875	CuRO_2_LCC_plant	IPR001117	Multicopper oxidase, type 1
	superfamily	166	310	5.47E-85	262.92	cl19115	Cupredoxin superfamily	IPR011707	Multicopper oxidase, type 3
	specific	407	545	1.72E-84	261.425	cd13897	CuRO_3_LCC_plant	IPR011706	Multicopper oxidase, type 2
	superfamily	407	545	1.72E-84	261.425	cl19115	Cupredoxin superfamily	IPR017761	Laccase
	specific	33	149	9.66E-72	227.142	cd13849	CuRO_1_LCC_plant		

	superfamily	33	149	9.66E-72	227.142	cl19115	Cupredoxin superfamily		
	multi-dom	28	562	0	989.238	TIGR03389	laccase		
LAC11_3	specific	408	546	6.04E-86	265.277	cd13897	CuRO_3_LCC_plant	IPR001117	Multicopper oxidase, type 1
	superfamily	408	546	6.04E-86	265.277	cl19115	Cupredoxin superfamily	IPR011707	Multicopper oxidase, type 3
	specific	167	311	6.36E-86	265.616	cd13875	CuRO_2_LCC_plant	IPR011706	Multicopper oxidase, type 2
	superfamily	167	311	6.36E-86	265.616	cl19115	Cupredoxin superfamily	IPR017761	Laccase
	specific	34	150	1.93E-69	221.364	cd13849	CuRO_1_LCC_plant		
	superfamily	34	150	1.93E-69	221.364	cl19115	Cupredoxin superfamily		
	multi-dom	29	563	0	970.749	TIGR03389	laccase		
LAC11_4	specific	167	311	1.57E-88	272.164	cd13875	CuRO_2_LCC_plant	IPR001117	Multicopper oxidase, type 1
	superfamily	167	311	1.57E-88	272.164	cl19115	Cupredoxin superfamily	IPR011707	Multicopper oxidase, type 3
	specific	408	546	3.44E-85	263.351	cd13897	CuRO_3_LCC_plant	IPR011706	Multicopper oxidase, type 2
	superfamily	408	546	3.44E-85	263.351	cl19115	Cupredoxin superfamily	IPR017761	Laccase
	specific	34	150	1.81E-69	221.364	cd13849	CuRO_1_LCC_plant		
	superfamily	34	150	1.81E-69	221.364	cl19115	Cupredoxin superfamily		
	multi-dom	29	563	0	988.853	TIGR03389	laccase		
LAC11_6	specific	301	439	9.76E-86	261.425	cd13897	CuRO_3_LCC_plant	IPR001117	Multicopper oxidase, type 1
	superfamily	301	439	9.76E-86	261.425	cl19115	Cupredoxin superfamily	IPR011707	Multicopper oxidase, type 3
	specific	167	297	4.17E-83	254.83	cd13875	CuRO_2_LCC_plant	IPR011706	Multicopper oxidase, type 2
	superfamily	167	297	4.17E-83	254.83	cl19115	Cupredoxin superfamily	IPR017761	Laccase
	specific	34	150	5.66E-70	219.823	cd13849	CuRO_1_LCC_plant		
	superfamily	34	150	5.66E-70	219.823	cl19115	Cupredoxin superfamily		
	multi-dom	29	456	0	776.223	TIGR03389	laccase		
LAC14_1	specific	398	536	2.75E-81	252.951	cd13897	CuRO_3_LCC_plant	IPR001117	Multicopper oxidase, type 1
	superfamily	398	536	2.75E-81	252.951	cl19115	Cupredoxin superfamily	IPR011707	Multicopper oxidase, type 3
	specific	152	298	3.12E-73	232.104	cd13875	CuRO_2_LCC_plant	IPR011706	Multicopper oxidase, type 2
	superfamily	152	298	3.12E-73	232.104	cl19115	Cupredoxin superfamily	IPR017761	Laccase
	specific	21	137	7.78E-62	200.949	cd13849	CuRO_1_LCC_plant		
	superfamily	21	137	7.78E-62	200.949	cl19115	Cupredoxin superfamily		
	multi-dom	16	553	0	695.716	TIGR03389	laccase		
LAC14_2	specific	405	543	6.57E-75	236.387	cd13897	CuRO_3_LCC_plant	IPR001117	Multicopper oxidase, type 1
	superfamily	405	543	6.57E-75	236.387	cl19115	Cupredoxin superfamily	IPR011707	Multicopper oxidase, type 3
	specific	157	303	2.09E-72	230.178	cd13875	CuRO_2_LCC_plant	IPR011706	Multicopper oxidase, type 2
	superfamily	157	303	2.09E-72	230.178	cl19115	Cupredoxin superfamily	IPR017761	Laccase
	specific	26	142	3.43E-61	199.408	cd13849	CuRO_1_LCC_plant		

	superfamily	26	142	3.43E-61	199.408	cl19115	Cupredoxin superfamily		
	multi-dom	21	560	0	682.62	TIGR03389	laccase		
LAC14_3	specific	408	546	1.48E-83	259.114	cd13897	CuRO_3_LCC_plant	IPR001117	Multicopper oxidase, type 1
	superfamily	408	546	1.48E-83	259.114	cl19115	Cupredoxin superfamily	IPR011707	Multicopper oxidase, type 3
	specific	161	307	1.31E-73	233.259	cd13875	CuRO_2_LCC_plant	IPR011706	Multicopper oxidase, type 2
	superfamily	161	307	1.31E-73	233.259	cl19115	Cupredoxin superfamily	IPR017761	Laccase
	specific	30	146	3.29E-61	199.793	cd13849	CuRO_1_LCC_plant		
	superfamily	30	146	3.29E-61	199.793	cl19115	Cupredoxin superfamily		
	multi-dom	25	563	0	679.923	TIGR03389	laccase		
LAC14_4	specific	396	534	1.01E-76	241.009	cd13897	CuRO_3_LCC_plant	IPR001117	Multicopper oxidase, type 1
	superfamily	396	534	1.01E-76	241.009	cl19115	Cupredoxin superfamily	IPR011707	Multicopper oxidase, type 3
	specific	155	294	4.66E-68	218.622	cd13875	CuRO_2_LCC_plant	IPR011706	Multicopper oxidase, type 2
	superfamily	155	294	4.66E-68	218.622	cl19115	Cupredoxin superfamily	IPR017761	Laccase
	specific	24	140	3.14E-61	199.408	cd13849	CuRO_1_LCC_plant		
	superfamily	24	140	3.14E-61	199.408	cl19115	Cupredoxin superfamily		
	multi-dom	19	551	0	669.523	TIGR03389	laccase		
LAC14_5	specific	404	542	8.94E-76	238.698	cd13897	CuRO_3_LCC_plant	IPR001117	Multicopper oxidase, type 1
	superfamily	404	542	8.94E-76	238.698	cl19115	Cupredoxin superfamily	IPR011707	Multicopper oxidase, type 3
	specific	155	301	6.33E-71	226.326	cd13875	CuRO_2_LCC_plant	IPR011706	Multicopper oxidase, type 2
	superfamily	155	301	6.33E-71	226.326	cl19115	Cupredoxin superfamily	IPR017761	Laccase
	specific	24	140	4.85E-64	207.112	cd13849	CuRO_1_LCC_plant		
	superfamily	24	140	4.85E-64	207.112	cl19115	Cupredoxin superfamily		
	multi-dom	19	559	0	674.53	TIGR03389	laccase		
LAC14_6	specific	29	145	4.19E-66	212.89	cd13849	CuRO_1_LCC_plant	IPR001117	Multicopper oxidase, type 1
	superfamily	29	145	4.19E-66	212.89	cl19115	Cupredoxin superfamily	IPR011707	Multicopper oxidase, type 3
	specific	160	309	1.68E-64	209.762	cd13875	CuRO_2_LCC_plant	IPR011706	Multicopper oxidase, type 2
	superfamily	160	309	1.68E-64	209.762	cl19115	Cupredoxin superfamily	IPR017761	Laccase
	superfamily	409	549	2.45E-63	206.342	cl19115	Cupredoxin superfamily		
	multi-dom	24	566	0	657.967	TIGR03389	laccase		
LAC14_7	specific	412	550	1.18E-78	246.402	cd13897	CuRO_3_LCC_plant	IPR001117	Multicopper oxidase, type 1
	superfamily	412	550	1.18E-78	246.402	cl19115	Cupredoxin superfamily	IPR011707	Multicopper oxidase, type 3
	specific	32	148	3.78E-65	210.193	cd13849	CuRO_1_LCC_plant	IPR011706	Multicopper oxidase, type 2
	superfamily	32	148	3.78E-65	210.193	cl19115	Cupredoxin superfamily	IPR017761	Laccase
	specific	163	308	2.25E-64	209.377	cd13875	CuRO_2_LCC_plant		
	superfamily	163	308	2.25E-64	209.377	cl19115	Cupredoxin superfamily		

	multi-dom	27	568	0	660.278	TIGR03389	laccase		
LAC14_8	specific	408	546	9.58E-84	259.499	cd13897	CuRO_3_LCC_plant	IPR001117	Multicopper oxidase, type 1
	superfamily	408	546	9.58E-84	259.499	cl19115	Cupredoxin superfamily	IPR011707	Multicopper oxidase, type 3
	specific	161	307	1.11E-73	233.645	cd13875	CuRO_2_LCC_plant	IPR011706	Multicopper oxidase, type 2
	superfamily	161	307	1.11E-73	233.645	cl19115	Cupredoxin superfamily	IPR017761	Laccase
	specific	30	146	1.07E-62	203.645	cd13849	CuRO_1_LCC_plant		
	superfamily	30	146	1.07E-62	203.645	cl19115	Cupredoxin superfamily		
	multi-dom	25	563	0	681.079	TIGR03389	laccase		
LAC15	specific	164	311	1.26E-83	259.838	cd13875	CuRO_2_LCC_plant	IPR001117	Multicopper oxidase, type 1
	superfamily	164	311	1.26E-83	259.838	cl19115	Cupredoxin superfamily	IPR011707	Multicopper oxidase, type 3
	specific	417	556	8.20E-79	246.787	cd13897	CuRO_3_LCC_plant	IPR011706	Multicopper oxidase, type 2
	superfamily	417	556	8.20E-79	246.787	cl19115	Cupredoxin superfamily	IPR017761	Laccase
	specific	33	149	7.83E-65	209.423	cd13849	CuRO_1_LCC_plant		
	superfamily	33	149	7.83E-65	209.423	cl19115	Cupredoxin superfamily		
	multi-dom	33	573	0	811.276	TIGR03389	laccase		
LAC17_2	specific	422	559	7.48E-84	260.269	cd13897	CuRO_3_LCC_plant	IPR001117	Multicopper oxidase, type 1
	superfamily	422	559	7.48E-84	260.269	cl19115	Cupredoxin superfamily	IPR011707	Multicopper oxidase, type 3
	specific	165	313	5.05E-82	255.601	cd13875	CuRO_2_LCC_plant	IPR011706	Multicopper oxidase, type 2
	superfamily	165	313	5.05E-82	255.601	cl19115	Cupredoxin superfamily	IPR017761	Laccase
	specific	34	149	2.39E-68	218.668	cd13849	CuRO_1_LCC_plant		
	superfamily	34	149	2.39E-68	218.668	cl19115	Cupredoxin superfamily		
	multi-dom	29	576	0	955.726	TIGR03389	laccase		
LAC17_3	specific	422	559	6.12E-85	262.966	cd13897	CuRO_3_LCC_plant	IPR001117	Multicopper oxidase, type 1
	superfamily	422	559	6.12E-85	262.966	cl19115	Cupredoxin superfamily	IPR011707	Multicopper oxidase, type 3
	specific	165	313	2.56E-82	256.371	cd13875	CuRO_2_LCC_plant	IPR011706	Multicopper oxidase, type 2
	superfamily	165	313	2.56E-82	256.371	cl19115	Cupredoxin superfamily	IPR017761	Laccase
	specific	34	150	5.23E-71	225.601	cd13849	CuRO_1_LCC_plant		
	superfamily	34	150	5.23E-71	225.601	cl19115	Cupredoxin superfamily		
	multi-dom	29	576	0	966.897	TIGR03389	laccase		
LAC17_4	specific	425	562	9.84E-84	259.884	cd13897	CuRO_3_LCC_plant	IPR001117	Multicopper oxidase, type 1
	superfamily	425	562	9.84E-84	259.884	cl19115	Cupredoxin superfamily	IPR011707	Multicopper oxidase, type 3
	specific	165	313	4.13E-83	258.682	cd13875	CuRO_2_LCC_plant	IPR011706	Multicopper oxidase, type 2
	superfamily	165	313	4.13E-83	258.682	cl19115	Cupredoxin superfamily	IPR017761	Laccase
	specific	34	150	5.41E-70	223.29	cd13849	CuRO_1_LCC_plant		
	superfamily	34	150	5.41E-70	223.29	cl19115	Cupredoxin superfamily		

	multi-dom	29	579	0	959.193	TIGR03389	laccase		
LAC17_5	specific	171	319	3.78E-86	266.772	cd13875	CuRO_2_LCC_plant	IPR001117	Multicopper oxidase, type 1
	superfamily	171	319	3.78E-86	266.772	cl19115	Cupredoxin superfamily	IPR011707	Multicopper oxidase, type 3
	specific	429	566	3.30E-81	253.336	cd13897	CuRO_3_LCC_plant	IPR011706	Multicopper oxidase, type 2
	superfamily	429	566	3.30E-81	253.336	cl19115	Cupredoxin superfamily	IPR017761	Laccase
	specific	40	156	6.63E-74	233.691	cd13849	CuRO_1_LCC_plant		
	superfamily	40	156	6.63E-74	233.691	cl19115	Cupredoxin superfamily		
	multi-dom	35	583	0	963.045	TIGR03389	laccase		
LAC17_6	specific	171	319	1.04E-86	267.927	cd13875	CuRO_2_LCC_plant	IPR001117	Multicopper oxidase, type 1
	superfamily	171	319	1.04E-86	267.927	cl19115	Cupredoxin superfamily	IPR011707	Multicopper oxidase, type 3
	specific	429	566	4.13E-81	253.336	cd13897	CuRO_3_LCC_plant	IPR011706	Multicopper oxidase, type 2
	superfamily	429	566	4.13E-81	253.336	cl19115	Cupredoxin superfamily	IPR017761	Laccase
	specific	40	156	9.55E-76	238.313	cd13849	CuRO_1_LCC_plant		
	superfamily	40	156	9.55E-76	238.313	cl19115	Cupredoxin superfamily		
	multi-dom	35	583	0	952.259	TIGR03389	laccase		
LAC17_7	specific	163	311	1.85E-81	254.06	cd13875	CuRO_2_LCC_plant	IPR001117	Multicopper oxidase, type 1
	superfamily	163	311	1.85E-81	254.06	cl19115	Cupredoxin superfamily	IPR011707	Multicopper oxidase, type 3
	specific	419	556	1.91E-80	251.025	cd13897	CuRO_3_LCC_plant	IPR011706	Multicopper oxidase, type 2
	superfamily	419	556	1.91E-80	251.025	cl19115	Cupredoxin superfamily	IPR017761	Laccase
	specific	32	148	1.84E-66	213.66	cd13849	CuRO_1_LCC_plant		
	superfamily	32	148	1.84E-66	213.66	cl19115	Cupredoxin superfamily		
	multi-dom	27	573	0	925.681	TIGR03389	laccase		
LAC17_8	specific	158	306	6.17E-83	257.912	cd13875	CuRO_2_LCC_plant	IPR001117	Multicopper oxidase, type 1
	superfamily	158	306	6.17E-83	257.912	cl19115	Cupredoxin superfamily	IPR011707	Multicopper oxidase, type 3
	specific	416	553	4.22E-79	247.558	cd13897	CuRO_3_LCC_plant	IPR011706	Multicopper oxidase, type 2
	superfamily	416	553	4.22E-79	247.558	cl19115	Cupredoxin superfamily	IPR017761	Laccase
	specific	27	143	2.02E-64	208.268	cd13849	CuRO_1_LCC_plant		
	superfamily	27	143	2.02E-64	208.268	cl19115	Cupredoxin superfamily		
	multi-dom	22	570	0	920.288	TIGR03389	laccase		
LAC17_9	specific	149	260	8.69E-68	216.696	cd13875	CuRO_2_LCC_plant	IPR001117	Multicopper oxidase, type 1
	superfamily	149	260	8.69E-68	216.696	cl19115	Cupredoxin superfamily	IPR011707	Multicopper oxidase, type 3
	superfamily	356	493	6.14E-70	222.135	cl19115	Cupredoxin superfamily	IPR011706	Multicopper oxidase, type 2
	superfamily	35	134	6.00E-47	160.118	cl19115	Cupredoxin superfamily		
	multi-dom	30	508	0	759.66	TIGR03389	laccase		

Supplementary Table S3C: Domain prediction analysis of all the identified Laccase family proteins from *G. hirsutum* A-subgenome.

	Conserved Domain Database							InterProScan	
**Query**	**Hit type**	**From**	**To**	**E-Value**	**Bitscore**	**Accession**	**Short name**	**Accession**	**Short name**
LAC02_1	specific	170	318	1.43E-83	259.838	cd13875	CuRO_2_LCC_plant	IPR001117	Multicopper oxidase, type 1
	superfamily	170	318	1.43E-83	259.838	cl19115	Cupredoxin superfamily	IPR011707	Multicopper oxidase, type 3
	specific	422	560	1.16E-80	251.795	cd13897	CuRO_3_LCC_plant	IPR011706	Multicopper oxidase, type 2
	superfamily	422	560	1.16E-80	251.795	cl19115	Cupredoxin superfamily	IPR017761	Laccase
	specific	39	155	6.08E-72	228.298	cd13849	CuRO_1_LCC_plant		
	superfamily	39	155	6.08E-72	228.298	cl19115	Cupredoxin superfamily		
	multi-dom	36	577	0	951.489	TIGR03389	laccase		
LAC02_2	specific	36	152	1.82E-72	228.298	cd13849	CuRO_1_LCC_plant	IPR001117	Multicopper oxidase, type 1
	superfamily	36	152	1.82E-72	228.298	cl19115	Cupredoxin superfamily	IPR011707	Multicopper oxidase, type 3
	specific	167	300	1.27E-65	211.303	cd13875	CuRO_2_LCC_plant	IPR011706	Multicopper oxidase, type 2
	superfamily	167	300	1.27E-65	211.303	cl19115	Cupredoxin superfamily	IPR017761	Laccase
	superfamily	408	520	3.61E-61	199.408	cl19115	Cupredoxin superfamily		
	multi-dom	31	520	0	800.876	TIGR03389	laccase		
LAC02_4	specific	423	560	2.91E-80	250.639	cd13897	CuRO_3_LCC_plant	IPR001117	Multicopper oxidase, type 1
	superfamily	423	560	2.91E-80	250.639	cl19115	Cupredoxin superfamily	IPR011707	Multicopper oxidase, type 3
	specific	167	315	3.99E-80	250.593	cd13875	CuRO_2_LCC_plant	IPR011706	Multicopper oxidase, type 2
	superfamily	167	315	3.99E-80	250.593	cl19115	Cupredoxin superfamily	IPR017761	Laccase
	specific	36	152	1.18E-70	224.831	cd13849	CuRO_1_LCC_plant		
	superfamily	36	152	1.18E-70	224.831	cl19115	Cupredoxin superfamily		
	multi-dom	31	577	0	936.081	TIGR03389	laccase		
LAC03_1	specific	168	314	6.06E-89	273.705	cd13875	CuRO_2_LCC_plant	IPR001117	Multicopper oxidase, type 1
	superfamily	168	314	6.06E-89	273.705	cl19115	Cupredoxin superfamily	IPR011707	Multicopper oxidase, type 3
	specific	421	559	1.92E-80	251.41	cd13897	CuRO_3_LCC_plant	IPR011706	Multicopper oxidase, type 2
	superfamily	421	559	1.92E-80	251.41	cl19115	Cupredoxin superfamily	IPR017761	Laccase
	specific	37	153	6.55E-73	230.609	cd13849	CuRO_1_LCC_plant		
	superfamily	37	153	6.55E-73	230.609	cl19115	Cupredoxin superfamily		
	multi-dom	32	576	0	842.477	TIGR03389	laccase		
LAC03_2	specific	169	314	4.81E-89	274.09	cd13875	CuRO_2_LCC_plant	IPR001117	Multicopper oxidase, type 1
	superfamily	169	314	4.81E-89	274.09	cl19115	Cupredoxin superfamily	IPR011707	Multicopper oxidase, type 3
	specific	421	560	3.33E-81	253.336	cd13897	CuRO_3_LCC_plant	IPR011706	Multicopper oxidase, type 2
	superfamily	421	560	3.33E-81	253.336	cl19115	Cupredoxin superfamily	IPR017761	Laccase
	specific	37	153	5.89E-72	228.298	cd13849	CuRO_1_LCC_plant		
	superfamily	37	153	5.89E-72	228.298	cl19115	Cupredoxin superfamily		

	multi-dom	34	577	0	847.485	TIGR03389	laccase		
LAC04_1	specific	404	541	3.20E-80	250.254	cd13897	CuRO_3_LCC_plant	IPR001117	Multicopper oxidase, type 1
	superfamily	404	541	3.20E-80	250.254	cl19115	Cupredoxin superfamily	IPR011707	Multicopper oxidase, type 3
	specific	159	305	7.77E-75	236.341	cd13875	CuRO_2_LCC_plant	IPR011706	Multicopper oxidase, type 2
	superfamily	159	305	7.77E-75	236.341	cl19115	Cupredoxin superfamily	IPR017761	Laccase
	specific	28	144	1.05E-59	195.556	cd13849	CuRO_1_LCC_plant		
	superfamily	28	144	1.05E-59	195.556	cl19115	Cupredoxin superfamily		
	multi-dom	24	558	0	924.14	TIGR03389	laccase		
LAC04_2	specific	402	539	2.22E-81	252.951	cd13897	CuRO_3_LCC_plant	IPR001117	Multicopper oxidase, type 1
	superfamily	402	539	2.22E-81	252.951	cl19115	Cupredoxin superfamily	IPR011707	Multicopper oxidase, type 3
	specific	158	303	6.20E-80	249.823	cd13875	CuRO_2_LCC_plant	IPR011706	Multicopper oxidase, type 2
	superfamily	158	303	6.20E-80	249.823	cl19115	Cupredoxin superfamily	IPR017761	Laccase
	specific	26	142	3.92E-69	220.209	cd13849	CuRO_1_LCC_plant		
	superfamily	26	142	3.92E-69	220.209	cl19115	Cupredoxin superfamily		
	multi-dom	21	556	0	978.453	TIGR03389	laccase		
LAC04_4	specific	402	539	8.79E-79	246.402	cd13897	CuRO_3_LCC_plant	IPR001117	Multicopper oxidase, type 1
	superfamily	402	539	8.79E-79	246.402	cl19115	Cupredoxin superfamily	IPR011707	Multicopper oxidase, type 3
	specific	161	305	1.69E-78	245.971	cd13875	CuRO_2_LCC_plant	IPR011706	Multicopper oxidase, type 2
	superfamily	161	305	1.69E-78	245.971	cl19115	Cupredoxin superfamily	IPR017761	Laccase
	specific	28	144	5.50E-72	227.913	cd13849	CuRO_1_LCC_plant		
	superfamily	28	144	5.50E-72	227.913	cl19115	Cupredoxin superfamily		
	multi-dom	23	556	0	974.601	TIGR03389	laccase		
LAC04_6	specific	402	539	9.18E-79	246.402	cd13897	CuRO_3_LCC_plant	IPR001117	Multicopper oxidase, type 1
	superfamily	402	539	9.18E-79	246.402	cl19115	Cupredoxin superfamily	IPR011707	Multicopper oxidase, type 3
	specific	162	305	1.20E-76	240.963	cd13875	CuRO_2_LCC_plant	IPR011706	Multicopper oxidase, type 2
	superfamily	162	305	1.20E-76	240.963	cl19115	Cupredoxin superfamily	IPR017761	Laccase
	specific	29	145	1.19E-70	224.446	cd13849	CuRO_1_LCC_plant		
	superfamily	29	145	1.19E-70	224.446	cl19115	Cupredoxin superfamily		
	multi-dom	24	556	0	958.423	TIGR03389	laccase		
LAC04_8	specific	401	538	5.38E-79	246.787	cd13897	CuRO_3_LCC_plant	IPR001117	Multicopper oxidase, type 1
	superfamily	401	538	5.38E-79	246.787	cl19115	Cupredoxin superfamily	IPR011707	Multicopper oxidase, type 3
	specific	27	143	1.75E-68	218.668	cd13849	CuRO_1_LCC_plant	IPR011706	Multicopper oxidase, type 2
	superfamily	27	143	1.75E-68	218.668	cl19115	Cupredoxin superfamily	IPR017761	Laccase
	specific	159	304	3.39E-68	219.007	cd13875	CuRO_2_LCC_plant		
	superfamily	159	304	3.39E-68	219.007	cl19115	Cupredoxin superfamily		
	multi-dom	23	555	0	925.295	TIGR03389	laccase		

LAC05_2	specific	159	305	1.32E-87	269.853	cd13875	CuRO_2_LCC_plant	IPR001117	Multicopper oxidase, type 1
	superfamily	159	305	1.32E-87	269.853	cl19115	Cupredoxin superfamily	IPR011707	Multicopper oxidase, type 3
	specific	413	551	1.79E-85	264.121	cd13897	CuRO_3_LCC_plant	IPR011706	Multicopper oxidase, type 2
	superfamily	413	551	1.79E-85	264.121	cl19115	Cupredoxin superfamily	IPR017761	Laccase
	specific	27	143	2.09E-72	229.068	cd13849	CuRO_1_LCC_plant		
	superfamily	27	143	2.09E-72	229.068	cl19115	Cupredoxin superfamily		
	multi-dom	22	568	0	910.273	TIGR03389	laccase		
LAC05_3	specific	159	305	4.23E-90	276.402	cd13875	CuRO_2_LCC_plant	IPR001117	Multicopper oxidase, type 1
	superfamily	159	305	4.23E-90	276.402	cl19115	Cupredoxin superfamily	IPR011707	Multicopper oxidase, type 3
	specific	413	551	3.11E-86	266.047	cd13897	CuRO_3_LCC_plant	IPR011706	Multicopper oxidase, type 2
	superfamily	413	551	3.11E-86	266.047	cl19115	Cupredoxin superfamily	IPR017761	Laccase
	specific	27	143	4.11E-72	228.298	cd13849	CuRO_1_LCC_plant		
	superfamily	27	143	4.11E-72	228.298	cl19115	Cupredoxin superfamily		
	multi-dom	22	568	0	918.362	TIGR03389	laccase		
LAC05_4	specific	160	305	1.17E-87	270.238	cd13875	CuRO_2_LCC_plant	IPR001117	Multicopper oxidase, type 1
	superfamily	160	305	1.17E-87	270.238	cl19115	Cupredoxin superfamily	IPR011707	Multicopper oxidase, type 3
	specific	414	552	1.51E-84	261.81	cd13897	CuRO_3_LCC_plant	IPR011706	Multicopper oxidase, type 2
	superfamily	414	552	1.51E-84	261.81	cl19115	Cupredoxin superfamily	IPR017761	Laccase
	specific	27	143	2.27E-71	226.372	cd13849	CuRO_1_LCC_plant		
	superfamily	27	143	2.27E-71	226.372	cl19115	Cupredoxin superfamily		
	multi-dom	22	569	0	901.798	TIGR03389	laccase		
LAC06_1	specific	164	311	7.40E-80	244.045	cd13875	CuRO_2_LCC_plant	IPR001117	Multicopper oxidase, type 1
	superfamily	164	311	7.40E-80	244.045	cl19115	Cupredoxin superfamily	IPR011707	Multicopper oxidase, type 3
	specific	33	149	1.38E-69	216.357	cd13849	CuRO_1_LCC_plant		
	superfamily	33	149	1.38E-69	216.357	cl19115	Cupredoxin superfamily		
LAC06_2	specific	194	338	1.49E-74	236.726	cd13875	CuRO_2_LCC_plant	IPR001117	Multicopper oxidase, type 1
	superfamily	194	338	1.49E-74	236.726	cl19115	Cupredoxin superfamily	IPR011707	Multicopper oxidase, type 3
	specific	440	578	7.73E-73	231.765	cd13897	CuRO_3_LCC_plant	IPR011706	Multicopper oxidase, type 2
	superfamily	440	578	7.73E-73	231.765	cl19115	Cupredoxin superfamily	IPR017761	Laccase
	specific	37	153	1.95E-67	216.742	cd13849	CuRO_1_LCC_plant		
	superfamily	37	153	1.95E-67	216.742	cl19115	Cupredoxin superfamily		
	multi-dom	32	595	0	725.762	TIGR03389	laccase		
LAC06_3	specific	108	254	1.05E-73	224.4	cd13875	CuRO_2_LCC_plant	IPR001117	Multicopper oxidase, type 1
	superfamily	108	254	1.05E-73	224.4	cl19115	Cupredoxin superfamily	IPR011707	Multicopper oxidase, type 3
	specific	1	93	2.87E-52	168.592	cd13849	CuRO_1_LCC_plant		
	superfamily	1	93	2.87E-52	168.592	cl19115	Cupredoxin superfamily		

LAC07_1	specific	159	310	2.73E-80	250.978	cd13875	CuRO_2_LCC_plant	IPR001117	Multicopper oxidase, type 1
	superfamily	159	310	2.73E-80	250.978	cl19115	Cupredoxin superfamily	IPR011707	Multicopper oxidase, type 3
	specific	415	556	1.16E-76	241.395	cd13897	CuRO_3_LCC_plant	IPR011706	Multicopper oxidase, type 2
	superfamily	415	556	1.16E-76	241.395	cl19115	Cupredoxin superfamily	IPR017761	Laccase
	specific	28	144	3.02E-64	207.882	cd13849	CuRO_1_LCC_plant		
	superfamily	28	144	3.02E-64	207.882	cl19115	Cupredoxin superfamily		
	multi-dom	23	573	0	728.073	TIGR03389	laccase		
LAC07_2	specific	158	305	9.47E-86	265.231	cd13875	CuRO_2_LCC_plant	IPR001117	Multicopper oxidase, type 1
	superfamily	158	305	9.47E-86	265.231	cl19115	Cupredoxin superfamily	IPR011707	Multicopper oxidase, type 3
	specific	27	143	1.10E-70	224.831	cd13849	CuRO_1_LCC_plant	IPR011706	Multicopper oxidase, type 2
	superfamily	27	143	1.10E-70	224.831	cl19115	Cupredoxin superfamily	IPR017761	Laccase
	superfamily	408	550	1.28E-70	225.216	cl19115	Cupredoxin superfamily		
	multi-dom	22	567	0	771.986	TIGR03389	laccase		
LAC07_5	specific	159	306	4.21E-82	255.601	cd13875	CuRO_2_LCC_plant	IPR001117	Multicopper oxidase, type 1
	superfamily	159	306	4.21E-82	255.601	cl19115	Cupredoxin superfamily	IPR011707	Multicopper oxidase, type 3
	specific	28	144	7.31E-68	217.127	cd13849	CuRO_1_LCC_plant	IPR011706	Multicopper oxidase, type 2
	superfamily	28	144	7.31E-68	217.127	cl19115	Cupredoxin superfamily	IPR017761	Laccase
	superfamily	406	547	1.54E-69	222.52	cl19115	Cupredoxin superfamily		
	multi-dom	24	564	0	718.443	TIGR03389	laccase		
LAC11_1	specific	167	311	4.75E-87	268.312	cd13875	CuRO_2_LCC_plant	IPR001117	Multicopper oxidase, type 1
	superfamily	167	311	4.75E-87	268.312	cl19115	Cupredoxin superfamily	IPR011707	Multicopper oxidase, type 3
	specific	408	546	1.21E-85	264.507	cd13897	CuRO_3_LCC_plant	IPR011706	Multicopper oxidase, type 2
	superfamily	408	546	1.21E-85	264.507	cl19115	Cupredoxin superfamily	IPR017761	Laccase
	specific	34	150	7.72E-70	222.52	cd13849	CuRO_1_LCC_plant		
	superfamily	34	150	7.72E-70	222.52	cl19115	Cupredoxin superfamily		
	multi-dom	29	563	0	977.297	TIGR03389	laccase		
LAC11_2	specific	166	310	2.38E-85	264.075	cd13875	CuRO_2_LCC_plant	IPR001117	Multicopper oxidase, type 1
	superfamily	166	310	2.38E-85	264.075	cl19115	Cupredoxin superfamily	IPR011707	Multicopper oxidase, type 3
	specific	407	545	1.21E-84	261.81	cd13897	CuRO_3_LCC_plant	IPR011706	Multicopper oxidase, type 2
	superfamily	407	545	1.21E-84	261.81	cl19115	Cupredoxin superfamily	IPR017761	Laccase
	specific	33	149	7.98E-72	227.527	cd13849	CuRO_1_LCC_plant		
	superfamily	33	149	7.98E-72	227.527	cl19115	Cupredoxin superfamily		
	multi-dom	28	562	0	990.009	TIGR03389	laccase		
LAC11_3	specific	112	256	6.22E-87	265.231	cd13875	CuRO_2_LCC_plant	IPR001117	Multicopper oxidase, type 1
	superfamily	112	256	6.22E-87	265.231	cl19115	Cupredoxin superfamily	IPR011707	Multicopper oxidase, type 3
	superfamily	353	465	8.45E-65	207.882	cl19115	Cupredoxin superfamily	IPR011706	Multicopper oxidase, type 2

	superfamily	34	117	2.87E-19	83.4628	cl19115	Cupredoxin superfamily	IPR017761	Laccase
	multi-dom	29	465	0	736.548	TIGR03389	laccase		
LAC11_4	specific	164	309	3.30E-85	263.69	cd13875	CuRO_2_LCC_plant	IPR001117	Multicopper oxidase, type 1
	superfamily	164	309	3.30E-85	263.69	cl19115	Cupredoxin superfamily	IPR011707	Multicopper oxidase, type 3
	specific	406	544	5.82E-85	262.581	cd13897	CuRO_3_LCC_plant	IPR011706	Multicopper oxidase, type 2
	superfamily	406	544	5.82E-85	262.581	cl19115	Cupredoxin superfamily	IPR017761	Laccase
	specific	34	147	3.63E-67	215.201	cd13849	CuRO_1_LCC_plant		
	superfamily	34	147	3.63E-67	215.201	cl19115	Cupredoxin superfamily		
	multi-dom	29	561	0	968.053	TIGR03389	laccase		
LAC11_7	specific	341	479	1.41E-86	264.892	cd13897	CuRO_3_LCC_plant	IPR001117	Multicopper oxidase, type 1
	superfamily	341	479	1.41E-86	264.892	cl19115	Cupredoxin superfamily	IPR011707	Multicopper oxidase, type 3
	specific	90	244	7.80E-82	252.904	cd13875	CuRO_2_LCC_plant	IPR011706	Multicopper oxidase, type 2
	superfamily	90	244	7.80E-82	252.904	cl19115	Cupredoxin superfamily	IPR017761	Laccase
	superfamily	34	87	2.53E-22	91.9372	cl19115	Cupredoxin superfamily		
	multi-dom	29	496	0	769.675	TIGR03389	laccase		
LAC12	specific	105	251	6.46E-87	266.386	cd13875	CuRO_2_LCC_plant	IPR001117	Multicopper oxidase, type 1
	superfamily	105	251	6.46E-87	266.386	cl19115	Cupredoxin superfamily	IPR011707	Multicopper oxidase, type 3
	specific	355	493	7.80E-85	260.655	cd13897	CuRO_3_LCC_plant	IPR011706	Multicopper oxidase, type 2
	superfamily	355	493	7.80E-85	260.655	cl19115	Cupredoxin superfamily	IPR017761	Laccase
	superfamily	3	89	1.14E-43	151.258	cl19115	Cupredoxin superfamily		
	multi-dom	4	510	0	825.529	TIGR03389	laccase		
LAC14_1	specific	161	307	4.79E-73	231.333	cd13875	CuRO_2_LCC_plant	IPR001117	Multicopper oxidase, type 1
	superfamily	161	307	4.79E-73	231.333	cl19115	Cupredoxin superfamily	IPR011707	Multicopper oxidase, type 3
	specific	30	146	3.55E-63	204.03	cd13849	CuRO_1_LCC_plant	IPR011706	Multicopper oxidase, type 2
	superfamily	30	146	3.55E-63	204.03	cl19115	Cupredoxin superfamily	IPR017761	Laccase
	superfamily	407	520	4.62E-58	191.319	cl19115	Cupredoxin superfamily		
	multi-dom	25	520	0	624.454	TIGR03389	laccase		
LAC14_2	specific	405	543	3.78E-75	237.157	cd13897	CuRO_3_LCC_plant	IPR001117	Multicopper oxidase, type 1
	superfamily	405	543	3.78E-75	237.157	cl19115	Cupredoxin superfamily	IPR011707	Multicopper oxidase, type 3
	specific	157	303	7.43E-69	220.933	cd13875	CuRO_2_LCC_plant	IPR011706	Multicopper oxidase, type 2
	superfamily	157	303	7.43E-69	220.933	cl19115	Cupredoxin superfamily	IPR017761	Laccase
	specific	26	142	2.04E-61	200.178	cd13849	CuRO_1_LCC_plant		
	superfamily	26	142	2.04E-61	200.178	cl19115	Cupredoxin superfamily		
	multi-dom	21	560	0	676.071	TIGR03389	laccase		
LAC14_3	specific	408	546	7.26E-84	259.884	cd13897	CuRO_3_LCC_plant	IPR001117	Multicopper oxidase, type 1
	superfamily	408	546	7.26E-84	259.884	cl19115	Cupredoxin superfamily	IPR011707	Multicopper oxidase, type 3

	specific	161	307	6.47E-73	231.719	cd13875	CuRO_2_LCC_plant	IPR011706	Multicopper oxidase, type 2
	superfamily	161	307	6.47E-73	231.719	cl19115	Cupredoxin superfamily	IPR017761	Laccase
	specific	30	146	7.97E-62	201.334	cd13849	CuRO_1_LCC_plant		
	superfamily	30	146	7.97E-62	201.334	cl19115	Cupredoxin superfamily		
	multi-dom	25	563	0	672.99	TIGR03389	laccase		
LAC14_4	specific	405	543	3.04E-76	239.854	cd13897	CuRO_3_LCC_plant	IPR001117	Multicopper oxidase, type 1
	superfamily	405	543	3.04E-76	239.854	cl19115	Cupredoxin superfamily	IPR011707	Multicopper oxidase, type 3
	specific	155	301	4.39E-69	221.318	cd13875	CuRO_2_LCC_plant	IPR011706	Multicopper oxidase, type 2
	superfamily	155	301	4.39E-69	221.318	cl19115	Cupredoxin superfamily	IPR017761	Laccase
	specific	24	140	7.59E-63	204.03	cd13849	CuRO_1_LCC_plant		
	superfamily	24	140	7.59E-63	204.03	cl19115	Cupredoxin superfamily		
	multi-dom	19	560	0	671.064	TIGR03389	laccase		
LAC14_6	specific	29	145	5.70E-68	217.897	cd13849	CuRO_1_LCC_plant	IPR001117	Multicopper oxidase, type 1
	superfamily	29	145	5.70E-68	217.897	cl19115	Cupredoxin superfamily	IPR011707	Multicopper oxidase, type 3
	specific	160	309	1.11E-64	210.147	cd13875	CuRO_2_LCC_plant	IPR011706	Multicopper oxidase, type 2
	superfamily	160	309	1.11E-64	210.147	cl19115	Cupredoxin superfamily	IPR017761	Laccase
	superfamily	409	548	2.47E-64	209.038	cl19115	Cupredoxin superfamily		
	multi-dom	24	565	0	666.826	TIGR03389	laccase		
LAC14_7	specific	412	550	2.57E-78	245.632	cd13897	CuRO_3_LCC_plant	IPR001117	Multicopper oxidase, type 1
	superfamily	412	550	2.57E-78	245.632	cl19115	Cupredoxin superfamily	IPR011707	Multicopper oxidase, type 3
	specific	32	148	3.37E-64	207.497	cd13849	CuRO_1_LCC_plant	IPR011706	Multicopper oxidase, type 2
	superfamily	32	148	3.37E-64	207.497	cl19115	Cupredoxin superfamily	IPR017761	Laccase
	specific	163	308	2.42E-63	206.681	cd13875	CuRO_2_LCC_plant		
	superfamily	163	308	2.42E-63	206.681	cl19115	Cupredoxin superfamily		
	multi-dom	27	568	0	656.426	TIGR03389	laccase		
LAC14_8	specific	408	546	1.53E-83	259.114	cd13897	CuRO_3_LCC_plant	IPR001117	Multicopper oxidase, type 1
	superfamily	408	546	1.53E-83	259.114	cl19115	Cupredoxin superfamily	IPR011707	Multicopper oxidase, type 3
	specific	161	307	4.66E-73	232.104	cd13875	CuRO_2_LCC_plant	IPR011706	Multicopper oxidase, type 2
	superfamily	161	307	4.66E-73	232.104	cl19115	Cupredoxin superfamily	IPR017761	Laccase
	specific	30	146	7.38E-63	204.03	cd13849	CuRO_1_LCC_plant		
	superfamily	30	146	7.38E-63	204.03	cl19115	Cupredoxin superfamily		
	multi-dom	25	563	0	681.464	TIGR03389	laccase		
LAC15	specific	164	311	4.85E-84	260.994	cd13875	CuRO_2_LCC_plant	IPR001117	Multicopper oxidase, type 1
	superfamily	164	311	4.85E-84	260.994	cl19115	Cupredoxin superfamily	IPR011707	Multicopper oxidase, type 3
	specific	418	557	2.13E-78	246.017	cd13897	CuRO_3_LCC_plant	IPR011706	Multicopper oxidase, type 2
	superfamily	418	557	2.13E-78	246.017	cl19115	Cupredoxin superfamily	IPR017761	Laccase

	specific	33	149	9.94E-65	209.038	cd13849	CuRO_1_LCC_plant		
	superfamily	33	149	9.94E-65	209.038	cl19115	Cupredoxin superfamily		
	multi-dom	33	574	0	809.735	TIGR03389	laccase		
LAC17_1	specific	165	312	6.55E-80	245.971	cd13875	CuRO_2_LCC_plant	IPR001117	Multicopper oxidase, type 1
	superfamily	165	312	6.55E-80	245.971	cl19115	Cupredoxin superfamily	IPR011707	Multicopper oxidase, type 3
	specific	34	150	6.83E-71	221.749	cd13849	CuRO_1_LCC_plant	IPR011706	Multicopper oxidase, type 2
	superfamily	34	150	6.83E-71	221.749	cl19115	Cupredoxin superfamily		
	superfamily	322	434	5.03E-61	196.712	cl19115	Cupredoxin superfamily		
	multi-dom	29	434	0	691.479	TIGR03389	laccase		
LAC17_2	specific	165	313	7.21E-83	257.912	cd13875	CuRO_2_LCC_plant	IPR001117	Multicopper oxidase, type 1
	superfamily	165	313	7.21E-83	257.912	cl19115	Cupredoxin superfamily	IPR011707	Multicopper oxidase, type 3
	specific	422	559	1.46E-82	256.803	cd13897	CuRO_3_LCC_plant	IPR011706	Multicopper oxidase, type 2
	superfamily	422	559	1.46E-82	256.803	cl19115	Cupredoxin superfamily	IPR017761	Laccase
	specific	34	150	2.69E-69	221.364	cd13849	CuRO_1_LCC_plant		
	superfamily	34	150	2.69E-69	221.364	cl19115	Cupredoxin superfamily		
	multi-dom	29	576	0	962.66	TIGR03389	laccase		
LAC17_3	specific	165	313	2.14E-81	252.904	cd13875	CuRO_2_LCC_plant	IPR001117	Multicopper oxidase, type 1
	superfamily	165	313	2.14E-81	252.904	cl19115	Cupredoxin superfamily	IPR011707	Multicopper oxidase, type 3
	specific	34	150	6.72E-69	219.053	cd13849	CuRO_1_LCC_plant	IPR011706	Multicopper oxidase, type 2
	superfamily	34	150	6.72E-69	219.053	cl19115	Cupredoxin superfamily	IPR017761	Laccase
	superfamily	422	511	2.88E-40	142.784	cl19115	Cupredoxin superfamily		
	multi-dom	29	528	0	835.159	TIGR03389	laccase		
LAC17_4	specific	425	562	7.54E-84	260.269	cd13897	CuRO_3_LCC_plant	IPR001117	Multicopper oxidase, type 1
	superfamily	425	562	7.54E-84	260.269	cl19115	Cupredoxin superfamily	IPR011707	Multicopper oxidase, type 3
	specific	165	313	2.48E-81	254.06	cd13875	CuRO_2_LCC_plant	IPR011706	Multicopper oxidase, type 2
	superfamily	165	313	2.48E-81	254.06	cl19115	Cupredoxin superfamily	IPR017761	Laccase
	specific	34	150	2.76E-70	224.061	cd13849	CuRO_1_LCC_plant		
	superfamily	34	150	2.76E-70	224.061	cl19115	Cupredoxin superfamily		
	multi-dom	29	579	0	959.578	TIGR03389	laccase		
LAC17_5	specific	171	319	1.70E-84	262.149	cd13875	CuRO_2_LCC_plant	IPR001117	Multicopper oxidase, type 1
	superfamily	171	319	1.70E-84	262.149	cl19115	Cupredoxin superfamily	IPR011707	Multicopper oxidase, type 3
	specific	429	566	5.91E-82	255.262	cd13897	CuRO_3_LCC_plant	IPR011706	Multicopper oxidase, type 2
	superfamily	429	566	5.91E-82	255.262	cl19115	Cupredoxin superfamily	IPR017761	Laccase
	specific	40	156	1.01E-73	232.92	cd13849	CuRO_1_LCC_plant		
	superfamily	40	156	1.01E-73	232.92	cl19115	Cupredoxin superfamily		
	multi-dom	35	583	0	959.963	TIGR03389	laccase		

LAC17_6	specific	171	319	7.89E-86	265.616	cd13875	CuRO_2_LCC_plant	IPR001117	Multicopper oxidase, type 1
	superfamily	171	319	7.89E-86	265.616	cl19115	Cupredoxin superfamily	IPR011707	Multicopper oxidase, type 3
	specific	429	566	2.59E-80	251.025	cd13897	CuRO_3_LCC_plant	IPR011706	Multicopper oxidase, type 2
	superfamily	429	566	2.59E-80	251.025	cl19115	Cupredoxin superfamily	IPR017761	Laccase
	specific	40	156	1.56E-74	235.231	cd13849	CuRO_1_LCC_plant		
	superfamily	40	156	1.56E-74	235.231	cl19115	Cupredoxin superfamily		
	multi-dom	35	583	0	948.022	TIGR03389	laccase		
LAC17_7	specific	419	556	1.92E-81	253.721	cd13897	CuRO_3_LCC_plant	IPR001117	Multicopper oxidase, type 1
	superfamily	419	556	1.92E-81	253.721	cl19115	Cupredoxin superfamily	IPR011707	Multicopper oxidase, type 3
	specific	163	311	2.60E-78	245.971	cd13875	CuRO_2_LCC_plant	IPR011706	Multicopper oxidase, type 2
	superfamily	163	311	2.60E-78	245.971	cl19115	Cupredoxin superfamily	IPR017761	Laccase
	specific	32	148	1.05E-65	211.734	cd13849	CuRO_1_LCC_plant		
	superfamily	32	148	1.05E-65	211.734	cl19115	Cupredoxin superfamily		
	multi-dom	27	573	0	909.887	TIGR03389	laccase		
LAC17_8	specific	158	306	2.44E-83	259.068	cd13875	CuRO_2_LCC_plant	IPR001117	Multicopper oxidase, type 1
	superfamily	158	306	2.44E-83	259.068	cl19115	Cupredoxin superfamily	IPR011707	Multicopper oxidase, type 3
	specific	416	553	8.30E-80	249.484	cd13897	CuRO_3_LCC_plant	IPR011706	Multicopper oxidase, type 2
	superfamily	416	553	8.30E-80	249.484	cl19115	Cupredoxin superfamily	IPR017761	Laccase
	specific	27	143	3.60E-64	207.497	cd13849	CuRO_1_LCC_plant		
	superfamily	27	143	3.60E-64	207.497	cl19115	Cupredoxin superfamily		
	multi-dom	22	570	0	924.525	TIGR03389	laccase		
LAC17_9	specific	357	494	1.09E-79	247.558	cd13897	CuRO_3_LCC_plant	IPR001117	Multicopper oxidase, type 1
	superfamily	357	494	1.09E-79	247.558	cl19115	Cupredoxin superfamily	IPR011707	Multicopper oxidase, type 3
	specific	156	261	1.10E-61	200.903	cd13875	CuRO_2_LCC_plant	IPR011706	Multicopper oxidase, type 2
	superfamily	156	261	1.10E-61	200.903	cl19115	Cupredoxin superfamily		
	superfamily	35	141	1.02E-47	162.429	cl19115	Cupredoxin superfamily		
	multi-dom	30	511	0	775.838	TIGR03389	laccase		

Supplementary Table S3D: Domain prediction analysis of all the identified Laccase family proteins from *G. hirsutum* D-subgenome.

	Conserved Domain Database							InterProScan	
**Query**	**Hit type**	**From**	**To**	**E-Value**	**Bitscore**	**Accession**	**Short name**	**Accession**	**short name**
LAC02_1	specific	170	318	8.74E-81	252.519	cd13875	CuRO_2_LCC_plant	IPR001117	Multicopper oxidase, type 1
	superfamily	170	318	8.74E-81	252.519	cl19115	Cupredoxin superfamily	IPR011707	Multicopper oxidase, type 3
	specific	422	560	2.41E-80	251.025	cd13897	CuRO_3_LCC_plant	IPR011706	Multicopper oxidase, type 2
	superfamily	422	560	2.41E-80	251.025	cl19115	Cupredoxin superfamily	IPR017761	Laccase
	specific	39	155	1.32E-72	229.839	cd13849	CuRO_1_LCC_plant		
	superfamily	39	155	1.32E-72	229.839	cl19115	Cupredoxin superfamily		
	multi-dom	36	577	0	945.711	TIGR03389	laccase		
LAC02_5	specific	423	560	6.34E-82	255.262	cd13897	CuRO_3_LCC_plant	IPR001117	Multicopper oxidase, type 1
	superfamily	423	560	6.34E-82	255.262	cl19115	Cupredoxin superfamily	IPR011707	Multicopper oxidase, type 3
	specific	167	315	1.12E-77	244.43	cd13875	CuRO_2_LCC_plant	IPR011706	Multicopper oxidase, type 2
	superfamily	167	315	1.12E-77	244.43	cl19115	Cupredoxin superfamily	IPR017761	Laccase
	specific	36	152	7.67E-74	233.305	cd13849	CuRO_1_LCC_plant		
	superfamily	36	152	7.67E-74	233.305	cl19115	Cupredoxin superfamily		
	multi-dom	31	577	0	943.015	TIGR03389	laccase		
LAC02_6	specific	423	560	1.15E-81	254.491	cd13897	CuRO_3_LCC_plant	IPR001117	Multicopper oxidase, type 1
	superfamily	423	560	1.15E-81	254.491	cl19115	Cupredoxin superfamily	IPR011707	Multicopper oxidase, type 3
	specific	167	315	4.45E-79	247.897	cd13875	CuRO_2_LCC_plant	IPR011706	Multicopper oxidase, type 2
	superfamily	167	315	4.45E-79	247.897	cl19115	Cupredoxin superfamily	IPR017761	Laccase
	specific	36	152	3.32E-71	226.372	cd13849	CuRO_1_LCC_plant		
	superfamily	36	152	3.32E-71	226.372	cl19115	Cupredoxin superfamily		
	multi-dom	31	577	0	933.385	TIGR03389	laccase		
LAC03_1	specific	168	314	3.21E-90	277.172	cd13875	CuRO_2_LCC_plant	IPR001117	Multicopper oxidase, type 1
	superfamily	168	314	3.21E-90	277.172	cl19115	Cupredoxin superfamily	IPR011707	Multicopper oxidase, type 3
	specific	421	559	5.90E-81	252.565	cd13897	CuRO_3_LCC_plant	IPR011706	Multicopper oxidase, type 2
	superfamily	421	559	5.90E-81	252.565	cl19115	Cupredoxin superfamily	IPR017761	Laccase
	specific	37	153	4.07E-72	228.683	cd13849	CuRO_1_LCC_plant		
	superfamily	37	153	4.07E-72	228.683	cl19115	Cupredoxin superfamily		
	multi-dom	32	576	0	841.322	TIGR03389	laccase		
LAC03_2	specific	169	314	1.92E-86	267.157	cd13875	CuRO_2_LCC_plant	IPR001117	Multicopper oxidase, type 1
	superfamily	169	314	1.92E-86	267.157	cl19115	Cupredoxin superfamily	IPR011707	Multicopper oxidase, type 3
	specific	421	560	9.92E-82	254.491	cd13897	CuRO_3_LCC_plant	IPR011706	Multicopper oxidase, type 2
	superfamily	421	560	9.92E-82	254.491	cl19115	Cupredoxin superfamily	IPR017761	Laccase

	specific	37	153	1.06E-71	227.527	cd13849	CuRO_1_LCC_plant		
	superfamily	37	153	1.06E-71	227.527	cl19115	Cupredoxin superfamily		
	multi-dom	34	577	0	847.87	TIGR03389	laccase		
LAC04_1	specific	404	541	1.88E-80	250.639	cd13897	CuRO_3_LCC_plant	IPR001117	Multicopper oxidase, type 1
	superfamily	404	541	1.88E-80	250.639	cl19115	Cupredoxin superfamily	IPR011707	Multicopper oxidase, type 3
	specific	159	305	2.09E-73	232.874	cd13875	CuRO_2_LCC_plant	IPR011706	Multicopper oxidase, type 2
	superfamily	159	305	2.09E-73	232.874	cl19115	Cupredoxin superfamily	IPR017761	Laccase
	specific	28	144	3.83E-60	196.712	cd13849	CuRO_1_LCC_plant		
	superfamily	28	144	3.83E-60	196.712	cl19115	Cupredoxin superfamily		
	multi-dom	24	558	0	924.14	TIGR03389	laccase		
LAC04_2	specific	402	539	3.74E-81	252.565	cd13897	CuRO_3_LCC_plant	IPR001117	Multicopper oxidase, type 1
	superfamily	402	539	3.74E-81	252.565	cl19115	Cupredoxin superfamily	IPR011707	Multicopper oxidase, type 3
	specific	158	303	1.26E-80	251.364	cd13875	CuRO_2_LCC_plant	IPR011706	Multicopper oxidase, type 2
	superfamily	158	303	1.26E-80	251.364	cl19115	Cupredoxin superfamily	IPR017761	Laccase
	specific	26	142	1.34E-69	221.364	cd13849	CuRO_1_LCC_plant		
	superfamily	26	142	1.34E-69	221.364	cl19115	Cupredoxin superfamily		
	multi-dom	21	556	0	983.846	TIGR03389	laccase		
LAC04_3	specific	402	539	3.33E-82	255.262	cd13897	CuRO_3_LCC_plant	IPR001117	Multicopper oxidase, type 1
	superfamily	402	539	3.33E-82	255.262	cl19115	Cupredoxin superfamily	IPR011707	Multicopper oxidase, type 3
	specific	157	303	5.66E-79	247.126	cd13875	CuRO_2_LCC_plant	IPR011706	Multicopper oxidase, type 2
	superfamily	157	303	5.66E-79	247.126	cl19115	Cupredoxin superfamily	IPR017761	Laccase
	specific	26	142	9.90E-73	229.839	cd13849	CuRO_1_LCC_plant		
	superfamily	26	142	9.90E-73	229.839	cl19115	Cupredoxin superfamily		
	multi-dom	21	556	0	992.32	TIGR03389	laccase		
LAC04_4	specific	402	539	3.18E-80	250.254	cd13897	CuRO_3_LCC_plant	IPR001117	Multicopper oxidase, type 1
	superfamily	402	539	3.18E-80	250.254	cl19115	Cupredoxin superfamily	IPR011707	Multicopper oxidase, type 3
	specific	161	305	3.08E-77	242.504	cd13875	CuRO_2_LCC_plant	IPR011706	Multicopper oxidase, type 2
	superfamily	161	305	3.08E-77	242.504	cl19115	Cupredoxin superfamily	IPR017761	Laccase
	specific	28	144	6.02E-73	230.224	cd13849	CuRO_1_LCC_plant		
	superfamily	28	144	6.02E-73	230.224	cl19115	Cupredoxin superfamily		
	multi-dom	23	556	0	979.223	TIGR03389	laccase		
LAC04_5	specific	428	565	2.89E-76	240.624	cd13897	CuRO_3_LCC_plant	IPR001117	Multicopper oxidase, type 1
	superfamily	428	565	2.89E-76	240.624	cl19115	Cupredoxin superfamily	IPR011707	Multicopper oxidase, type 3
	specific	158	331	6.46E-69	221.318	cd13875	CuRO_2_LCC_plant	IPR011706	Multicopper oxidase, type 2
	superfamily	158	331	6.46E-69	221.318	cl19115	Cupredoxin superfamily	IPR017761	Laccase

	specific	26	142	2.75E-68	218.668	cd13849	CuRO_1_LCC_plant		
	superfamily	26	142	2.75E-68	218.668	cl19115	Cupredoxin superfamily		
	multi-dom	21	582	0	949.563	TIGR03389	laccase		
LAC04_6	specific	162	305	3.76E-80	250.208	cd13875	CuRO_2_LCC_plant	IPR001117	Multicopper oxidase, type 1
	superfamily	162	305	3.76E-80	250.208	cl19115	Cupredoxin superfamily	IPR011707	Multicopper oxidase, type 3
	specific	402	539	1.32E-78	246.017	cd13897	CuRO_3_LCC_plant	IPR011706	Multicopper oxidase, type 2
	superfamily	402	539	1.32E-78	246.017	cl19115	Cupredoxin superfamily	IPR017761	Laccase
	specific	29	145	1.70E-69	221.364	cd13849	CuRO_1_LCC_plant		
	superfamily	29	145	1.70E-69	221.364	cl19115	Cupredoxin superfamily		
	multi-dom	24	556	0	964.971	TIGR03389	laccase		
LAC04_8	specific	401	538	5.61E-79	246.787	cd13897	CuRO_3_LCC_plant	IPR001117	Multicopper oxidase, type 1
	superfamily	401	538	5.61E-79	246.787	cl19115	Cupredoxin superfamily	IPR011707	Multicopper oxidase, type 3
	specific	27	143	1.25E-68	219.053	cd13849	CuRO_1_LCC_plant	IPR011706	Multicopper oxidase, type 2
	superfamily	27	143	1.25E-68	219.053	cl19115	Cupredoxin superfamily	IPR017761	Laccase
	specific	159	304	7.72E-68	217.851	cd13875	CuRO_2_LCC_plant		
	superfamily	159	304	7.72E-68	217.851	cl19115	Cupredoxin superfamily		
	multi-dom	23	555	0	923.755	TIGR03389	laccase		
LAC05_3	specific	159	305	3.44E-89	274.09	cd13875	CuRO_2_LCC_plant	IPR001117	Multicopper oxidase, type 1
	superfamily	159	305	3.44E-89	274.09	cl19115	Cupredoxin superfamily	IPR011707	Multicopper oxidase, type 3
	specific	413	551	5.53E-85	262.966	cd13897	CuRO_3_LCC_plant	IPR011706	Multicopper oxidase, type 2
	superfamily	413	551	5.53E-85	262.966	cl19115	Cupredoxin superfamily	IPR017761	Laccase
	specific	27	143	4.29E-72	228.298	cd13849	CuRO_1_LCC_plant		
	superfamily	27	143	4.29E-72	228.298	cl19115	Cupredoxin superfamily		
	multi-dom	22	568	0	917.977	TIGR03389	laccase		
LAC05_4	specific	160	305	5.95E-87	268.312	cd13875	CuRO_2_LCC_plant	IPR001117	Multicopper oxidase, type 1
	superfamily	160	305	5.95E-87	268.312	cl19115	Cupredoxin superfamily	IPR011707	Multicopper oxidase, type 3
	specific	414	552	2.24E-85	263.736	cd13897	CuRO_3_LCC_plant	IPR011706	Multicopper oxidase, type 2
	superfamily	414	552	2.24E-85	263.736	cl19115	Cupredoxin superfamily	IPR017761	Laccase
	specific	27	143	1.92E-71	226.757	cd13849	CuRO_1_LCC_plant		
	superfamily	27	143	1.92E-71	226.757	cl19115	Cupredoxin superfamily		
	multi-dom	22	569	0	901.798	TIGR03389	laccase		
LAC05_5	specific	218	356	8.04E-87	261.425	cd13897	CuRO_3_LCC_plant	IPR001117	Multicopper oxidase, type 1
	superfamily	218	356	8.04E-87	261.425	cl19115	Cupredoxin superfamily	IPR011706	Multicopper oxidase, type 2
	specific	10	110	6.81E-59	189.732	cd13875	CuRO_2_LCC_plant		
	superfamily	10	110	6.81E-59	189.732	cl19115	Cupredoxin superfamily		

LAC05_6	specific	155	286	1.13E-76	235.185	cd13875	CuRO_2_LCC_plant	IPR001117	Multicopper oxidase, type 1
	superfamily	155	286	1.13E-76	235.185	cl19115	Cupredoxin superfamily	IPR011707	Multicopper oxidase, type 3
	specific	27	139	9.00E-67	208.653	cd13849	CuRO_1_LCC_plant		
	superfamily	27	139	9.00E-67	208.653	cl19115	Cupredoxin superfamily		
LAC06_1	specific	170	317	2.13E-77	243.66	cd13875	CuRO_2_LCC_plant	IPR001117	Multicopper oxidase, type 1
	superfamily	170	317	2.13E-77	243.66	cl19115	Cupredoxin superfamily	IPR011707	Multicopper oxidase, type 3
	specific	419	557	1.32E-74	236.002	cd13897	CuRO_3_LCC_plant	IPR011706	Multicopper oxidase, type 2
	superfamily	419	557	1.32E-74	236.002	cl19115	Cupredoxin superfamily	IPR017761	Laccase
	specific	39	155	5.30E-66	212.505	cd13849	CuRO_1_LCC_plant		
	superfamily	39	155	5.30E-66	212.505	cl19115	Cupredoxin superfamily		
	multi-dom	34	574	0	751.57	TIGR03389	laccase		
LAC06_2	specific	194	338	3.15E-74	235.956	cd13875	CuRO_2_LCC_plant	IPR001117	Multicopper oxidase, type 1
	superfamily	194	338	3.15E-74	235.956	cl19115	Cupredoxin superfamily	IPR011707	Multicopper oxidase, type 3
	specific	440	578	2.01E-72	230.609	cd13897	CuRO_3_LCC_plant	IPR011706	Multicopper oxidase, type 2
	superfamily	440	578	2.01E-72	230.609	cl19115	Cupredoxin superfamily	IPR017761	Laccase
	specific	37	153	8.36E-67	215.201	cd13849	CuRO_1_LCC_plant		
	superfamily	37	153	8.36E-67	215.201	cl19115	Cupredoxin superfamily		
	multi-dom	32	595	0	729.614	TIGR03389	laccase		
LAC06_3	specific	141	288	1.40E-75	238.267	cd13875	CuRO_2_LCC_plant	IPR001117	Multicopper oxidase, type 1
	superfamily	141	288	1.40E-75	238.267	cl19115	Cupredoxin superfamily	IPR011707	Multicopper oxidase, type 3
	specific	398	537	1.63E-73	232.535	cd13897	CuRO_3_LCC_plant	IPR011706	Multicopper oxidase, type 2
	superfamily	398	537	1.63E-73	232.535	cl19115	Cupredoxin superfamily	IPR017761	Laccase
	specific	14	126	2.75E-63	204.801	cd13849	CuRO_1_LCC_plant		
	superfamily	14	126	2.75E-63	204.801	cl19115	Cupredoxin superfamily		
	multi-dom	14	542	0	678.382	TIGR03389	laccase		
LAC07_2	specific	158	305	1.14E-86	267.542	cd13875	CuRO_2_LCC_plant	IPR001117	Multicopper oxidase, type 1
	superfamily	158	305	1.14E-86	267.542	cl19115	Cupredoxin superfamily	IPR011707	Multicopper oxidase, type 3
	specific	27	143	1.20E-69	221.749	cd13849	CuRO_1_LCC_plant	IPR011706	Multicopper oxidase, type 2
	superfamily	27	143	1.20E-69	221.749	cl19115	Cupredoxin superfamily	IPR017761	Laccase
	superfamily	408	550	1.21E-68	220.209	cl19115	Cupredoxin superfamily		
	multi-dom	22	567	0	771.216	TIGR03389	laccase		
LAC07_3	specific	423	564	1.06E-75	239.083	cd13897	CuRO_3_LCC_plant	IPR001117	Multicopper oxidase, type 1
	superfamily	423	564	1.06E-75	239.083	cl19115	Cupredoxin superfamily	IPR011707	Multicopper oxidase, type 3
	specific	170	317	2.92E-67	217.081	cd13875	CuRO_2_LCC_plant	IPR011706	Multicopper oxidase, type 2
	superfamily	170	317	2.92E-67	217.081	cl19115	Cupredoxin superfamily	IPR017761	Laccase

	specific	39	155	9.06E-63	204.03	cd13849	CuRO_1_LCC_plant		
	superfamily	39	155	9.06E-63	204.03	cl19115	Cupredoxin superfamily		
	multi-dom	34	581	0	695.331	TIGR03389	laccase		
LAC07_4	specific	159	310	6.14E-78	244.815	cd13875	CuRO_2_LCC_plant	IPR001117	Multicopper oxidase, type 1
	superfamily	159	310	6.14E-78	244.815	cl19115	Cupredoxin superfamily	IPR011707	Multicopper oxidase, type 3
	specific	415	556	1.82E-75	238.313	cd13897	CuRO_3_LCC_plant	IPR011706	Multicopper oxidase, type 2
	superfamily	415	556	1.82E-75	238.313	cl19115	Cupredoxin superfamily	IPR017761	Laccase
	specific	28	144	8.07E-64	206.727	cd13849	CuRO_1_LCC_plant		
	superfamily	28	144	8.07E-64	206.727	cl19115	Cupredoxin superfamily		
	multi-dom	23	556	0	702.65	TIGR03389	laccase		
LAC07_5	specific	159	306	1.29E-82	256.756	cd13875	CuRO_2_LCC_plant	IPR001117	Multicopper oxidase, type 1
	superfamily	159	306	1.29E-82	256.756	cl19115	Cupredoxin superfamily	IPR011707	Multicopper oxidase, type 3
	specific	28	144	2.25E-67	215.971	cd13849	CuRO_1_LCC_plant	IPR011706	Multicopper oxidase, type 2
	superfamily	28	144	2.25E-67	215.971	cl19115	Cupredoxin superfamily	IPR017761	Laccase
	superfamily	406	547	1.55E-68	219.823	cl19115	Cupredoxin superfamily		
	multi-dom	23	564	0	714.206	TIGR03389	laccase		
LAC11_1	specific	167	311	6.20E-87	267.927	cd13875	CuRO_2_LCC_plant	IPR001117	Multicopper oxidase, type 1
	superfamily	167	311	6.20E-87	267.927	cl19115	Cupredoxin superfamily	IPR011707	Multicopper oxidase, type 3
	specific	408	546	9.26E-86	264.892	cd13897	CuRO_3_LCC_plant	IPR011706	Multicopper oxidase, type 2
	superfamily	408	546	9.26E-86	264.892	cl19115	Cupredoxin superfamily	IPR017761	Laccase
	specific	34	150	1.07E-69	222.135	cd13849	CuRO_1_LCC_plant		
	superfamily	34	150	1.07E-69	222.135	cl19115	Cupredoxin superfamily		
	multi-dom	29	563	0	969.593	TIGR03389	laccase		
LAC11_2	specific	407	545	1.18E-85	264.507	cd13897	CuRO_3_LCC_plant	IPR001117	Multicopper oxidase, type 1
	superfamily	407	545	1.18E-85	264.507	cl19115	Cupredoxin superfamily	IPR011707	Multicopper oxidase, type 3
	specific	166	310	2.35E-85	264.075	cd13875	CuRO_2_LCC_plant	IPR011706	Multicopper oxidase, type 2
	superfamily	166	310	2.35E-85	264.075	cl19115	Cupredoxin superfamily	IPR017761	Laccase
	specific	33	149	8.87E-72	227.527	cd13849	CuRO_1_LCC_plant		
	superfamily	33	149	8.87E-72	227.527	cl19115	Cupredoxin superfamily		
	multi-dom	28	562	0	993.476	TIGR03389	laccase		
LAC11_3	specific	301	439	7.78E-86	261.81	cd13897	CuRO_3_LCC_plant	IPR001117	Multicopper oxidase, type 1
	superfamily	301	439	7.78E-86	261.81	cl19115	Cupredoxin superfamily	IPR011707	Multicopper oxidase, type 3
	specific	167	297	3.78E-82	252.519	cd13875	CuRO_2_LCC_plant	IPR011706	Multicopper oxidase, type 2
	superfamily	167	297	3.78E-82	252.519	cl19115	Cupredoxin superfamily	IPR017761	Laccase
	specific	34	150	2.19E-70	220.979	cd13849	CuRO_1_LCC_plant		

	superfamily	34	150	2.19E-70	220.979	cl19115	Cupredoxin superfamily		
	multi-dom	29	456	0	776.223	TIGR03389	laccase		
LAC11_4	specific	402	540	3.68E-84	260.269	cd13897	CuRO_3_LCC_plant	IPR001117	Multicopper oxidase, type 1
	superfamily	402	540	3.68E-84	260.269	cl19115	Cupredoxin superfamily	IPR011707	Multicopper oxidase, type 3
	specific	164	305	7.41E-82	254.83	cd13875	CuRO_2_LCC_plant	IPR011706	Multicopper oxidase, type 2
	superfamily	164	305	7.41E-82	254.83	cl19115	Cupredoxin superfamily	IPR017761	Laccase
	specific	34	147	2.14E-64	207.882	cd13849	CuRO_1_LCC_plant		
	superfamily	34	147	2.14E-64	207.882	cl19115	Cupredoxin superfamily		
	multi-dom	29	557	0	951.104	TIGR03389	laccase		
LAC11_6	specific	34	150	1.93E-70	217.127	cd13849	CuRO_1_LCC_plant	IPR011706	Multicopper oxidase, type 2
	superfamily	34	150	1.93E-70	217.127	cl19115	Cupredoxin superfamily	IPR011707	Multicopper oxidase, type 3
	superfamily	207	310	1.70E-66	207.497	cl19115	Cupredoxin superfamily		
	superfamily	167	226	2.55E-22	90.7355	cl19115	Cupredoxin superfamily		
	multi-dom	29	327	########	402.194	TIGR03389	laccase		
LAC12	specific	132	278	3.27E-82	249.438	cd13875	CuRO_2_LCC_plant	IPR001117	Multicopper oxidase, type 1
	superfamily	132	278	3.27E-82	249.438	cl19115	Cupredoxin superfamily	IPR011707	Multicopper oxidase, type 3
	specific	3	116	5.64E-67	209.038	cd13849	CuRO_1_LCC_plant		
	superfamily	3	116	5.64E-67	209.038	cl19115	Cupredoxin superfamily		
LAC14_1	specific	407	545	4.90E-81	252.565	cd13897	CuRO_3_LCC_plant	IPR001117	Multicopper oxidase, type 1
	superfamily	407	545	4.90E-81	252.565	cl19115	Cupredoxin superfamily	IPR011707	Multicopper oxidase, type 3
	specific	161	307	5.35E-73	231.719	cd13875	CuRO_2_LCC_plant	IPR011706	Multicopper oxidase, type 2
	superfamily	161	307	5.35E-73	231.719	cl19115	Cupredoxin superfamily	IPR017761	Laccase
	specific	30	146	3.26E-62	202.104	cd13849	CuRO_1_LCC_plant		
	superfamily	30	146	3.26E-62	202.104	cl19115	Cupredoxin superfamily		
	multi-dom	25	562	0	697.257	TIGR03389	laccase		
LAC14_5	specific	404	542	4.39E-76	239.469	cd13897	CuRO_3_LCC_plant	IPR001117	Multicopper oxidase, type 1
	superfamily	404	542	4.39E-76	239.469	cl19115	Cupredoxin superfamily	IPR011707	Multicopper oxidase, type 3
	specific	155	301	7.22E-68	218.237	cd13875	CuRO_2_LCC_plant	IPR011706	Multicopper oxidase, type 2
	superfamily	155	301	7.22E-68	218.237	cl19115	Cupredoxin superfamily	IPR017761	Laccase
	specific	24	140	2.36E-63	205.186	cd13849	CuRO_1_LCC_plant		
	superfamily	24	140	2.36E-63	205.186	cl19115	Cupredoxin superfamily		
	multi-dom	19	559	0	664.515	TIGR03389	laccase		
LAC14_6	specific	29	145	2.27E-67	216.357	cd13849	CuRO_1_LCC_plant	IPR001117	Multicopper oxidase, type 1
	superfamily	29	145	2.27E-67	216.357	cl19115	Cupredoxin superfamily	IPR011707	Multicopper oxidase, type 3
	specific	160	309	1.77E-64	209.762	cd13875	CuRO_2_LCC_plant	IPR011706	Multicopper oxidase, type 2

	superfamily	160	309	1.77E-64	209.762	cl19115	Cupredoxin superfamily	IPR017761	Laccase
	superfamily	409	549	1.66E-60	198.638	cl19115	Cupredoxin superfamily		
	multi-dom	24	566	0	655.656	TIGR03389	laccase		
LAC14_7	specific	412	550	1.18E-78	246.402	cd13897	CuRO_3_LCC_plant	IPR001117	Multicopper oxidase, type 1
	superfamily	412	550	1.18E-78	246.402	cl19115	Cupredoxin superfamily	IPR011707	Multicopper oxidase, type 3
	specific	32	148	3.78E-65	210.193	cd13849	CuRO_1_LCC_plant	IPR011706	Multicopper oxidase, type 2
	superfamily	32	148	3.78E-65	210.193	cl19115	Cupredoxin superfamily	IPR017761	Laccase
	specific	163	308	2.25E-64	209.377	cd13875	CuRO_2_LCC_plant		
	superfamily	163	308	2.25E-64	209.377	cl19115	Cupredoxin superfamily		
	multi-dom	27	568	0	660.278	TIGR03389	laccase		
LAC15	specific	164	311	2.96E-83	259.068	cd13875	CuRO_2_LCC_plant	IPR001117	Multicopper oxidase, type 1
	superfamily	164	311	2.96E-83	259.068	cl19115	Cupredoxin superfamily	IPR011707	Multicopper oxidase, type 3
	specific	422	561	2.01E-77	243.321	cd13897	CuRO_3_LCC_plant	IPR011706	Multicopper oxidase, type 2
	superfamily	422	561	2.01E-77	243.321	cl19115	Cupredoxin superfamily	IPR017761	Laccase
	specific	33	149	3.83E-64	207.497	cd13849	CuRO_1_LCC_plant		
	superfamily	33	149	3.83E-64	207.497	cl19115	Cupredoxin superfamily		
	multi-dom	33	578	0	803.572	TIGR03389	laccase		
LAC17_2	specific	422	559	9.83E-83	257.188	cd13897	CuRO_3_LCC_plant	IPR001117	Multicopper oxidase, type 1
	superfamily	422	559	9.83E-83	257.188	cl19115	Cupredoxin superfamily	IPR011707	Multicopper oxidase, type 3
	specific	165	313	5.13E-81	252.904	cd13875	CuRO_2_LCC_plant	IPR011706	Multicopper oxidase, type 2
	superfamily	165	313	5.13E-81	252.904	cl19115	Cupredoxin superfamily	IPR017761	Laccase
	specific	34	150	4.76E-71	225.987	cd13849	CuRO_1_LCC_plant		
	superfamily	34	150	4.76E-71	225.987	cl19115	Cupredoxin superfamily		
	multi-dom	29	576	0	960.734	TIGR03389	laccase		
LAC17_3	specific	422	559	2.99E-84	261.04	cd13897	CuRO_3_LCC_plant	IPR001117	Multicopper oxidase, type 1
	superfamily	422	559	2.99E-84	261.04	cl19115	Cupredoxin superfamily	IPR011707	Multicopper oxidase, type 3
	specific	165	313	1.72E-82	256.756	cd13875	CuRO_2_LCC_plant	IPR011706	Multicopper oxidase, type 2
	superfamily	165	313	1.72E-82	256.756	cl19115	Cupredoxin superfamily	IPR017761	Laccase
	specific	34	150	5.57E-71	225.601	cd13849	CuRO_1_LCC_plant		
	superfamily	34	150	5.57E-71	225.601	cl19115	Cupredoxin superfamily		
	multi-dom	29	576	0	963.815	TIGR03389	laccase		
LAC17_4	specific	425	562	2.21E-83	259.114	cd13897	CuRO_3_LCC_plant	IPR001117	Multicopper oxidase, type 1
	superfamily	425	562	2.21E-83	259.114	cl19115	Cupredoxin superfamily	IPR011707	Multicopper oxidase, type 3
	specific	165	313	4.80E-83	258.297	cd13875	CuRO_2_LCC_plant	IPR011706	Multicopper oxidase, type 2
	superfamily	165	313	4.80E-83	258.297	cl19115	Cupredoxin superfamily	IPR017761	Laccase

	specific	34	150	4.72E-70	223.29	cd13849	CuRO_1_LCC_plant		
	superfamily	34	150	4.72E-70	223.29	cl19115	Cupredoxin superfamily		
	multi-dom	29	579	0	957.652	TIGR03389	laccase		
LAC17_5	specific	171	319	7.31E-85	263.305	cd13875	CuRO_2_LCC_plant	IPR001117	Multicopper oxidase, type 1
	superfamily	171	319	7.31E-85	263.305	cl19115	Cupredoxin superfamily	IPR011707	Multicopper oxidase, type 3
	specific	429	566	2.48E-81	253.721	cd13897	CuRO_3_LCC_plant	IPR011706	Multicopper oxidase, type 2
	superfamily	429	566	2.48E-81	253.721	cl19115	Cupredoxin superfamily	IPR017761	Laccase
	specific	40	156	4.53E-74	234.076	cd13849	CuRO_1_LCC_plant		
	superfamily	40	156	4.53E-74	234.076	cl19115	Cupredoxin superfamily		
	multi-dom	35	583	0	962.275	TIGR03389	laccase		
LAC17_6	specific	171	319	1.77E-85	264.846	cd13875	CuRO_2_LCC_plant	IPR001117	Multicopper oxidase, type 1
	superfamily	171	319	1.77E-85	264.846	cl19115	Cupredoxin superfamily	IPR011707	Multicopper oxidase, type 3
	specific	429	566	4.95E-81	252.951	cd13897	CuRO_3_LCC_plant	IPR011706	Multicopper oxidase, type 2
	superfamily	429	566	4.95E-81	252.951	cl19115	Cupredoxin superfamily	IPR017761	Laccase
	specific	40	156	9.45E-76	238.313	cd13849	CuRO_1_LCC_plant		
	superfamily	40	156	9.45E-76	238.313	cl19115	Cupredoxin superfamily		
	multi-dom	35	583	0	952.645	TIGR03389	laccase		
LAC17_7	specific	163	311	3.86E-81	253.29	cd13875	CuRO_2_LCC_plant	IPR001117	Multicopper oxidase, type 1
	superfamily	163	311	3.86E-81	253.29	cl19115	Cupredoxin superfamily	IPR011707	Multicopper oxidase, type 3
	specific	419	556	9.46E-81	251.795	cd13897	CuRO_3_LCC_plant	IPR011706	Multicopper oxidase, type 2
	superfamily	419	556	9.46E-81	251.795	cl19115	Cupredoxin superfamily	IPR017761	Laccase
	specific	32	148	1.71E-66	213.66	cd13849	CuRO_1_LCC_plant		
	superfamily	32	148	1.71E-66	213.66	cl19115	Cupredoxin superfamily		
	multi-dom	27	573	0	923.369	TIGR03389	laccase		
LAC17_8	specific	415	552	1.35E-79	248.713	cd13897	CuRO_3_LCC_plant	IPR001117	Multicopper oxidase, type 1
	superfamily	415	552	1.35E-79	248.713	cl19115	Cupredoxin superfamily	IPR011707	Multicopper oxidase, type 3
	specific	163	305	2.13E-79	248.667	cd13875	CuRO_2_LCC_plant	IPR011706	Multicopper oxidase, type 2
	superfamily	163	305	2.13E-79	248.667	cl19115	Cupredoxin superfamily	IPR017761	Laccase
	specific	27	143	5.20E-65	209.808	cd13849	CuRO_1_LCC_plant		
	superfamily	27	143	5.20E-65	209.808	cl19115	Cupredoxin superfamily		
	multi-dom	22	569	0	897.176	TIGR03389	laccase		
LAC17_9	specific	377	514	1.20E-72	229.839	cd13897	CuRO_3_LCC_plant	IPR001117	Multicopper oxidase, type 1
	superfamily	377	514	1.20E-72	229.839	cl19115	Cupredoxin superfamily	IPR011707	Multicopper oxidase, type 3
	superfamily	36	142	3.25E-48	163.97	cl19115	Cupredoxin superfamily	IPR011706	Multicopper oxidase, type 2
	superfamily	157	265	4.38E-40	142.737	cl19115	Cupredoxin superfamily		

	multi-dom	31	529	0	715.747	TIGR03389	laccase		

Supplementary Table S4: Sub grouping of laccase genes from *Gossypium* species					
**Genes**	**subgroups of gene**	**G. arboreum**	**G.raimondii**	**G.hirsutum (A^T)**	**G.hirsutum (D^T)**
LAC02	*LAC02_1*	Cotton_A_20043	Gorai.013G263200	Gh_A13G1977	Gh_D13G2373
	LAC02_2	Cotton_A_30643	**NP**	Gh_A03G2084	**NP**
	LAC02_3	Cotton_A_30645	**NP**	**NP**	**NP**
	LAC02_4	Cotton_A_30646	**NP**	Gh_A03G2082	**NP**
	LAC02_5	**NP**	Gorai.003G129800	**NP**	Gh_D03G1181
	LAC02_6	**NP**	Gorai.003G129700	**NP**	Gh_D03G1180
LAC03	*LAC03_1*	Cotton_A_04178	Gorai.003G150200	Gh_A03G2057	Gh_D03G1367
	LAC03_2	Cotton_A_24290	Gorai.004G234200	Gh_A08G2350	Gh_D08G2159
LAC04	*LAC04_1*	Cotton_A_00335	Gorai.002G261500	Gh_A01G1948	Gh_D01G2209
	LAC04_2	Cotton_A_05572	Gorai.007G378200	Gh_A11G2936	Gh_D11G3322
	LAC04_3	Cotton_A_06597	Gorai.012G111900	**NP**	Gh_D04G1243
	LAC04_4	Cotton_A_12917	Gorai.009G093800	Gh_A05G0758	Gh_D05G0888
	LAC04_5	Cotton_A_13553	Gorai.011G279600	**NP**	Gh_D10G2466
	LAC04_6	Cotton_A_20282	Gorai.003G124600	Gh_A03G0417	Gh_D03G1128
	LAC04_7	Cotton_A_29171	**NP**	**NP**	**NP**
	LAC04_8	Cotton_A_32213	Gorai.009G321900	Gh_A05G2622	Gh_D05G2912
LAC05	*LAC05_1*	*Cotton_A_13817*	**NP**	**NP**	**NP**
	LAC05_2	*Cotton_A_13818*	**NP**	*Gh_A06G1413*	**NP**
	LAC05_3	*Cotton_A_13820*	*Gorai.010G194600*	*Gh_A06G1415*	*Gh_D06G1762*
	LAC05_4	*Cotton_A_15837*	*Gorai.011G290000*	*Gh_A10G2140*	*Gh_D10G2461*
	LAC05_5	**NP**	*Gorai.010G194200*	**NP**	*Gh_D06G2354*
	LAC05_6	**NP**	*Gorai.010G194300*	**NP**	*Gh_D06G1759*
LAC06	*LAC06_1*	Cotton_A_14417	Gorai.007G110500	Gh_A11G0894	Gh_D11G1042
	LAC06_2	Cotton_A_25874	Gorai.008G126500	Gh_A12G1019	Gh_D12G1138
	LAC06_3	NP	Gorai.007G205400	Gh_A11G1716	Gh_D11G1874
LAC07	*LAC07_1*	Cotton_A_22687	**NP**	Gh_A04G0743	**NP**
	LAC07_2	Cotton_A_30033	Gorai.012G110000	Gh_A04G0744	Gh_D04G1224
	LAC07_3	Cotton_A_30034	Gorai.012G109900	**NP**	Gh_D04G1223
	LAC07_4	Cotton_A_30035	Gorai.012G109700	**NP**	Gh_D04G1221
	LAC07_5	Cotton_A_35771	Gorai.002G148200	Gh_A13G2215	Gh_D13G2524
LAC09	*LAC09*	**NP**	Gorai.012G109500	**NP**	**NP**
LAC11	*LAC11_1*	Cotton_A_00882	Gorai.013G027600	Gh_A13G0237	Gh_D13G0253
	LAC11_2	Cotton_A_17036	Gorai.011G101300	Gh_A10G0858	Gh_D10G0895
	LAC11_3	Cotton_A_19723	Gorai.013G036200	Gh_A13G2102	Gh_D13G2551
	LAC11_4	Cotton_A_26217	Gorai.003G096600	Gh_A03G0583	Gh_D03G0865

	LAC11_5	Cotton_A_26221	**NP**	**NP**	**NP**
	LAC11_6	**NP**	Gorai.013G036000	**NP**	Gh_D13G0328
	LAC11_7	**NP**	**NP**	Gh_A13G2103	**NP**
LAC12	*LAC12*	Cotton_A_31477	**NP**	Gh_A05G2631	Gh_D05G2921
LAC14	*LAC14_1*	Cotton_A_04514	Gorai.009G260600	Gh_A05G2100	Gh_D05G2354
	LAC14_2	Cotton_A_04517	Gorai.009G260800	Gh_A05G2117	**NP**
	LAC14_3	Cotton_A_04519	Gorai.009G261000	Gh_A05G2116	**NP**
	LAC14_4	Cotton_A_04522	Gorai.009G261500	Gh_A05G2113	**NP**
	LAC14_5	Cotton_A_04526	Gorai.009G261300	**NP**	Gh_D05G2356
	LAC14_6	Cotton_A_10403	Gorai.005G076600	Gh_A02G0638	Gh_D02G0685
	LAC14_7	Cotton_A_37880	Gorai.010G130100	Gh_A06G0997	Gh_D06G1188
	LAC14_8	**NP**	Gorai.009G261100	Gh_A05G2115	**NP**
LAC15	*LAC15*	Cotton_A_04513	Gorai.009G260400	Gh_A05G2099	Gh_D05G2353
LAC17	*LAC17_1*	Cotton_A_00902	**NP**	Gh_A13G0218	**NP**
	LAC17_2	Cotton_A_00905	Gorai.013G025500	Gh_A13G0216	Gh_D13G0231
	LAC17_3	Cotton_A_00947	Gorai.013G021400	Gh_A13G0179	Gh_D13G0194
	LAC17_4	Cotton_A_07013	Gorai.009G103200	Gh_A05G0849	Gh_D05G3888
	LAC17_5	Cotton_A_12054	Gorai.006G171500	Gh_A09G1445	Gh_D09G1454
	LAC17_6	**NP**	Gorai.007G376800	Gh_A11G2922	Gh_D11G3307
	LAC17_7	**NP**	Gorai.002G257100	Gh_A01G1905	Gh_D01G2166
	LAC17_8	**NP**	Gorai.007G376600	Gh_A11G2920	Gh_D11G3305
	LAC17_9	**NP**	Gorai.009G103600	Gh_A05G0853	Gh_Sca005020G01
	TOTAL	**44 GENES**	**46 GENES**	**42 GENES**	**42 GENES**

NP - Not present

Supplementary Table S5: Sub-cellular localization analysis details of identified cotton laccase proteins.

Species	Laccase ID	Name	cTP	mTP	SP	other	Loc	RC
G. arboreum	LAC02_1	Cotton_A_20043	0.411	0.071	0.145	0.226	C	5
	LAC02_2	Cotton_A_30643	0.224	0.137	0.061	0.333	_	5
	LAC02_3	Cotton_A_30645	0.222	0.077	0.233	0.169	S	5
	LAC02_4	Cotton_A_30646	0.227	0.129	0.093	0.264	_	5
	LAC03_1	Cotton_A_04178	0.283	0.097	0.096	0.139	C	5
	LAC03_2	Cotton_A_24290	0.152	0.026	0.623	0.024	S	3
	LAC04_1	Cotton_A_00335	0.678	0.126	0.035	0.169	C	3
	LAC04_2	Cotton_A_05572	0.525	0.136	0.013	0.411	C	5
	LAC04_3	Cotton_A_06597	0.68	0.113	0.021	0.359	C	4
	LAC04_4	Cotton_A_12917	0.428	0.15	0.034	0.452	_	5
	LAC04_5	Cotton_A_13553	0.332	0.231	0.024	0.536	_	4
	LAC04_6	Cotton_A_20282	0.197	0.222	0.066	0.465	_	4
	LAC04_7	Cotton_A_29171	0.182	0.098	0.029	0.406	_	4
	LAC04_8	Cotton_A_32213	0.431	0.141	0.017	0.516	_	5
	LAC05_1	Cotton_A_13817	0.606	0.115	0.03	0.242	C	4
	LAC05_2	Cotton_A_13818	0.579	0.131	0.034	0.253	C	4
	LAC05_3	Cotton_A_13820	0.302	0.05	0.243	0.127	C	5
	LAC05_4	Cotton_A_15837	0.315	0.04	0.489	0.07	S	5
	LAC06_1	Cotton_A_14417	0.577	0.093	0.138	0.165	C	3
	LAC06_2	Cotton_A_25874	0.204	0.084	0.013	0.732	_	3
	LAC07_1	Cotton_A_22687	0.363	0.176	0.033	0.448	_	5
	LAC07_2	Cotton_A_30033	0.168	0.146	0.038	0.572	_	3
	LAC07_3	Cotton_A_30034	0.241	0.126	0.033	0.623	_	4
	LAC07_4	Cotton_A_30035	0.219	0.146	0.022	0.623	_	3
	LAC07_5	Cotton_A_35771	0.322	0.097	0.037	0.464	_	5
	LAC11_1	Cotton_A_00882	0.533	0.194	0.022	0.29	C	4
	LAC11_2	Cotton_A_17036	0.375	0.208	0.018	0.293	C	5
	LAC11_3	Cotton_A_19723	0.275	0.162	0.057	0.269	C	5
	LAC11_4	Cotton_A_26217	0.403	0.06	0.162	0.196	C	4

	LAC11_5	Cotton_A_26221	0.314	0.139	0.039	0.507	_	5
	LAC12	Cotton_A_31477	0.214	0.035	0.477	0.102	S	4
	LAC14_1	Cotton_A_04514	0.279	0.142	0.053	0.31	_	5
	LAC14_2	Cotton_A_04517	0.197	0.251	0.017	0.584	_	4
	LAC14_3	Cotton_A_04519	0.195	0.057	0.03	0.758	_	3
	LAC14_4	Cotton_A_04522	0.499	0.107	0.014	0.565	_	5
	LAC14_5	Cotton_A_04526	0.477	0.103	0.014	0.598	_	5
	LAC14_6	Cotton_A_10403	0.463	0.086	0.08	0.268	C	5
	LAC14_7	Cotton_A_37880	0.166	0.209	0.027	0.624	_	3
	LAC15	Cotton_A_04513	0.207	0.117	0.024	0.719	_	3
	LAC17_1	Cotton_A_00902	0.561	0.107	0.03	0.254	C	4
	LAC17_2	Cotton_A_00905	0.503	0.124	0.023	0.251	C	4
	LAC17_3	Cotton_A_00947	0.401	0.079	0.07	0.192	C	4
	LAC17_4	Cotton_A_07013	0.348	0.026	0.166	0.122	C	5
	LAC17_5	Cotton_A_12054	0.319	0.068	0.096	0.318	C	5
	LAC02_1	Gr013G263200	0.011	0.031	0.907	0.091	S	1
	LAC02_5	Gr003G129800	0.022	0.018	0.911	0.132	S	2
	LAC02_6	Gr003G129700	0.041	0.024	0.747	0.32	S	3
	LAC03_1	Gr003G150200	0.002	0.039	0.966	0.373	S	3
	LAC03_2	Gr004G234200	0.003	0.039	0.983	0.113	S	1
	LAC04_1	Gr002G261500	0.002	0.041	0.98	0.24	S	2
	LAC04_2	Gr007G378200	0.003	0.054	0.982	0.061	S	1
	LAC04_3	Gr012G111900	0.005	0.072	0.962	0.064	S	1
	LAC04_4	Gr009G093800	0.005	0.34	0.89	0.006	S	3
	LAC04_5	Gr011G279600	0.01	0.111	0.911	0.037	S	1
	LAC04_6	Gr003G124600	0.003	0.125	0.946	0.048	S	1
	LAC04_8	Gr009G321900	0.019	0.053	0.949	0.022	S	1
	LAC05_3	Gr010G194600	0.013	0.022	0.982	0.021	S	1
	LAC05_4	Gr011G290000	0.009	0.023	0.968	0.119	S	1
	LAC05_5	Gr010G194200	0.059	0.277	0.122	0.631	_	4
	LAC05_6	Gr010G194300	0.012	0.027	0.979	0.018	S	1

G. raimondii	LAC06_1	Gr007G110500	0.011	0.053	0.956	0.063	S	1
	LAC06_2	Gr008G126500	0.007	0.067	0.984	0.027	S	1
	LAC06_3	Gr007G205400	0.041	0.539	0.053	0.575	_	5
	LAC07_2	Gr012G110000	0.012	0.055	0.929	0.03	S	1
	LAC07_3	Gr012G109900	0.055	0.012	0.872	0.037	S	1
	LAC07_4	Gr012G109700	0.014	0.102	0.968	0.006	S	1
	LAC07_5	Gr002G148200	0.023	0.06	0.93	0.025	S	1
	LAC09	Gr012G109500	0.014	0.089	0.967	0.005	S	1
	LAC11_1	Gr013G027600	0.113	0.096	0.035	0.86	_	2
	LAC11_2	Gr011G101300	0.003	0.044	0.949	0.164	S	2
	LAC11_3	Gr013G036200	0.001	0.059	0.992	0.049	S	1
	LAC11_4	Gr003G096600	0.005	0.06	0.963	0.036	S	1
	LAC11_6	Gr013G036000	0.001	0.059	0.992	0.049	S	1
	LAC14_1	Gr009G260600	0.012	0.215	0.783	0.105	S	3
	LAC14_2	Gr009G260800	0.006	0.037	0.974	0.083	S	1
	LAC14_3	Gr009G261000	0.002	0.342	0.959	0.018	S	2
	LAC14_4	Gr009G261500	0.016	0.039	0.967	0.074	S	1
	LAC14_5	Gr009G261300	0.014	0.036	0.976	0.068	S	1
	LAC14_6	Gr005G076600	0.003	0.018	0.993	0.117	S	1
	LAC14_7	Gr010G130100	0.003	0.052	0.989	0.032	S	1
	LAC14_8	Gr009G261100	0.002	0.124	0.983	0.023	S	1
	LAC15	Gr009G260400	0.001	0.045	0.996	0.036	S	1
	LAC17_2	Gr013G025500	0.005	0.04	0.97	0.038	S	1
	LAC17_3	Gr013G021400	0.007	0.029	0.965	0.037	S	1
	LAC17_4	Gr009G103200	0.013	0.021	0.948	0.047	S	1
	LAC17_5	Gr006G171500	0.002	0.035	0.987	0.083	S	1
	LAC17_6	Gr007G376800	0.004	0.025	0.978	0.037	S	1
	LAC17_7	Gr002G257100	0.018	0.019	0.918	0.106	S	1
	LAC17_8	Gr007G376600	0.013	0.038	0.968	0.037	S	1
	LAC17_9	Gr009G103600	0.004	0.043	0.964	0.031	S	1
	LAC02_1	Gh_A13G1977	0.01	0.019	0.958	0.052	S	1

G. hirsutum A- subgenome	LAC02_2	Gh_A03G2084	0.029	0.015	0.841	0.217	S	2
	LAC02_4	Gh_A03G2082	0.047	0.013	0.801	0.176	S	2
	LAC03_1	Gh_A03G2057	0.002	0.034	0.963	0.349	S	2
	LAC03_2	Gh_A08G2350	0.004	0.031	0.984	0.124	S	1
	LAC04_1	Gh_A01G1948	0.002	0.041	0.98	0.24	S	2
	LAC04_2	Gh_A11G2936	0.002	0.092	0.974	0.035	S	1
	LAC04_4	Gh_A05G0758	0.009	0.126	0.941	0.013	S	1
	LAC04_6	Gh_A03G0417	0.003	0.125	0.946	0.048	S	1
	LAC04_8	Gh_A05G2622	0.039	0.059	0.888	0.029	S	1
	LAC05_2	Gh_A06G1413	0.007	0.034	0.983	0.016	S	1
	LAC05_3	Gh_A06G1415	0.011	0.026	0.98	0.021	S	1
	LAC05_4	Gh_A10G2140	0.009	0.021	0.965	0.142	S	1
	LAC06_1	Gh_A11G0894	0.042	0.13	0.678	0.155	S	3
	LAC06_2	Gh_A12G1019	0.01	0.062	0.983	0.029	S	1
	LAC06_3	Gh_A11G1716	0.173	0.07	0.105	0.9	_	2
	LAC07_1	Gh_A04G0743	0.009	0.233	0.957	0.002	S	2
	LAC07_2	Gh_A04G0744	0.013	0.049	0.932	0.037	S	1
	LAC07_5	Gh_A13G2215	0.01	0.031	0.978	0.025	S	1
	LAC11_1	Gh_A13G0237	0.003	0.039	0.984	0.048	S	1
	LAC11_2	Gh_A10G0858	0.003	0.044	0.949	0.164	S	2
	LAC11_3	Gh_A13G2102	0.002	0.164	0.947	0.051	S	2
	LAC11_4	Gh_A03G0583	0.005	0.059	0.947	0.039	S	1
	LAC11_7	Gh_A13G2103	0.002	0.182	0.945	0.048	S	2
	LAC12	Gh_A05G2631	0.211	0.322	0.04	0.462	_	5
	LAC14_1	Gh_A05G2100	0.003	0.05	0.985	0.097	S	1
	LAC14_2	Gh_A05G2117	0.004	0.125	0.949	0.044	S	1
	LAC14_3	Gh_A05G2116	0.002	0.083	0.99	0.028	S	1
	LAC14_4	Gh_A05G2113	0.013	0.04	0.968	0.073	S	1
	LAC14_6	Gh_A02G0638	0.003	0.024	0.99	0.118	S	1
	LAC14_7	Gh_A06G0997	0.003	0.053	0.988	0.03	S	1
	LAC14_8	Gh_A05G2115	0.002	0.115	0.983	0.027	S	1
	LAC15	Gh_A05G2099	0.001	0.109	0.991	0.027	S	1
	LAC17_1	Gh_A13G0218	0.006	0.027	0.971	0.037	S	1

	LAC17_2	Gh_A13G0216	0.007	0.03	0.97	0.032	S	1
	LAC17_3	Gh_A13G0179	0.004	0.031	0.975	0.066	S	1
	LAC17_4	Gh_A05G0849	0.016	0.022	0.94	0.036	S	1
	LAC17_5	Gh_A09G1445	0.002	0.039	0.987	0.078	S	1
	LAC17_6	Gh_A11G2922	0.005	0.026	0.968	0.042	S	1
	LAC17_7	Gh_A01G1905	0.019	0.015	0.961	0.045	S	1
	LAC17_8	Gh_A11G2920	0.013	0.04	0.967	0.036	S	1
	LAC17_9	Gh_A05G0853	0.003	0.054	0.959	0.041	S	1
tum D- subgenome	LAC02_1	Gh_D13G2373	0.014	0.029	0.898	0.08	S	1
	LAC02_5	Gh_D03G1181	0.024	0.016	0.906	0.137	S	2
	LAC02_6	Gh_D03G1180	0.034	0.025	0.755	0.342	S	3
	LAC03_1	Gh_D03G1367	0.002	0.039	0.966	0.373	S	3
	LAC03_2	Gh_D08G2159	0.004	0.031	0.984	0.124	S	1
	LAC04_1	Gh_D01G2209	0.002	0.041	0.98	0.24	S	2
	LAC04_2	Gh_D11G3322	0.003	0.054	0.982	0.061	S	1
	LAC04_3	Gh_D04G1243	0.005	0.071	0.966	0.067	S	1
	LAC04_4	Gh_D05G0888	0.003	0.451	0.911	0.007	S	3
	LAC04_5	Gh_D10G2466	0.008	0.103	0.929	0.038	S	1
	LAC04_6	Gh_D03G1128	0.003	0.123	0.945	0.049	S	1
	LAC04_8	Gh_D05G2912	0.019	0.053	0.949	0.022	S	1
	LAC05_3	Gh_D06G1762	0.013	0.022	0.982	0.021	S	1
	LAC05_4	Gh_D10G2461	0.006	0.02	0.975	0.121	S	1
	LAC05_5	Gh_D06G2354	0.027	0.073	0.722	0.397	S	4
	LAC05_6	Gh_D06G1759	0.005	0.028	0.985	0.031	S	1
	LAC06_1	Gh_D11G1042	0.011	0.053	0.956	0.063	S	1
	LAC06_2	Gh_D12G1138	0.007	0.067	0.984	0.027	S	1
	LAC06_3	Gh_D11G1874	0.12	0.217	0.146	0.601	_	4
	LAC07_2	Gh_D04G1224	0.012	0.055	0.929	0.03	S	1
	LAC07_3	Gh_D04G1223	0.055	0.012	0.872	0.037	S	1
	LAC07_4	Gh_D04G1221	0.014	0.102	0.968	0.006	S	1
	LAC07_5	Gh_D13G2524	0.021	0.052	0.933	0.032	S	1

G. hirsu	**LAC11_1**	Gh_D13G0253	0.003	0.039	0.984	0.048	S	1
	**LAC11_2**	Gh_D10G0895	0.003	0.044	0.949	0.164	S	2
	**LAC11_3**	Gh_D13G2551	0.002	0.064	0.983	0.071	S	1
	**LAC11_4**	Gh_D03G0865	0.004	0.054	0.959	0.044	S	1
	**LAC11_6**	Gh_D13G0328	0.001	0.059	0.992	0.049	S	1
	LAC12	Gh_D05G2921	0.234	0.38	0.043	0.338	M	5
	**LAC14_1**	Gh_D05G2354	0.003	0.057	0.985	0.08	S	1
	**LAC14_5**	Gh_D05G2356	0.015	0.037	0.971	0.071	S	1
	**LAC14_6**	Gh_D02G0685	0.003	0.018	0.994	0.12	S	1
	**LAC14_7**	Gh_D06G1188	0.003	0.052	0.989	0.032	S	1
	LAC15	Gh_D05G2353	0.001	0.045	0.996	0.038	S	1
	LAC17_2	Gh_D13G0231	0.006	0.034	0.968	0.04	S	1
	LAC17_3	Gh_D13G0194	0.007	0.031	0.961	0.039	S	1
	LAC17_4	Gh_D05G3888	0.013	0.021	0.948	0.047	S	1
	LAC17_5	Gh_D09G1454	0.002	0.033	0.988	0.073	S	1
	LAC17_6	Gh_D11G3307	0.004	0.025	0.978	0.037	S	1
	LAC17_7	Gh_D01G2166	0.018	0.019	0.918	0.106	S	1
	LAC17_8	Gh_D11G3305	0.013	0.04	0.968	0.036	S	1
	LAC17_9	Gh_Sca00502	0.002	0.062	0.952	0.068	S	1

Supplementary Table S6: Computational prediction of miRNA target sites in the *G. arboreum* Laccace gene family members.

Gene name	Target_Acc.	miRNA_Acc.	Expecta--tion	UPE	miRNA_start	miRNA_end	Target_start	Target_end	miRNA_aligned_fragment	Target_aligned_fragment	Inhibition	Multiplicity
GaLAC02_1	Cotton_A_20043	ath-miR397a	0.5	16.44	1	20	577	596	UCAUUGAGUGCAGCGUUGAU	AUCAAUGCUGCACUCAAUGA	Cleavage	1
		ath-miR397b	1.5	16.44	1	20	577	596	UCAUUGAGUGCAUCGUUGAU	AUCAAUGCUGCACUCAAUGA	Cleavage	1
		osa-miR397a	0.5	16.44	1	20	577	596	UCAUUGAGUGCAGCGUUGAU	AUCAAUGCUGCACUCAAUGA	Cleavage	1
		osa-miR397b	1	16.44	1	20	577	596	UUAUUGAGUGCAGCGUUGAU	AUCAAUGCUGCACUCAAUGA	Cleavage	1
		ptc-miR397a	0.5	16.44	1	20	577	596	UCAUUGAGUGCAGCGUUGAU	AUCAAUGCUGCACUCAAUGA	Cleavage	1
		ptc-miR397b	1.5	16.44	1	20	577	596	CCAUUGAGUGCAGCGUUGAU	AUCAAUGCUGCACUCAAUGA	Cleavage	1
		bna-miR397a	0.5	16.44	1	20	577	596	UCAUUGAGUGCAGCGUUGAU	AUCAAUGCUGCACUCAAUGA	Cleavage	1
		bna-miR397b	0.5	16.44	1	20	577	596	UCAUUGAGUGCAGCGUUGAU	AUCAAUGCUGCACUCAAUGA	Cleavage	1
		vvi-miR397a	0.5	16.44	1	20	577	596	UCAUUGAGUGCAGCGUUGAU	AUCAAUGCUGCACUCAAUGA	Cleavage	1
		sly-miR397	1.5	16.163	1	20	575	594	AUUGAGUGCAGCGUUGAUGA	UAAUCAAUGCUGCACUCAAU	Cleavage	1
		sbi-miR397-5p	0.5	16.44	1	20	577	596	UCAUUGAGUGCAGCGUUGAU	AUCAAUGCUGCACUCAAUGA	Cleavage	1
		bdi-miR397a	0.5	16.44	1	20	577	596	UCAUUGAGUGCAGCGUUGAU	AUCAAUGCUGCACUCAAUGA	Cleavage	1
		zma-miR397a-5p	1.5	16.44	1	20	577	596	UCAUUGAGCGCAGCGUUGAU	AUCAAUGCUGCACUCAAUGA	Translation	1
		zma-miR397b-5p	1.5	16.44	1	20	577	596	UCAUUGAGCGCAGCGUUGAU	AUCAAUGCUGCACUCAAUGA	Translation	1
		rco-miR397	0.5	16.44	1	20	577	596	UCAUUGAGUGCAGCGUUGAU	AUCAAUGCUGCACUCAAUGA	Cleavage	1
		aly-miR397a-5p	0.5	16.44	1	20	577	596	UCAUUGAGUGCAGCGUUGAU	AUCAAUGCUGCACUCAAUGA	Cleavage	1
		aly-miR397b-5p	0.5	16.44	1	20	577	596	UCAUUGAGUGCAGCGUUGAU	AUCAAUGCUGCACUCAAUGA	Cleavage	1
		pab-miR397	1.5	16.44	1	20	577	596	UCAUUGAGUGCAGCGUUGAC	AUCAAUGCUGCACUCAAUGA	Cleavage	1

		hvu-miR397	2	16.44	1	20	577	596	CCGUUGAGUGCAGCGUUGAU	AUCAAUGCUGCACUCAAUGA	Cleavage	1
		csi-miR397	0.5	16.44	1	20	577	596	UCAUUGAGUGCAGCGUUGAU	AUCAAUGCUGCACUCAAUGA	Cleavage	1
		tcc-miR397	0.5	16.44	1	20	577	596	UCAUUGAGUGCAGCGUUGAU	AUCAAUGCUGCACUCAAUGA	Cleavage	1
		gma-miR397a	0.5	16.44	1	20	577	596	UCAUUGAGUGCAGCGUUGAU	AUCAAUGCUGCACUCAAUGA	Cleavage	1
		gma-miR397b-5p	0.5	16.44	1	20	577	596	UCAUUGAGUGCAGCGUUGAU	AUCAAUGCUGCACUCAAUGA	Cleavage	1
		ssl-miR397	0.5	16.44	1	20	577	596	UCAUUGAGUGCAGCGUUGAU	AUCAAUGCUGCACUCAAUGA	Cleavage	1
		dpr-miR397	1.5	16.44	1	20	577	596	CCAUUGAGUGCAGCGUUGAU	AUCAAUGCUGCACUCAAUGA	Cleavage	1
		mdm-miR397a	1.5	16.092	1	20	574	593	UUGAGUGCAGCGUUGAUGAA	UUAAUCAAUGCUGCACUCAA	Cleavage	1
		mdm-miR397b	1.5	16.092	1	20	574	593	UUGAGUGCAGCGUUGAUGAA	UUAAUCAAUGCUGCACUCAA	Cleavage	1
		cme-miR397	0.5	16.44	1	20	577	596	UCAUUGAGUGCAGCGUUGAU	AUCAAUGCUGCACUCAAUGA	Cleavage	1
		lja-miR397	2	16.22	1	21	575	595	UAUUGAGUGCAGCGUUGAUGA	UAAUCAAUGCUGCACUCAAUG	Cleavage	1
		mtr-miR397-5p	0.5	16.44	1	20	577	596	UCAUUGAGUGCAGCGUUGAU	AUCAAUGCUGCACUCAAUGA	Cleavage	1
GaLAC02_1	Cotton_A_20043	bdi-miR397b-5p	1.5	16.163	1	21	574	594	AUUGAGUGCAGCGUUGAUGAA	UUAAUCAAUGCUGCACUCAAU	Cleavage	1
		stu-miR397-5p	1.5	16.163	1	20	575	594	AUUGAGUGCAGCGUUGAUGA	UAAUCAAUGCUGCACUCAAU	Cleavage	1
		ath-miR397a	1	21.348	1	20	694	713	UCAUUGAGUGCAGCGUUGAU	AUCAAUGCUGCGCUCAAUGA	Cleavage	1
		ath-miR397b	2	21.348	1	20	694	713	UCAUUGAGUGCAUCGUUGAU	AUCAAUGCUGCGCUCAAUGA	Cleavage	1
		osa-miR397a	1	21.348	1	20	694	713	UCAUUGAGUGCAGCGUUGAU	AUCAAUGCUGCGCUCAAUGA	Cleavage	1
		osa-miR397b	1.5	21.348	1	20	694	713	UUAUUGAGUGCAGCGUUGAU	AUCAAUGCUGCGCUCAAUGA	Cleavage	1
		ptc-miR397a	1	21.348	1	20	694	713	UCAUUGAGUGCAGCGUUGAU	AUCAAUGCUGCGCUCAAUGA	Cleavage	1
		ptc-miR397b	2	21.348	1	20	694	713	CCAUUGAGUGCAGCGUUGAU	AUCAAUGCUGCGCUCAAUGA	Cleavage	1

GaLAC02_2	Cotton_A_30643	bna-miR397a	1	21.348	1	20	694	713	UCAUUGAGUGCAGCGUUGAU	AUCAAUGCUGCGCUCAAUGA	Cleavage	1
		bna-miR397b	1	21.348	1	20	694	713	UCAUUGAGUGCAGCGUUGAU	AUCAAUGCUGCGCUCAAUGA	Cleavage	1
		vvi-miR397a	1	21.348	1	20	694	713	UCAUUGAGUGCAGCGUUGAU	AUCAAUGCUGCGCUCAAUGA	Cleavage	1
		sly-miR397	2	19.098	1	20	692	711	AUUGAGUGCAGCGUUGAUGA	UGAUCAAUGCUGCGCUCAAU	Cleavage	1
		sbi-miR397-5p	1	21.348	1	20	694	713	UCAUUGAGUGCAGCGUUGAU	AUCAAUGCUGCGCUCAAUGA	Cleavage	1
		bdi-miR397a	1	21.348	1	20	694	713	UCAUUGAGUGCAGCGUUGAU	AUCAAUGCUGCGCUCAAUGA	Cleavage	1
		zma-miR397a-5p	0.5	21.348	1	20	694	713	UCAUUGAGCGCAGCGUUGAU	AUCAAUGCUGCGCUCAAUGA	Cleavage	1
		zma-miR397b-5p	0.5	21.348	1	20	694	713	UCAUUGAGCGCAGCGUUGAU	AUCAAUGCUGCGCUCAAUGA	Cleavage	1
		rco-miR397	1	21.348	1	20	694	713	UCAUUGAGUGCAGCGUUGAU	AUCAAUGCUGCGCUCAAUGA	Cleavage	1
		aly-miR397a-5p	1	21.348	1	20	694	713	UCAUUGAGUGCAGCGUUGAU	AUCAAUGCUGCGCUCAAUGA	Cleavage	1
		aly-miR397b-5p	1	21.348	1	20	694	713	UCAUUGAGUGCAGCGUUGAU	AUCAAUGCUGCGCUCAAUGA	Cleavage	1
		pab-miR397	2	21.348	1	20	694	713	UCAUUGAGUGCAGCGUUGAC	AUCAAUGCUGCGCUCAAUGA	Cleavage	1
		csi-miR397	1	21.348	1	20	694	713	UCAUUGAGUGCAGCGUUGAU	AUCAAUGCUGCGCUCAAUGA	Cleavage	1
		tcc-miR397	1	21.348	1	20	694	713	UCAUUGAGUGCAGCGUUGAU	AUCAAUGCUGCGCUCAAUGA	Cleavage	1
		gma-miR397a	1	21.348	1	20	694	713	UCAUUGAGUGCAGCGUUGAU	AUCAAUGCUGCGCUCAAUGA	Cleavage	1
		gma-miR397b-5p	1	21.348	1	20	694	713	UCAUUGAGUGCAGCGUUGAU	AUCAAUGCUGCGCUCAAUGA	Cleavage	1
		ssl-miR397	1	21.348	1	20	694	713	UCAUUGAGUGCAGCGUUGAU	AUCAAUGCUGCGCUCAAUGA	Cleavage	1
		dpr-miR397	2	21.348	1	20	694	713	CCAUUGAGUGCAGCGUUGAU	AUCAAUGCUGCGCUCAAUGA	Cleavage	1
		mdm-miR397a	2	18.079	1	20	691	710	UUGAGUGCAGCGUUGAUGAA	UUGAUCAAUGCUGCGCUCAA	Cleavage	1
		mdm-miR397b	2	18.079	1	20	691	710	UUGAGUGCAGCGUUGAUGAA	UUGAUCAAUGCUGCGCUCAA	Cleavage	1

		cme-miR397	1	21.348	1	20	694	713	UCAUUGAGUGCAGCGUUGAU	AUCAAUGCUGCGCUCAAUGA	Cleavage	1
		mtr-miR397-5p	1	21.348	1	20	694	713	UCAUUGAGUGCAGCGUUGAU	AUCAAUGCUGCGCUCAAUGA	Cleavage	1
		bdi-miR397b-5p	2	19.098	1	21	691	711	AUUGAGUGCAGCGUUGAUGAA	UUGAUCAAUGCUGCGCUCAAU	Cleavage	1
		stu-miR397-5p	2	19.098	1	20	692	711	AUUGAGUGCAGCGUUGAUGA	UGAUCAAUGCUGCGCUCAAU	Cleavage	1
GaLAC02_3	Cotton_A_30645	ath-miR397a	1.5	17.664	1	20	694	713	UCAUUGAGUGCAGCGUUGAU	ACCAAUGCUGCACUCAAUGA	Cleavage	1
		osa-miR397a	1.5	17.664	1	20	694	713	UCAUUGAGUGCAGCGUUGAU	ACCAAUGCUGCACUCAAUGA	Cleavage	1
		osa-miR397b	2	17.664	1	20	694	713	UUAUUGAGUGCAGCGUUGAU	ACCAAUGCUGCACUCAAUGA	Cleavage	1
		ptc-miR397a	1.5	17.664	1	20	694	713	UCAUUGAGUGCAGCGUUGAU	ACCAAUGCUGCACUCAAUGA	Cleavage	1
		bna-miR397a	1.5	17.664	1	20	694	713	UCAUUGAGUGCAGCGUUGAU	ACCAAUGCUGCACUCAAUGA	Cleavage	1
		bna-miR397b	1.5	17.664	1	20	694	713	UCAUUGAGUGCAGCGUUGAU	ACCAAUGCUGCACUCAAUGA	Cleavage	1
		vvi-miR397a	1.5	17.664	1	20	694	713	UCAUUGAGUGCAGCGUUGAU	ACCAAUGCUGCACUCAAUGA	Cleavage	1
		sbi-miR397-5p	1.5	17.664	1	20	694	713	UCAUUGAGUGCAGCGUUGAU	ACCAAUGCUGCACUCAAUGA	Cleavage	1
		bdi-miR397a	1.5	17.664	1	20	694	713	UCAUUGAGUGCAGCGUUGAU	ACCAAUGCUGCACUCAAUGA	Cleavage	1
		rco-miR397	1.5	17.664	1	20	694	713	UCAUUGAGUGCAGCGUUGAU	ACCAAUGCUGCACUCAAUGA	Cleavage	1
		aly-miR397a-5p	1.5	17.664	1	20	694	713	UCAUUGAGUGCAGCGUUGAU	ACCAAUGCUGCACUCAAUGA	Cleavage	1
		aly-miR397b-5p	1.5	17.664	1	20	694	713	UCAUUGAGUGCAGCGUUGAU	ACCAAUGCUGCACUCAAUGA	Cleavage	1
		csi-miR397	1.5	17.664	1	20	694	713	UCAUUGAGUGCAGCGUUGAU	ACCAAUGCUGCACUCAAUGA	Cleavage	1
		tcc-miR397	1.5	17.664	1	20	694	713	UCAUUGAGUGCAGCGUUGAU	ACCAAUGCUGCACUCAAUGA	Cleavage	1
		gma-miR397a	1.5	17.664	1	20	694	713	UCAUUGAGUGCAGCGUUGAU	ACCAAUGCUGCACUCAAUGA	Cleavage	1
		gma-miR397b-5p	1.5	17.664	1	20	694	713	UCAUUGAGUGCAGCGUUGAU	ACCAAUGCUGCACUCAAUGA	Cleavage	1

		ssl-miR397	1.5	17.664	1	20	694	713	UCAUUGAGUGCAGCGUUGAU	ACCAAUGCUGCACUCAAUGA	Cleavage	1
		cme-miR397	1.5	17.664	1	20	694	713	UCAUUGAGUGCAGCGUUGAU	ACCAAUGCUGCACUCAAUGA	Cleavage	1
		mtr-miR397-5p	1.5	17.664	1	20	694	713	UCAUUGAGUGCAGCGUUGAU	ACCAAUGCUGCACUCAAUGA	Cleavage	1
GaLAC02_4	Cotton_A_30646	ath-miR397a	2	19.835	1	20	694	713	UCAUUGAGUGCAGCGUUGAU	AUAAAUGCUGCGCUCAAUGA	Cleavage	1
		osa-miR397a	2	19.835	1	20	694	713	UCAUUGAGUGCAGCGUUGAU	AUAAAUGCUGCGCUCAAUGA	Cleavage	1
		ptc-miR397a	2	19.835	1	20	694	713	UCAUUGAGUGCAGCGUUGAU	AUAAAUGCUGCGCUCAAUGA	Cleavage	1
		bna-miR397a	2	19.835	1	20	694	713	UCAUUGAGUGCAGCGUUGAU	AUAAAUGCUGCGCUCAAUGA	Cleavage	1
		bna-miR397b	2	19.835	1	20	694	713	UCAUUGAGUGCAGCGUUGAU	AUAAAUGCUGCGCUCAAUGA	Cleavage	1
		vvi-miR397a	2	19.835	1	20	694	713	UCAUUGAGUGCAGCGUUGAU	AUAAAUGCUGCGCUCAAUGA	Cleavage	1
		sbi-miR397-5p	2	19.835	1	20	694	713	UCAUUGAGUGCAGCGUUGAU	AUAAAUGCUGCGCUCAAUGA	Cleavage	1
		bdi-miR397a	2	19.835	1	20	694	713	UCAUUGAGUGCAGCGUUGAU	AUAAAUGCUGCGCUCAAUGA	Cleavage	1
		zma-miR397a-5p	1.5	19.835	1	20	694	713	UCAUUGAGCGCAGCGUUGAU	AUAAAUGCUGCGCUCAAUGA	Cleavage	1
		zma-miR397b-5p	1.5	19.835	1	20	694	713	UCAUUGAGCGCAGCGUUGAU	AUAAAUGCUGCGCUCAAUGA	Cleavage	1
		rco-miR397	2	19.835	1	20	694	713	UCAUUGAGUGCAGCGUUGAU	AUAAAUGCUGCGCUCAAUGA	Cleavage	1
		aly-miR397a-5p	2	19.835	1	20	694	713	UCAUUGAGUGCAGCGUUGAU	AUAAAUGCUGCGCUCAAUGA	Cleavage	1
		aly-miR397b-5p	2	19.835	1	20	694	713	UCAUUGAGUGCAGCGUUGAU	AUAAAUGCUGCGCUCAAUGA	Cleavage	1
		csi-miR397	2	19.835	1	20	694	713	UCAUUGAGUGCAGCGUUGAU	AUAAAUGCUGCGCUCAAUGA	Cleavage	1
		tcc-miR397	2	19.835	1	20	694	713	UCAUUGAGUGCAGCGUUGAU	AUAAAUGCUGCGCUCAAUGA	Cleavage	1
		gma-miR397a	2	19.835	1	20	694	713	UCAUUGAGUGCAGCGUUGAU	AUAAAUGCUGCGCUCAAUGA	Cleavage	1
		gma-miR397b-5p	2	19.835	1	20	694	713	UCAUUGAGUGCAGCGUUGAU	AUAAAUGCUGCGCUCAAUGA	Cleavage	1

		ssl-miR397	2	19.835	1	20	694	713	UCAUUGAGUGCAGCGUUGAU	AUAAAUGCUGCGCUCAAUGA	Cleavage	1
		cme-miR397	2	19.835	1	20	694	713	UCAUUGAGUGCAGCGUUGAU	AUAAAUGCUGCGCUCAAUGA	Cleavage	1
		mtr-miR397-5p	2	19.835	1	20	694	713	UCAUUGAGUGCAGCGUUGAU	AUAAAUGCUGCGCUCAAUGA	Cleavage	1
GaLAC03_1	Cotton_A_04178	stu-miR408a-3p	1	16.079	1	21	131	151	UGCACAGCCUCUUCCCUGGUU	AACCAGUGAAGAGGCUGUGCA	Cleavage	1
GaLAC03_2	Cotton_A_24290	stu-miR408a-3p	1.5	20.767	1	21	131	151	UGCACAGCCUCUUCCCUGGUU	AGCCAGUGAAGAGGCUGUGCA	Cleavage	1
		ath-miR397a	1	16.136	1	21	663	683	UCAUUGAGUGCAGCGUUGAUG	CAUCAAUGCUGCACUUAAUGA	Cleavage	1
		ath-miR397b	2	16.136	1	21	663	683	UCAUUGAGUGCAUCGUUGAUG	CAUCAAUGCUGCACUUAAUGA	Cleavage	1
		osa-miR397a	1	16.136	1	21	663	683	UCAUUGAGUGCAGCGUUGAUG	CAUCAAUGCUGCACUUAAUGA	Cleavage	1
		osa-miR397b	1.5	16.136	1	21	663	683	UUAUUGAGUGCAGCGUUGAUG	CAUCAAUGCUGCACUUAAUGA	Cleavage	1
		ptc-miR397a	1	16.136	1	21	663	683	UCAUUGAGUGCAGCGUUGAUG	CAUCAAUGCUGCACUUAAUGA	Cleavage	1
		ptc-miR397b	2	16.136	1	21	663	683	CCAUUGAGUGCAGCGUUGAUG	CAUCAAUGCUGCACUUAAUGA	Cleavage	1
		bna-miR397a	1	16.136	1	21	663	683	UCAUUGAGUGCAGCGUUGAUG	CAUCAAUGCUGCACUUAAUGA	Cleavage	1
		bna-miR397b	1	16.136	1	21	663	683	UCAUUGAGUGCAGCGUUGAUG	CAUCAAUGCUGCACUUAAUGA	Cleavage	1
		vvi-miR397a	1	16.136	1	21	663	683	UCAUUGAGUGCAGCGUUGAUG	CAUCAAUGCUGCACUUAAUGA	Cleavage	1
		sly-miR397	1	15.989	1	20	662	681	AUUGAGUGCAGCGUUGAUGA	UCAUCAAUGCUGCACUUAAU	Cleavage	1
		sbi-miR397-5p	1	16.136	1	21	663	683	UCAUUGAGUGCAGCGUUGAUG	CAUCAAUGCUGCACUUAAUGA	Cleavage	1
		bdi-miR397a	1	16.136	1	21	663	683	UCAUUGAGUGCAGCGUUGAUG	CAUCAAUGCUGCACUUAAUGA	Cleavage	1
		zma-miR397a-5p	2	16.136	1	21	663	683	UCAUUGAGCGCAGCGUUGAUG	CAUCAAUGCUGCACUUAAUGA	Translation	1
		zma-miR397b-5p	2	16.136	1	21	663	683	UCAUUGAGCGCAGCGUUGAUG	CAUCAAUGCUGCACUUAAUGA	Translation	1
		rco-miR397	1	16.136	1	21	663	683	UCAUUGAGUGCAGCGUUGAUG	CAUCAAUGCUGCACUUAAUGA	Cleavage	1

GaLAC04_2	Cotton_A_05572	aly-miR397a-5p	1	16.136	1	21	663	683	UCAUUGAGUGCAGCGUUGAUG	CAUCAAUGCUGCACUUAAUGA	Cleavage	1
		aly-miR397b-5p	1	16.136	1	21	663	683	UCAUUGAGUGCAGCGUUGAUG	CAUCAAUGCUGCACUUAAUGA	Cleavage	1
		pab-miR397	2	16.136	1	21	663	683	UCAUUGAGUGCAGCGUUGACG	CAUCAAUGCUGCACUUAAUGA	Cleavage	1
		csi-miR397	1	16.136	1	21	663	683	UCAUUGAGUGCAGCGUUGAUG	CAUCAAUGCUGCACUUAAUGA	Cleavage	1
		tcc-miR397	1	16.136	1	21	663	683	UCAUUGAGUGCAGCGUUGAUG	CAUCAAUGCUGCACUUAAUGA	Cleavage	1
		gma-miR397a	1	16.136	1	21	663	683	UCAUUGAGUGCAGCGUUGAUG	CAUCAAUGCUGCACUUAAUGA	Cleavage	1
		gma-miR397b-5p	1	16.136	1	21	663	683	UCAUUGAGUGCAGCGUUGAUG	CAUCAAUGCUGCACUUAAUGA	Cleavage	1
		ssl-miR397	1	16.136	1	21	663	683	UCAUUGAGUGCAGCGUUGAUG	CAUCAAUGCUGCACUUAAUGA	Cleavage	1
		dpr-miR397	2	16.136	1	21	663	683	CCAUUGAGUGCAGCGUUGAUG	CAUCAAUGCUGCACUUAAUGA	Cleavage	1
		nta-miR397	2	15.989	1	20	662	681	AUUGAGUGCAGCGUUGAUGU	UCAUCAAUGCUGCACUUAAU	Cleavage	1
		mdm-miR397a	2	15.763	1	20	661	680	UUGAGUGCAGCGUUGAUGAA	CUCAUCAAUGCUGCACUUAA	Cleavage	1
		mdm-miR397b	2	15.763	1	20	661	680	UUGAGUGCAGCGUUGAUGAA	CUCAUCAAUGCUGCACUUAA	Cleavage	1
		cme-miR397	1	16.136	1	21	663	683	UCAUUGAGUGCAGCGUUGAUG	CAUCAAUGCUGCACUUAAUGA	Cleavage	1
		mes-miR397	2	15.989	1	20	662	681	UUUGAGUGCAGCGUUGAUGA	UCAUCAAUGCUGCACUUAAU	Cleavage	1
		lja-miR397	1.5	16.094	1	21	662	682	UAUUGAGUGCAGCGUUGAUGA	UCAUCAAUGCUGCACUUAAUG	Cleavage	1
		mtr-miR397-5p	1	16.136	1	21	663	683	UCAUUGAGUGCAGCGUUGAUG	CAUCAAUGCUGCACUUAAUGA	Cleavage	1
		bdi-miR397b-5p	1	15.989	1	20	662	681	AUUGAGUGCAGCGUUGAUGA	UCAUCAAUGCUGCACUUAAU	Cleavage	1
		stu-miR397-5p	1	15.989	1	20	662	681	AUUGAGUGCAGCGUUGAUGA	UCAUCAAUGCUGCACUUAAU	Cleavage	1
		ath-miR397a	0	13.294	1	20	664	683	UCAUUGAGUGCAGCGUUGAU	AUCAACGCUGCACUCAAUGA	Cleavage	1
		ath-miR397b	1	13.294	1	20	664	683	UCAUUGAGUGCAUCGUUGAU	AUCAACGCUGCACUCAAUGA	Cleavage	1

GaLAC04_3	Cotton_A_06597	osa-miR397a	0	13.294	1	20	664	683	UCAUUGAGUGCAGCGUUGAU	AUCAACGCUGCACUCAAUGA	Cleavage	1
		osa-miR397b	0.5	13.294	1	20	664	683	UUAUUGAGUGCAGCGUUGAU	AUCAACGCUGCACUCAAUGA	Cleavage	1
		ptc-miR397a	0	13.294	1	20	664	683	UCAUUGAGUGCAGCGUUGAU	AUCAACGCUGCACUCAAUGA	Cleavage	1
		ptc-miR397b	1	13.294	1	20	664	683	CCAUUGAGUGCAGCGUUGAU	AUCAACGCUGCACUCAAUGA	Cleavage	1
		bna-miR397a	0	13.294	1	20	664	683	UCAUUGAGUGCAGCGUUGAU	AUCAACGCUGCACUCAAUGA	Cleavage	1
		bna-miR397b	0	13.294	1	20	664	683	UCAUUGAGUGCAGCGUUGAU	AUCAACGCUGCACUCAAUGA	Cleavage	1
		vvi-miR397a	0	13.294	1	20	664	683	UCAUUGAGUGCAGCGUUGAU	AUCAACGCUGCACUCAAUGA	Cleavage	1
		sly-miR397	1	13.099	1	20	662	681	AUUGAGUGCAGCGUUGAUGA	UAAUCAACGCUGCACUCAAU	Cleavage	1
		sbi-miR397-5p	0	13.294	1	20	664	683	UCAUUGAGUGCAGCGUUGAU	AUCAACGCUGCACUCAAUGA	Cleavage	1
		bdi-miR397a	0	13.294	1	20	664	683	UCAUUGAGUGCAGCGUUGAU	AUCAACGCUGCACUCAAUGA	Cleavage	1
		zma-miR397a-5p	1	13.294	1	20	664	683	UCAUUGAGCGCAGCGUUGAU	AUCAACGCUGCACUCAAUGA	Translation	1
		zma-miR397b-5p	1	13.294	1	20	664	683	UCAUUGAGCGCAGCGUUGAU	AUCAACGCUGCACUCAAUGA	Translation	1
		rco-miR397	0	13.294	1	20	664	683	UCAUUGAGUGCAGCGUUGAU	AUCAACGCUGCACUCAAUGA	Cleavage	1
		aly-miR397a-5p	0	13.294	1	20	664	683	UCAUUGAGUGCAGCGUUGAU	AUCAACGCUGCACUCAAUGA	Cleavage	1
		aly-miR397b-5p	0	13.294	1	20	664	683	UCAUUGAGUGCAGCGUUGAU	AUCAACGCUGCACUCAAUGA	Cleavage	1
		pab-miR397	1	13.294	1	20	664	683	UCAUUGAGUGCAGCGUUGAC	AUCAACGCUGCACUCAAUGA	Cleavage	1
		hvu-miR397	1.5	13.294	1	20	664	683	CCGUUGAGUGCAGCGUUGAU	AUCAACGCUGCACUCAAUGA	Cleavage	1
		csi-miR397	0	13.294	1	20	664	683	UCAUUGAGUGCAGCGUUGAU	AUCAACGCUGCACUCAAUGA	Cleavage	1
		tcc-miR397	0	13.294	1	20	664	683	UCAUUGAGUGCAGCGUUGAU	AUCAACGCUGCACUCAAUGA	Cleavage	1
		gma-miR397a	0	13.294	1	20	664	683	UCAUUGAGUGCAGCGUUGAU	AUCAACGCUGCACUCAAUGA	Cleavage	1

		gma-miR397b-5p	0	13.294	1	20	664	683	UCAUUGAGUGCAGCGUUGAU	AUCAACGCUGCACUCAAUGA	Cleavage	1
		ssl-miR397	0	13.294	1	20	664	683	UCAUUGAGUGCAGCGUUGAU	AUCAACGCUGCACUCAAUGA	Cleavage	1
		dpr-miR397	1	13.294	1	20	664	683	CCAUUGAGUGCAGCGUUGAU	AUCAACGCUGCACUCAAUGA	Cleavage	1
		nta-miR397	2	13.099	1	20	662	681	AUUGAGUGCAGCGUUGAUGU	UAAUCAACGCUGCACUCAAU	Cleavage	1
		mdm-miR397a	2	14.328	1	20	661	680	UUGAGUGCAGCGUUGAUGAA	CUAAUCAACGCUGCACUCAA	Cleavage	1
		mdm-miR397b	2	14.328	1	20	661	680	UUGAGUGCAGCGUUGAUGAA	CUAAUCAACGCUGCACUCAA	Cleavage	1
		cme-miR397	0	13.294	1	20	664	683	UCAUUGAGUGCAGCGUUGAU	AUCAACGCUGCACUCAAUGA	Cleavage	1
		mes-miR397	2	13.099	1	20	662	681	UUUGAGUGCAGCGUUGAUGA	UAAUCAACGCUGCACUCAAU	Cleavage	1
		lja-miR397	1.5	13.198	1	21	662	682	UAUUGAGUGCAGCGUUGAUGA	UAAUCAACGCUGCACUCAAUG	Cleavage	1
		mtr-miR397-5p	0	13.294	1	20	664	683	UCAUUGAGUGCAGCGUUGAU	AUCAACGCUGCACUCAAUGA	Cleavage	1
		bdi-miR397b-5p	1	13.099	1	20	662	681	AUUGAGUGCAGCGUUGAUGA	UAAUCAACGCUGCACUCAAU	Cleavage	1
		stu-miR397-5p	1	13.099	1	20	662	681	AUUGAGUGCAGCGUUGAUGA	UAAUCAACGCUGCACUCAAU	Cleavage	1
		ath-miR397a	1.5	12.992	1	21	669	689	UCAUUGAGUGCAGCGUUGAUG	CAUCAACGCUGCACUGAAUGA	Cleavage	1
		osa-miR397a	1.5	12.992	1	21	669	689	UCAUUGAGUGCAGCGUUGAUG	CAUCAACGCUGCACUGAAUGA	Cleavage	1
		osa-miR397b	2	12.992	1	21	669	689	UUAUUGAGUGCAGCGUUGAUG	CAUCAACGCUGCACUGAAUGA	Cleavage	1
		ptc-miR397a	1.5	12.992	1	21	669	689	UCAUUGAGUGCAGCGUUGAUG	CAUCAACGCUGCACUGAAUGA	Cleavage	1
		bna-miR397a	1.5	12.992	1	21	669	689	UCAUUGAGUGCAGCGUUGAUG	CAUCAACGCUGCACUGAAUGA	Cleavage	1
		bna-miR397b	1.5	12.992	1	21	669	689	UCAUUGAGUGCAGCGUUGAUG	CAUCAACGCUGCACUGAAUGA	Cleavage	1
		vvi-miR397a	1.5	12.992	1	21	669	689	UCAUUGAGUGCAGCGUUGAUG	CAUCAACGCUGCACUGAAUGA	Cleavage	1
		sly-miR397	1.5	12.031	1	20	668	687	AUUGAGUGCAGCGUUGAUGA	UCAUCAACGCUGCACUGAAU	Cleavage	1

GaLAC04_4	Cotton_A_ 12917	sbi-miR397-5p	1.5	12.992	1	21	669	689	UCAUUGAGUGCAG CGUUGAUG	CAUCAACGCUGCAC UGAAUGA	Cleavage	1
		bdi-miR397a	1.5	12.992	1	21	669	689	UCAUUGAGUGCAG CGUUGAUG	CAUCAACGCUGCAC UGAAUGA	Cleavage	1
		rco-miR397	1.5	12.992	1	21	669	689	UCAUUGAGUGCAG CGUUGAUG	CAUCAACGCUGCAC UGAAUGA	Cleavage	1
		aly-miR397a-5p	1.5	12.992	1	21	669	689	UCAUUGAGUGCAG CGUUGAUG	CAUCAACGCUGCAC UGAAUGA	Cleavage	1
		aly-miR397b-5p	1.5	12.992	1	21	669	689	UCAUUGAGUGCAG CGUUGAUG	CAUCAACGCUGCAC UGAAUGA	Cleavage	1
		csi-miR397	1.5	12.992	1	21	669	689	UCAUUGAGUGCAG CGUUGAUG	CAUCAACGCUGCAC UGAAUGA	Cleavage	1
		tcc-miR397	1.5	12.992	1	21	669	689	UCAUUGAGUGCAG CGUUGAUG	CAUCAACGCUGCAC UGAAUGA	Cleavage	1
		gma-miR397a	1.5	12.992	1	21	669	689	UCAUUGAGUGCAG CGUUGAUG	CAUCAACGCUGCAC UGAAUGA	Cleavage	1
		gma-miR397b-5p	1.5	12.992	1	21	669	689	UCAUUGAGUGCAG CGUUGAUG	CAUCAACGCUGCAC UGAAUGA	Cleavage	1
		ssl-miR397	1.5	12.992	1	21	669	689	UCAUUGAGUGCAG CGUUGAUG	CAUCAACGCUGCAC UGAAUGA	Cleavage	1
		cme-miR397	1.5	12.992	1	21	669	689	UCAUUGAGUGCAG CGUUGAUG	CAUCAACGCUGCAC UGAAUGA	Cleavage	1
		lja-miR397	2	12.558	1	21	668	688	UAUUGAGUGCAGC GUUGAUGA	UCAUCAACGCUGCA CUGAAUG	Cleavage	1
		mtr-miR397-5p	1.5	12.992	1	21	669	689	UCAUUGAGUGCAG CGUUGAUG	CAUCAACGCUGCAC UGAAUGA	Cleavage	1
		bdi-miR397b-5p	1.5	12.031	1	20	668	687	AUUGAGUGCAGCG UUGAUGA	UCAUCAACGCUGCA CUGAAU	Cleavage	1
		stu-miR397-5p	1.5	12.031	1	20	668	687	AUUGAGUGCAGCG UUGAUGA	UCAUCAACGCUGCA CUGAAU	Cleavage	1
		ath-miR397a	1.5	13.293	1	21	669	689	UCAUUGAGUGCAG CGUUGAUG	CAUCAACGCUGCAC UGAAUGA	Cleavage	1
		osa-miR397a	1.5	13.293	1	21	669	689	UCAUUGAGUGCAG CGUUGAUG	CAUCAACGCUGCAC UGAAUGA	Cleavage	1
		osa-miR397b	2	13.293	1	21	669	689	UUAUUGAGUGCAG CGUUGAUG	CAUCAACGCUGCAC UGAAUGA	Cleavage	1
		ptc-miR397a	1.5	13.293	1	21	669	689	UCAUUGAGUGCAG CGUUGAUG	CAUCAACGCUGCAC UGAAUGA	Cleavage	1
		bna-miR397a	1.5	13.293	1	21	669	689	UCAUUGAGUGCAG CGUUGAUG	CAUCAACGCUGCAC UGAAUGA	Cleavage	1

GaLAC04_6	Cotton_A_20282	bna-miR397b	1.5	13.293	1	21	669	689	UCAUUGAGUGCAGCGUUGAUG	CAUCAACGCUGCACUGAAUGA	Cleavage	1
		vvi-miR397a	1.5	13.293	1	21	669	689	UCAUUGAGUGCAGCGUUGAUG	CAUCAACGCUGCACUGAAUGA	Cleavage	1
		sly-miR397	1.5	12.833	1	20	668	687	AUUGAGUGCAGCGUUGAUGA	UCAUCAACGCUGCACUGAAU	Cleavage	1
		sbi-miR397-5p	1.5	13.293	1	21	669	689	UCAUUGAGUGCAGCGUUGAUG	CAUCAACGCUGCACUGAAUGA	Cleavage	1
		bdi-miR397a	1.5	13.293	1	21	669	689	UCAUUGAGUGCAGCGUUGAUG	CAUCAACGCUGCACUGAAUGA	Cleavage	1
		rco-miR397	1.5	13.293	1	21	669	689	UCAUUGAGUGCAGCGUUGAUG	CAUCAACGCUGCACUGAAUGA	Cleavage	1
		aly-miR397a-5p	1.5	13.293	1	21	669	689	UCAUUGAGUGCAGCGUUGAUG	CAUCAACGCUGCACUGAAUGA	Cleavage	1
		aly-miR397b-5p	1.5	13.293	1	21	669	689	UCAUUGAGUGCAGCGUUGAUG	CAUCAACGCUGCACUGAAUGA	Cleavage	1
		csi-miR397	1.5	13.293	1	21	669	689	UCAUUGAGUGCAGCGUUGAUG	CAUCAACGCUGCACUGAAUGA	Cleavage	1
		tcc-miR397	1.5	13.293	1	21	669	689	UCAUUGAGUGCAGCGUUGAUG	CAUCAACGCUGCACUGAAUGA	Cleavage	1
		gma-miR397a	1.5	13.293	1	21	669	689	UCAUUGAGUGCAGCGUUGAUG	CAUCAACGCUGCACUGAAUGA	Cleavage	1
		gma-miR397b-5p	1.5	13.293	1	21	669	689	UCAUUGAGUGCAGCGUUGAUG	CAUCAACGCUGCACUGAAUGA	Cleavage	1
		ssl-miR397	1.5	13.293	1	21	669	689	UCAUUGAGUGCAGCGUUGAUG	CAUCAACGCUGCACUGAAUGA	Cleavage	1
		cme-miR397	1.5	13.293	1	21	669	689	UCAUUGAGUGCAGCGUUGAUG	CAUCAACGCUGCACUGAAUGA	Cleavage	1
		lja-miR397	2	13.288	1	21	668	688	UAUUGAGUGCAGCGUUGAUGA	UCAUCAACGCUGCACUGAAUG	Cleavage	1
		mtr-miR397-5p	1.5	13.293	1	21	669	689	UCAUUGAGUGCAGCGUUGAUG	CAUCAACGCUGCACUGAAUGA	Cleavage	1
		bdi-miR397b-5p	1.5	12.833	1	20	668	687	AUUGAGUGCAGCGUUGAUGA	UCAUCAACGCUGCACUGAAU	Cleavage	1
		stu-miR397-5p	1.5	12.833	1	20	668	687	AUUGAGUGCAGCGUUGAUGA	UCAUCAACGCUGCACUGAAU	Cleavage	1
GaLAC05_4	Cotton_A_15837	stu-miR408a-3p	1	19.548	1	20	12	31	UGCACAGCCUCUUCCCUGGU	ACCAGUGAAGAGGCUGUGCA	Cleavage	1
		sly-miR397	2	10.921	1	20	596	615	AUUGAGUGCAGCGUUGAUGA	UUAUCAACGCUGCAGUCAAU	Cleavage	1

GaLAC07_1	Cotton_A_22687	bdi-miR397b-5p	2	10.921	1	20	596	615	AUUGAGUGCAGCGUUGAUGA	UUAUCAACGCUGCAGUCAAU	Cleavage	1
		stu-miR397-5p	2	10.921	1	20	596	615	AUUGAGUGCAGCGUUGAUGA	UUAUCAACGCUGCAGUCAAU	Cleavage	1
GaLAC07_2	Cotton_A_30033	hvu-miR397	1.5	12.505	1	20	667	686	CCGUUGAGUGCAGCGUUGAU	GUCAACACUGCACUCAACGG	Cleavage	1
		ath-miR397a	2	11.11	1	21	669	689	UCAUUGAGUGCAGCGUUGAUG	CAUCAAUGCUGCACUCAAUCA	Cleavage	1
		osa-miR397a	2	11.11	1	21	669	689	UCAUUGAGUGCAGCGUUGAUG	CAUCAAUGCUGCACUCAAUCA	Cleavage	1
		osa-miR397b	2	11.11	1	21	669	689	UUAUUGAGUGCAGCGUUGAUG	CAUCAAUGCUGCACUCAAUCA	Cleavage	1
		ptc-miR397a	2	11.11	1	21	669	689	UCAUUGAGUGCAGCGUUGAUG	CAUCAAUGCUGCACUCAAUCA	Cleavage	1
		bna-miR397a	2	11.11	1	21	669	689	UCAUUGAGUGCAGCGUUGAUG	CAUCAAUGCUGCACUCAAUCA	Cleavage	1
		bna-miR397b	2	11.11	1	21	669	689	UCAUUGAGUGCAGCGUUGAUG	CAUCAAUGCUGCACUCAAUCA	Cleavage	1
		vvi-miR397a	2	11.11	1	21	669	689	UCAUUGAGUGCAGCGUUGAUG	CAUCAAUGCUGCACUCAAUCA	Cleavage	1
		sly-miR397	0.5	11.91	1	20	668	687	AUUGAGUGCAGCGUUGAUGA	UCAUCAAUGCUGCACUCAAU	Cleavage	1
		sbi-miR397-5p	2	11.11	1	21	669	689	UCAUUGAGUGCAGCGUUGAUG	CAUCAAUGCUGCACUCAAUCA	Cleavage	1
		bdi-miR397a	2	11.11	1	21	669	689	UCAUUGAGUGCAGCGUUGAUG	CAUCAAUGCUGCACUCAAUCA	Cleavage	1
		rco-miR397	2	11.11	1	21	669	689	UCAUUGAGUGCAGCGUUGAUG	CAUCAAUGCUGCACUCAAUCA	Cleavage	1
		aly-miR397a-5p	2	11.11	1	21	669	689	UCAUUGAGUGCAGCGUUGAUG	CAUCAAUGCUGCACUCAAUCA	Cleavage	1
		aly-miR397b-5p	2	11.11	1	21	669	689	UCAUUGAGUGCAGCGUUGAUG	CAUCAAUGCUGCACUCAAUCA	Cleavage	1
GaLAC07_5	Cotton_A_35771	csi-miR397	2	11.11	1	21	669	689	UCAUUGAGUGCAGCGUUGAUG	CAUCAAUGCUGCACUCAAUCA	Cleavage	1
		tcc-miR397	2	11.11	1	21	669	689	UCAUUGAGUGCAGCGUUGAUG	CAUCAAUGCUGCACUCAAUCA	Cleavage	1
		gma-miR397a	2	11.11	1	21	669	689	UCAUUGAGUGCAGCGUUGAUG	CAUCAAUGCUGCACUCAAUCA	Cleavage	1
		gma-miR397b-5p	2	11.11	1	21	669	689	UCAUUGAGUGCAGCGUUGAUG	CAUCAAUGCUGCACUCAAUCA	Cleavage	1

		ssl-miR397	2	11.11	1	21	669	689	UCAUUGAGUGCAGCGUUGAUG	CAUCAAUGCUGCACUCAAUCA	Cleavage	1
		nta-miR397	1.5	11.91	1	20	668	687	AUUGAGUGCAGCGUUGAUGU	UCAUCAAUGCUGCACUCAAU	Cleavage	1
		mdm-miR397a	1.5	12.007	1	21	666	686	UUGAGUGCAGCGUUGAUGAAA	UAUCAUCAAUGCUGCACUCAA	Cleavage	1
		mdm-miR397b	1.5	12.007	1	21	666	686	UUGAGUGCAGCGUUGAUGAAA	UAUCAUCAAUGCUGCACUCAA	Cleavage	1
		cme-miR397	2	11.11	1	21	669	689	UCAUUGAGUGCAGCGUUGAUG	CAUCAAUGCUGCACUCAAUCA	Cleavage	1
		mes-miR397	1.5	11.91	1	20	668	687	UUUGAGUGCAGCGUUGAUGA	UCAUCAAUGCUGCACUCAAU	Cleavage	1
		lja-miR397	1.5	11.69	1	21	668	688	UAUUGAGUGCAGCGUUGAUGA	UCAUCAAUGCUGCACUCAAUC	Cleavage	1
		mtr-miR397-5p	2	11.11	1	21	669	689	UCAUUGAGUGCAGCGUUGAUG	CAUCAAUGCUGCACUCAAUCA	Cleavage	1
		bdi-miR397b-5p	0.5	11.91	1	20	668	687	AUUGAGUGCAGCGUUGAUGA	UCAUCAAUGCUGCACUCAAU	Cleavage	1
		stu-miR397-5p	0.5	11.91	1	20	668	687	AUUGAGUGCAGCGUUGAUGA	UCAUCAAUGCUGCACUCAAU	Cleavage	1
		ath-miR397a	0	16.824	1	21	687	707	UCAUUGAGUGCAGCGUUGAUG	UAUCAACGCUGCACUCAAUGA	Cleavage	1
		ath-miR397b	1	16.824	1	21	687	707	UCAUUGAGUGCAUCGUUGAUG	UAUCAACGCUGCACUCAAUGA	Cleavage	1
		osa-miR397a	0	16.824	1	21	687	707	UCAUUGAGUGCAGCGUUGAUG	UAUCAACGCUGCACUCAAUGA	Cleavage	1
		osa-miR397b	0.5	16.824	1	21	687	707	UUAUUGAGUGCAGCGUUGAUG	UAUCAACGCUGCACUCAAUGA	Cleavage	1
		ptc-miR397a	0	16.824	1	21	687	707	UCAUUGAGUGCAGCGUUGAUG	UAUCAACGCUGCACUCAAUGA	Cleavage	1
		ptc-miR397b	1	16.824	1	21	687	707	CCAUUGAGUGCAGCGUUGAUG	UAUCAACGCUGCACUCAAUGA	Cleavage	1
		bna-miR397a	0	16.824	1	21	687	707	UCAUUGAGUGCAGCGUUGAUG	UAUCAACGCUGCACUCAAUGA	Cleavage	1
		bna-miR397b	0	16.824	1	21	687	707	UCAUUGAGUGCAGCGUUGAUG	UAUCAACGCUGCACUCAAUGA	Cleavage	1
		vvi-miR397a	0	16.824	1	21	687	707	UCAUUGAGUGCAGCGUUGAUG	UAUCAACGCUGCACUCAAUGA	Cleavage	1
		sly-miR397	0.5	15.575	1	20	686	705	AUUGAGUGCAGCGUUGAUGA	UUAUCAACGCUGCACUCAAU	Cleavage	1

GaLAC11_1	Cotton_A_00882	sbi-miR397-5p	0	16.824	1	21	687	707	UCAUUGAGUGCAGCGUUGAUG	UAUCAACGCUGCACUCAAUGA	Cleavage	1
		bdi-miR397a	0	16.824	1	21	687	707	UCAUUGAGUGCAGCGUUGAUG	UAUCAACGCUGCACUCAAUGA	Cleavage	1
		zma-miR397a-5p	1	16.824	1	21	687	707	UCAUUGAGCGCAGCGUUGAUG	UAUCAACGCUGCACUCAAUGA	Translation	1
		zma-miR397b-5p	1	16.824	1	21	687	707	UCAUUGAGCGCAGCGUUGAUG	UAUCAACGCUGCACUCAAUGA	Translation	1
		rco-miR397	0	16.824	1	21	687	707	UCAUUGAGUGCAGCGUUGAUG	UAUCAACGCUGCACUCAAUGA	Cleavage	1
		aly-miR397a-5p	0	16.824	1	21	687	707	UCAUUGAGUGCAGCGUUGAUG	UAUCAACGCUGCACUCAAUGA	Cleavage	1
		aly-miR397b-5p	0	16.824	1	21	687	707	UCAUUGAGUGCAGCGUUGAUG	UAUCAACGCUGCACUCAAUGA	Cleavage	1
		pab-miR397	1	16.824	1	20	688	707	UCAUUGAGUGCAGCGUUGAC	AUCAACGCUGCACUCAAUGA	Cleavage	1
		hvu-miR397	1.5	16.824	1	21	687	707	CCGUUGAGUGCAGCGUUGAUG	UAUCAACGCUGCACUCAAUGA	Cleavage	1
		csi-miR397	0	16.824	1	21	687	707	UCAUUGAGUGCAGCGUUGAUG	UAUCAACGCUGCACUCAAUGA	Cleavage	1
		tcc-miR397	0	16.824	1	21	687	707	UCAUUGAGUGCAGCGUUGAUG	UAUCAACGCUGCACUCAAUGA	Cleavage	1
		gma-miR397a	0	16.824	1	21	687	707	UCAUUGAGUGCAGCGUUGAUG	UAUCAACGCUGCACUCAAUGA	Cleavage	1
		gma-miR397b-5p	0	16.824	1	21	687	707	UCAUUGAGUGCAGCGUUGAUG	UAUCAACGCUGCACUCAAUGA	Cleavage	1
		ssl-miR397	0	16.824	1	21	687	707	UCAUUGAGUGCAGCGUUGAUG	UAUCAACGCUGCACUCAAUGA	Cleavage	1
		dpr-miR397	1	16.824	1	21	687	707	CCAUUGAGUGCAGCGUUGAUG	UAUCAACGCUGCACUCAAUGA	Cleavage	1
		nta-miR397	1.5	15.575	1	20	686	705	AUUGAGUGCAGCGUUGAUGU	UUAUCAACGCUGCACUCAAU	Cleavage	1
		mdm-miR397a	1.5	14.666	1	20	685	704	UUGAGUGCAGCGUUGAUGAA	AUUAUCAACGCUGCACUCAA	Cleavage	1
		mdm-miR397b	1.5	14.666	1	20	685	704	UUGAGUGCAGCGUUGAUGAA	AUUAUCAACGCUGCACUCAA	Cleavage	1
		cme-miR397	0	16.824	1	21	687	707	UCAUUGAGUGCAGCGUUGAUG	UAUCAACGCUGCACUCAAUGA	Cleavage	1
		mes-miR397	1.5	15.575	1	20	686	705	UUUGAGUGCAGCGUUGAUGA	UUAUCAACGCUGCACUCAAU	Cleavage	1

		lja-miR397	1	16.63	1	21	686	706	UAUUGAGUGCAGCGUUGAUGA	UUAUCAACGCUGCACUCAAUG	Cleavage	1
		mtr-miR397-5p	0	16.824	1	21	687	707	UCAUUGAGUGCAGCGUUGAUG	UAUCAACGCUGCACUCAAUGA	Cleavage	1
		bdi-miR397b-5p	0.5	15.575	1	20	686	705	AUUGAGUGCAGCGUUGAUGA	UUAUCAACGCUGCACUCAAU	Cleavage	1
		stu-miR397-5p	0.5	15.575	1	20	686	705	AUUGAGUGCAGCGUUGAUGA	UUAUCAACGCUGCACUCAAU	Cleavage	1
GaLAC11_3	Cotton_A_19723	ath-miR397a	2	13.392	1	21	687	707	UCAUUGAGUGCAGCGUUGAUG	CAUCAAUGCUGCACUCAACGA	Cleavage	1
		osa-miR397a	2	13.392	1	21	687	707	UCAUUGAGUGCAGCGUUGAUG	CAUCAAUGCUGCACUCAACGA	Cleavage	1
		ptc-miR397a	2	13.392	1	21	687	707	UCAUUGAGUGCAGCGUUGAUG	CAUCAAUGCUGCACUCAACGA	Cleavage	1
		bna-miR397a	2	13.392	1	21	687	707	UCAUUGAGUGCAGCGUUGAUG	CAUCAAUGCUGCACUCAACGA	Cleavage	1
		bna-miR397b	2	13.392	1	21	687	707	UCAUUGAGUGCAGCGUUGAUG	CAUCAAUGCUGCACUCAACGA	Cleavage	1
		vvi-miR397a	2	13.392	1	21	687	707	UCAUUGAGUGCAGCGUUGAUG	CAUCAAUGCUGCACUCAACGA	Cleavage	1
		sly-miR397	1.5	12.29	1	20	686	705	AUUGAGUGCAGCGUUGAUGA	UCAUCAAUGCUGCACUCAAC	Cleavage	1
		sbi-miR397-5p	2	13.392	1	21	687	707	UCAUUGAGUGCAGCGUUGAUG	CAUCAAUGCUGCACUCAACGA	Cleavage	1
		bdi-miR397a	2	13.392	1	21	687	707	UCAUUGAGUGCAGCGUUGAUG	CAUCAAUGCUGCACUCAACGA	Cleavage	1
		rco-miR397	2	13.392	1	21	687	707	UCAUUGAGUGCAGCGUUGAUG	CAUCAAUGCUGCACUCAACGA	Cleavage	1
		aly-miR397a-5p	2	13.392	1	21	687	707	UCAUUGAGUGCAGCGUUGAUG	CAUCAAUGCUGCACUCAACGA	Cleavage	1
		aly-miR397b-5p	2	13.392	1	21	687	707	UCAUUGAGUGCAGCGUUGAUG	CAUCAAUGCUGCACUCAACGA	Cleavage	1
		hvu-miR397	1.5	13.392	1	21	687	707	CCGUUGAGUGCAGCGUUGAUG	CAUCAAUGCUGCACUCAACGA	Cleavage	1
		csi-miR397	2	13.392	1	21	687	707	UCAUUGAGUGCAGCGUUGAUG	CAUCAAUGCUGCACUCAACGA	Cleavage	1
		tcc-miR397	2	13.392	1	21	687	707	UCAUUGAGUGCAGCGUUGAUG	CAUCAAUGCUGCACUCAACGA	Cleavage	1
		gma-miR397a	2	13.392	1	21	687	707	UCAUUGAGUGCAGCGUUGAUG	CAUCAAUGCUGCACUCAACGA	Cleavage	1

		gma-miR397b-5p	2	13.392	1	21	687	707	UCAUUGAGUGCAGCGUUGAUG	CAUCAAUGCUGCACUCAACGA	Cleavage	1
		ssl-miR397	2	13.392	1	21	687	707	UCAUUGAGUGCAGCGUUGAUG	CAUCAAUGCUGCACUCAACGA	Cleavage	1
		mdm-miR397a	1.5	12.179	1	20	685	704	UUGAGUGCAGCGUUGAUGAA	AUCAUCAAUGCUGCACUCAA	Cleavage	1
		mdm-miR397b	1.5	12.179	1	20	685	704	UUGAGUGCAGCGUUGAUGAA	AUCAUCAAUGCUGCACUCAA	Cleavage	1
		cme-miR397	2	13.392	1	21	687	707	UCAUUGAGUGCAGCGUUGAUG	CAUCAAUGCUGCACUCAACGA	Cleavage	1
		mes-miR397	1.5	12.29	1	20	686	705	UUUGAGUGCAGCGUUGAUGA	UCAUCAAUGCUGCACUCAAC	Cleavage	1
		mtr-miR397-5p	2	13.392	1	21	687	707	UCAUUGAGUGCAGCGUUGAUG	CAUCAAUGCUGCACUCAACGA	Cleavage	1
		bdi-miR397b-5p	1.5	12.29	1	20	686	705	AUUGAGUGCAGCGUUGAUGA	UCAUCAAUGCUGCACUCAAC	Cleavage	1
		stu-miR397-5p	1.5	12.29	1	20	686	705	AUUGAGUGCAGCGUUGAUGA	UCAUCAAUGCUGCACUCAAC	Cleavage	1
GaLAC11_4	Cotton_A_26217	ath-miR397a	2	13.56	1	21	603	623	UCAUUGAGUGCAGCGUUGAUG	UAUCAAUGCUGCACUGAAUGA	Cleavage	1
		osa-miR397a	2	13.56	1	21	603	623	UCAUUGAGUGCAGCGUUGAUG	UAUCAAUGCUGCACUGAAUGA	Cleavage	1
		ptc-miR397a	2	13.56	1	21	603	623	UCAUUGAGUGCAGCGUUGAUG	UAUCAAUGCUGCACUGAAUGA	Cleavage	1
		bna-miR397a	2	13.56	1	21	603	623	UCAUUGAGUGCAGCGUUGAUG	UAUCAAUGCUGCACUGAAUGA	Cleavage	1
		bna-miR397b	2	13.56	1	21	603	623	UCAUUGAGUGCAGCGUUGAUG	UAUCAAUGCUGCACUGAAUGA	Cleavage	1
		vvi-miR397a	2	13.56	1	21	603	623	UCAUUGAGUGCAGCGUUGAUG	UAUCAAUGCUGCACUGAAUGA	Cleavage	1
		sbi-miR397-5p	2	13.56	1	21	603	623	UCAUUGAGUGCAGCGUUGAUG	UAUCAAUGCUGCACUGAAUGA	Cleavage	1
		bdi-miR397a	2	13.56	1	21	603	623	UCAUUGAGUGCAGCGUUGAUG	UAUCAAUGCUGCACUGAAUGA	Cleavage	1
		rco-miR397	2	13.56	1	21	603	623	UCAUUGAGUGCAGCGUUGAUG	UAUCAAUGCUGCACUGAAUGA	Cleavage	1
		aly-miR397a-5p	2	13.56	1	21	603	623	UCAUUGAGUGCAGCGUUGAUG	UAUCAAUGCUGCACUGAAUGA	Cleavage	1
		aly-miR397b-5p	2	13.56	1	21	603	623	UCAUUGAGUGCAGCGUUGAUG	UAUCAAUGCUGCACUGAAUGA	Cleavage	1

		csi-miR397	2	13.56	1	21	603	623	UCAUUGAGUGCAGCGUUGAUG	UAUCAAUGCUGCACUGAAUGA	Cleavage	1
		tcc-miR397	2	13.56	1	21	603	623	UCAUUGAGUGCAGCGUUGAUG	UAUCAAUGCUGCACUGAAUGA	Cleavage	1
		gma-miR397a	2	13.56	1	21	603	623	UCAUUGAGUGCAGCGUUGAUG	UAUCAAUGCUGCACUGAAUGA	Cleavage	1
		gma-miR397b-5p	2	13.56	1	21	603	623	UCAUUGAGUGCAGCGUUGAUG	UAUCAAUGCUGCACUGAAUGA	Cleavage	1
		ssl-miR397	2	13.56	1	21	603	623	UCAUUGAGUGCAGCGUUGAUG	UAUCAAUGCUGCACUGAAUGA	Cleavage	1
		cme-miR397	2	13.56	1	21	603	623	UCAUUGAGUGCAGCGUUGAUG	UAUCAAUGCUGCACUGAAUGA	Cleavage	1
		mtr-miR397-5p	2	13.56	1	21	603	623	UCAUUGAGUGCAGCGUUGAUG	UAUCAAUGCUGCACUGAAUGA	Cleavage	1
		ath-miR397a	1	14.269	1	21	486	506	UCAUUGAGUGCAGCGUUGAUG	UAUCAAUGUUGCACUCAAUGA	Cleavage	1
		ath-miR397b	1.5	14.269	1	21	486	506	UCAUUGAGUGCAUCGUUGAUG	UAUCAAUGUUGCACUCAAUGA	Cleavage	1
		osa-miR397a	1	14.269	1	21	486	506	UCAUUGAGUGCAGCGUUGAUG	UAUCAAUGUUGCACUCAAUGA	Cleavage	1
		osa-miR397b	1.5	14.269	1	21	486	506	UUAUUGAGUGCAGCGUUGAUG	UAUCAAUGUUGCACUCAAUGA	Cleavage	1
		ptc-miR397a	1	14.269	1	21	486	506	UCAUUGAGUGCAGCGUUGAUG	UAUCAAUGUUGCACUCAAUGA	Cleavage	1
		ptc-miR397b	2	14.269	1	21	486	506	CCAUUGAGUGCAGCGUUGAUG	UAUCAAUGUUGCACUCAAUGA	Cleavage	1
		bna-miR397a	1	14.269	1	21	486	506	UCAUUGAGUGCAGCGUUGAUG	UAUCAAUGUUGCACUCAAUGA	Cleavage	1
		bna-miR397b	1	14.269	1	21	486	506	UCAUUGAGUGCAGCGUUGAUG	UAUCAAUGUUGCACUCAAUGA	Cleavage	1
		vvi-miR397a	1	14.269	1	21	486	506	UCAUUGAGUGCAGCGUUGAUG	UAUCAAUGUUGCACUCAAUGA	Cleavage	1
		sly-miR397	1.5	13.407	1	20	485	504	AUUGAGUGCAGCGUUGAUGA	UUAUCAAUGUUGCACUCAAU	Cleavage	1
		sbi-miR397-5p	1	14.269	1	21	486	506	UCAUUGAGUGCAGCGUUGAUG	UAUCAAUGUUGCACUCAAUGA	Cleavage	1
		bdi-miR397a	1	14.269	1	21	486	506	UCAUUGAGUGCAGCGUUGAUG	UAUCAAUGUUGCACUCAAUGA	Cleavage	1
		zma-miR397a-5p	2	14.269	1	21	486	506	UCAUUGAGCGCAGCGUUGAUG	UAUCAAUGUUGCACUCAAUGA	Translation	1

GaLAC11_5	Cotton_A_26221	zma-miR397b-5p	2	14.269	1	21	486	506	UCAUUGAGCGCAGCGUUGAUG	UAUCAAUGUUGCACUCAAUGA	Translation	1
		rco-miR397	1	14.269	1	21	486	506	UCAUUGAGUGCAGCGUUGAUG	UAUCAAUGUUGCACUCAAUGA	Cleavage	1
		aly-miR397a-5p	1	14.269	1	21	486	506	UCAUUGAGUGCAGCGUUGAUG	UAUCAAUGUUGCACUCAAUGA	Cleavage	1
		aly-miR397b-5p	1	14.269	1	21	486	506	UCAUUGAGUGCAGCGUUGAUG	UAUCAAUGUUGCACUCAAUGA	Cleavage	1
		pab-miR397	2	14.269	1	20	487	506	UCAUUGAGUGCAGCGUUGAC	AUCAAUGUUGCACUCAAUGA	Cleavage	1
		csi-miR397	1	14.269	1	21	486	506	UCAUUGAGUGCAGCGUUGAUG	UAUCAAUGUUGCACUCAAUGA	Cleavage	1
		tcc-miR397	1	14.269	1	21	486	506	UCAUUGAGUGCAGCGUUGAUG	UAUCAAUGUUGCACUCAAUGA	Cleavage	1
		gma-miR397a	1	14.269	1	21	486	506	UCAUUGAGUGCAGCGUUGAUG	UAUCAAUGUUGCACUCAAUGA	Cleavage	1
		gma-miR397b-5p	1	14.269	1	21	486	506	UCAUUGAGUGCAGCGUUGAUG	UAUCAAUGUUGCACUCAAUGA	Cleavage	1
		ssl-miR397	1	14.269	1	21	486	506	UCAUUGAGUGCAGCGUUGAUG	UAUCAAUGUUGCACUCAAUGA	Cleavage	1
		dpr-miR397	2	14.269	1	21	486	506	CCAUUGAGUGCAGCGUUGAUG	UAUCAAUGUUGCACUCAAUGA	Cleavage	1
		cme-miR397	1	14.269	1	21	486	506	UCAUUGAGUGCAGCGUUGAUG	UAUCAAUGUUGCACUCAAUGA	Cleavage	1
		lja-miR397	2	13.824	1	21	485	505	UAUUGAGUGCAGCGUUGAUGA	UUAUCAAUGUUGCACUCAAUG	Cleavage	1
		mtr-miR397-5p	1	14.269	1	21	486	506	UCAUUGAGUGCAGCGUUGAUG	UAUCAAUGUUGCACUCAAUGA	Cleavage	1
		bdi-miR397b-5p	1.5	13.407	1	20	485	504	AUUGAGUGCAGCGUUGAUGA	UUAUCAAUGUUGCACUCAAU	Cleavage	1
		stu-miR397-5p	1.5	13.407	1	20	485	504	AUUGAGUGCAGCGUUGAUGA	UUAUCAAUGUUGCACUCAAU	Cleavage	1
GaLAC12	Cotton_A_31477	stu-miR408a-3p	1	14.929	1	20	12	31	UGCACAGCCUCUUCCCUGGU	ACCAGUGAAGAGGCUGUGCA	Cleavage	1
		ath-miR397a	2	13.389	1	20	688	707	UCAUUGAGUGCAGCGUUGAU	GUCAAUGCGGCACUCAAUGA	Cleavage	1
		osa-miR397a	2	13.389	1	20	688	707	UCAUUGAGUGCAGCGUUGAU	GUCAAUGCGGCACUCAAUGA	Cleavage	1
		ptc-miR397a	2	13.389	1	20	688	707	UCAUUGAGUGCAGCGUUGAU	GUCAAUGCGGCACUCAAUGA	Cleavage	1

		bna-miR397a	2	13.389	1	20	688	707	UCAUUGAGUGCAG CGUUGAU	GUCAAUGCGGCAC UCAAUGA	Cleavage	1
		bna-miR397b	2	13.389	1	20	688	707	UCAUUGAGUGCAG CGUUGAU	GUCAAUGCGGCAC UCAAUGA	Cleavage	1
		vvi-miR397a	2	13.389	1	20	688	707	UCAUUGAGUGCAG CGUUGAU	GUCAAUGCGGCAC UCAAUGA	Cleavage	1
		sbi-miR397-5p	2	13.389	1	20	688	707	UCAUUGAGUGCAG CGUUGAU	GUCAAUGCGGCAC UCAAUGA	Cleavage	1
		bdi-miR397a	2	13.389	1	20	688	707	UCAUUGAGUGCAG CGUUGAU	GUCAAUGCGGCAC UCAAUGA	Cleavage	1
		rco-miR397	2	13.389	1	20	688	707	UCAUUGAGUGCAG CGUUGAU	GUCAAUGCGGCAC UCAAUGA	Cleavage	1
GaLAC17_1	Cotton_A_ 00902	aly-miR397a-5p	2	13.389	1	20	688	707	UCAUUGAGUGCAG CGUUGAU	GUCAAUGCGGCAC UCAAUGA	Cleavage	1
		aly-miR397b-5p	2	13.389	1	20	688	707	UCAUUGAGUGCAG CGUUGAU	GUCAAUGCGGCAC UCAAUGA	Cleavage	1
		pab-miR397	1.5	13.389	1	20	688	707	UCAUUGAGUGCAG CGUUGAC	GUCAAUGCGGCAC UCAAUGA	Cleavage	1
		csi-miR397	2	13.389	1	20	688	707	UCAUUGAGUGCAG CGUUGAU	GUCAAUGCGGCAC UCAAUGA	Cleavage	1
		tcc-miR397	2	13.389	1	20	688	707	UCAUUGAGUGCAG CGUUGAU	GUCAAUGCGGCAC UCAAUGA	Cleavage	1
		gma-miR397a	2	13.389	1	20	688	707	UCAUUGAGUGCAG CGUUGAU	GUCAAUGCGGCAC UCAAUGA	Cleavage	1
		gma-miR397b-5p	2	13.389	1	20	688	707	UCAUUGAGUGCAG CGUUGAU	GUCAAUGCGGCAC UCAAUGA	Cleavage	1
		ssl-miR397	2	13.389	1	20	688	707	UCAUUGAGUGCAG CGUUGAU	GUCAAUGCGGCAC UCAAUGA	Cleavage	1
		cme-miR397	2	13.389	1	20	688	707	UCAUUGAGUGCAG CGUUGAU	GUCAAUGCGGCAC UCAAUGA	Cleavage	1
		mtr-miR397-5p	2	13.389	1	20	688	707	UCAUUGAGUGCAG CGUUGAU	GUCAAUGCGGCAC UCAAUGA	Cleavage	1
GaLAC17_2	Cotton_A_ 00905	pab-miR397	2	12.75	1	20	688	707	UCAUUGAGUGCAG CGUUGAC	GUCAAUGCGGCAC UUAAUGA	Cleavage	1
		sly-miR397	2	17.753	1	20	797	816	AUUGAGUGCAGCG UUGAUGA	UCGUCAAUGCUGC ACUCAAC	Cleavage	1
		pab-miR397	2	17.702	1	21	798	818	UCAUUGAGUGCAG CGUUGACG	CGUCAAUGCUGCA CUCAACGA	Cleavage	1
		hvu-miR397	2	17.702	1	21	798	818	CCGUUGAGUGCAG CGUUGAUG	CGUCAAUGCUGCA CUCAACGA	Cleavage	1

GaLAC17_5	Cotton_A_ 12054	mdm-miR397a	2	17.538	1	21	795	815	UUGAGUGCAGCGU UGAUGAAA	UCUCGUCAAUGCU GCACUCAA	Cleavage	1
		mdm-miR397b	2	17.538	1	21	795	815	UUGAGUGCAGCGU UGAUGAAA	UCUCGUCAAUGCU GCACUCAA	Cleavage	1
		mes-miR397	2	17.753	1	20	797	816	UUUGAGUGCAGCG UUGAUGA	UCGUCAAUGCUGC ACUCAAC	Cleavage	1
		bdi-miR397b-5p	2	17.753	1	20	797	816	AUUGAGUGCAGCG UUGAUGA	UCGUCAAUGCUGC ACUCAAC	Cleavage	1
		stu-miR397-5p	2	17.753	1	20	797	816	AUUGAGUGCAGCG UUGAUGA	UCGUCAAUGCUGC ACUCAAC	Cleavage	1

Supplementary Table S7. Primers used for qPCR analysis of laccase genes.

**Gene Name**	**Forward Primer (5' – 3')**	**Reverse Primer (5' – 3')**
GaLAC02_1	GTTGAAAGAAACACCGTTG	TGATGTGAATTTTTGTGTTTAAAAGG
GaLAC02_2	TGGATAGTGTTGGATGGAGAATTC	GCTTAGGCAAAATAACACACGGC
GaLAC02_3	TGGGGGTTAGGAATGGCATGG	TTATGGACGCATGTAAAGCTTGATG
GaLAC02_4	GCTTAGGAATGGCATGGATGGTG	AATTATGGACGCACGTAAAGCTTGTTG
GaLAC03_1	GCTTTCCTAGTTGAGAATGGGGTTGG	TGATAATGCCAAATAAGTTCCCTAGC
GaLAC03_2	GCTTTGCTAGTTGAAAACGGAGTTGG	GTGGAATTCAGGACGTATAACATTC
GaLAC04_1	CCGTGCTGATAATCCAGGAGTTTG	TCTTCCGAACTCGAAAATCTCGG
GaLAC04_2	CCATTTGGAAGTGCATACGAC	CATTCTCAACTCTTGCTTTGC
GaLAC04_3	GATGGCATTTTTGGTAGACAATG	TGTTATTCGACATTTACCCAAGAG
GaLAC04_4	TGGCATTTGTTGTGGATAATGGC	CTCTCTAAAATTTCCGTTTCTTTC
GaLAC04_5	TGCAGGGTGGACTGCAATAAG	TCTAACTCAGCATCTGATTCTCC
GaLAC04_6	ACCATTGGGGTTCCATCTGGTGGAT	CCTCAGCATTTTGGTTTGGTTA
GaLAC04_7	CGGATCCTAACCCTGCCAAGG	TAGAAATAAACATGGTTGCTGTCTG
GaLAC05_1	AATGGAATCACCCTTGGTAGTG	GGTTTATGCATTGCCACTTAGACAT
GaLAC05_3	CTTTCCTTGTCGAAAATGGAGTTGG	TTCCCAAATGCAGGGAAGAATTGG
GaLAC05_4	GAAACACAGTTGGAGTACCTGTTAATG	TTATTTTGCTTTCTTGAATTTTGG
GaLAC06_1	GGTTTATGCATTGCCACTTAGACAT	ACACCAAACGTATAGCCAAATAG
GaLAC06_2	GTGTTGATAGTGGAAAATGGAGAAGG	CAATCTTTACTTCTTTACATACACAAC
GaLAC07_1	GTTTATGCATTGCCACTTTGA	ATTTTAACTTGTAAAAAGAAGGTCAGG
GaLAC07_2	TTCATGGTTGAGAATGGACCAACTC	CGCGTTGTATAATTGGATATCATTAT
GaLAC07_3	TTCGTGGTTGAGAACGGACCAAC	GAATAGTGTCAGTGAAATCCTTTCC
GaLAC07_4	GCCTTCATTGTTGAGAACGGACCAACT	CAACAACAGAGTTTTGACTTTTGAC
GaLAC07_5	TTCGTGGTTGAGAACGGCCCAAC	ATTATTACGTCGACAATATCTTGC
GaLAC11_1	GATGGCCTTTGTGGTCGAGAACG	GGCATTATGATCATGGGATATTTTG
GaLAC11_2	AATCCAGGTGTTTGGTTTATGCATTG	TTTGCAGAACATACATATAGCATG
GaLAC11_3	ACCCGATCAGTCCATTTTGC	TTATATACCTTCCTCCTCATTGCAG
GaLAC12	TCCTGGTTGAGAATGGATTTGG	ATCTTTCATGACACCCGCTTTA
GaLAC14_1	GTTTTCATTGTGAGGAATGGTACCACC	TTAATAGCCTTGATGAAATAATAGTCC
GaLAC14_2	CGGGGTGTGGTATATGCATTGCCAT	CGTGTCTATTTGTTGTATCCTGGC
GaLAC14_3	TGGAGACTACAAGCAAATTATTGATGAC	AGGTATATCTTCTCAGAATCAACTTGC
GaLAC14_4	CAGGATGGGTTGCCCTCAG	CATGCTGGTTTCGGTGGT
GaLAC14_5	TGTGGTATATGCATTGCCATTTCG	TAATCCACTTTATTCAATGCTTGTAC
GaLAC14_6	TGATAGTGAAGAATGGCAGCAATC	TAAAGTTCAAACCTTAGCATGTGC
GaLAC14_7	GGTTTTCACACTGCCACTTC	CCTTGAATAGTCCTCTAACCTATCC
GaLAC15	GTTTTCATAGTAAAAGACGGAGACCAG	TTTGAATTTAAACCATGACCTAGCC
GaLAC17_1	TGGTTCATGCATTGCCATCTA	GCTCGCTTACTGGTTTATGTCC
GaLAC17_2	ATGGTTCATGCATTGCCATCTA	AATAAATTGCTTGCTGACTGTCC
GaLAC17_3	ATGGCTTGGATTGTCTTGGATGGTG	CAAAATAAAGCTCGCTTACTATCG
GaLAC17_4	GGCTTGAGGATGGCTTGGATTG	TGTTTTTGGTCAAACTGGACCCG
GaLAC17_5	AAGATGGCTTGGGTGGTCAACGACG	TAATCTATACCAAAATCTGCACCAG
GaHistone3b	GAAGCCTCATCGATACCGTC	GCAAAGGTTGGTGTCTTCAAA
